# Imaging cellular activity across all organs reveals body-wide circuits

**DOI:** 10.1038/s41586-026-10979-6

**Published:** 2026-09-09

**Authors:** Virginie M. S. Ruetten, Wei Zheng, Igor Siwanowicz, Brett D. Mensh, Mark Eddison, Amy Hu, Yunfeng Chi, Andrew L. Lemire, Caiying Guo, Mykola Kadobianskyi, Marc Renz, Sara Lelek-Greskovic, Yisheng He, Kari Close, Gudrun Ihrke, Aparna Dev, Alyson Petruncio, Yinan Wan, Rongwei Zhang, Mark C. Fishman, Florian Engert, Benjamin Judkewitz, Mikail Rubinov, Philipp J. Keller, Chie Satou, Guoqiang Yu, Paul W. Tillberg, Maneesh Sahani, Misha B. Ahrens

**Affiliations:** 1 Janelia Research Campus, HHMI, Ashburn, VA USA; 2 Gatsby Computational Neuroscience Unit, UCL, London, UK; 3 Virginia Tech, Blacksburg, VA USA; 4 Tsinghua University, Beijing, China; 5 Charité Universitätsmedizin Berlin, Berlin, Germany; 6 Harvard University, Cambridge, MA USA; 7 Biozentrum, University of Basel, Basel, Switzerland; 8 Vanderbilt University, Nashville, TN USA

**Keywords:** Neuroscience, Population dynamics, Physiology, Time-lapse imaging, Bioinformatics

## Abstract

An animal’s ability to survive and thrive—whether fleeing from danger, eating a meal, or fighting an infection—arises from the collective moment-to-moment activity of many interacting cell types throughout the body. Physiology seeks to elucidate these cellular interactions that span organs, cell types and timescales, but has been limited by the inability to record this time-varying cellular activity simultaneously throughout the entire body. Here we develop WHOLISTIC (WHole-Organism Live-Imaging System for recording Tissue and IntraCellular activity), a method to image second-timescale activity of cells across the entire vertebrate body at cellular resolution. WHOLISTIC advances and integrates volumetric fluorescence microscopy, machine learning, and pancellular transgenic expression of calcium sensors^1^, demonstrated in larval zebrafish, with proof of concept in adult *Danionella cerebrum*. To access information about the molecular and ultrastructural substrates for the measured dynamics, we advanced whole-body expansion microscopy^2^. At the cellular scale, body-wide screening revealed unexpected responses, including chondrocyte reactions to cold and meningeal responses to ketamine. At the organ scale, WHOLISTIC identified rhythmic travelling waves along the renal nephron. At the multi-organ scale, it revealed unknown muscle synergies and muscle–organ interactions. At the whole-organism scale, the method captured brainstem-controlled redistribution of body-wide blood flow. Combining optogenetics with WHOLISTIC enabled all-optical causal dissection of brain–body interactions. These advances establish a paradigm for systems biology that bridges cellular and organismal physiology, enabling comprehensive discovery across scales—from fundamental mechanisms to therapeutic targets.

## Abstract (web summary)

An imaging system developed to record cellular activity throughout the whole body of zebrafish captures cellular organ dynamics and identifies multiple distributed circuits.

## Main

Cells across an organism must continuously coordinate with one another to sustain life and adapt to changing external environments and alterations within the body. Homeostasis is maintained through a complex network of dynamic interactions. Disease can arise from breakdowns in these intercellular feedback mechanisms. Although biomedicine has identified key interactions, such as stress responses mediated by neuroendocrine signalling or neural pathways between the brain and the gut, a vast number of mechanisms of whole-organism function remain unknown^3^. Our understanding is limited by the challenges of accessing body-wide cellular dynamics, driving the need for technologies to measure, analyse and model body-wide control mechanisms at the cellular level.

Modern synergies between imaging technology and protein engineering allow for time-varying molecular signals to be recorded in tissues. This has caused revolutions in fields such as neuroscience through the imaging of calcium—a fast, universal intracellular messenger involved in a wide range of cellular processes, including neuronal action potentials^1^—and other signals including voltage and neuromodulators across many neurons simultaneously^4^. However, time-varying activity patterns of most cell types in the body have not yet been recorded.

For most vertebrate models, optical access to large, opaque tissues poses a currently insurmountable challenge to whole-body imaging. Transparent vertebrate animals such as young zebrafish and adult *Danionella cerebrum*^5^ overcome this barrier, making them uniquely suited as models for in vivo studies of cellular dynamics across the body, offering unparalleled access to the inner workings of evolutionarily conserved organs such as the liver, pancreas, gut, brain, and the immune and cardiovascular systems.

This study introduces WHOLISTIC, a platform for in vivo imaging of cellular calcium dynamics, generalizable to other molecular dynamics, across nearly all cells of transparent vertebrates, such as the young zebrafish. By extending and integrating pancellular transgenic lines expressing genetically encoded calcium indicators in almost all cells in the body, high-speed volumetric fluorescence imaging, and a suite of computational methods for registration and cell population analysis, along with whole-body expansion microscopy (WB-ExM), we captured, analysed and interpreted cellular activity across tissues and organ systems.

## Body-wide cellular calcium imaging

**Fig. 1:**
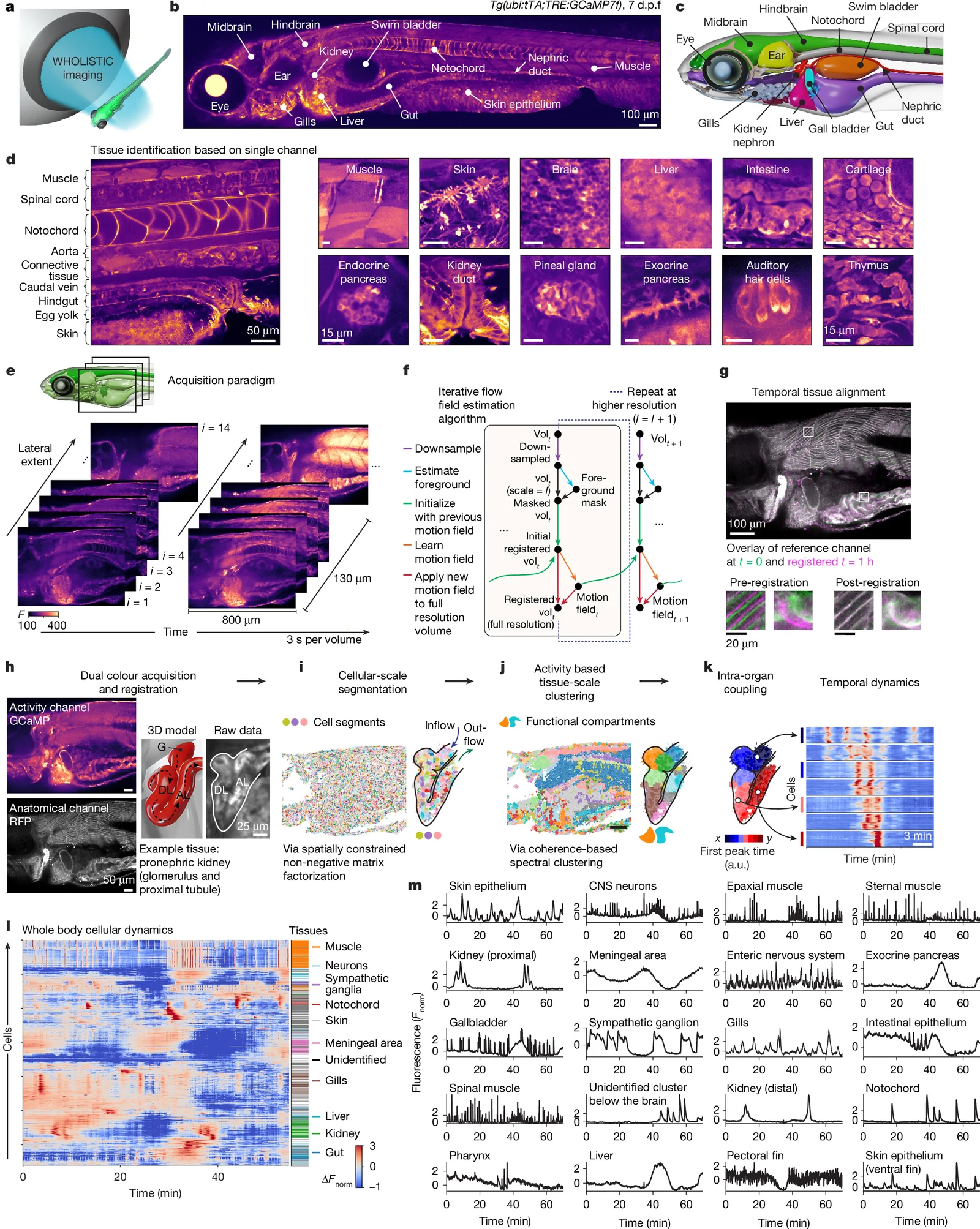
WHOLISTIC imaging of intracellular calcium dynamics across the body. **a**, Schematic of WHOLISTIC. **b**, Transgenic zebrafish (7 days post-fertilization) expressing GCaMP7f pancellularly (*Tg(ubi:tTA;TRE:GCaMP7f)*), imaged with spinning-disk confocal microscopy; samples were mounted laterally for visceral access. **c**, Annotated 3D anatomical model of young zebrafish derived from whole-body expansion microscopy (WB-ExM) data. **d**, Single fluorescence channel enables identification of organs and tissues via distinct visual textures (enlarged examples). **e**, Volumetric imaging acquisition paradigm with typical acquisition parameters. Fluorescence intensity variations reflect changes in intracellular calcium. Images covering an area of 800 μm × 600 μm are acquired in steps of approximately 9 μm, spanning a depth of approximately 130 μm using a ×20 0.75 NA objective. **f**, Registration workflow using multiscale iterative estimation of local motion fields. **g**, Registration results. Overlay of recording at the start (green) and 1 h into the experiment (magenta; top). The insets show myocytes and gut epithelium before (bottom left) and after (bottom right) registration. **h**, Dual-colour imaging (pancellular GCaMP plus membrane-targeted RFP; left), and example tissue (right), showing a 3D model and an enlarged view of the anterior part of pronephric kidney (glomerulus (G) and proximal tubules: descending limb (DL) and ascending limb (AL)). **i**, Cellular-scale segmentation (constrained non-negative matrix factorization). The inset shows an enlarged view of the segmentation results for the kidney nephron (1,265 cellular fragments). **j**, Coherence-based spectral clustering. The inset shows an enlarged view of clustering results for the kidney nephron (11 functional tissue ensembles). **k**, Nephron clusters ordered by time to first calcium activity peak within a burst (left), and raster of cellular activity within a single nephric burst, grouped by functional compartment and ordered within compartments using Rastermap (right)^68^. a.u., arbitrary units. **l**, Body-wide cellular activity raster ordered using Rastermap. On the right, a spectral cluster assignment is shown for some of the major organ groups. For visualization purposes, a random subset of 50 cells per cluster was included in the plot to reduce density of the figure, which was then ordered by Rastermap. **m**, Population-level dynamics. Mean activity of example functional tissue ensembles showing distinct dynamic profiles: high-frequency (for example, neurons and muscles), slower-evolving (for example, nephric tissue), tonic bistable (for example, sympathetic ganglion) and oscillatory (for example, enteric tissue) activity.

**Extended Data Fig. 1:**
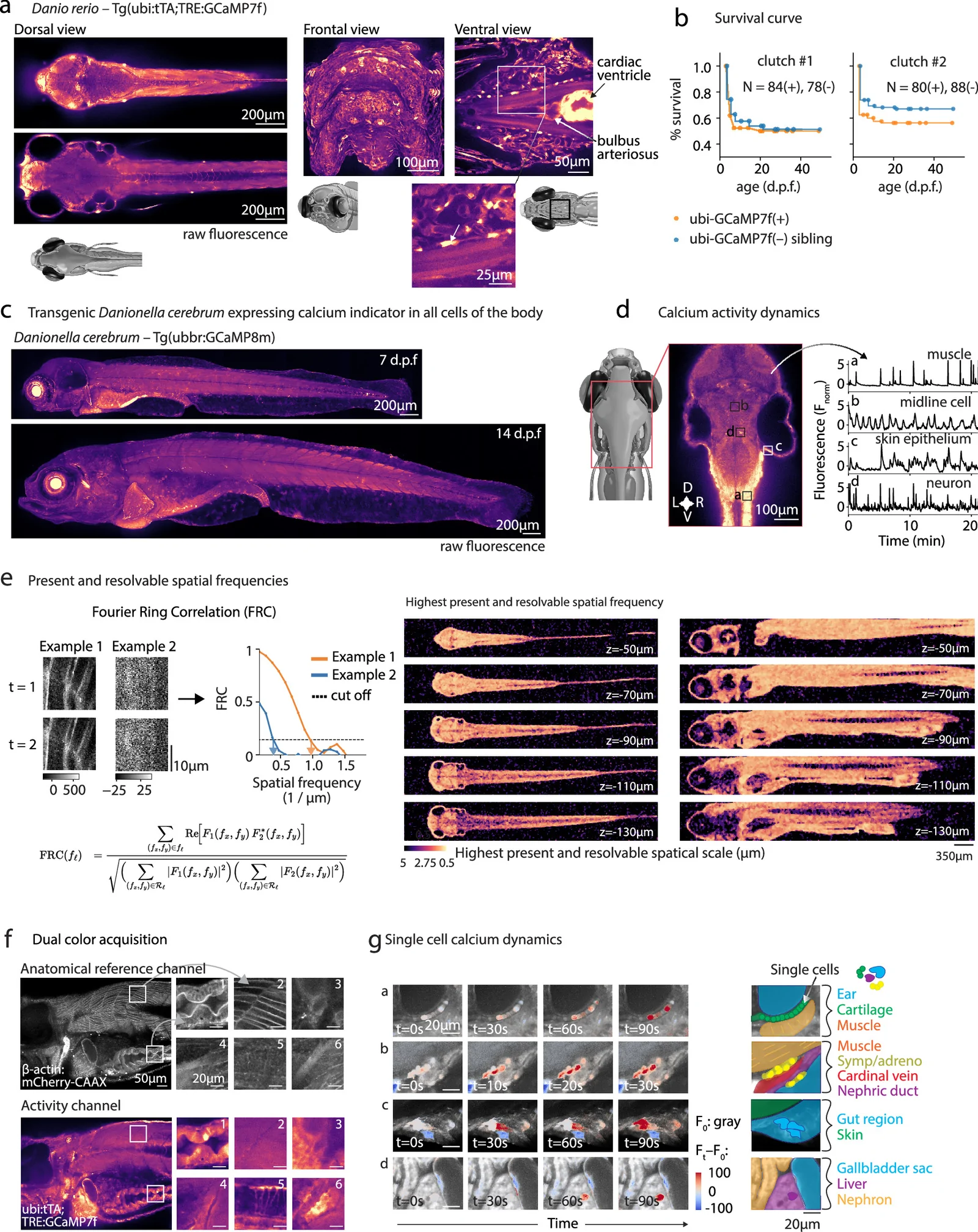
WHOLISTIC transgenics and workflow. **a**, Complementary views of WHOLISTIC zebrafish transgenic. *Left to right*: 1) dorsal view (two planes at different depths), 2) frontal view (maximum projection), 3) ventral views of gill and heart regions with cardiac ventricle and bulbus arteriosus labeled, along with enlarged view containing unidentified cell types. **b**, Survival curves for zebrafish pancellular GCaMP7f transgenic line. Embryos were segregated into GCaMP positive embryos (N = 84 and 80, represented by the orange line) and GCaMP negative siblings (N = 78 and 88 indicated by the blue line). **c**, WHOLISTIC for *Danionella* species. Pancellular GCaMP transgenic line. Sagittal view of *Tg(ubb*^*R*^:*jGCaMP8m)Danionella cerebrum* (7 and 14 days post fertilization), imaged using spinning-disk confocal microscopy. **d**, Calcium activity dynamics from *Danionella cerebrum* WHOLISTIC line. *Left*: dorsal view of brain. *Right*: example time-series from a) muscle, b) cell in midline of brain (unidentified), c) skin epithelium and d) neuron. **e**, Present and resolvable frequencies across the sample. *Left*: schematic of Fourier Ring Correlation (FRC), a method for quantifying the reliably resolvable spatial frequencies within an image. Two independent images of the same object are acquired, their 2D Fourier transforms computed, and the phase correlation calculated, radially averaged, and normalized. The spatial frequency at which the correlation falls below 1/7 is typically taken as the resolution cut-off. *Right*: spatial map of the highest spatial frequency at which the FRC remains above the 1/7 threshold. High spatial frequencies are detectable across most of the body, with localized reductions in regions between the ears and in deep pharyngeal areas between the gills. **f**, Single-cell dynamics across various body regions. Sequential frames displaying fluorescence intensity variations (blue-red) superimposed on anatomical reference (gray). Specific images depict *top to bottom*: 1) the ear with surrounding active chondrocytes; 2) the midline cardinal vein surrounded by active cells; 3) the ventral fin containing large active skin epithelial cells; 4) the liver displaying active hepatic cells. **g**, Volumetric tissue registration via dual-color imaging using double transgenic line (*Tg(ubi:tTA; TRE:GCaMP7f)*; *Tg(β*-*actin2:mCherry-CAAX)*) line. *Top*: anatomical reference channel showing membrane labeling. *Bottom*: activity channel. Insets showing enlarged views of: (1) gut, (2) muscle, (3) gallbladder sphincter, (4) blood vessel, (5) spinal cord, and (6) neural tract.

To image dynamic fluctuations of intracellular calcium across cell types of the body of young zebrafish (Fig. 1a), we engineered a pancellular transgenic zebrafish line that expresses the genetically encoded calcium indicator GCaMP7f under the control of the *ubiquitin* promoter^6,7^, *Tg(ubi:tTA; TRE:GCaMP7f)* (Fig. 1b,c). To avoid transient or sparse expression^8^, we used the binary expression system tTA–TRE to enhance the concentration of GCaMP7f^9^, which resulted in extensive and sustained pancellular expression with no observed pathological manifestations (Fig. 1b and Extended Data Fig. 1a,b). Here we chose to focus on intracellular calcium, given its broad involvement in numerous cellular processes across all cell types^1^. The approach generalizes to other sensors of voltage, membrane tension and other molecules.

The utility of pancellular imaging is contingent on the ability to discern organs and cell types within densely labelled tissue. The ubiquitous expression of GCaMP7f in tightly packed cells might have led to a conglomeration of indistinguishable cells; however, we found that major organs and tissue types could be distinguished by their distinct visual textures arising from variations in cell size, morphology, subcellular structure, baseline calcium and sensor expression level (Fig. 1d). Together, this enables the recognition of bodily tissues, including muscle, liver, kidney, brain and intestine, as well as smaller cellular populations such as the pineal gland and the endocrine pancreas (Fig. 1d), subsequently verified at a more detailed scale using WB-ExM (Methods). Furthermore, we developed a pancellular *Danionella cerebrum* GCaMP transgenic line to adapt WHOLISTIC to adult transparent vertebrates (Extended Data Fig. 1c,d). Thus, the expression of calcium sensors under the *ubiquitin* promoter has the potential to enable the monitoring of dynamic cellular signals throughout the organism, both developing and mature, while also affording the identification of tissue types, with or without the addition of cell-type-specific markers.

To acquire and process spatiotemporal WHOLISTIC data and extract interpretable cellular calcium activity time series that can be related to the tissues of origin, we developed a data collection and analysis workflow (Methods and Fig. 1e–j). Acquiring high-quality data across the body was more challenging than imaging the brain due to greater tissue inhomogeneities and complex geometry that increase scattering and diffraction, such that light-sheet microscopy^10,11^ was unsuitable. Among the microscopy techniques evaluated, spinning-disk confocal microscopy proved most robust to tissue scattering and diffraction (Extended Data Fig. 1e). As most major internal organs in zebrafish are located in the anterior third of the body, we opted to primarily image this anterior region from the side (Fig. 1e and Supplementary Videos 1 and 2).

**Extended Data Fig. 2:**
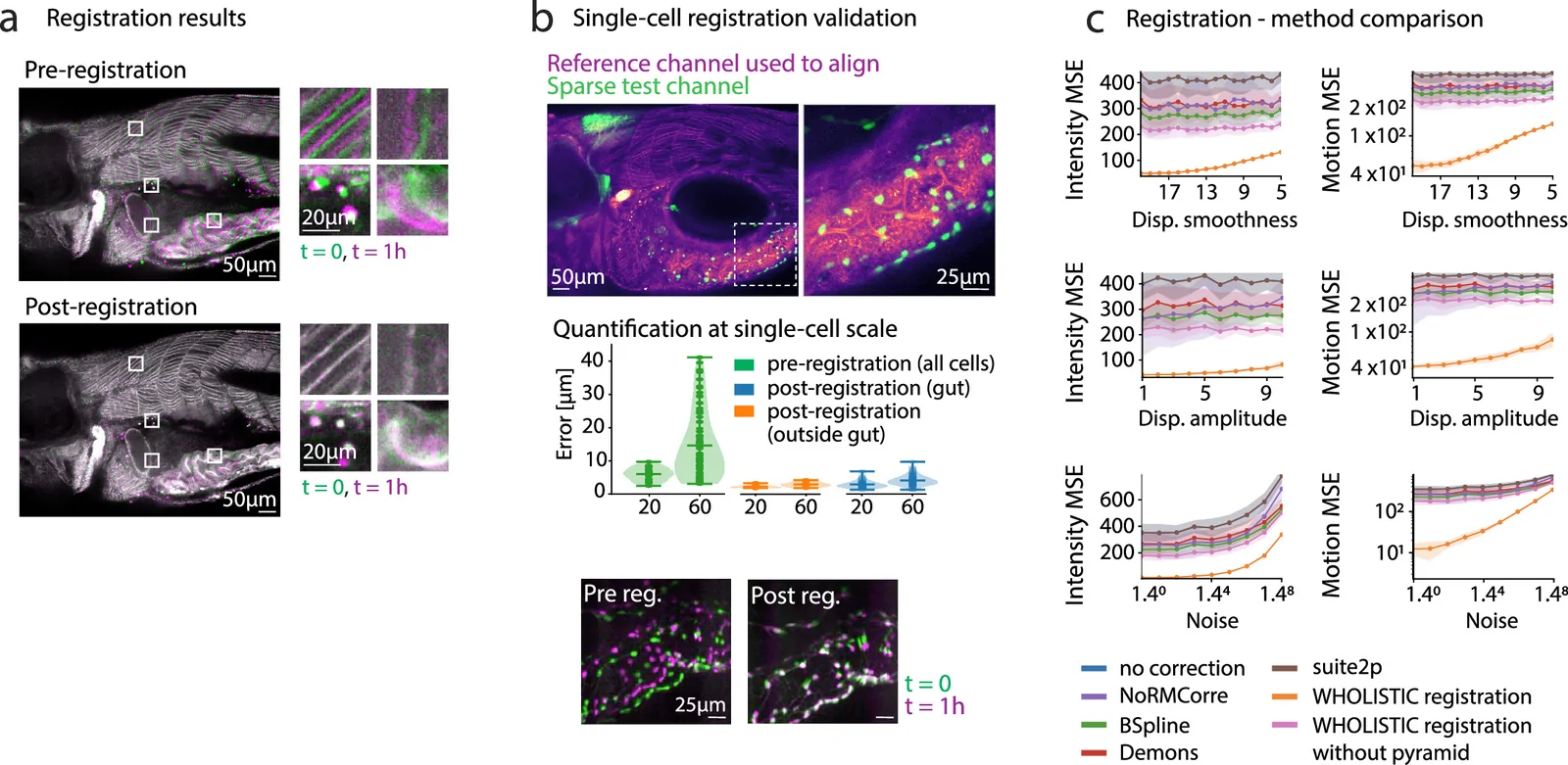
WHOLISTIC registration. **a**, Registration results. Overlay of data from the start of an experiment (green) and 1 hour into recording (magenta). *Top left*: pre-registration overlay showing significant drift. *Bottom left*: post-registration overlay showing alignment correction in the same regions and at the same timepoints. *Right*: enlarged views of muscle, gallbladder, skin, and gut regions pre- and post-registration. **b**, Validation of registration results. *Top left*: double transgenic line used for validation (*Tg(β*-*actin2:mCherry-CAAX; phox2bb:eGFP)*) with inset showing enlarged view of the gut - the most challenging region to register. *Top right*: overlay of sparse cell line at the start of the experiment (green) and 1 hour in (magenta), pre-registration (left) and post-registration (right). *Bottom*: violin plots of cellular displacement after 20 and 60 minutes: pre-registration displacement (green), post-registration displacement in gut (blue), and in the rest of the viscera and brain (orange). (Left to right violin plots: N = 35, 95, 30, 15, 45, 83 cells, all from one animal. Error bars show mean and extrema.) **c**, Registration method comparison. Synthetic datasets were generated by applying known motion fields to experimental images, varying displacement smoothness (*σ*_*k*_), amplitude (*A*), and noise (*σ*_*e*_*ta*) to test robustness. Each dataset was registered with six methods: WHOLISTIC registration, WHOLISTIC registration without pyramid scheme, B-spline, Demons, NoRMCorre, Suite2p. Mean squared error (MSE) between reference and registered pixel values was computed, as well as MSE of the motion vector field to penalize potential over-fitting.

**Extended Data Fig. 3:**
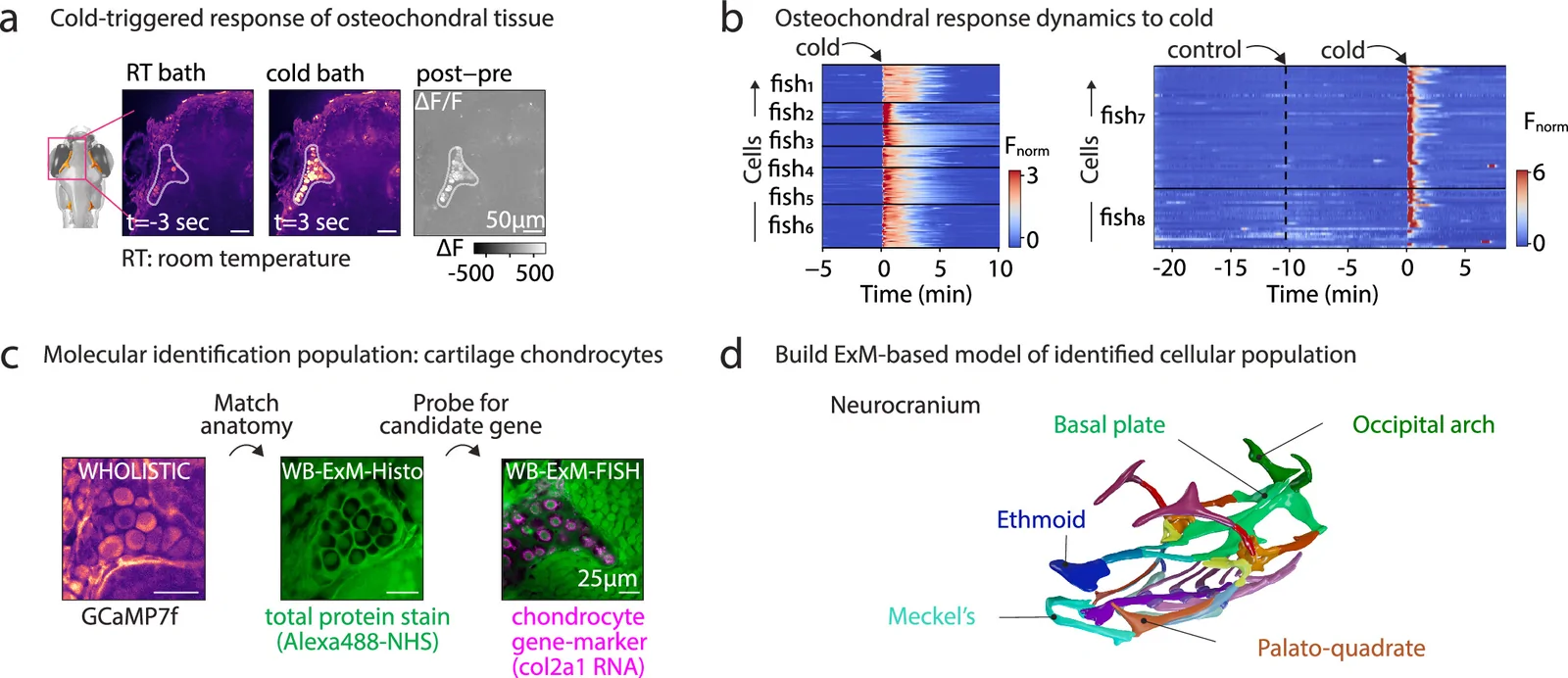
WHOLISTIC screening for cellular responses to stimuli. **a**, WHOLISTIC screen for cold-responsive cells. Following a 5-minute period of baseline recording, 10 °C water is introduced to the imaging chamber. Transverse section of the anterior portion of the cranium shown pre- and post-exposure to cold, and ΔF. Cold-responsive population outlined in gray. **b**, *Left*: raster of Ca^2+^ levels in osteochondral cells aligned to cold stimulus onset, showing time-locked responses in 6 animals. *Right*: control assay - raster of Ca^2+^ levels in osteochondral cells showing little to no time-locked responses to control stimulus (room temperature water) in 2 animals and responses to cold stimulus. **c**, Cell type identification and modeling via Whole-Body ExM. Matching cellular morphology between in vivo and ExM data. *Left to right*: 1) view of cold-responsive region in WHOLISTIC in vivo data, 2) corresponding region in WB-ExM-Histo data matched by location, cell morphology, and spatial distribution, using Alexa488-NHS (green), 3) corresponding region in WB-ExM-FISH sample stained against chondrocytic gene-marker, col2a1 (magenta). **d**, Reconstruction of the neurocranium - 3D model of young zebrafish cartilage generated from ExM data, annotated based on literature.

**Extended Data Fig. 4:**
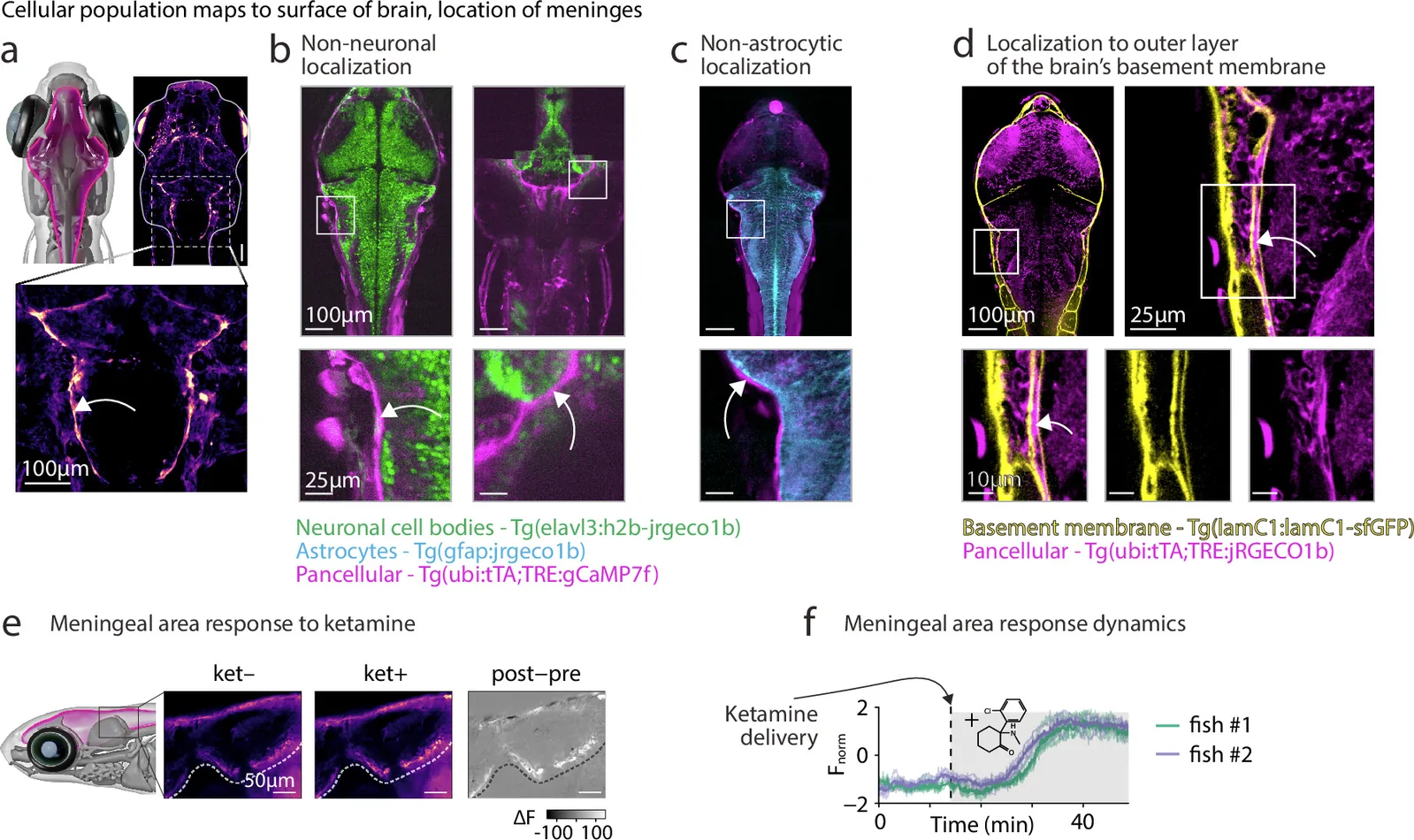
WHOLISTIC screening for cellular responses to drugs. **a**, Maximum intensity projection showing a brightly fluorescent cellular layer surrounding the brain (top). Enlarged view of the hindbrain (bottom). **b**, Border cells (visible among magenta cells) do not colocalize to neurons (green). Image of double transgenic line at dorsal (*left*) and ventral (*right*) planes (*Tg(elavl3:H2B-jRGECO1b; ubi:tTA;TRE:GCaMP7f)*) with inset showing enlarged view of border region. Contrast of magenta adjusted to highlight border cells. **c**, Border cells (visible among magenta cells) do not colocalize to astrocytes (blue). Image of double transgenic line (*Tg(gfap:jRGECO1b; ubi:tTA;TRE:GCaMP7f)*) showing non-colocalization of border cells and astrocytes, with inset showing enlarged view of border region. Contrast of magenta channel adjusted to highlight border cells. **d**, Border cells (visible among magenta cells) localize to the outer layer of the brain’s basement membrane (yellow). Image of double transgenic line (*Tg(lamC1:lamC1-sfGFP; ubi:tTA;TRE:jRGECO1b)*) showing intercalation of border cell and brain basement membrane, with inset showing enlarged view of border region. Contrast of magenta channel adjusted to highlight border cells. **e**, Meningeal area response to ketamine exposure. Animals were exposed to ketamine following a 25 min period of baseline imaging. Sagittal section showing pre- and post-exposure to ketamine, and ΔF. **f**, Activity traces from meningeal regions; population average in darker shade.

Smooth and skeletal muscle contractions throughout the body result in the displacement of cells (Supplementary Video 3), such that accurately extracting the activity of individual cells over time requires accounting for this cellular displacement. Although motion in the brain is relatively rigid, motion in the viscera is much less constrained, making standard registration algorithms ineffective or slow. To overcome this challenge, we first performed dual-colour imaging of calcium dynamics concurrently with an anatomical reference channel to aid alignment (Extended Data Fig. 1f,g). Next, we developed a registration algorithm based on iterative optical flow estimation^12^ that estimates motion patch-wise (11 × 11 pixels) and combines smoothness regularization, signal-to-noise weighting of gradients and multi-resolution registration to favour convergence to an optimal tissue alignment for each time point (Methods and Fig. 1f). This achieved high-quality alignment, and most cells were accurately registered throughout the experiments (Fig. 1g, Extended Data Fig. 2a–c and Supplementary Video 4). Subsequent analysis allowed for the identification of single-cell responses to externally applied stimuli, including previously unknown responses of cartilage chondrocytes to cold temperatures (Extended Data Fig. 3 and Supplementary Video 5) and responses to ketamine by cells located at the meningeal boundary of the brain (Extended Data Fig. 4). Thus, WHOLISTIC enables the analysis of spontaneous and stimulus-driven responses across the animal at the single-cell level.

### Identifying functional tissue ensembles

To extract cellular-scale calcium activity traces from registered imaging data (Fig. 1h), we used a segmentation method based on constrained non-negative matrix factorization (Voluseg)^13^ (Methods and Fig. 1i). We tuned this approach conservatively to minimize false mergers, over-parcellating the data into subcellular segments, that can later be aggregated.

We found that the majority of cells exhibit measurable, time-varying calcium dynamics even at rest (Fig. 1j–m and Supplementary Video 1). Although consistent with the known importance of calcium signalling, to our knowledge, it has never been demonstrated that most cell types across disparate organs exhibit measurable second-scale calcium fluctuations even in the absence of specific stimuli, nor that the dynamic range of GCaMP7f is sufficiently broad to capture them.

The utility of pancellular imaging increases with the ability to readily assign cells to their organs and cell types of origin. Although imaged volumes can be manually annotated with the identity of tissue types based solely on anatomical features (Fig. 1d), it is laborious, especially for sparse or highly distributed populations, and misses finer delineations within cellular populations that are defined not by their appearance but by their calcium activity patterns. We thus explored whether a more data-driven methodology, centred on the aggregation of cells with related activity patterns, could more effectively delineate functional anatomical boundaries within and across tissues. This would also provide a more compact, dimensionally reduced and interpretable representation of the data. To this end, we implemented spectral clustering^14^ and used a coherence-based similarity metric. Unlike correlation, coherence is invariant to phase lags and thus groups cells engaged in temporally structured activity such as travelling waves (Methods and Fig. 1j).

**Extended Data Fig. 5:**
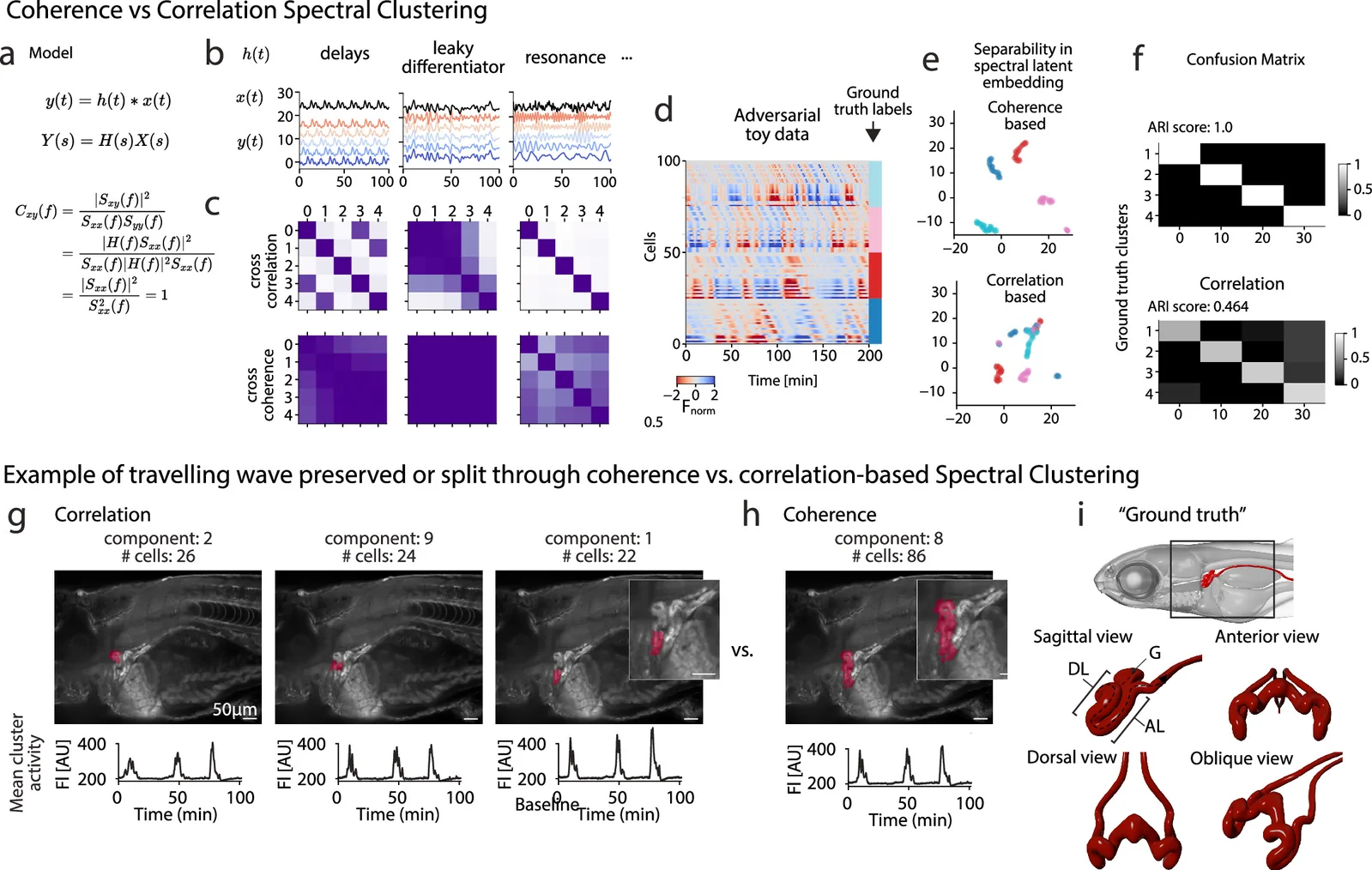
Coherence vs. correlation-based spectral clustering. **a**, Any two signals *y*(*t*), *x*(*t*) which are linearly filtered versions of each other will have a coherence of one. Definition of coherence. **b**, Example of time-series which are coherent but not necessarily correlated. Time-series *y*(*t*) are filtered versions of *x*(*t*), illustrating delay lines (left), leaky differentiators (center) and resonance (right). **c**, Cross-coherence vs. cross-correlation matrix of signals in b. Coherence better captures the relationship between these timeseries. **d**, Adversarial toy data constructed similarly as in b. The data consists of 4 clusters of noisy coherent timeseries. Left: ground truth labels of cluster identity. **e**, Spectral latent embedding. Embedding of timeseries using coherence-based clustering (top) or correlation-based clustering (bottom). **f**, Confusion matrix. Results of coherence vs. correlation-based clustering on adversarial toy data fit with 4 clusters. Values correspond to fraction of cells that were correctly assigned. ARI (adjusted Rand index): 1 for coherence, and 0.464 for correlation. **g**, Comparison of coherence vs. correlation-based spectral clustering. Using correlation as a metric, the descending nephron is grouped into three clusters. Anatomical footprint of clusters (red) superimposed on anatomical reference (gray). Mean cluster activity trace (bottom). **h**, Using coherence as a metric, the descending nephron is grouped into a single cluster. Anatomical footprint of clusters (red) superimposed on anatomical reference (gray). Mean cluster activity trace (bottom). **i**, Model of pronephric kidney (glomerulus and proximal tubules - G: glomerulus, DL: descending limb, AL: ascending limb) based on WB-ExM-Histo data viewed from multiple angles (same as in Fig. 1h).

**Extended Data Fig. 6:**
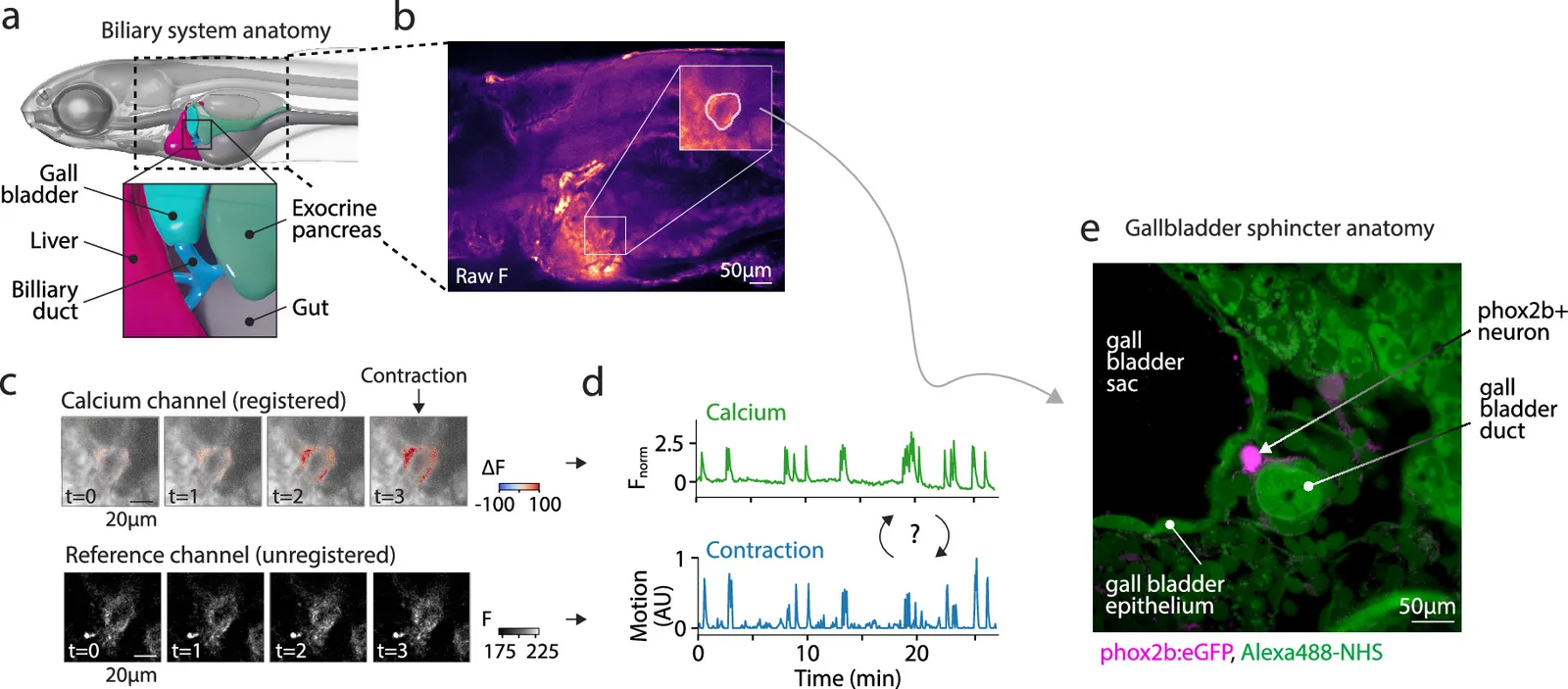
Example of functional tissue ensembles identified through WHOLISTIC coherence-based spectral clustering: biliary system. **a**, Contractile biliary sphincter with contraction-locked calcium activity bursts. ExM-based reconstruction of the hepato-biliary duct system. **b**, Sagittal view showing a sphincter at the base of the gallbladder sac, with an enlarged view and outline of the associated functional tissue ensemble. **c**, Calcium activity concurrent with sphincter contraction. *Top*: Registered Δ*F* (red) on anatomical reference (gray). *Lower*: Unregistered reference showing sphincter constriction. **d**, *Top*: average Ca^2+^ activity trace from the sphincter functional tissue ensemble. *Bottom*: average motion magnitude in the same region computed from the fitted motion field of the area. **e**, Visualization of gallbladder sphincter using WB-ExM. View of biliary system with gallbladder sac, epithelia and duct visible, along with local post-ganglionic vagal neuron. WB-ExM of transgenic fish labeling the autonomic nervous system (*Tg(phox2bb:eGFP)*, magenta, total protein stain Alexa488-NHS, green).

Examining the anatomical footprint of the spectral clusters, we found that they predominantly corresponded to sub-regions of individual tissue types, such as the kidney, gallbladder, liver, gut and muscle (Fig. 1j,k,m and Extended Data Figs. 5 and 6). This can be leveraged to organize the data into interpretable and readily identifiable clusters at the tissue level (Fig. 1j,k), which we denote ‘functional tissue ensembles’. These can then be annotated with tentative tissue-type identities when discernible. As an example, the WHOLISTIC workflow parcellates the pronephric kidney nephron into contiguous functional tissue ensembles, each exhibiting distinct dynamics, that together couple into travelling waves that occur in bursts (Fig. 1j,k). The spatial propagation and inter-burst quiescence suggest that kidney filtration and/or intraluminal flow—conceptualized as a continuous or continuously oscillating process^15,16^—may in fact be intermittent and pulsatile on shorter timescales, warranting follow-up experiments. Clusters across the body exhibited a range of calcium activity, showcasing diverse temporal spectra, including oscillatory, pulsatile and bistable activity (Fig. 1l,m).

Collectively, these findings demonstrate how the ubiquitous expression of molecular sensors, in conjunction with advanced motion-corrected spinning-disk confocal microscopy and the computational workflow for cellular and tissue segmentation, together comprising WHOLISTIC, facilitate the study of cellular interactions across tissues of the body.

## Optogenetic perturbation of inter-organ coupling

### Body-wide skeletal muscle coordination

**Fig. 2:**
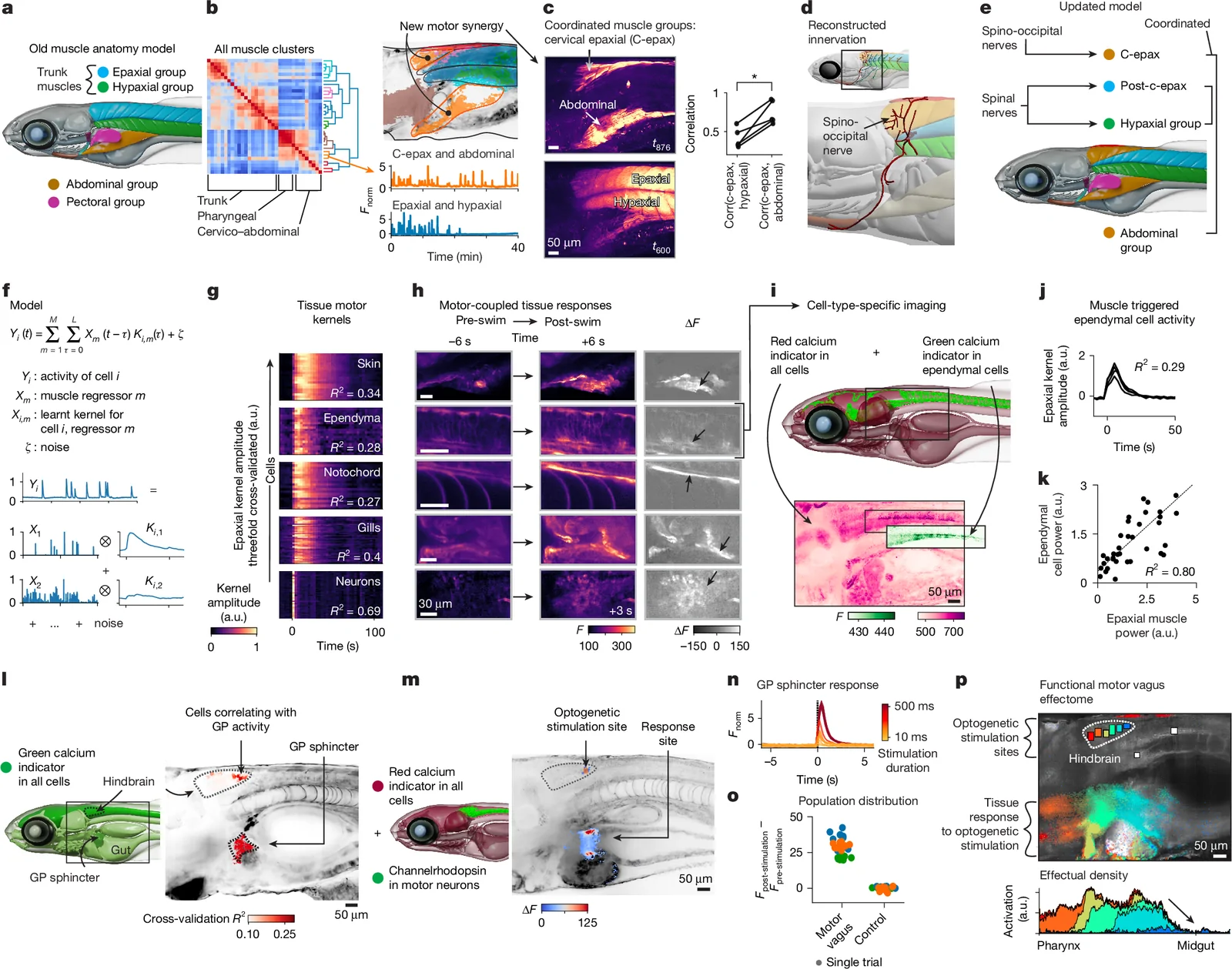
Identification and causal test of intra-organ and inter-organ coupling through WHOLISTIC. **a**, Model of zebrafish muscle anatomy. **b**, Cross-correlation matrix of muscle functional ensembles hierarchically ordered with dendrogram (left). Spatial footprints of muscle clusters (coloured as in the dendrogram) on anatomical reference (grey); the abdominal muscle and cervical epaxial (‘C-epax’) muscle are part of the same functional cluster (orange; top right). Average calcium activity traces from muscle clusters (bottom right). **c**, Time points highlighting co-activation of distinct muscle groups (maximum intensity projection (Δ*F*); left). Correlation (Corrr) between C-epax and abdominal muscles versus hypaxial muscles (right; five animals, one-sided Wilcoxon signed-rank test, *P* = 0.03125). **d**, Reconstruction of anterior motor nerve tracts: spino-occipital nerves innervating the cervical portion of the epaxial muscle and sternohyoid muscles (top), and spinal nerves innervating post-c-epaxial and hypaxial muscles (bottom). **e**, Revised muscle anatomy model. **f**, Lag-regression model of tissue motor responsiveness. **g**, Motor response kernels and threefold cross-validated variance explained (cell averaged). **h**, Example of tissue activity 6 s pre-swim and 6 s post-swim and Δ*F* (grey). From top to bottom: (1) ventral fin skin epithelium, (2) midline cells in the spinal cord (later identified as ependyma), (3) notochord sheath cells, (4) gills, and (5) neurons in the hindbrain (here 3-s post-swim activity is shown as activity is short lived). Arrows point to cells of the aforementioned tissue. **i**, Strategy to validate ependymal cell motor responsiveness (top). Sagittal section of the line expressing GCaMP7f in ependymal cells and red calcium indicator pancellularly, with the inset of the hindbrain and spinal cord (bottom). **j**, Motor response kernel of ependymal cells fit using independent channels shown in panel **i** from *Tg(foxj1a:GCaMP7f)*; *Tg(ubi:tTA;TRE:jRGECO1b)*. **k**, Scatter plot of muscle power versus ependymal cell activity power for individual motor events. The dashed line denotes the linear fit capturing 80.0% of variance. **l**, Location of the hindbrain and the gastropharyngeal (GP) sphincter (left). Maximum projection of the brain, alpha weighted by the variance explained by sphincter calcium activity (*R*^2^) in held-out data, overlaid onto anatomical reference (grey); the gastropharyngeal sphincter is in flat red (right). **m**, Strategy for optogenetic stimulation of motor vagal cells during WHOLISTIC imaging (left). Stimulus-triggered average responses overlaid with anatomical reference (number of trials = 10); stimulation site and full motor vagus outline are indicated (right). **n**, Gastropharyngeal sphincter calcium activity showing dose-dependent response to optogenetic activation of caudal motor vagal neurons. **o**, Change in fluorescence at the gastric sphincter following stimulation of caudal motor vagus or control location along the spinal cord (three fish, more than nine trials per fish). **p**, Optogenetic tiling across the motor vagus (top). Trial-triggered averages, pixels weighted and colour coded according to the most-activating region of interest inducing the most activity (10 trials per location), overlaid with anatomical reference (grey). Responsive tissue volume (activation density) along the anteroposterior axis of the gastrointestinal tract (bottom).

**Extended Data Fig. 7:**
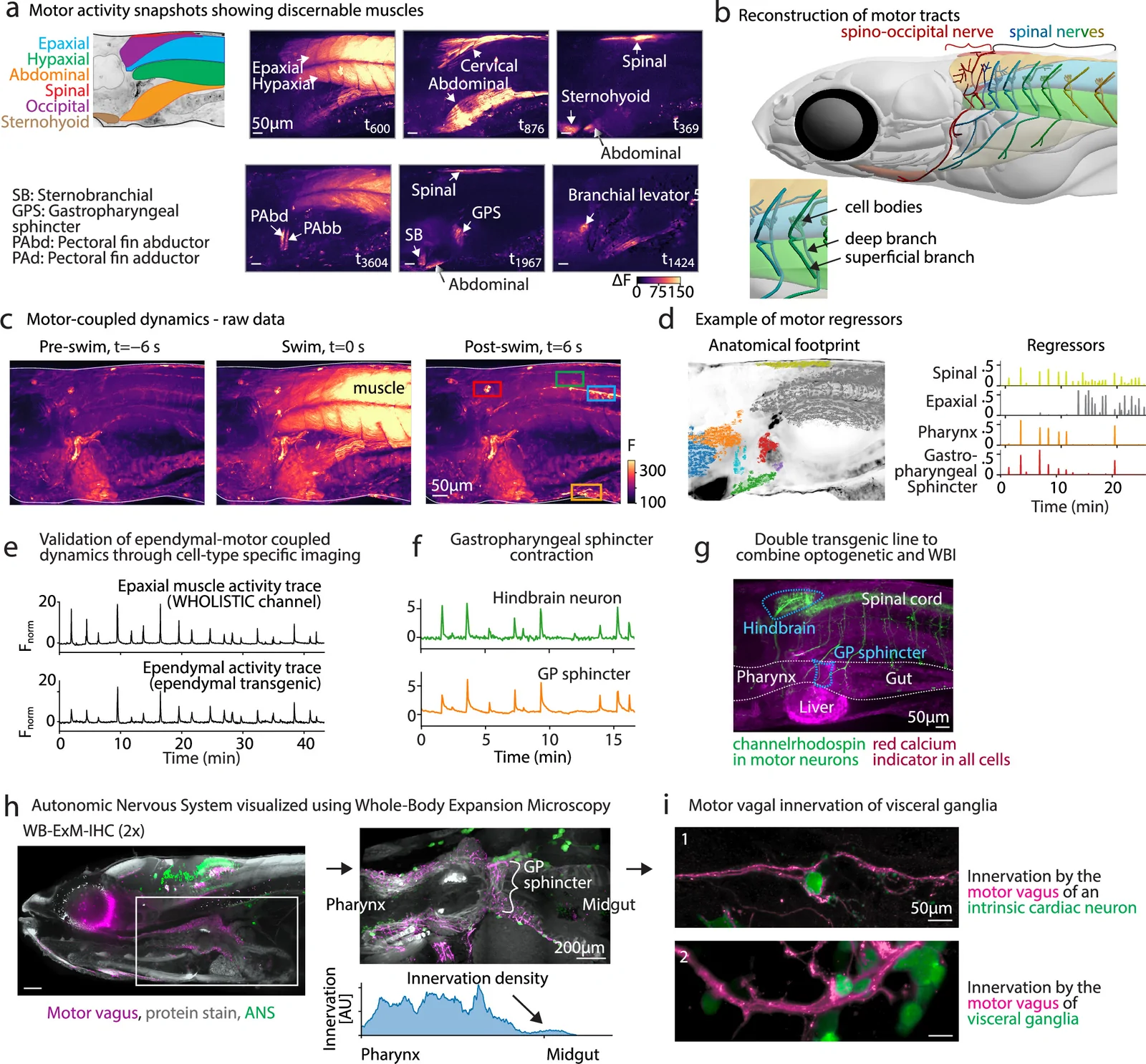
Body-wide motor coupled dynamics. **a**, Distinguishable muscle groups. Example timepoints highlighting the contraction of various muscle groups. *Left to right*: 1) Outline of muscles, 2) epaxial and hypaxial, 3) cervical portion of the epaxial muscle and abdominal muscle (same as in Fig. 2), 4) spinal muscle (not yet reported in the literature) and sternohyoid muscle, 5) fin abductor and adductor muscles, 6) gastropharyngeal sphincter muscle, sternobranchial muscle, 7) branchial levator muscle. **b**, Enlarged view of Fig. 2d showing reconstruction of spino-occipital nerves (red) innervating anterior portion of the epaxial muscle and spinal nerves innervating the remaining epaxial and hypaxial muscles. Each group of cell bodies projects both deep and superficial axonal tracts. **c**, Full field of view of data shown in Fig. 2h. **d**, Spatial footprint and average time series of muscle regressors used. **e**, Trace of average ependymal cell activity along the hindbrain and spinal cord extracted from *Tg(foxj1a:GCaMP7f)* channel and concurrent average epaxial activity extracted from *Tg(ubi:tTA;TRE:jRGECO1b)*. **f**, Trace of gastropharyngeal mean sphincter activity used with the lag-regression model and example hindbrain neuron identified as correlated. **g**, Double transgenic fish for optogenetic activation experiments. A transgenic line expressing channelrhodopsin in motor neurons is crossed to a red pancellular calcium indicator (*Tg(VAChTa:CoChR-eGFP)*; *Tg(ubi:tTA;TRE:jRGECO1b)*). Maximum projection of the green channel (green), overlaid on midline plane of red channel for anatomical reference (magenta). **h**, *Left*: sagittal section of double transgenic animal labeling the motor vagus and autonomic nervous system (*Tg(isl1CREST-hsp70l:mRFP)* x *Tg(phox2bb:eGFP)*), stained against RFP (magenta) and eGFP (green) with total protein stain (Alexa488-NHS, gray) (WB-ExM-IF, expanded ~2×). *Top right*: enlarged view of the anterior gastrointestinal tract. *Bottom right*: innervation density along the anterior-posterior axis showing dense innervation up to, but not beyond, the anterior gut. **i**, WB-ExM-IF highlights innervation of visceral post-ganglionic neurons by motor vagus. *Top*: innervation of intrinsic cardiac neurons by motor vagus. *Bottom*: innervation of enteric neuron by motor vagus.

To exemplify the use of WHOLISTIC to study coupling of calcium activity within tissue types, we began by inspecting functional tissue ensembles corresponding to skeletal muscle, in which the rise of intracellular calcium induces contraction. Muscle ensembles were classified based on their distinctive cellular morphology (Fig. 1d), stereotyped spatial location (Fig. 2a) and characteristic activity waveform. Next, the ensembles were organized through hierarchical clustering of their calcium activity patterns and visualized by projecting their spatial footprint back into anatomical space (Methods and Fig. 2b). This approach readily enabled the identification of the primary epaxial and hypaxial trunk muscles, which exhibited correlated activity patterns (as represented by the union of the blue and green clusters in Fig. 2b), underscoring their propensity to contract synchronously, as documented in other species^17^. This method also identified the abdominal (also known as hypaxialis), sternohyoid, pectoral fin and branchial muscles^18^ (Extended Data Fig. 7a). More surprisingly, the anterodorsal portion of the epaxial muscle grouped in a distinct functional tissue ensemble (‘cervical epaxial muscle’), with calcium activity correlated with the abdominal muscle (Fig. 2c, right), a finding that, to our knowledge, has not been previously reported. These results can be visualized within the raw data by examining time points at which specific muscle groups are selectively activated (Fig. 2c, left).

To ascertain the neural underpinning of the independence of activity of the cervical epaxial muscle from the primary epaxial muscle, we traced the anterior motor nerve tracts using a transgenic line labelling motor neurons, *Tg(VAChTa:eGFP)* (Methods). This revealed that, although the spinal nerves predominantly innervate the majority of the epaxial and hypaxial musculature, the spino-occipital nerve—known to innervate the ventral sternohyoid and pectoral fin muscles^19^—extends axons dorsally to innervate the cervical portion of the epaxial muscle (Fig. 2d and Extended Data Fig. 7b). This dual innervation supports the independent regional activation, consistent with previous studies in other species demonstrating regional activation^20^. Together, this shows how WHOLISTIC can uncover, even within extensively studied tissue dynamics—muscles—new functional units, as well as reveal previously unidentified synergies and independencies among muscle groups (Fig. 2e).

### Muscle-coupled dynamics

To demonstrate the ability of WHOLISTIC to identify inter-organ interactions, we next studied the coupling between skeletal muscle and other bodily tissues. Beyond moving the animal, skeletal muscles generate internal mechanical forces detected by mechanosensitive cells body wide^21^. Connections through the nervous, vascular and other systems can additionally induce coupling between muscles and other organs. Yet, the acute effects of muscle activity on non-muscle tissue types and vice versa remain largely uncharted.

We thus asked how motor activity acutely affects organ and tissue dynamics throughout the body. We examined the temporal relationships between the activation of skeletal muscles and calcium signalling in other cells of the body (Fig. 2f–h) by fitting a lag-regression model to all cells within the imaged volume, in which cellular calcium activity is modelled as the convolution of muscle activity and a cell-specific motor-response kernel (Methods, Fig. 2f and Extended Data Fig. 7c,d; we note that this model does not purport to infer causal direction). We quantified motor-related cellular activity throughout the imaged volume using statistical parametric mapping, which highlighted responses in various tissues, including the brain, skin epithelium, notochord sheath cells and gills (Fig. 2g,h). This coupling exhibited distinct, tissue-specific responses, often enduring beyond the timescale of muscle activity, except in the brain, where a cluster of brainstem neurons showed short coupling timescales (Fig. 2g,h, row 4).

Within the brain, beyond motor-coupled neurons, a cohort of cells in the midline of the hindbrain and spinal cord exhibited temporally extended responses to muscular activity, enduring significantly longer than the neuronal responses (approximately 15 s; Fig. 2g,h, row 2). On the basis of their anatomical position and morphology, we hypothesized that these were ependymal cells, glia-like cells that line the ventricles and the central canal^22^. To test this hypothesis, we generated a transgenic line expressing GCaMP7f specifically in ependymal cells (*Tg(foxj1a:GCaMP7f)*), as well as a new pancellular transgenic line expressing a red fluorescent calcium sensor^23^, *Tg(ubi:tTA;TRE:jRGECO1b)*. Crossing these two lines enabled the simultaneous recording of definitive ependymal cell calcium activity, as well as comprehensive whole-body dynamics in independent channels (Fig. 2i) and confirmed that ependymal cells exhibit Ca^2+^ transients subsequent to motor activity, with response amplitudes that are proportional to the intensity of motor events (Fig. 2j,k and Extended Data Fig. 7e). This suggests that ependymal cells integrate body movements into their cellular state, potentially contributing to ventricular and cerebrospinal fluid homeostasis.

Revealing this coupling between ependymal calcium and muscle activity illustrates the sensitivity of WHOLISTIC for discovering functional properties even amid sparse and distributed cell populations.

### Optogenetic test of brain–body coupling

To expand the analysis to include visceral organs as regressors, we considered relationships between cellular and smooth muscle activity, which exist in tissues forming the gut, the lining of blood vessels, the gallbladder and others, by incorporating smooth muscle ensembles into the lag-regression model. Among the various relationships identified, the model captured a relationship between the caudal hindbrain and smooth muscle of the gastropharyngeal sphincter. Indeed, this regressor accounted for the largest fraction of variance of a small group of cells within the caudal hindbrain, specifically within the region of the motor vagal nuclei (Fig. 2l and Extended Data Fig. 7f). As the model is agnostic to causal direction, this is consistent with these motor vagal neurons driving the sphincter calcium activation via the vagus nerve. Given the established connection between the motor vagus and the gut across species^24^, this finding represents the identification of a conserved vagus–gut circuit from an unbiased WHOLISTIC analysis.

We sought to probe whether the relationship between this motor vagal area and the gastropharyngeal sphincter was causal. To do this, we combined WHOLISTIC with optogenetics by crossing a transgenic line expressing channelrhodopsin in the motor vagus with the pancellular genetically encoded calcium indicator line (*Tg(VAChTa:gal4;UAS:CoChR-eGFP)*; *Tg(ubi:tTA;TRE:jRGECO1b)*; Extended Data Fig. 7g). We found that activation of neurons within the most caudal segment of the motor vagus induces a dose-dependent calcium activation across the gastric sphincter (Fig. 2m–o), recapitulating the observed coupling identified by the model. Moreover, examination of the entire motor vagus through the successive activation of subsets of neighbouring vagal neurons revealed a topological mapping between the motor vagus and visceral tissues along the anteroposterior axis (Fig. 2p), with more anterior motor vagal neurons controlling more anterior pharyngeal muscles^25^. We noted that neural activity exerted control of smooth muscle up to the anterior gut but not beyond. To assess whether there was an anatomical underpinning for this spatially restricted control, we visualized the distribution of motor vagal axons along the gastrointestinal tract using WB-ExM (Methods and Extended Data Fig. 7h,i), revealing a significant drop in innervation density at the level of the anterior gut, paralleling the functional results and offering a potential anatomical basis for the functional findings. This combination of WHOLISTIC, optogenetics and ExM-based anatomical mapping underscores its capacity to rapidly uncover and mechanistically examine brain–body circuits.

## Ultraslow oscillations during motor quiescence

As a demonstration of the broad applicability of WHOLISTIC, we next considered the converse of movement-coupled modulation of tissue activity: processes that occur during extended periods of motor quiescence. In between phases of sustained motor activity, animals engage in equally important periods of muscle inactivity associated with rest, sleep and tonic immobility. These quiescent states are active phases of physiological regulation, yet although the neural substrate during them is extensively studied, the contributions of other cell types remain less characterized.

**Fig. 3:**
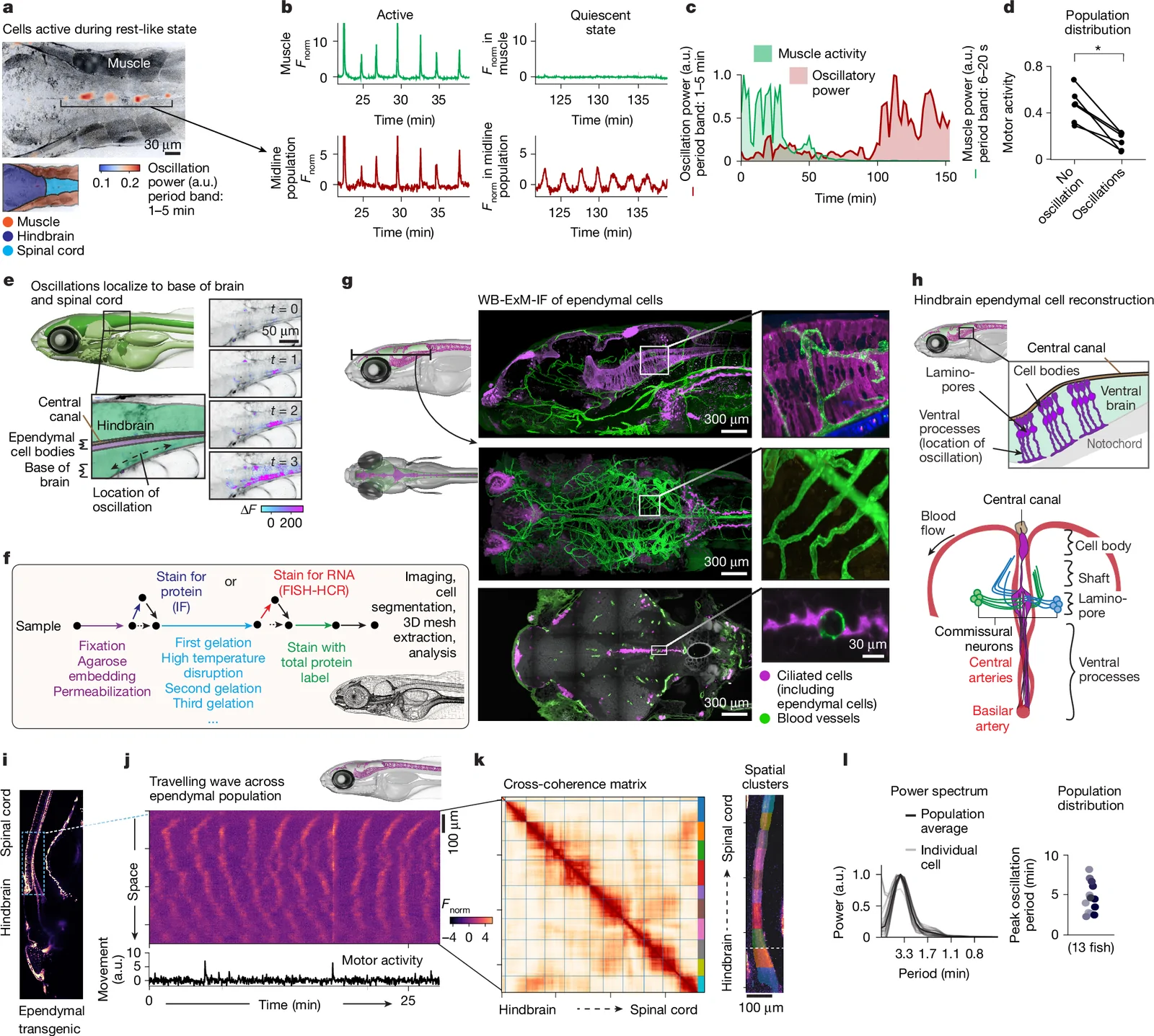
Discovery of motor-quiescence-coupled ultraslow oscillations and cell type of origin. **a**, Midline cells in the hindbrain and spinal cord display high-amplitude ultraslow oscillations during periods of extended motor quiescence. Heatmap of oscillatory power (periodicity band: 1–5 min), overlaid on anatomical reference (grey). **b**, Traces during the two activity states: active period, coupled muscle and ependymal cell activity (left), and motor-quiescent period, ependymal oscillatory dynamics (right). **c**, Spectral power of oscillatory cells and muscle activity over a 3-h window showing anticorrelated dynamics (periodicity band: 1–5 min and 6–20 s for the midline population and muscles, respectively). **d**, Quantification of motor activity during oscillatory and non-oscillatory periods, pooled from fish showing both motor states (six fish, one-sided Wilcoxon signed-rank test, *P* = 0.016). **e**, Propagation of the Ca^2+^ wave along the base of the hindbrain. Sequential time points and Δ*F* overlaid onto anatomical reference (grey) are shown. **f**, Outline of the WB-ExM protocol. IF, immunofluorescence; HCR, hybridization chain reaction. **g**, Ependymal cell location. The sagittal and dorsal sections of the double transgenic sample labelling the ventricular and vascular systems (*Tg(foxj1a:eGFP)* × *Tg(flk1:dsRED-CAAX)*), stained against eGFP (magenta) and dsRED (green), with total protein stain, Alexa488-NHS (grey; WB-ExM-IF, 10 days post-fertilization, expanded approximately 2×). **h**, Schematic of individual hindbrain ependymal cells. **i**, Genetic strategy for imaging ependymal cells through the use of the *foxj1a* promoter. Sagittal section of the *foxj1a*-transgenic line (maximum intensity projection). **j**, Kymograph of ependymal cell activity along the hindbrain and spinal cord showing local travelling waves and occasional coupling to sparse motor activity over the course of approximately 30 min (top). Data were derived from a cell-type-specific transgenic line (*Tg(foxj1a:GCaMP7f)*). A normalized motor activity trace showing two motor events during the recording (bottom). **k**, Cross-coherence matrix derived from the kymograph data in panel **j**, showing a block-diagonal structure indicative of local coherence clusters (left). Coherence-based *k*-means clusters projected onto the anatomical space, showing spatial continuity (right). **l**, Periodogram of ependymal cell activity (left), with the population mean (dark) and individual cells (light) from an example specimen shown. Population distribution of peak oscillatory frequency is also shown (right).

**Extended Data Fig. 8:**
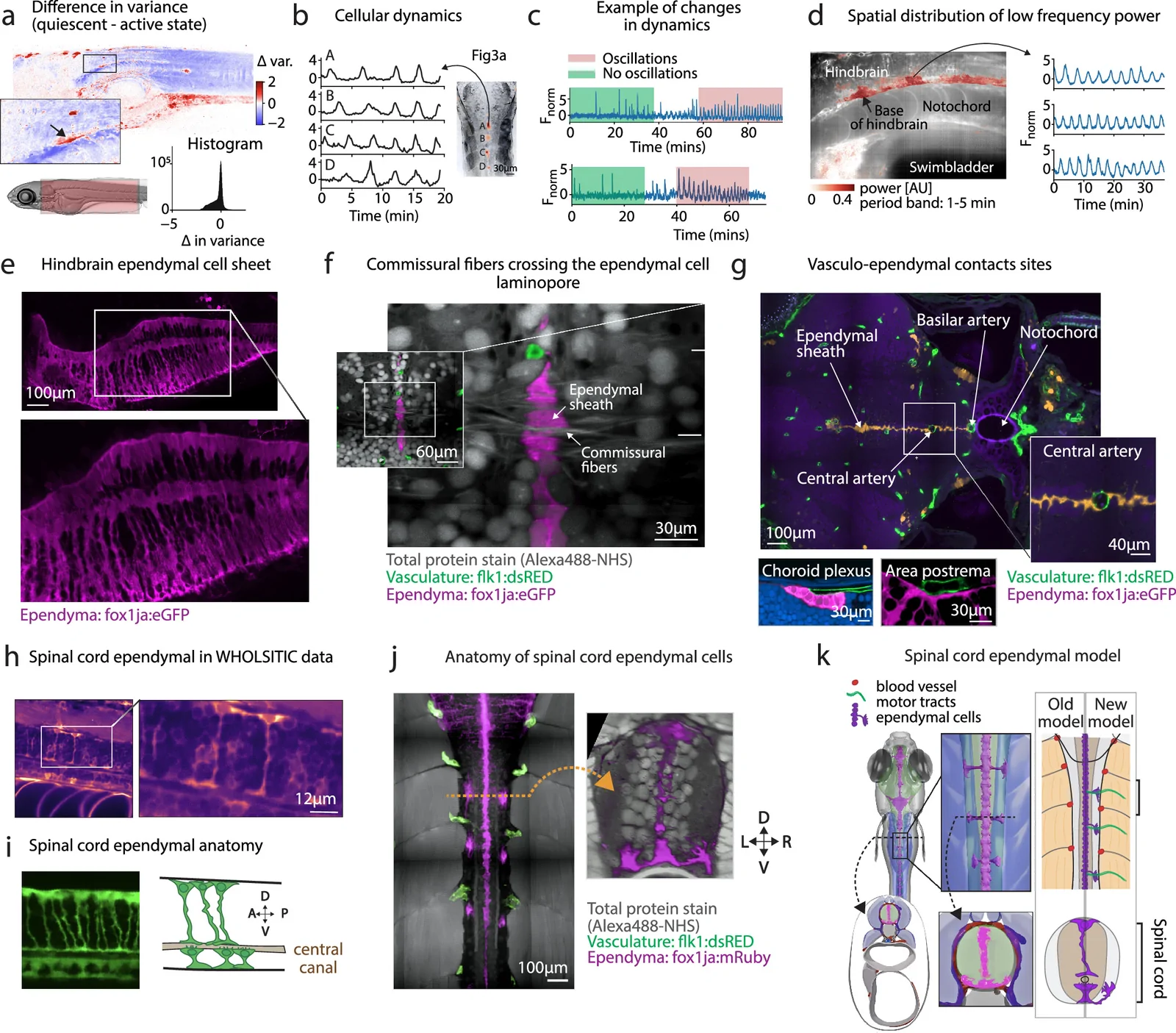
Motor-quiescence coupled ultraslow oscillations and cell-type of origin. **a**, Ultra-slow oscillations during periods of prolonged quiescence. *Top*: maximum projection of difference in activity variance across the body between periods of quiescence and active state. Enlarged view of ventral hindbrain. *Bottom*: histogram of change in variance of cells. **b**, Example of ultra-slow dynamics at different regions along the hindbrain and spinal cord. Regions show similar frequency, however, appear phase shifted. (Same animal as in Fig. 3a). **c**, Example of changes in cellular dynamics observed with cells initially without oscillations (green phase) gradually displaying slow oscillations (magenta phase). **d**, Spatial distribution of low frequency power during periods of quiescence mostly localizes to the base of the hindbrain and spinal cord. **e**, *Top*: hindbrain ependymal cell midline sheet viewed sagittally. *Bottom*: enlarged view annotated with location of cell body layer, shaft, laminopore and ventral processes layer. Sagittal section of transgenic sample labeling the ventricular system (*Tg(foxj1a:eGFP), magenta*, labelled and expanded using WB-ExM-IF). **f**, Visualization of commissural fibers crossing the midline at the level of the laminopore. Total protein stain (Alexa488-NHS, gray), vasculature (*Tg(flk1:dsRED-CAAX)*, green), ependyma (*Tg(foxj1a:eGFP)*, magenta). **g**, Visualization of vasculo-ependymal cell contact sites with annotations. Same sample as in Fig. 3g. Total protein stain (Alexa488-NHS, dark blue), vasculature (*Tg(flk1:dsRED-CAAX)*, green), ependyma (*Tg(foxj1a:eGFP)*, gold top, magenta bottom). **h**, Appearance of spinal cord ependymal cells in WHOLISTIC data. Sagittal view of the spinal cord of *Tg(ubi:tTA;TRE:GCaMP7f)* line. **i**, Appearance of spinal cord ependymal cells in transgenic line labeling motile ciliated cells. *Left*: sagittal view of the spinal cord of *Tg(foxj1a:eGFP)* line imaged using spinning disk confocal microscopy. *Right*: graphical representation of the dorsal and ventral ependymal cell population. **j**, Visualization of spinal cord ependymal cells in WB-ExM-IF data. Same sample as **f**. *Left*: dorsal view. *Right*: coronal view highlighting bilateral projections in between somites, location of spinal nerve exit sites. Total protein stain (Alexa488-NHS, gray), vasculature (*Tg(flk1:dsRED-CAAX)*, green), ependyma (*Tg(foxj1a:eGFP)*, magenta). **k**, Graphical model of spinal cord ependymal cells.

We therefore aimed to identify cells—whether neuronal or non-neuronal, within or outside the brain—that exhibited heightened activity during motor-quiescent periods. We analysed the variance in cellular calcium activity during episodes of extended muscle inactivity (more than 10 min) compared with periods of regular motor activity. This analysis highlighted a region in the central nervous system that exhibited increased activity during rest (Extended Data Fig. 8a). These signals were identifiable along the midline of the hindbrain and spinal cord, displaying high-amplitude, ultraslow oscillatory activity during motor-quiescent phases, with a periodicity of 3–7 min (Fig. 3a and Extended Data Fig. 8b,c). By contrast, during periods of motor activity, faster and abrupt muscle-locked activity was observed in these cells, and the slow oscillations were either absent or significantly attenuated (Fig. 3b–d). Imaging neuronal and astrocyte-specific lines (*Tg(elavl3:H2B-GCaMP7f)* and *Tg(gfap:jRGECO1b)*) failed to recover such dynamics, leading to the hypothesis that these slow signals may originate from ependymal cells. Ependymal cells have been associated with sleep and alterations in cerebrospinal fluid flow^26,27^ and are juxtaventricular cells, with their cell bodies residing along the central canal and ventricles^22^. However, the oscillations were primarily observed at the base of the hindbrain (Fig. 3e), above the notochord—an area far from the ventricles or central canal, where ependymal cells are typically assumed to reside (Extended Data Fig. 8d)—bringing into question whether the oscillations originate from ependymal cells. To resolve this discrepancy, we sought to visualize the detailed morphology of ependymal cells that reside deep within the brain.

### Whole-body expansion microscopy

**Extended Data Fig. 9:**
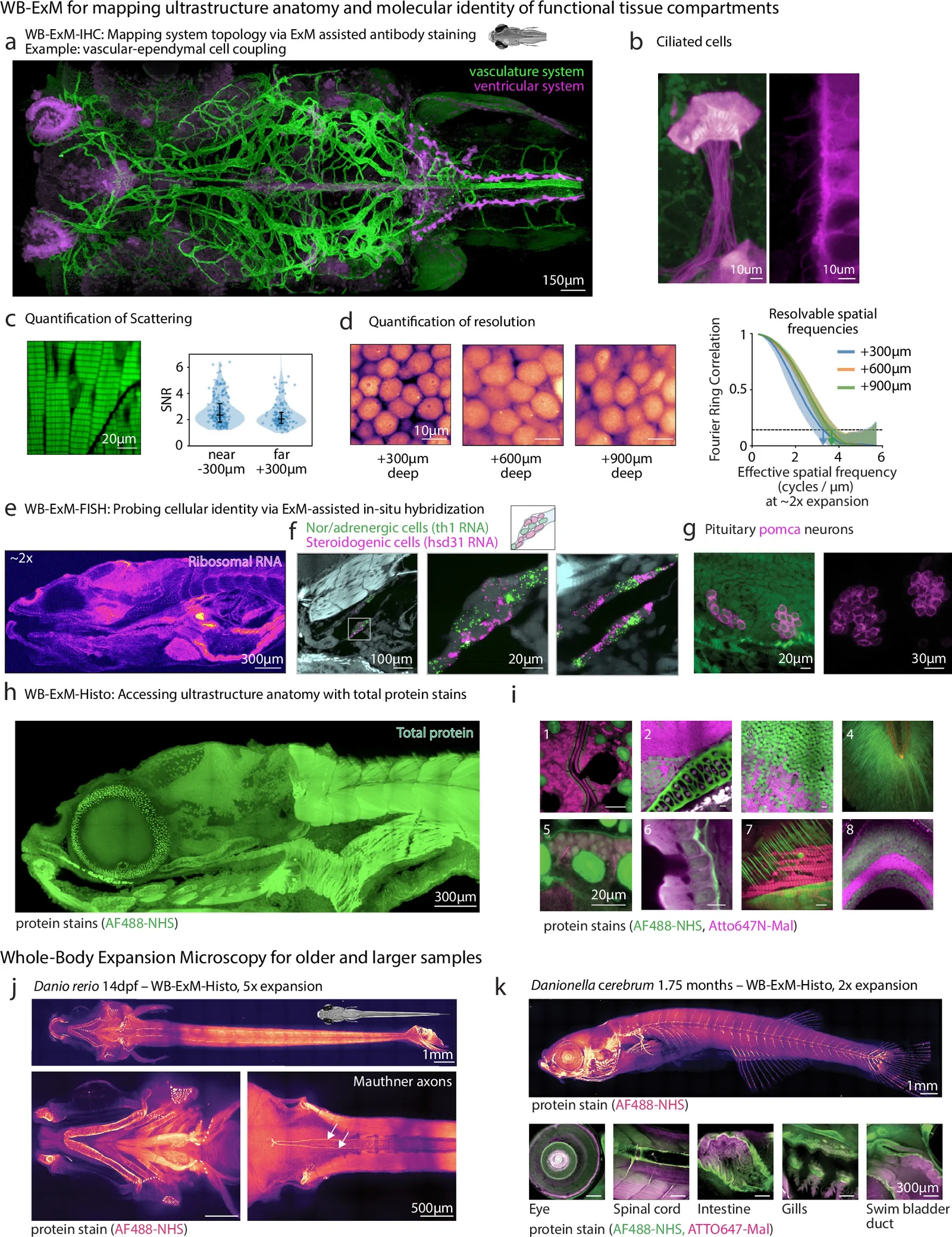
Whole-Body Expansion Microscopy (WB-ExM) to map ultrastructure, RNA and protein across the body. **a**, Enlarged view of Fig. 3g. Dorsal section of double transgenic sample labeling the ventricular and vascular systems (*Tg(foxj1a:eGFP)* x *Tg(flk1:dsRED-CAAX)*), stained against eGFP (magenta) and dsRED (green) (10 days post-fertilization, expanded ~2×). **b**, Enlarged view of a cell from sample **a** in the nephron epithelium projecting cilia into the nephric lumen. **c**, Quantification of clearing quality of WB-ExM sample. *Left*: Image of muscle sarcomeres in WB-ExM sample stained with total protein stain (Alexa488-NHS). *Right*: Distribution of signal to noise ratio (peak to trough) in sarcomeres at far and near side of the sample (Violins show the distribution; black bars mark the median and IQR. N=152, 208 sarcomeres, from one sample). **d**, Quantification of present and resolvable spatial frequencies in WB-ExM sample. *Left*: Image of neurons located at different depths within the brain, stained with total protein stain (Alexa488-NHS). *Right*: Fourier Ring Correlation of WB-ExM data at different depths showing little attenuation in resolvable frequencies. The spatial frequency at which the correlation falls below 1/7 is typically taken as the resolution cut-off. Data presented as mean +/− SD. **e**, Whole-Body Expansion Microscopy combined with fluorescent in situ hybridization (WB-ExM-FISH) enables RNA-based cell identification across tissue. Sagittal section of an expanded sample stained against ribosomal RNA, demonstrating full probe access to all tissues (8 days post-fertilization, ~2× expanded). **f**, Localization of nor/adrenergic cells and steroidogenic cells using WB-ExM-FISH. *Left*: sagittal section of an expanded sample (2×) stained against *th* (green) and *hsd31b* (magenta) RNA and with total protein stain (Alexa488-NHS, cyan). *Right*: enlarged view showing intermingled cellular populations. **g**, Localization of pituitary pomc neurons using WB-ExM-FISH, cells situated at the base of the brain that are typically hard to optically access. *Left*: coronal view of expanded sample (2×) stained against *pomca* (magenta) RNA and with total protein stain (Alexa488-NHS, green), showing two distinct populations. *Right*: rendering of volume. **h**, Whole-Body Expansion Microscopy combined with total protein stains (WB-ExM-Histo) to provide contextual information. **i**, Higher magnification views of expanded sample: 1) enteric goblet cell, 2) osteochondral tissue at the base of the brain, 3) midbrain neurons, 4) tail fin, 5) epithelial cell of nephron with primary cilia visible, 6) intestinal epithelium with basement membrane, 7) collagen fibers, 8) multilayered retina. Total protein stains: Alexa488-NHS (green) and Atto647N-Maleimide (magenta). **j**, Whole-Body ExM for older samples. WB-ExM-Histo of 14 days post fertilization zebrafish after 4 gelation rounds leading to ~5× expansion, stained with total protein stain Alexa488-NHS. **k**, Whole-Body ExM for *Danionella cerebrum*. WB-ExM-Histo of 1.75 months *Danionella cerebrum* stained with total protein stain Alexa488-NHS (green) and Atto647N-Maleimide (magenta). *Top*: maximum intensity projection of entire sample. *Bottom, left to right*: enlarged views of same sample showing 1) eye, 2) spinal cord, 3) intestine, 4) gills, 5) swim bladder duct.

**Extended Data Fig. 10:**
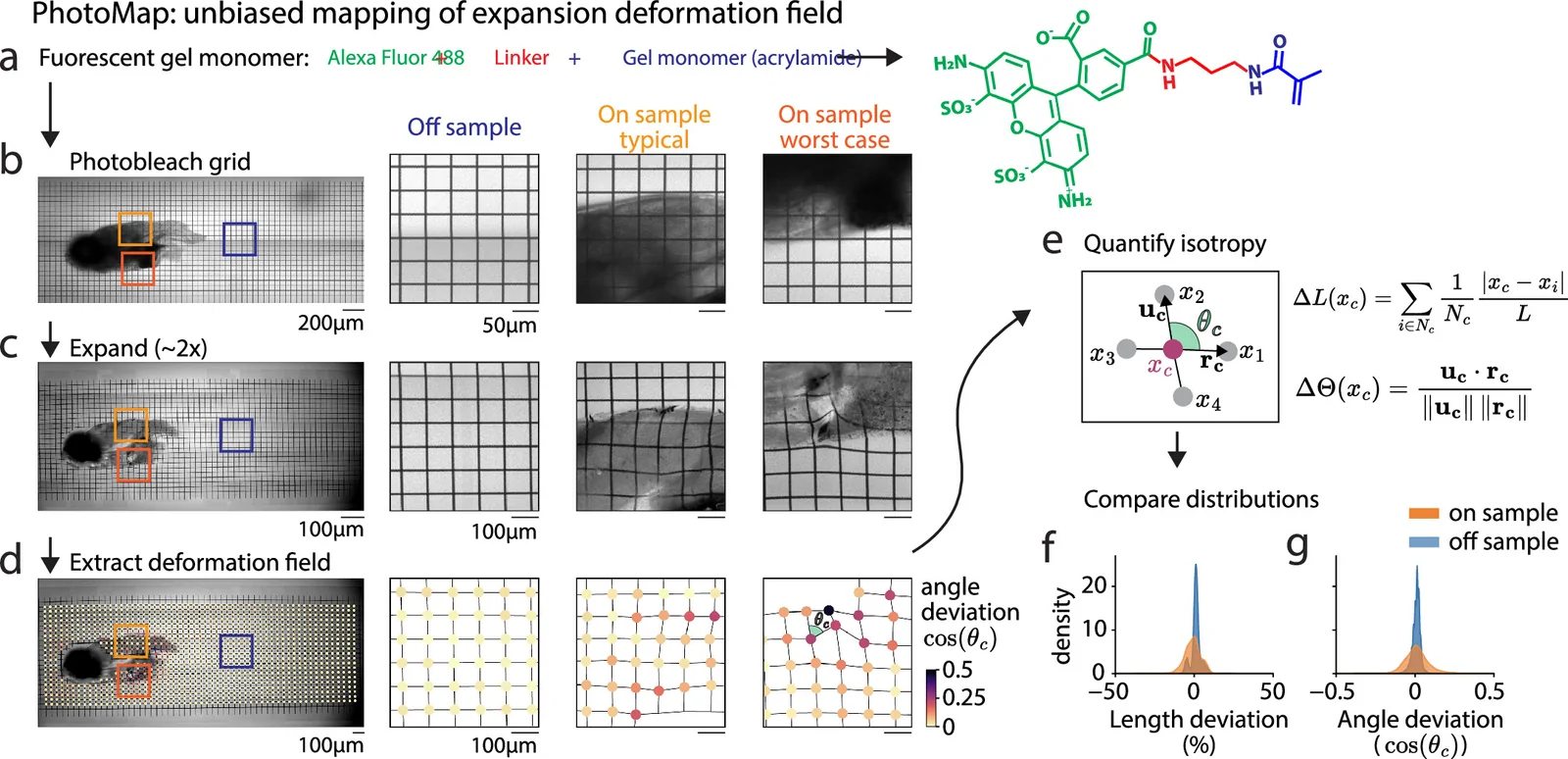
PhotoMap: Unbiased mapping of expansion deformation field. **a**, Schematic of fluorescent monomer. A fluorescent dye (Alexa Fluor 488) is covalently conjugated to the gel monomer via a link, rendering the gel intrinsically fluorescent. **b**, Grid photobleaching. Using a two-photon microscope, a three-dimensional grid (50 × 50 × 50 μm) was photobleached into both the sample and the surrounding gel. *Left*: whole-gel image with the grid visible. *Right*: example crops showing regions containing no sample, brain tissue, and the cleithrum, the earliest mineralizing bone in zebrafish. **c**, Visualization of deformation after expansion. Same views as in **b** imaged after tissue disruption and gel expansion. **d**, Extraction of the deformation field. Grid intersections were algorithmically detected throughout the sample. Same views as in **b**, with intersection points color-coded by their deviation from the ideal 90° intersection angle. **e**, Quantification of isotropy. For each grid intersection, the mean deviation from the target length *L* to its four nearest neighbors was measured, along with the deviation from the ideal 90° intersection angle. **f**,**g**, Deformation field comparison. Distributions of length deviation (**f**) and angle deviation (**g**) measured in off-sample (blue) and on-sample (orange) regions. On-sample distributions are broader but remain within a relatively narrow range.

Despite the relative transparency of the zebrafish, the accumulation of refractive index variations across the body results in light scattering, making it difficult to visualize the fine anatomy of such midline structures. Existing clearing and expansion methods are either only applicable to younger samples (5 days or less post-fertilization), achieve clearing at the expense of extensive proteolytic digestion^28,29^ or require extensive wash steps to clear adequately without expansion^30^. To overcome these limitations, we developed an advanced enzyme-free, rapid and robust WB-ExM protocol^2^ (Fig. 3f) that enables high-quality clearing and up to 5× uniform expansion, along with the acquisition of molecular information at subcellular resolution (Extended Data Fig. 9a–i) throughout entire larval and juvenile zebrafish (Extended Data Fig. 9j), as well as mature *Danionella cerebrum* (Extended Data Fig. 9k). This protocol combines high-temperature disruption with a multi-round embedding strategy, gradually reinforcing and expanding the sample to ensure homogeneous expansion (Methods). WB-ExM retains immunofluorescence signals (WB-ExM-IF; Methods, Extended Data Fig. 9a,b and Supplementary Video 6) and, with modifications, is compatible with in situ hybridization (WB-ExM-FISH; Methods; Extended Data Fig. 9e–g). In addition, the use of total protein stains yields rich anatomical information (WB-ExM-Histo) with which to contextualize the immunofluorescence and FISH signals^31^ (Extended Data Fig. 9h and Supplementary Video 7). To quantitatively assess expansion quality across the sample, we developed PhotoMap, an unbiased method for extracting the deformation field of the gel (Extended Data Fig. 10), inspired by GelMap^32^. Expansion was found to be largely uniform (Extended Data Fig. 10f,g), except in regions of mineralizing bone, where tissue pinching was observed but remained spatially confined (Extended Data Fig. 10c,d). These tools provide a high-throughput route to comprehensive whole-body anatomical and molecular data, complementing histological, X-ray and electron microscopy approaches, and a vital basis for interpreting and validating WHOLISTIC findings.

We used WB-ExM-IF to characterize the fine morphological features of ependymal cells (Fig. 3g and Extended Data Fig. 8e–g) and found that this population of hindbrain ependymal cells, typically considered to be small cuboidal cells^33^, actually extends long and dense ventral projections (approximately 120 μm), forming a sheet-like structure that runs along the entire midline of the hindbrain, reaching the floor of the brain (Fig. 3g,h and Supplementary Video 8), thereby explaining the spatial distribution of the oscillatory signal. The morphology of these ependymal cells echoes a class of ependymal cells, including tanycytes, that retain morphological features of their progenitor, radial glia cells^22^, and are widely present in the adult mammalian brain, suggesting an evolutionarily conserved lineage^34^. These cells also displayed previously undescribed morphological features (Extended Data Fig. 8f–k): processes ensheathing commissural fibre crossings approximately 25 μm below the central canal, ensheathment of passing arteries and evenly spaced lateral projections surrounding neuronal tracts exiting the spinal cord echoing those recently identified in mice^26,35^. Such findings, not readily observable in non-expanded samples, show how WB-ExM resolves the cellular and extracellular provenance of functional signals, supporting hypothesis generation.

Cell-type-specific imaging of ependymal cells recovered the motor quiescent-locked ultraslow oscillatory dynamics, confirming the ependymal cell origin of the ultraslow oscillations (Fig. 3i–l). To understand the spatiotemporal structure of ependymal activity, we performed cross-coherence analysis to assess the presence of coupling between the oscillatory dynamics of cells (Methods). This uncovered a spatial organization manifesting as local waves along the anteroposterior axis of the fish, encompassing both the hindbrain and the spinal cord (Fig. 3j), forming a block-like structure in the cross-coherence matrix with a length scale of approximately 75 μm. To quantitatively evaluate this, we derived a method for utilizing coherence as a metric in *k*-means clustering (Methods); the algorithm, blind to the anatomical location of the cells, recovered spatially continuous clusters (Fig. 3k). Ependymal cells are interconnected via gap junctions^36^, probably accounting for the localized propagation of the signal. Calcium within ependymal cells has been associated with F-actin-mediated cellular shrinkage, resulting in increased paracellular space between ependymal cells, thereby facilitating efflux through perineuronal routes^26^. These findings support the hypothesis that these cells form an interconnected network across the brain and spinal cord, capable of integrating and modulating neural activity, vascular signals, and cerebrospinal fluid composition. Moreover, it suggests that the observed state-dependent calcium oscillatory modes of ependymal cell activity may correspond to prolonged periods of altered cerebrospinal fluid exchange during extended motor quiescence.

Together, these analyses show that WHOLISTIC can identify state-dependent sparse cellular signals within densely labelled tissue that can subsequently be validated and followed up using cell-type-specific transgenics and WB-ExM, and highlights previously overlooked populations active during specific behavioural states.

## Brain–body responses to hypoxia

To demonstrate the power of WHOLISTIC for studying organism-wide responses to physiological stress, in addition to spontaneous activity (Figs. 2 and 3), we examined how animals adapt to hypoxia: a condition of reduced oxygen availability that engages systemic responses across tissues and organs. Hypoxia is a fundamental physiological challenge that arises in contexts ranging from high-altitude adaptation to stroke and heart failure, and triggers genetic, metabolic, cellular and behavioural adaptations that maintain oxygen homeostasis and prioritize energy allocation to critical tissues^37^. However, the dynamic interplay between single-cell activity changes and whole-organism physiology during hypoxia remains poorly understood, largely due to the technical challenges of simultaneously monitoring multiple tissues in real time.

**Fig. 4:**
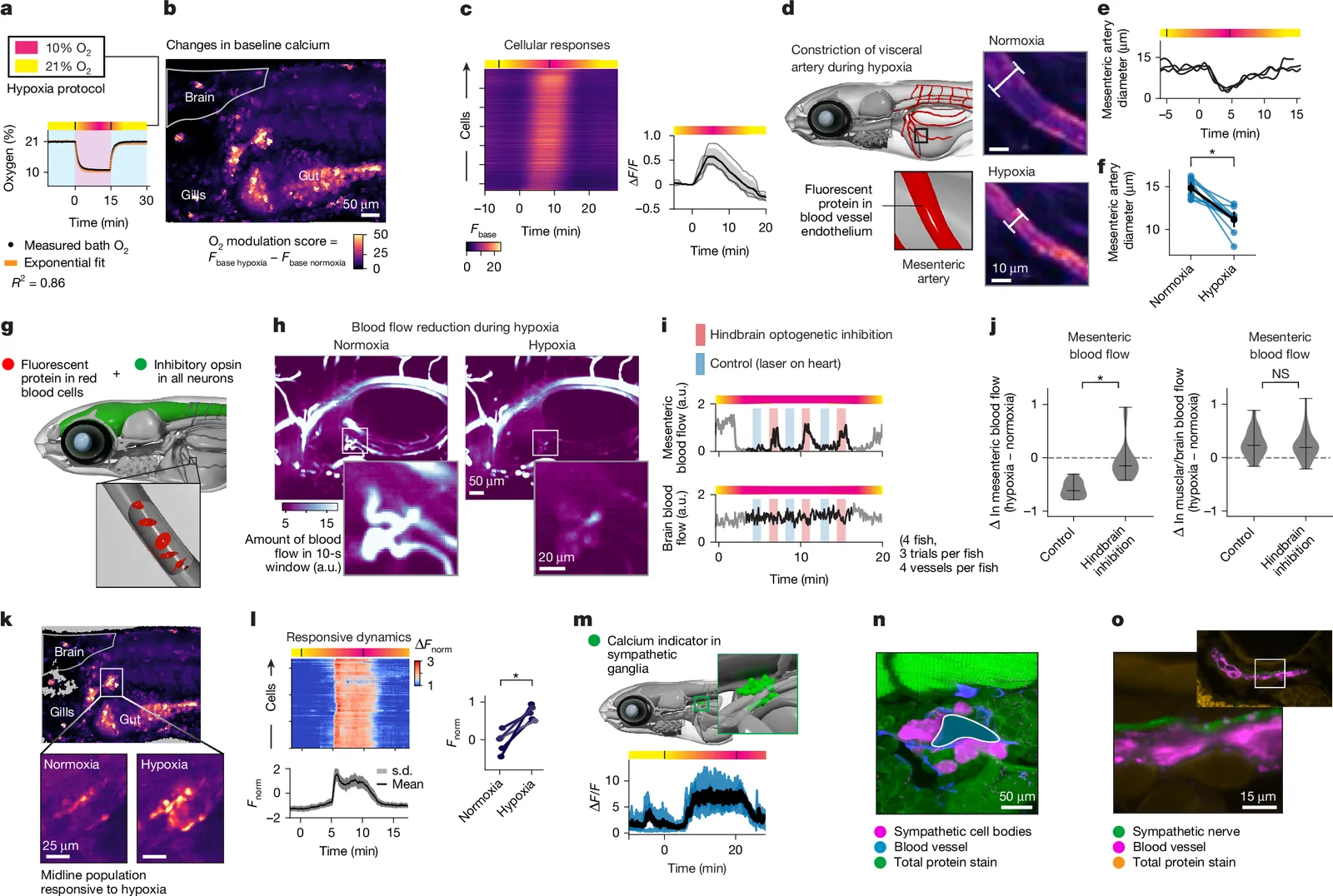
Uncovering of a body-wide circuit engaged in response to stress. **a**, Hypoxia paradigm. Cyclic normoxia (21% O_2_) and hypoxia (10% O_2_) periods. The black line denotes measured oxygen levels within the water-submerged agarose, approximating exponential kinetics (*τ* ≈ 1.2 min). **b**, Whole-body map of cellular oxygen modulation score (maximum intensity projection). The gut, liver and kidney show increased baseline Ca^2+^ levels during hypoxia. **c**, Raster of baseline Ca^2+^ levels across visceral tissues, ordered by peak time (left). Average hepatic baseline calcium levels in response to hypoxia (right; dark denotes the mean response, and light indicates individual animals; *n* = 4 fish). **d**, Strategy to image and estimate blood vessel diameter (*Tg(flk1:dsRED-CAAX)*). The mesenteric artery during normoxia (top right) and hypoxia (bottom right) shows a narrower diameter (maximum intensity projection). **e**, Diameter of the mesenteric artery during the experiment (three trials for the same animal). **f**, Mesenteric artery diameter during normoxia and hypoxia (three animals, three or more trials per animal, one-sided Wilcoxon signed-rank test, *P* = 0.00097). **g**, Strategy for optogenetic silencing of the brain while imaging blood flow; a line expressing an inhibitory opsin in all neurons (*Tg(elavl3:gtACR2-eYFP)*) is crossed to a line labelling red blood cells (*Tg(gata1:dsRED)*). **h**, Mesenteric blood flow decreases during hypoxia. A sagittal section of an animal, averaged during 10 s, during normoxia (left) and hypoxia (right). **i**, Optogenetic hindbrain inhibition restores blood flow to the gut. Blood flow in mesenteric (top) and cerebral (bottom) arteries in response to hypoxia and to optogenetic neural inhibition is shown. Optogenetic inactivation of the hindbrain is in red, and control light stimulus on the heart is in blue. **j**, Blood flow to the mesenteric artery and to the brain under normoxic and hypoxic conditions (four fish, three trials per fish, four vessels per fish, independent two-sided Student’s *t*-test, hindbrain inhibition: *P* = 2.0 × 10^−9^, control: *P* = 0.56; horizontal dashed line marks zero for visual reference; horizontal lines mark median and data extrema). **k**, Midline compact cell population shows acute and sustained response to hypoxia, probably being the sympathetic ganglion. Same as panel **b** with the location of the population shown (top). Enlarged views of the population during normoxia (bottom left) and hypoxia (bottom right) are also shown. **l**, Raster of a functional tissue cluster that maps to midline population (top left), and mean and s.d. of the raster (bottom left). Average population calcium levels during hypoxia versus normoxia (right) are also shown (five animals, one-sided Wilcoxon signed-rank test, *P* = 0.03125). **m**, Strategy to specifically image the sympathetic ganglia (*Tg(th:gal4;UAS:GCaMP6f)*). Response dynamics of the sympathetic ganglion to hypoxia. Individual cells (blue) and population average (black) are shown. **n**, Localization of the sympathetic ganglion. A sagittal section of the double transgenic sample labelling the autonomic nervous system and vascular systems (*Tg(phox2bb:eGFP)* × *Tg(flk1:dsRED-CAAX)*), stained against eGFP (magenta) and dsRED (blue), with total protein stain, Alexa488-NHS (green; WB-ExM-IF, 10 days post-fertilization, expanded approximately 2×); sympathetic neurons line the cardinal vein. **o**, Juxtaposition of sympathetic fibres (green) along the mesenteric artery (magenta). Same sample as in panel **n**. The vascular system (magenta), autonomic nervous system (green) and total protein stain (dark gold) are shown.

**Extended Data Fig. 11:**
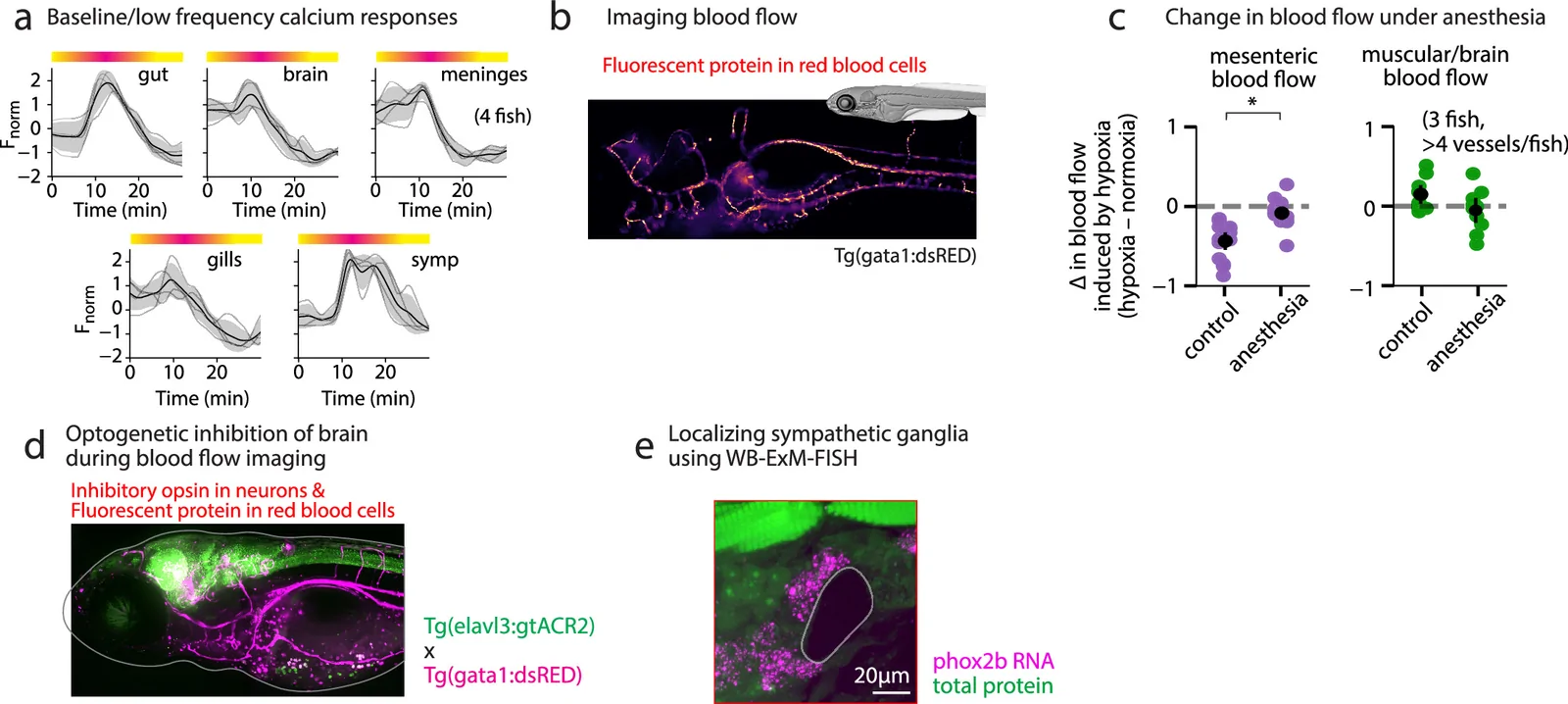
Body-wide circuit engaged in response to hypoxic stress. **a**, Hypoxia induces changes in baseline calcium levels in multiple organs. Average organ baseline calcium levels (dark: mean response, light: individual animals, shaded gray: standard deviation, N=4). **b**, Hypoxia induces reduction of blood flow to the gut. Genetic strategy for monitoring blood flow during normoxia and hypoxia using a transgenic line labeling red blood cells (*Tg(gata1:dsRED)*). **c**, Quantitative comparison of blood flow changes in brain/muscle (green) and visceral organs (purple) during hypoxia, with and without anesthesia (*N* = 3 fish; error bars: mean  ± 2 SEM; two-sided Welch’s *t*-test. Gut: *p* = 6.11 × 10^−9^, *N* = 15 vessels; brain: *p* = 0.091, *N* = 10 vessels). **d**, Double transgenic fish for optogenetic inhibition experiments. A transgenic line expressing gtACR2 (*Tg(elavl3:gtACR2-eYFP)*), an inhibitory opsin, in all neurons is crossed to a transgenic line labeling red blood cells (*Tg(gata1:dsRED)*). Maximum projection of the green channel (green), overlaid on midline plane of red channel (magenta). **e**, Localization of the sympathetic ganglia using WB-ExM-FISH. Sagittal section of animal probed for phox2b mRNA (magenta), stained with total protein stain, Alexa488-NHS (green). A cluster of phox2b+ cells localizes below the spinal cord, surrounding the cardinal vein. (WB-ExM-FISH, 7 days post-fertilization, expanded ~2×).

To systematically evaluate the systemic effects of hypoxia across the body, animals were exposed to alternating periods of normoxia (normal O_2_ levels, 21%, approximately 15 min) and hypoxia (reduced O_2_ levels, 10% O_2_, approximately 15 min; Fig. 4a). We observed widespread changes in baseline levels of calcium, quantified by computing the difference in average baseline calcium levels between normoxic and hypoxic phases, referred to as the ‘oxygen modulation score’ (Methods and Fig. 4b). Of note, gastrointestinal tissues showed a systematic increase in baseline calcium levels during hypoxia (Fig. 4c), potentially reflecting reduced calcium buffering capacity of the cells as a result of diminished oxygen availability^38^. Other organs also showed changes in baseline calcium levels, although the gastrointestinal tract, at the average tissue level, showed among the most notable initial increase in baseline calcium levels (Extended Data Fig. 11a). Given that the arterial blood supply to the brain and gut would contain similar oxygen levels, we sought to understand these disparities in responses.

While considering possible causes for the differential modulation of calcium in the visceral organs compared with the brain, we noticed that the mesenteric artery, the primary artery supplying the visceral organs, appeared constricted during episodes of hypoxia (in unregistered imaging data where tissue motion is visible). This suggests a reallocation of oxygen across the body by rerouting blood flow during physiological stress, a conserved response^39^ that has never been imaged in real time. To assess this arterial constriction in more detail, we imaged a transgenic line that selectively labels blood vessels, *Tg(flk1:dsRED-CAAX)* (Fig. 4d) and confirmed the constriction of the main mesenteric artery during hypoxia (Methods, Fig. 4e,f). As the diameter of a blood vessel is an indirect indicator of blood flow, we imaged a transgenic line labelling red blood cells, *Tg(gata1:dsRED)* (Extended Data Fig. 11b), and found a near-complete cessation of blood flow to the gut during hypoxic conditions, which recovers upon returning to normoxic conditions (Extended Data Fig. 11c and Supplementary Video 9). By contrast, blood flow to the brain and muscle remained largely unaltered, potentially explaining the difference in baseline modulation between these tissues. Thus, consistent with mammals and certain aquatic animals^39,40^, WHOLISTIC shows that hypoxia induces a rapid redistribution of blood away from the gut, and reveals the full temporal dynamics of the surprisingly fast redirection of oxygen as well as its spatial distribution, and concurrent activity changes in other cells across the body.

To test whether the reduction in gastric blood flow was neurally regulated, we inhibited the nervous system using the anaesthetic tricaine and observed that visceral blood flow was no longer diminished during hypoxia (Extended Data Fig. 11c), suggesting that the reduction in visceral blood flow is mediated by neurons, and showing that this brain–body circuit is already developed at 7 days post-fertilization. To more directly test this, we optogenetically inhibited the hindbrain during hypoxia while measuring blood flow (Fig. 4g and Extended Data Fig. 11d). We found that inhibiting the hindbrain induced, within a few seconds, the return of blood flow to the gut (Fig. 4h,i). In spite of the hypoxic condition, near-normal blood flow was maintained for as long as the hindbrain was inhibited, with no corresponding effect observed when directing the stimulation light to a control location (Fig. 4j). Thus, hindbrain activity is necessary for the constriction of the mesenteric artery during hypoxia, which we speculate serves to redistribute oxygen, thereby preserving energy for the brain and other tissues whose activity must be prioritized, and may also be a control mechanism to decrease enteric cellular activity.

During hypoxia, we also observed a set of cells in the medial plane, below the notochord, that showed a systematic and sustained increase in activity during hypoxic periods (Fig. 4k,l). Given their location, and that sympathetic neurons induce vasoconstriction via noradrenergic activation of vascular smooth muscle cells^41^, we hypothesized that this cluster represented the sympathetic ganglion. Using cell-type-specific imaging (*Tg(th:gal4; UAS:GCaMP6f)*)^42^, along with WB-ExM-IF and WB-ExM-FISH, we showed that this group of cells indeed corresponds to sympathetic neurons and that they send axons along the mesenteric artery (Fig. 4m–o and Extended Data Fig. 11e). Together, these results establish a brain-to-mesenteric artery pathway already functional at 7 days post-fertilization. Combined with optogenetic perturbation, WHOLISTIC can thus causally dissect cell-type-specific brain–body pathways engaged during physiological stress. This work, moreover, establishes the young zebrafish as a powerful model for studying the consequences of stress-induced blood shunting and, more generally, organism-wide feedback loops engaged by stress.

## Discussion

The organism is a cohesive entity, comprising networks of cells that communicate and coordinate across multiple scales^43^. Feedback loops within these networks enable animals to respond to, predict and prepare for both internal and external challenges. Although many regulatory pathways—such as those governing hunger, glucose balance and immune responses—are well characterized^44^, the complete picture of organism-wide control systems remains a formidable challenge. Given the interdependence of cellular interactions, understanding the organism as an integrated whole is essential^3,45–47^. Yet, methods that probe the entire vertebrate organism while retaining cellular-level access have remained elusive. This study introduces a novel approach for imaging and analysing genetically encoded sensors expressed ubiquitously across cells, providing unprecedented insights into cellular interactions and organism-wide control systems.

Future implementations of WHOLISTIC in freely behaving animals will enable the study of body-wide cellular control systems in naturalistic settings, rather than an embedded preparation. This will facilitate investigation of the interplay between physiological states and behavioural strategies, such as goal-driven navigation and sleep–wake transitions. A parallel route will involve experimental systems incorporating multimodal virtual-reality environments^48^ composed of visual, thermal, chemical, mechanical and other stimuli that respond to the behaviour of an animal. Further advances in protein engineering and microscopy that improve closed-loop monitoring and activation of cellular signals across the body^4^ will open new avenues of scientific inquiry.

Calcium sensors^6,23^ provide an effective proxy for cellular activity due to the ubiquitous role of calcium in cellular processes^1^. The finding that most cells exhibit calcium-activity dynamics within the range of standard sensors has enabled extraction of many cellular dynamics. However, this remains a limited view of signalling within and between cells, which occurs via a multitude of molecular, voltage and mechanical pathways. Measuring additional molecules and physical cellular properties will provide deeper insights. WHOLISTIC can be extended to co-image signals within the rapidly expanding repertoire of molecular, voltage and mechanical sensors^49–54^, providing access to additional information channels, including metabolic states, hormones, ATP and neuromodulators that mediate intercellular communication.

Given the scale and complexity of multicellular interactions, uncovering higher-order interactions will require advanced analytical and modelling frameworks leveraging modern artificial intelligence, including graph neural networks^55^, large-scale biophysical models and model-free predictive methods such as empirical dynamic modelling^56^.

Platform automation^57^ and artificial intelligence-in-the-loop systems, combining measurements of whole-body cellular dynamics with online cell-targeted perturbation, will support gaining fundamental biological insights as well as prototyping real-time medical interventions^57–59^.

The fact that all findings presented here—pertaining to different cell types, in different parts of the body, under different physiological assays and screens—arose from the same WHOLISTIC methodology underscores its broad applicability to fundamental biology and medical research. The methodology can serve as a discovery tool, whereas validation and deeper insight can be gained using more specific molecular techniques, as performed here to understand phenomena such as the brainstem’s control of blood flow redistribution during physiological stress and the molecular identification of the quiescence-related signals as originating from ependymal cells.

Cellular function is determined by molecular and biophysical properties, tissue environment and connectivity to other cells^60,61^. Combining organism-wide cellular activity imaging with whole-body expansion microscopy offers a means to bridge function and mechanism at scale, but will require the development of body-wide registration methods, capable of matching cells between in vivo and ex vivo expanded data cell by cell.

Much information throughout the body flows through the central and peripheral nervous systems, whose connectivity is becoming increasingly accessible through light-based connectomics^62^ and machine learning algorithms for connectome reconstruction^63^. This work will seed an atlas of the mechanistic and molecular basis of functional coupling at a cellular level across the organism.

Beyond fundamental biology, WHOLISTIC enables disease modelling that tracks the effects of pathologies and treatments across spatial scales, from cells to the whole body, and temporal scales, from seconds to days. Most drug-screening workflows examine selected aspects of drug action, such as molecular binding^64^, effects on individual cells or tissue subsets^65^, or behaviour^66,67^. WHOLISTIC offers a complementary lens through which to observe the effect of drugs and genetic interventions on the entire body, revealing off-target effects and system-wide interactions that would otherwise be missed when studying cells, tissues or organs in isolation.

In conclusion, WHOLISTIC bridges cellular and organismal physiology, systems neuroscience and behaviour, opening a new frontier in systems biology. By embracing organismal complexity while retaining the individual cell as a fundamental unit of analysis, this work provides a holistic yet mechanistic approach to unravelling the cellular interactions governing health and disease across organism-wide networks.

## Methods

### Experimental model and subject details

#### Zebrafish husbandry

Zebrafish were reared at 28.5 °C in 14–10-h light–dark cycles (conductivity of 1,000 μS, adjusted via Instant Ocean Sea Salt (approximately 30 g l^−1^), pH 7.0, adjusted using sodium bicarbonate)^69^. Zebrafish from 5 to 14 days post-fertilization were fed rotifers and used for experiments. All experiments complied with protocols approved by the Institutional Animal Care and Use Committee of Janelia Research Campus. Zebrafish sex cannot be determined until approximately 4 weeks post-fertilization^70^, so the sex of the experimental animals was unknown. Where relevant, fish were randomized across conditions.

No blinding was used in either data collection or analysis. Blinding during data collection was not possible because the experimental condition determined the acquisition protocol and was therefore necessarily known to the experimenter at the microscope. Blinding during analysis was not applied because all reported quantities were extracted by automated pipelines using identical parameters across conditions.

#### *Danionella cerebrum* husbandry

*Danionella cerebrum* were reared at 26.5 °C in 14–10-h light–dark cycles (conductivity of 450 μS, pH 7.5). Feeding protocols were adjusted according to age: (1) at 5–28 days post-fertilization, rotifers were administered once daily, (2) at 16–28 days post-fertilization, in addition to rotifers, GEMMA 75 was provided twice daily, and (3) at 29 days post-fertilization and beyond, the diet was composed of GEMMA 75 twice daily and Artemia once daily. Adult *Danionella cerebrum* were maintained in group housing with stock density of approximately 45 fish in 3.5-l tanks (Tecniplast). Fish younger than 6 weeks of age are sexually immature and could not be sexed; the sex of fish older than 6 weeks of age is mentioned in the main text. For egg collection, 10-cm-long custom-made acrylic tubes were used. All experiments complied with protocols approved by the Institutional Animal Care and Use Committee of Janelia Research Campus.

#### Zebrafish transgenics and transgenesis

**Extended Data Fig. 12:**
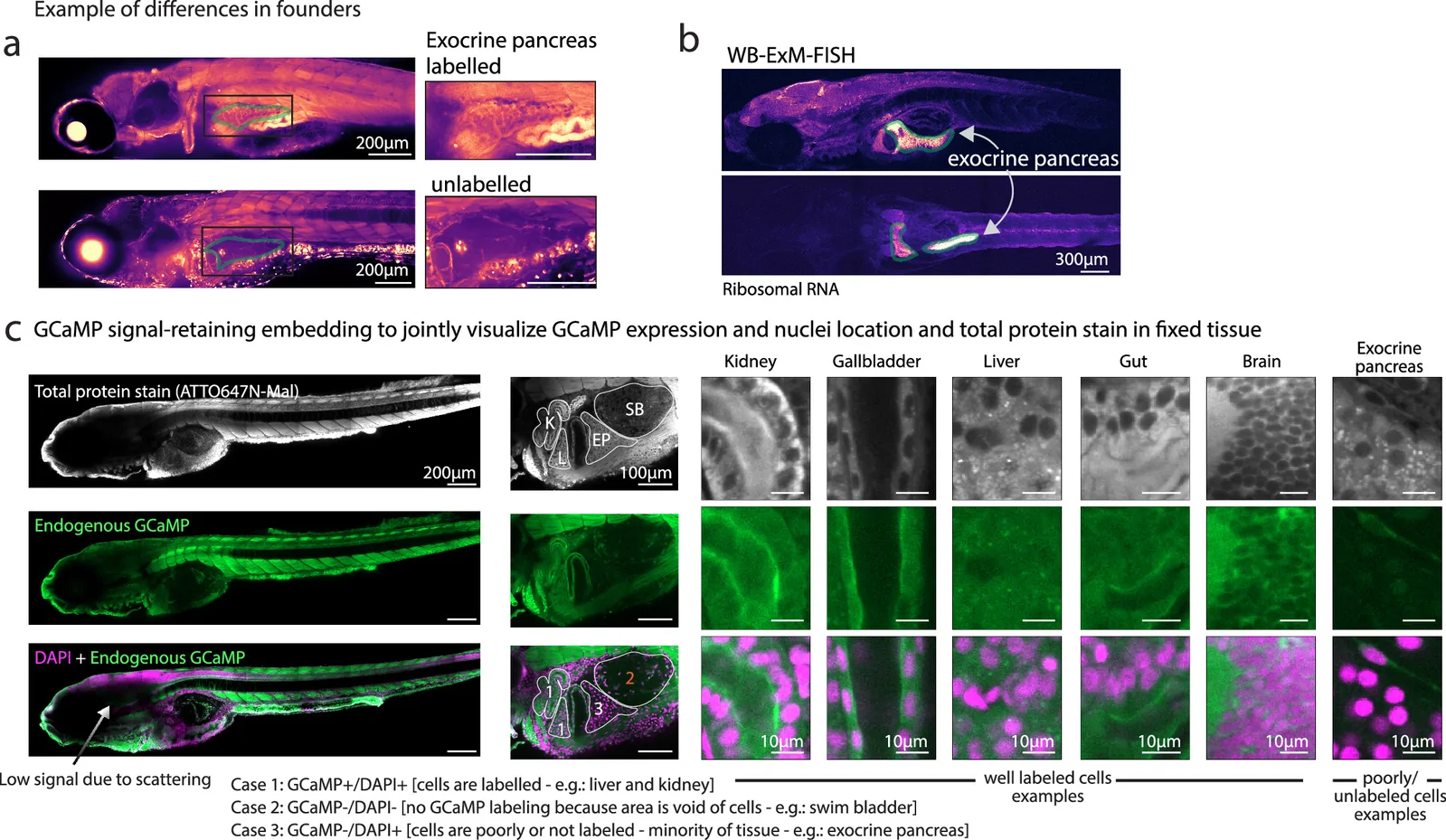
Example of differences in pancellular founders and joint visualization of GCaMP signal along with nuclei. **a**, Transgenic lines generated via Tol2 transgenesis have non-deterministic landing sites and are therefore sensitive to positional effects. Consequently, founders exhibit variable expression patterns and must be carefully screened for dense, uniform expression. Shown are offspring from two founders in which the exocrine pancreas is (*top*) or is not (*bottom*) labeled. **b**, Sagittal (*top*) and ventral (*bottom*) views of a whole-body expansion microscopy (WB-ExM) sample (7 days post-fertilization) stained for ribosomal RNA, highlighting dense labeling in the exocrine pancreas. **c**, GCaMP signal-retaining gel embedding for assessing expression density. Determining which cells express GCaMP requires unbiased localization of all cell bodies, achievable through nuclear labeling. Expansion protocols induce a drop in fluorescence signal and so could not be used. For nuclear DAPI staining, samples need to be fixed which results in more tissue scattering compared to live samples. We developed an embedding method that preserves endogenous GCaMP signal while still providing decent optical access throughout the tissue; we note that the quality of live-imaging or ExM data is still superior to this. *Left*: Example sample showing total-protein stain (ATTO647N-Maleimide, *top*), preserved endogenous GCaMP (*middle*), and DAPI (*bottom*). *Right*: Enlarged views of various tissues (pronephric kidney, gallbladder, liver, exocrine pancreas, intestine, brain) illustrating three cases: (1) cells positive for both GCaMP and DAPI; (2) regions lacking both GCaMP and DAPI, corresponding to acellular structures such as the swim bladder and notochord; (3) DAPI-positive nuclei with little to no surrounding GCaMP signal, indicating poorly labeled cells (e.g., exocrine pancreas in this founder). In general, we found the vast majority of cells to be well labeled.

Transgenic zebrafish were maintained in the *Casper* or *Nacre* background^71^. All lines were generated using the Tol2 system^72^ and genes were codon optimized using CodonZ^73^. Codon-optimized GCaMP7f and jRGECO1b were synthesized (Twist) and used for subsequent cloning. For cloning, restriction digest cloning was used throughout and all genes (*GCaMP7f*, *jRGECO1b* and *tTA*) were cloned with a preceding Kozak sequence and followed by an SV40 poly(A) signal sequence. Plasmids (150 ng μl^−1^), along with Tol2 transposase mRNA (50 ng μl^−1^), were co-injected (0.5 nl total injected volume) into one-cell stage embryos. Embryos were screened at 7 days post-fertilization for expression, and positive embryos were reared to maturity. At maturity, these adults were individually screened for dense expression in progeny, and the best founders were retained. We note that due to the non-deterministic landing site of the transgene, founders have variation in expression and need to be carefully screened for dense expression (Extended Data Fig. 12).

The ubi:tTA and TRE elements were obtained from multiple plasmids, a gift from D. Feliciano and I. Espinosa-Medina. These included a ubiquitous promoter containing vector or p5E-ubi^7^ (Addgene 27320), a vector containing the tTA advanced Tet-off transcriptional activator from pTet-Off Advanced Vector (631070, Takara) inserted into the multiple-cloning site of the pME entry vector^74^, and a vector containing the tetracycline-responsive element promoter p5E-TRE^75^. To generate the *Tg(ubi:tTA;TRE:jRGECO1b)* animals, the ubi:tTA;TRE elements were cloned and a codon-optimized jRGECO1b sequence placed downstream. To generate the *Tg(ubi:tTA);Tg(TRE:GCaMP7f)* animals, plasmids containing ubi:tTA and TRE:GCaMP7f were independently cloned and co-injected at equimolarity. To generate the *Tg(foxj1a:GCaMP7f)* animals, the *foxj1a* promoter was cloned (Addgene plasmid 163829)^36^ and a codon-optimized GCaMP7f sequence placed downstream using restriction digest cloning. To generate the *Tg(elavl3:gtACR2-eYFP)* transgenics, the promoter was cloned using a known *elavl3* promoter sequence^76^ and gtACR2-eYFP sequence placed downstream^77^. To generate the *Tg*(*β*-*actin2:mCherry-CAAX; myl7:GFP*) transgenic line the *bactin2* promoter was cloned (Addgene plasmid 82583)^78^ and the mCherry-CAAX sequence placed downstream. For optogenetic activation of motor vagal neurons, the transgenic lines *Tg(VAChTa:Gal4)*^79^ and *Tg(UAS:CoChR-eGFP)*^*jf44*^ (ref. ^13^) were utilized. To record activity of the sympathetic ganglia, the transgenic lines *Tg(th:Gal4)*^42^ and *Tg(UAS:GCaMP6f)*^*jf46*^ (ref. ^13^) were crossed and imaged. To quantify blood vessel diameter and track blood flow, the transgenic lines *Tg(flk1:dsRED-CAAX)*^80^ and *Tg(gata1:dsRED)*^81^ were imaged. To check the colocalization of neurons and astrocytes with the identified ‘brain-border’ population, and its location relative to the brain’s basement membrane, the transgenic lines *Tg(elavl3:H2B-jRGECO1b)*^82^, *Tg(gfap:jRGECO1b)*^13^ and *TgBAC(lamC1:lamC1-sfGFP)*^83^ were respectively used. Additional lines used for expansion microscopy were: *Tg(isl1CREST-hsp70l:mRFP)*^84^, *Tg(phox2bb:eGFP)*^85^ and *Tg(foxj1a:eGFP)*^86^.

#### *Danionella cerebrum* transgenics and transgenesis

To generate pigmentless *D. cerebrum* mutants, we used the CRISPR–Cas9 genome-editing technique to disrupt the function of the *mitfa* gene, following established protocols^5^ and utilizing a *mitfa*-targeting guide RNA (sequence: CAGCATTATACACTAAGAGT). Mutations were confirmed via PCR amplification and subsequent sequencing, resulting in the establishment of *D. cerebrum* *mitfa*^−/−^ colonies.

To generate the *Danionella cerebrum* transgenic line, *Tg(ubb*^*R*^:*jGCaMP8m)*, a Tol2 vector was constructed containing the *ubb*^*R*^ promoter^87,88^, provided by Balciunas and Lazutka. Following the promoter, a zebrafish codon-optimized jGCaMP8m sequence^88^ and the SV40 polyadenylation signal were arranged sequentially. This plasmid (25 ng μl^−1^), along with Tol2 transposase mRNA (20 ng μl^−1^), was co-injected (0.5 nl total injected volume) into one-cell stage *mitfa*^−/−^ embryos. Embryos at 3 days post-fertilization were screened, and those exhibiting strong jGCaMP8m expression were reared to maturity. Upon reaching adulthood, these fish were screened collectively, and the most robust founders were selected for further study.

### Experimental procedures

#### Sample preparation for functional imaging

Before imaging, zebrafish and *D. cerebrum* samples were embedded on their right side in a drop of 2% low-melting point agarose (A9414, Sigma) in a glass-bottom Petri dish (35 mm, P35G-1.5-14-C, Mattek). The right pectoral fin was moved away from the flank of the fish to point either tangentially or anteriorly such as to prevent it from covering visceral organs. Following agarose solidification, the dish was filled with E3 fish water. Samples were left to rest and settle for 30 min before the imaging session to minimize drift during the experiment. Cutaneous respiration is considered to be able to meet the oxygen needs of young zebrafish^89^ and thus agarose was not removed from the gills, nor mouth, and no oxygen perfusion was required.

#### Microscope and data acquisition

**Extended Data Fig. 13:**
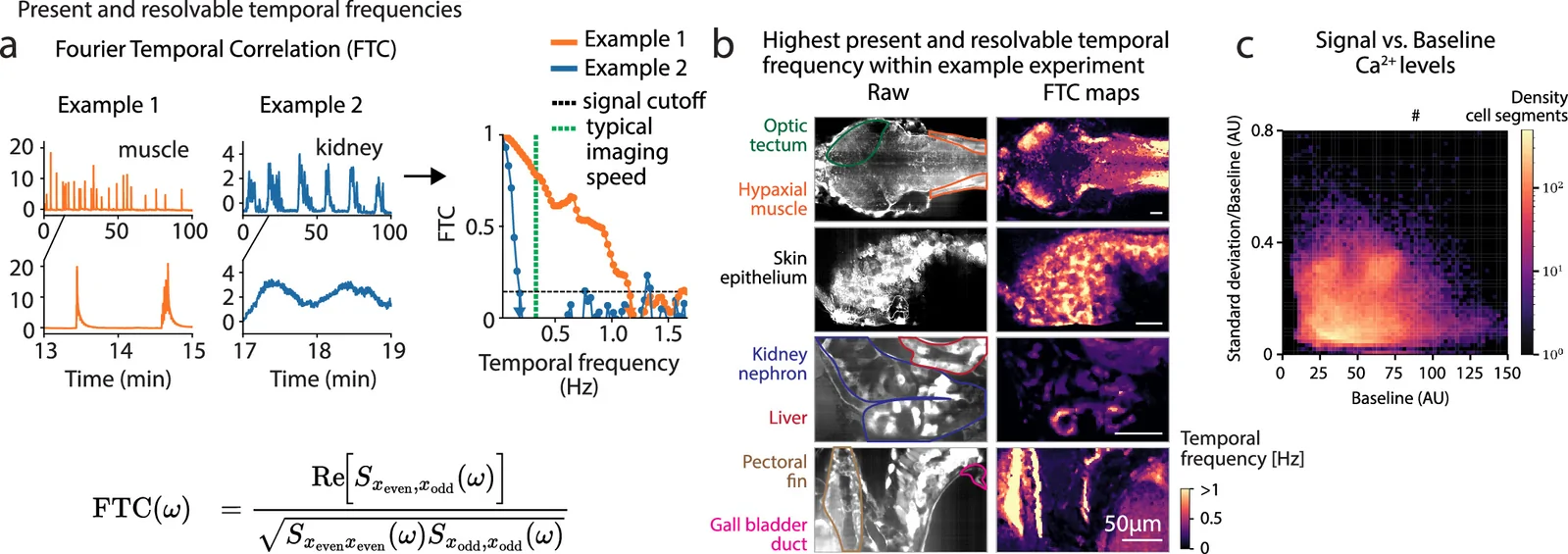
Characterization of GCaMP signal statistics. **a**, Schematic of the Fourier Temporal Correlation (FTC) method for quantifying the present and reliably resolvable temporal frequencies within a timeseries. Alternating timepoints are used as independent observations of an underlying process, their cross-spectrum is computed, and the phase correlation calculated. The temporal frequency at which the correlation falls below a user-set value (e.g., 1/7) is taken as the resolution cut-off. This provides an estimate of the minimum imaging speed required to capture temporal dynamics in different tissues without losing information. The vertical dashed line indicates the typical imaging speed used in this manuscript. **b**, Spatial maps of the highest temporal frequency at which the FTC score remained above the signal threshold (user adjustable). The scores were computed on ~45 min single plane higher-speed (>6 Hz) datasets. We note that this provides a lower bound: organs may exhibit higher frequencies that can be resolved but that were not observed in the experiment. Neurons and muscles (orange trace in a) exhibited as expected high-frequency activity, whilst the pronephric kidney (blue trace in a) only had resolvable power in the lower temporal frequencies. **c**, Baseline normalized standard deviation versus baseline brightness plotted for the dataset presented in Fig. 1l. Normalized standard deviation is not systematically lower in cells with very low or very high baseline brightness, suggesting that in the majority of the cells, the sensor does not appear to be under or over saturated.

Functional data were acquired using a Nikon spinning-disk inverted confocal microscope (CSU-W1) with a ×20 0.75 NA air objective (field of view of 800  μm × 600 μm, working distance of 1 mm) or a ×40 1.15 NA water immersion objective. For dual-colour acquisition of GCaMP7f and mCherry signals, excitation lasers at 488 nm and 594 nm were used (3–4% and 3–5% power). Emitted light was split using a 560 long-pass dichroic and passed through emission filters (525/36, 610 LP) before reaching the cameras (Hamamatsu ORCA-Fusion BT). For jRGECO1b imaging, a 561-nm laser line was used with a 610/75-nm emission filter. Camera exposure times were set between 120 ms and 200 ms. A piezo motor was used to acquire fast *z*-stacks with a typical inter-plane interval of 7–9 μm, resulting in a volumetric scan rate of approximately 0.3 Hz. Smaller *z*-steps were used for more targeted investigations (for example, during vascular imaging). There is a trade-off between the speed of volumetric imaging and sampling density (number of planes acquired), with the upper limit set by the sensor (for example, GCaMP), which acts as a low-pass filter. We have provided a quantitative means of estimating the present and resolvable temporal frequency content across tissues (Extended Data Fig. 13).

#### Ketamine treatment

Animals were imaged for a 25-min baseline period, after which ketamine was manually added to the imaging dish to achieve a final concentration of 100  μg ml^−1^ (400 μM).

#### Tricaine treatment

Animals were treated with 750 μM tricaine (MS-222, E10521-10G, Sigma) diluted in E3 water. The samples were incubated with the drug for 15 min before and throughout the duration of the experiments.

#### Cold stimulus delivery

Fish water was cooled to 10 °C, and 350 μl of cold water was manually added to the imaging dish after 5 min of baseline imaging. To rule out any neural influence or motion, fish were anaesthetized before and during the experiment with tricaine (750 μM; MS-222, E10521-10G, Sigma).

#### Hypoxia treatment

Oxygen levels were programmatically varied using an Okolab O_2_ controller module, controlled via the Nikon spinning-disk microscope software (Elements). The module varies O_2_ levels by altering the ratio of N_2_ and O_2_ mixed and the oxygen levels at the chamber inlet were recorded. A baseline period (21% O_2_) of at least 5 min was recorded, after which oxygen levels were lowered to 10% for 10–20 min, after which levels were returned to 21%. Oxygen levels were measured in the bath using an oxygen micro-optode (O_2_ MicroOptode, Unisense) inserted in the agarose (Fig. 4a). We fit a mono-exponential model to the data using the scipy.optimize.curve_fit function. The model accounts for 86% of the variance with a time constant of approximately 1.2 min. We note that hypoxia is expected to cause changes in acid–base balance within cells^90^. As the aim was to study the physiological consequences of hypoxia, we did not attempt to control tissue pH. We further note that even if the agarose is 98% water, and commonly used in the field, given that water is not flowing, this might still result in oxygen levels that are slightly lower than if there was no agarose, and if the animals were freely swimming. We therefore consider our assay to be one in which the change in oxygen levels are what is most important.

#### Optogenetics

A commercial integrated digital micromirror device (DMD) module within the Nikon spinning-disk confocal microscope was used for all optogenetic experiments in conjunction with a 488-nm LED. The duty cycle of the DMD was set to 10%, and LED power set to approximately 8–10%. A dual-colour reference image stack was acquired at the beginning of the experiment to get the anatomical location of the cells expressing CoChR–eGFP. On the basis of this 3D volume, regions of interest (ROIs) to be illuminated were defined in 3D via the graphical user interface and programmatically stored. For optogenetic activation of motor vagal cells (Fig. 2), stimulus intensity was initially calibrated by collecting a dose–response curve with progressively increasing stimulus intensity until a reliable response was observed in the neurons being stimulated (approximately 8% LED power). Once calibrated, the experimental protocol was programmed using Nikon software (Elements v5) with inter-stimulus intervals of 10 s, with ROIs selected randomly (choose without repeat) or sequentially. ROIs were illuminated for varying durations ranging from 10 to 500 ms during dose–response experiments, and for all other experiments for one frame (approximately 180 ms). The data and metadata were extracted using the Python package ND2. For each trial type (that is, specific ROI stimulated), the difference in activity pre-stimulus and post-stimulus was extracted and averaged over an approximately 5-s window (two volumes). To display the data within a single graph, the averaged stimulation-induced maps were combined, with each pixel coloured according to the ROI inducing the most change in fluorescence, alpha-weighted by the magnitude of change with alpha set to 0 when the change was below noise level (estimated by the average change induced by the control ROI). For optogenetic inhibition of the hindbrain (Fig. 4), the same stimulus intensity was used as in the optogenetic activation experiment and was confirmed to result in loss of all motor output. After 5 min of onset of lower oxygen levels (10%), an ROI over the hindbrain or the heart (control) was illuminated in alternation for 1 min followed by 1 min of no illumination. After three alternating trials of each location, oxygen levels were returned to 21%.

### Data processing workflow

#### Registration

The registration pipeline was based on local iterative motion estimation.

*Iterative patch-wise optical flow*. We referred to the static image as the ‘template image’, *I*_temp_, and referred to the data that we aimed to transform as the ‘moving image’, *I*_mov_. The key part of the registration algorithm is the iterative patch-wise optical flow module. This module estimates motion using a modified version of the optical flow algorithm^12^. It aims to minimize the intensity difference between the template and the moving image while encouraging the motion field to vary smoothly over space. We formulated an optimization problem defined by the following loss function (for clarity, the one-dimensional version is described; in practice, this is extended to three dimensions):

1 $${\mathcal{L}}(\Delta X)=\mathop{\sum }\limits_{n}^{N}\left[\mathop{\sum }\limits_{x}^{Z}{[{I}_{{\rm{t}}{\rm{e}}{\rm{m}}{\rm{p}},n}(x)-{I}_{{\rm{m}}{\rm{o}}{\rm{v}},n}(x+\Delta {x}^{n})]}^{2}+\beta {(\Delta {x}^{n}-\overline{\Delta {x}^{n}})}^{2}\right]$$

where Δ*X* ∈ *R*^*N*^ is the motion field to be estimated, Δ*x*^*n*^ is the *n*th element of Δ*X*, *N* is the total number of patches the image is split into, *Z* is the number of pixels per patch, Δ*x*^*n*^ is the estimate of the spatial displacement for patch *n* and $\overline{\Delta {x}^{n}}$ is the average Δ*x*^*n*^ for the neighbouring patches of *n*.

This original optimization problem is non-convex and cannot be efficiently solved. Therefore, we re-formulated the problem into one that can be solved iteratively through a set of convex problems using a modified version of the optical flow algorithm^12^:

2 $${\mathcal{L}}(\Delta {X}_{s})=\mathop{\sum }\limits_{n}^{N}\left(\mathop{\sum }\limits_{x}^{Z}{\Vert {I}_{{\rm{t}}{\rm{e}}{\rm{m}}{\rm{p}},n}(x)-{I}_{{\rm{m}}{\rm{o}}{\rm{v}},n}(x+\Delta {x}_{a}^{n})-\frac{\partial {I}_{{\rm{m}}{\rm{o}}{\rm{v}},n}(x+\Delta {x}_{a}^{n})}{\partial x}\Delta {x}_{s}^{n}\Vert }^{2}+\beta \Vert \Delta {x}_{a}^{n}+\Delta {x}_{s}^{n}-\overline{\Delta {x}_{a}^{n}}{\Vert }^{2}\right)$$

Computing spatial intensity gradients can be prone to noise if done pixel-wise; to overcome this, a locally averaged estimate of the spatial intensity gradient was used. The image was divided into patches of ([2*r* + 1] × [2*r* + 1]) pixels and the average spatial gradient was computed (*r* = 5 pixels). The accumulated motion $\Delta {x}_{a}^{n}$ was estimated and optimized per patch. Once convergence was reached, the motion between patch centres was linearly interpolated. The estimated displacement is also desired to be somewhat smooth over space. Therefore, the loss function was augmented with a penalty term weighted by *β*, which penalizes the difference between each motion estimate of a patch’s motion and the average of the immediately adjacent patches. This individual optimization step is a quadratic function and thus has a closed-form solution. Once a motion step Δ*X*_*s*_ was computed, the accumulated motion was updated and linearly interpolated between patch centres, then the accumulated motion was applied to the moving frame, and this was iterated until the loss ceased to decrease beyond a user-defined threshold or reached the maximum iteration number of each pyramid layer *N*_iter_. To take into account that it is the accumulated motion that ought to be spatially smooth, the terms weighted by *β* are the accumulated patch and patch neighbourhood motion. Furthermore, to enable the parallelization of calculations, the gradients of the previous time steps were used for the neighbourhood motion estimation.

*Image processing scheme and registration reliability mask*. First, one needed to define the template and the moving image. For short datasets, the first frame served as the template, and each subsequent frame was treated as the moving image. This setup allowed for the alignment of all frames one by one to the initial frame. For longer-duration datasets, where bleaching occurs, a floating template was introduced along with an initialized motion field. The floating template was defined as the median of the last *N*_template_ selected motion-corrected frames, whereas the initial motion field was chosen as the one closest to the centre of these *N*_template_ motion fields.

To enhance the algorithm’s robustness to noise, the ‘foreground’ in both the template and the current frame was defined. This step excluded regions containing moving immune cells, which appear as small, bright, connected components that otherwise introduce distortions into the estimated motion field. After pre-processing the data, pyramid downsampling was performed, a technique that reduces image resolution by iteratively smoothing and subsampling the image. This reduced the size of the template, moving image and the initialized motion field to 1/2^*L*^ of their original dimensions in the *x* and *y* directions, where *L* is the number of levels. The original size along the *z* direction was maintained. On the basis of specified patch size parameters, iterative patch-wise optical flow registration was performed on the downsampled data, which gives a rough estimate of the motion field. After this, the data were upsampled and the motion field further refined, thereby enhancing its accuracy. Once fit, using the refined motion field, the calcium channel was corrected by relocating the moved cells to their original positions as seen in the first frame. This systematic approach provides robust frame alignment and motion correction, accommodating dynamic cell movements and variations in image intensity throughout the functional recording. Finally, to filter out any data that were either not sufficiently well or unreliably registered, a registration reliability mask was computed. To identify unreliable textures, characterized by low spatial intensity gradients, the amplitude of the spatial gradient of the template image was computed and standardized using *z*-scores. Regions exhibiting a score below 0 had little texture and were flagged as potentially unreliable; in addition, the mean squared error was computed for each patch across time, and all patches whose error was above a user-set threshold were discarded from downstream analysis.

*Parameters and implementation*. The algorithm was executed with *β* = 0.01 (smoothness parameters), *r* = 5 (motion correction patch size, measured in pixels), *N*_*L*_ = 4 (number of pyramid layers used for motion correction), *N*_template_ = 5 (number of frames incorporated in the floating template), *N*_moving_ = 5 (number of frames to consider when initializing motion) and *N*_iter_ = 10 (maximum iteration number of each pyramid layer). The code was developed and run in Python and MATLAB (R2024b) and is available on GitHub (https://github.com/vruetten/wholistic_registration, https://github.com/Weizheng96/WHOLISTIC-registration).

*Validation*. To validate the registration at single-cell resolution, the transgenic line used for the registration, pancellular membrane marker (*Tg(β*-*actin2:mCherry-CAAX)*) was crossed to a sparse transgenic line, (*Tg(phox2bb:eGFP)*), to use the latter as held-out ground truth. Dual-colour data were acquired for 1 h. Using the reference channel alone, the motion field was estimated and applied to the held-out green (sparse) channel. In both unregistered and registered data, individual cells within the sparse held-out channel were manually located in the ‘template’ (first time point) and 20 and 60 min into the recording within Fiji software. Displacement distance was subsequently extracted for individual cells.

### Registration: method comparison

#### Synthetic dataset generation

To benchmark registration performance under controlled but challenging conditions, we created a library of three-dimensional simulated recordings in which the signal-to-noise ratio, motion smoothness and displacement amplitude were varied independently.

*Seed volume*. We selected an anatomically rich volume of 256 × 256 × 13 voxels and termed it the reference stack (*I*_ref_).

*Motion field synthesis*. A zero-mean, unit-variance three-dimensional Gaussian random field *G*(*x*, *y*, *z*) was convolved with an isotropic Gaussian kernel of standard deviation *σ*_*k*_. The smoothed field was normalized to unit peak magnitude and scaled by an amplitude factor *A* to yield the displacement field. Finally, we added a bias term *R* to simulate rigid deformation to the final motion field.

3 $$\Delta (x,y,z)=A\,\frac{{G}^{* }{\mathcal{N}}\,(0,{\sigma }_{k}^{2})}{{\parallel {G}^{* }{\mathcal{N}}(0,{\sigma }_{k}^{2})\parallel }_{\infty }}+R$$

Larger *σ*_*k*_ produces more spatially coherent motion, whereas increasing *A* and *R* increases the maximum voxel displacement.

*Noise injection and simulation parameters*. Additive white Gaussian noise $\eta  \sim {\mathcal{N}}(0,{\sigma }_{\eta })$ was applied independently to the reference and the warped (moving) image: 9 noise levels *σ*_*η*_ = (1.4^0^…1.4^8^), 16 *σ*_*k*_ = (5–20), and 10 amplitudes *A* = (1–10 pixels) were explored. When one parameter was swept, the remaining two were fixed at *σ*_*η*_ = 5, *σ*_*k*_ = 20 and *A* = 5 pixels. *R* was a 2D vector, of length 15 pixels added to each plane, with direction randomized between realizations. Ten stochastic realizations were generated for every parameter combination and averaged to obtain a robust estimate.

#### Performance evaluation

For every simulated dataset, we measured two scores:

1. Image fidelity: the mean squared error (MSE) between *I*_ref_ and the registered image, $\widehat{I}$.
2. Motion fidelity: the MSE between the ground-truth displacement field Δ and the field recovered by the algorithm $\widehat{\Delta }$.

Data along the borders of the image (20 pixels) were not included in the MSE as none of the registration methods can extrapolate meaningfully and so borders were discarded in downstream analysis to avoid artefacts.

#### Benchmark algorithms

WHOLISTIC registration was compared against four widely used non-rigid registration frameworks, each tuned for volumetric calcium-imaging data. The results are presented in Extended Data Fig. 2.

*WHOLISTIC registration*. Method parameters were set to pyramid_layer = 3, smooth_penalty = 0.01 and *r * = 5. In addition, we have provided the results without the pyramid registration.

*Suite2p (MATLAB GPU port)*. Multi-plane registration was enabled; the field of view was partitioned into 40 × 40 *xy* blocks with 2-pixel overlap.

*NoRMCorre*. Parameters were set to grid_size = [16, 16, 1], mot_uf = [4, 4, 1], max_shift = [15, 15, 5], max_dev = [3, 3, 1] and overlap_pre = [4, 4, 1].

*Demons (SimpleITK)*. Intensity histograms were matched (1,024 bins, 16 match points and background threshold = mean). The algorithm ran for 200 iterations with Gaussian-smoothed updates (*σ* = 0.5).

*B-spline FFD (SimpleITK)*. A 24 × 24 × 13 control-point grid was optimized with L-BFGS-B (tol = 10^−5^, 100 iterations, 5 corrections, 1,000 evaluations; cost-function convergence factor = 10^7^). Correlation similarity and linear interpolation were used; the final transform was converted to a dense displacement field.

#### Cellular segmentation

In dense volumetric fluorescence imaging, camera pixels inevitably receive photons from multiple overlapping sources due to the optical point spread function and tissue scattering. To take into account the mixed sources, we used non-negative matrix factorization, a linear source separation method that explicitly models the data as the superposition of multiple spatially overlapping sources with distinct temporal dynamics.

The registered data were segmented on a cellular scale using Voluseg, a pipeline that implements locally constrained non-negative matrix factorization to transform neighbouring correlated pixels into functional segments^13^ (https://github.com/mikarubi/voluseg/). In brief, an intensity-based brain mask was created and divided the volume into spatially contiguous three-dimensional blocks, which overlap slightly to capture cells on the borders, and cell detection was run in parallel on these through the use of Spark, a distributed cluster-computing framework. The algorithm fits the following model:

4 $${V}_{(n\times t)}\approx {W}_{(n\times c)}{H}_{(c\times t)}+{X}_{(n\times 1)}{I}_{(1\times t)}$$

where *V* is the full spatiotemporal fluorescence matrix for each block, *W* and *H* are, respectively, the spatial footprint and time series of segmented cells, and *X* and *I* are rank 1 spatiotemporal model of the background signal. The time series were then normalized (mean-subtracted, and divided by the standard deviation of the time series). This formulation explicitly represents each pixel’s fluorescence as a weighted linear combination of cellular sources contributing to that pixel, enabling computational separation of overlapping sources. This resultant normalized time series *H* is denoted *F*_norm_.

#### Denoising

Synchronous Ca^2+^ bursts arising from muscle activation result in significant fluorescence. Camera pixels that ought to only receive light from non-muscle cells can, if situated in close proximity to muscle tissue, capture some scattered photons from muscle cells, which confounds downstream analysis. Although the contribution of a constant baseline fluorescence from muscle is effectively mitigated by mean subtraction of the data, fluctuations in such fluorescence remain problematic.

To address this challenge, the timepoints of muscle activity are located and nearby highly correlated cells identified. These potentially contaminated data points were masked, and linear interpolation was applied to the time series. This method may omit activity genuinely linked to muscle activations but is a conservative approach that could be refined in future work, or two-photon microscopy can be used instead of one-photon confocal to avoid out-of-focus excitation. More specifically, the functional tissue ensembles corresponding to muscle were labelled, separating (owing to per-plane variations in contamination) and averaging them by imaging plane to form ‘muscle group’ time series. Muscle activation timepoints are determined using the probabilistic oasis model from Suite2p^91^ with parameters (window = maxmin, win baseline = 120 s and sig baseline = 2). Activation timepoints were almost always isolated, that is, neighbouring timepoints contained no muscle activation by virtue of swimming being sparse in time in these experiments. Activation events with a probability over 0.6 are deemed real, and correlated cells above 0.35 close to the muscle group (within one plane above or below the muscle group) were masked at these timepoints, and the signal was then linearly interpolated between neighbouring time points.

#### Resolvable and present spatial-frequency estimation via FRC

To quantify the resolvable and present spatial frequencies across imaged volumes, we computed the Fourier ring correlation (FRC)^92^ of pairs of independent images of the same object (*x*, *y*).

The FRC is defined as the real-valued, normalized cross-power:

5 $${\rm{FRC}}(k)=\frac{\sum _{({f}_{x},\,{f}_{y})\in k}{\rm{Re}}\,[{F}_{1}({f}_{x},\,{f}_{y})\,{F}_{2}^{* }({f}_{x},\,{f}_{y})]}{\sqrt{\left(\sum _{({f}_{x},\,{f}_{y})\in k}| {F}_{1}({f}_{x},\,{f}_{y}){| }^{2}\right)\left(\sum _{({f}_{x},\,{f}_{y})\in k}| {F}_{2}({f}_{x},\,{f}_{y}){| }^{2}\right)}}.$$

Here *k* denotes a radial shell in Fourier space, and *F*_1_(*f*_*x*_, *f*_*y*_) and *F*_2_(*f*_*x*_, *f*_*y*_) are the complex Fourier coefficients of the two independent images at frequencies *f*_*x*_, *f*_*y*_. Writing each coefficient in polar form ${F}_{j}=| {F}_{j}| \,{e}^{i{\phi }_{j}}$ and using ${\rm{Re}}[{F}_{1}{F}_{2}^{* }]=| {F}_{1}| | {F}_{2}| \cos ({\phi }_{1}-{\phi }_{2})$, we obtain:

6 $${\rm{FRC}}(k)=\frac{\sum _{({f}_{x},\,{f}_{y})\in k}| {F}_{1}({f}_{x},\,{f}_{y})| \,| {F}_{2}({f}_{x},\,{f}_{y})| \,\cos [{\phi }_{1}({f}_{x},\,{f}_{y})-{\phi }_{2}({f}_{x},\,{f}_{y})]}{\sqrt{\left(\sum _{({f}_{x},\,{f}_{y})\in k}| {F}_{1}({f}_{x},\,{f}_{y}){| }^{2}\right)\left(\sum _{({f}_{x},\,{f}_{y})\in k}| {F}_{2}({f}_{x},\,{f}_{y}){| }^{2}\right)}}.$$

This expression highlights that the FRC is the weighted mean cosine of the phase differences in shell *k*, with weights given by the product of Fourier magnitudes, normalized so that −1 ≤ FRC ≤ 1.

FRC maps were computed for volumes acquired dorsally and sagittally at 0.1625 × 0.1625 resolution. To ensure that the results were not limited by the true spatial frequency content of the specimen, we used a transgenic line labelling all cellular membranes, guaranteeing high-frequency content throughout most of the sample. We note that organs such as the ear, swim bladder and gallbladder containing acellular spaces have no high-frequency content and thus, regardless of the achievable resolution, will have a lower FRC score. The analysis was run with a patch size of 20 μm with 75% overlap. The spatial frequency at which the FRC fell below 1/7 was taken as the resolution cut-off, following established practice^92^, and was mapped across the sample (user adjustable).

#### Resolvable and present temporal-frequency estimation via FTC

To quantify the resolvable and present temporal frequencies in imaged volumes, we generalized the FRC to the time domain, which we term Fourier temporal correlation (FTC). Instead of taking two independent images of the same sample, we considered independent samples of a time series by splitting the time series into odd and even bins, *x*_1_ and *x*_2_.

We define FTC as the real-valued, normalized cross-spectrum:

7 $${\rm{FTC}}(\omega )=\frac{{\rm{Re}}[{S}_{{x}_{1},{x}_{2}}(\omega )]}{\sqrt{{S}_{{x}_{1}{x}_{1}}(\omega ){S}_{{x}_{2},{x}_{2}}(\omega )}}$$

8 $$\,=\,\frac{{\rm{Re}}| {\mathbb{E}}[{\widehat{x}}_{1}(\omega )\cdot {\overline{\widehat{x}}}_{2}(\omega )]| }{\sqrt{{\mathbb{E}}[{\widehat{x}}_{1}(\omega )\cdot {\overline{\widehat{x}}}_{1}(\omega )]{\mathbb{E}}[{\widehat{x}}_{2}(\omega )\cdot {\overline{\widehat{x}}}_{2}(\omega )]}}$$

9 $$\,=\,\frac{{\rm{Re}}\left|\frac{1}{K}{\sum }_{k=1}^{K}{\widehat{x}}_{k}(\omega )\cdot {\overline{\widehat{x}}}_{2k}(\omega )\right|}{\sqrt{\left(\frac{1}{K}{\sum }_{k=1}^{K}{\widehat{x}}_{1k}(\omega )\cdot {\overline{\widehat{x}}}_{1k}(\omega )\right)\left(\frac{1}{K}{\sum }_{k=1}^{K}{\widehat{x}}_{2k}(\omega )\cdot {\overline{\widehat{x}}}_{2k}(\omega )\right)}}$$

where ${S}_{{x}_{1},{x}_{2}}$ is the cross-spectrum between time series *x*_1_ and *x*_2_ obtained by splitting the original time series *x* into odd and even frames. The spectrum is estimated using Welch’s method, with *K* being the number of time windows the time series is split into. Like FRC, −1 ≤ FTC ≤ 1.

*Sensor bandwidth*. We note that the published single-spike half-decay times (*t*_1/2_ = 0.27 s)^88^ indicate $\tau ={t}_{1/2}/\mathrm{ln}2=0.39\,{\rm{s}}$. The fluorescence impulse response of jGCaMP7f can be approximated by a mono-exponential kernel $h(t)=\frac{1}{\tau }\,{e}^{-t/\tau }$, which has Fourier magnitude $| H(f)| =\frac{1}{\sqrt{1+{(2\pi f\tau )}^{2}}}$. Substituting *f* = 3.5 Hz gives ∣*H*(3.5 Hz)∣_*τ*_ _= 0.39_ = 0.12, that is, a nearly 10× loss in amplitude or ≈100× loss in power. Consequently, frequencies above approximately 3.5 Hz are already attenuated by nearly one order of magnitude.

#### Coherence-based spectral clustering for identification of functional tissue ensembles

To cluster the data, spectral clustering^14^ was performed using a new coherence-based distance measure to define the adjacency graph. The similarity function was defined to be: ${W}_{i,j}=\exp \,\left(-\frac{1-{\mathcal{C}}({{\boldsymbol{x}}}_{i},{{\boldsymbol{x}}}_{j})}{\tau }\right)$ where ${\mathcal{C}}({{\boldsymbol{x}}}_{j},{{\boldsymbol{x}}}_{j})(\omega )$ is the coherence between unit ***x***_*i*_ and ***x***_*j*_. This can be demonstrated to be a valid positive semi-definite kernel. Coherence is estimated using Welch’s method, that is, interpreting it as the windowed magnitude-squared coherence estimator for stationary signals:

10 $${\mathcal{C}}({\boldsymbol{x}},{\boldsymbol{y}})=\sum _{\omega }{\mathcal{C}}({\boldsymbol{x}},{\boldsymbol{y}})(\omega )=\sum _{\omega }\frac{{| {S}_{x,y}(\omega )| }^{2}}{{S}_{xx}(\omega ){S}_{y,y}(\omega )}$$

11 $$=\sum _{\omega }\frac{{| {\mathbb{E}}[\widehat{x}(\omega )\cdot \overline{\widehat{y}}(\omega )]| }^{2}}{{\mathbb{E}}[\widehat{y}(\omega )\cdot \overline{\widehat{y}}(\omega )]{\mathbb{E}}[\widehat{x}(\omega )\cdot \overline{\widehat{x}}(\omega )]}$$

12 $$=\sum _{\omega }\frac{{\left|\frac{1}{K}{\sum }_{k=1}^{K}{\widehat{x}}_{k}(\omega )\cdot {\overline{\widehat{y}}}_{k}(\omega )\right|}^{2}}{\left(\frac{1}{K}{\sum }_{k=1}^{K}{\widehat{y}}_{k}(\omega )\cdot {\overline{\widehat{y}}}_{k}(\omega )\right)\left(\frac{1}{K}{\sum }_{k=1}^{K}{\widehat{x}}_{k}(\omega )\cdot {\overline{\widehat{x}}}_{k}(\omega )\right)}$$

where *K* is the number of time windows.

This definition of coherence weights each ${\mathcal{C}}(\omega )$ at each frequency irrespective of the power in that frequency band, this can be detrimental as bands of low power can be dominated by noise. To avoid this, the measure was normalized by the total power across all frequency bands:

13 $${\mathcal{C}}({\boldsymbol{x}},{\boldsymbol{y}})=\frac{{\sum }_{\omega }{| {S}_{x,y}(\omega )| }^{2}}{{\sum }_{\omega }{S}_{xx}(\omega ){S}_{y,y}(\omega )}$$

which remains bounded between 0 and 1. Having defined this adjacency graph, standard spectral clustering was performed. The Laplacian of the adjacency matrix was computed: *L*_norm_ = *I* − *D*^−1^*W* where *D* is a diagonal matrix and *d*_*i*,*i*_ = ∑_*j*_*W*_*i*,*j*_. The lowest *N* eigenvectors of the matrix were computed and used as an encoding basis, and finally the *k*-means algorithm was run to identify clusters. The *k*-means clusters were initialized with *k*-means++^93^, an algorithm for choosing good initializations of the cluster centroids. The model was fit with: *N*_clusters_ = 400, *τ* = 0.3, for coherence estimation, a time window of approximately 8 min was used, with 80% overlap and a Hanning tapering window.

We chose to use coherence rather than correlation as the similarity metric to accommodate phase lagged or delayed activity patterns that are prevalent in our data (Extended Data Fig. 5). 

#### Hierarchical clustering and lag-regression model

Spectral clusters mapping to muscle were identified manually based on their anatomy and time series, and the mean activity of each cluster was computed. Hierarchical clustering was performed on the cluster means using the linkage function from the Python SciPy package, utilizing correlation as a metric and Ward linkage. A dendrogram was plotted, with muscle groups with greater than 0.5 correlation colour coded in similar shades, which we have denoted hyper-clusters, and shown in anatomical space with the same colour scheme. To confirm the greater synchrony of the cervical epaxial muscle with the ventral abdominal muscle, rather than with the neighbouring hypaxial muscle, the cluster corresponding to the cervical epaxial muscle was identified in different samples and cross-correlation between the activity of hypaxial and abdominal muscle computed, and a one-sided Wilcoxon signed-rank test used.

The mean activity of the muscle hyper-clusters identified in hierarchical cluster analysis was used as the basis for the regressors in the lag-regression model. A lag-regression model is a predictive model for time-series data in which a regression equation is used to predict the current value of the dependent variable, *y*(*t*), based on both the current and the past (that is, lagged), values of an explanatory variable, *x*(*t*), *x*(*t* − 1),⋯, *x*(*t* − *L*) where *L* is the maximum number of lags. This is the equivalent to assuming that the observed dependent variable arises from the convolution of the independent variable with a learnt kernel, which is parametrized by a set of weights, one for each lag:

14 $$y(t)=\mathop{\sum }\limits_{m=1}^{M}\mathop{\sum }\limits_{\tau =0}^{L}{x}_{m}(t-\tau )\cdot {k}_{m}(\tau )+{\epsilon }$$

where *M* is the number of regressors or independent variables, and *L* is the maximum number of lags.

The kernel parameters (*k*_*m*_) are fit to minimize the mean squared reconstruction error (that is, $\parallel y-\bar{y}{\parallel }_{2}^{2}$, where *y* is a vector containing the observations, and $\bar{y}$ is the model prediction), by finding the least squares solution using the numpy.linalg.solve function. This approach allows each tissue to have different temporal response properties (for example, neurons show fast responses, whereas other tissues tend to show slower, more prolonged responses to motor activity).

The mean activity of the muscle hyper-clusters (grouped clusters or cellular ensembles) were converted into a weighted binary trace by identifying the onset of motor contraction and weighting them by the power of the contraction. Contraction onset and offset were identified by finding positive deflections above the noise floor and the time at which the curve returned to the noise floor; power was defined by the area under the curve of these two time points. For each cell *y*_*i*_ ∈ *Y*, a lag-regression model was fit, ${y}_{i}(t)={\sum }_{m=1}^{M}{\sum }_{\tau =0}^{L}{x}_{m}(t-\tau )\cdot {k}_{m}(\tau )+{\epsilon }$ where *M* is the number of muscle regressors included (4–6), *L* is the number of time lags included (60 s), *y*_*i*_(*t*) and *x*_*m*_(*t*) are the activity of a cell *i* (*y*_*i*_), and muscle regressor *m* (*x*_*m*_), at time *t*. A time lag of 60 s was chosen, as we wanted to capture the relatively short-lived responses. The identified kernel values were threefold cross-validated and the average *R*^2^ value on held-out data is reported.

#### Kymograph analysis

Peak oscillation frequency was defined as the frequency with largest power excluding the zero-frequency power. To compute kymographs of ependymal cell activity from imaging data acquired from *Tg(foxj1a:GCaMP7f)* imaging, the ventral and dorsal boundaries of the brain were first anatomically identified. Line integrals perpendicular to the ventral boundary were computed, resulting in a 1 × *N* vector with *N* being the number of spatial bins used. When repeated over frames, this results in a *N* × *T* matrix. The data were denoised through spatial smoothing using a Gaussian kernel with a standard deviation of 4 pixels and temporally filtered with a bandpass Butterworth filter of order two, using cut-off periodicities of 0.5–9 min.

#### Coherence *k*-means algorithm

Below is the derivation of the algorithm used in Fig. 3k. The *k*-means algorithm alternates between computing cluster centroids and identifying the clusters to which data points belong. We used coherence as a measure of similarity. We define the following: *M* as the number of latent clusters, *K* as the number of sub-samples used to average over to calculate coherence, *T* as the total number of time points, *L* as the number of time points per sub-sample, ***x***_*i*_ ∈ *R*^*T*^ as sample *i*, ***x***_*ik*_ ∈ *R*^*L*^ as sub-sample *k* of sample *i* of length *L*, ${{\boldsymbol{\mu }}}_{m}\in {{\mathbb{R}}}^{L}$ as the centroid of cluster *m*, and ${\mathcal{C}}(x,y)(\omega )$ as the coherence between *x* and *y* at frequency *ω*.

We wished to find a centroid ***μ***_*m*_ such that the sum of the coherences of points within that cluster ${\sum }_{i}{\mathcal{C}}({{\boldsymbol{x}}}_{i},{{\boldsymbol{\mu }}}_{m})$ is maximal. This quantity is invariant to an arbitrary real scaling of ${\widehat{\mu }}_{mk}$, so the following constraint was added: *S*_*μμ*_(*ω*) = 1, that is, ${\mathbb{E}}[| {\widehat{\mu }}_{m}(\omega ){| }^{2}]=1$ so $\frac{1}{K}{\sum }_{k=i}^{K}| {\widehat{\mu }}_{mk}(\omega ){| }^{2}=1$.

Thus, we aimed to maximize:

15 $${\mathcal{L}}({\widehat{{\boldsymbol{\mu }}}}_{m}(\omega ))=\mathop{\sum }\limits_{i=1}^{N}{\mathcal{C}}({\widehat{x}}_{i}(\omega ),{\widehat{\mu }}_{m}(\omega ))=\mathop{\sum }\limits_{i=1}^{N}{\lambda }_{i}{\left|\mathop{\sum }\limits_{k=i}^{K}{\overline{\widehat{x}}}_{ik}(\omega )\cdot {\widehat{\mu }}_{mk}(\omega )\right|}^{2}$$

16 $$=\mathop{\sum }\limits_{i=1}^{N}{\lambda }_{i}\left|\mathop{\sum }\limits_{k=i}^{K}\mathop{\sum }\limits_{{k}^{{\prime} }=i}^{K}{\overline{\widehat{x}}}_{ik}(\omega )\cdot {\widehat{\mu }}_{mk}(\omega )\cdot {\widehat{x}}_{i{k}^{{\prime} }}(\omega )\cdot {\overline{\widehat{\mu }}}_{m{k}^{{\prime} }}(\omega )\right|$$

17 $$=\left|\mathop{\sum }\limits_{k=i}^{K}\mathop{\sum }\limits_{{k}^{{\prime} }=i}^{K}{\widehat{\mu }}_{mk}(\omega )\left(\mathop{\sum }\limits_{i=1}^{N}{\lambda }_{i}{\overline{\widehat{x}}}_{ik}(\omega )\cdot {\widehat{x}}_{i{k}^{{\prime} }}(\omega )\cdot \right){\overline{\widehat{\mu }}}_{m{k}^{{\prime} }}(\omega )\right|$$

18 $$=\left|\mathop{\sum }\limits_{k=i}^{K}\mathop{\sum }\limits_{{k}^{{\prime} }=i}^{K}{\widehat{\mu }}_{mk}(\omega ){A}_{k,{k}^{{\prime} }}{\overline{\widehat{\mu }}}_{m{k}^{{\prime} }}(\omega )\right|$$

where $${\lambda }_{i}=\frac{1}{{K}^{2}{S}_{{x}_{i}{x}_{i}}(\omega ){S}_{\mu \mu }(\omega )}=\frac{1}{{K}^{2}{S}_{{x}_{i}{x}_{i}}(\omega )}$$

19 $${A}_{k,{k}^{{\prime} }}(\omega )=\sum _{i\in {{\mathcal{S}}}_{m}}{\lambda }_{i}[{\widehat{x}}_{ik}(\omega )\cdot {\overline{\widehat{x}}}_{i{k}^{{\prime} }}(\omega )]$$

We note that ${A}_{k,{k}^{{\prime} }}(\omega )={A}_{{k}^{{\prime} },k}^{* }(\omega )$ so the elements form a Hermitian matrix *A*(*ω*).

Vectorizing *μ*_*m*_, the objective can be written to maximize as:

20 $${\mathcal{O}}({\widehat{{\boldsymbol{\mu }}}}_{m})={\widehat{{\boldsymbol{\mu }}}}_{m}{(\omega )}^{\top }A(\omega ){\overline{\widehat{{\boldsymbol{\mu }}}}}_{m}(\omega )\,\,{\rm{subject\; to}}\,\,{\widehat{{\boldsymbol{\mu }}}}_{m}{(\omega )}^{\top }{\overline{\widehat{{\boldsymbol{\mu }}}}}_{m}(\omega )=1$$

from which it follows that ${\widehat{{\boldsymbol{\mu }}}}_{m}(\omega )$ is the leading eigenvector of *A*(*ω*).

To initialize the group labels, the *k*-means++ algorithm^93^ was first run to choose initial centroid values. Cluster centroids were calculated as above, and cluster allocation reassigned the cluster ID based on coherence. The process was iterated until the convergence of the labels.

#### Characterizing hypoxia-induced physiological changes

*Estimation of blood vessel diameter*. To measure blood vessel diameter, a transgenic line labelling vascular endothelium (*Tg(flk1:dsRED-CAAX)*) was imaged. To ensure accurate width estimation, volumetric stacks were acquired with 2-μm *z*-spacing spanning the entire width of the mesenteric blood vessel, and maximum intensity projection of each stack was computed. A line crossing the middle of the vessel orthogonally was defined, and the kymograph was computed using Fiji’s kymograph function, resulting in a matrix with *T* rows, where *T* is the number of time points. To denoise the data, the matrix was row-wise median filtered with a window size of 5 pixels. The matrix was thresholded at the midrange value. The largest connected component was identified using the scipy-ndimage label function, which corresponds to the main blood vessel. The width of the mask at each row was computed, and the resulting time series was smoothed over time using a rolling mean with a window length of 10 time points. Finally, the width was scaled by the resolution of the data to have units of microns. Baseline and hypoxic-state vessel widths were defined as the mean width over the 1-min interval preceding the onset of the gas switch or at steady-state hypoxia (10 min following the gas switch from 21% to 10% O_2_), respectively.

*Estimation of blood flow*. To measure blood flow, a transgenic line labelling red blood cells (*Tg(gata1:dsRED)*) was imaged. For faster imaging, a single plane was acquired at 10 Hz. The plane was selected to cover the mesenteric artery, which runs parallel to the body. The frame rate was too low to track individual red blood cells; instead, local bulk blood flow was estimated by quantifying changes in fluorescence induced by red blood cells moving in and out of any specific ROI. ROIs over arteries feeding the brain, muscle and mesentery were manually defined. The absolute value temporal difference of the mean was computed for each of these ROIs. As passing red blood cells result in differing changes in magnitude depending on whether the entire or a fraction of the cell was in the ROI, the time series was clipped between 0 and 3× the median value of the beginning of the time series (normoxic period, 2 min). The time series was finally normalized by dividing by the standard deviation of this baseline period. The baseline and hypoxic-state blood flow were defined as the mean blood flow over the 1-min interval preceding the onset of the gas switch or at steady-state hypoxia (10 min following the gas switch from 21% to 10% O_2_), respectively. Change in blood flow was defined as the difference between these values and baseline blood flow (mean blood flow before gas switch, approximately 2 min). To compare the effects of optogenetic inhibition of the hindbrain and control illumination of the heart, mean blood flow over each 1-min stimulation period was computed for each condition (hindbrain or control).

*Baseline oxygen modulation score*. To quantify the effect of hypoxia on baseline calcium levels, activity traces were passed through a minimum–maximum filter using centred windows of length 3 min, and were subsequently smoothed using a Gaussian kernel with a standard deviation of 15 s. The mean baseline activity during normoxia and hypoxia over a 3-min window (taken at the end of the normoxic–hypoxic phases to ensure steady-state oxygen levels had been reached) was computed on a per-cell basis and subtracted.

### WB-ExM

Comprehensive, step-by-step protocols are available on protocols.io: WHOLISTIC ExM: whole-body expansion microscopy with immunofluorescence and histological stains (https://doi.org/10.17504/protocols.io.dm6gp9wxjvzp/v5) and WHOLISTIC ExM: whole-body expansion microscopy with fluorescence in situ hybridization (WB-ExM FISH; https://doi.org/10.17504/protocols.io.5qpvo9y89v4o/v1).

#### WB-ExM-IF

*Fixation, immunofluorescence and agarose embedding*. Whole larval zebrafish were fixed with 4% paraformaldehyde overnight at 4 °C on a shaker, then washed with 1× PBS four times for 15 min. Fixed fish were permeabilized for 5 h with 0.5% Triton X-100 in 1× PBS (PBST-0.5) at room temperature with gentle agitation. Permeabilization must be included for all specimens, whether or not they were stained with antibodies. For immunofluorescence, specimens were blocked for 3 h with blocking buffer (5% goat serum, 0.5% Triton X-100 and 0.1% Na-azide in 1× PBS) at room temperature with gentle agitation. Blocked specimens were incubated with primary antibodies diluted 1:100 in blocking buffer for 3 days at room temperature, followed by washing with PBST-0.5 three times for 2 h. Specimens were then incubated with secondary antibodies diluted 1:100 in blocking buffer for 2 days at room temperature, followed by washing with PBST-0.5 three times for 2 h. Samples were embedded in a thin layer of 1% low-melting temperature agarose to ensure the desired sample orientation. The following antibodies were used: rabbit anti-eGFP (A11122, Invitrogen), chicken anti-RFP (409006, Synaptic Systems), goat anti-rabbit Atto647N (40839, Sigma) and donkey anti-chicken Alexa568 (A78950, Invitrogen).

*Protein anchoring*. Specimens were incubated in Acryloyl-X SE at 20 µg ml^−1^ in 1× PBS (anchoring solution) for 1 h at room temperature followed by washing in 1× PBS. Anchoring solution was prepared just before use from a 10 mg ml^−1^ stock solution dissolved in anhydrous DMSO. Samples were washed in 1× PBS three times for 5 min.

*Gelation and digestion*. Specimens were incubated in the first gelation solution (10% acrylamide, 0.5 M sodium acrylate, 0.1% bis-acrylamide, 0.01% 4HT, 0.2% TEMED, 0.2% APS and 1× PBS) on ice three times for 10 min with gentle agitation. Gelation chambers were constructed using an uncharged glass slide as the bottom piece and side walls consisting of 11 layers of Scotch tape (approximately 600 μm thick to approximate the thickness of the agarose block) serving as spacers. Coverslips were placed on top of the chamber. The chambers were filled with the first gelation solution by pipetting in solution from the open side. Fully assembled chambers were carefully placed in a humidified incubator for 2 h at 37 °C for gelation. After gelation, the chambers were carefully disassembled, and extra gel was trimmed around the samples using a scalpel, leaving an approximately 2-mm margin around the fish. Gelled specimens were treated with disruption buffer (5% SDS, 50 mM Tris pH 7.5 and 200 mM NaCl in H_2_O) at 100 °C overnight in Eppendorf tubes, then washed with 1× PBS three times for 20 min.

*Re-embedding and staining*. Gelled and disrupted specimens were incubated in the second monomer solution (10% acrylamide, 0.5 M sodium acrylate, 0.02% bisacrylamide, 0.01% 4HT, 0.2% TEMED, 0.2% APS and 1× PBS; this is the same as the first gelation solution except with bisacrylamide reduced from 0.1% to 0.02%) on ice for 3 × 10 min with gentle agitation. Gels imbued with the second gelation solution were placed on uncharged glass slides with side spacers as before, composed this time of 20 layers of Scotch tape (approximately 1.2 mm). A coverslip was positioned on top as the chamber top piece. The space surrounding the first gel was filled with additional second gelation solution. Fully assembled chambers were carefully placed in a humidified incubator for 2 h at 37 °C. After gelation, the chambers were gently disassembled, and excess gel was trimmed around the embedded fish with a scalpel, leaving an approximately 1-mm margin. Gelled specimens were stained with Alexa 488-NHS ester (A20000, Thermo Fisher Scientific) and/or Atto647N-maleimide (2857, AAT Bioquest) dyes. Samples were incubated in dye solution (1:1,000 in PBS from 10 mg ml^−1^ stocks dissolved in anhydrous DMSO (D12345, Thermo Fisher)) for 1 h at room temperature with shaking, then washed three times for 1 h with 1× PBS. The re-embedding process can be repeated up to four times resulting in approximately 5× expansion.

*Imaging, tile stitching and data registration*. Samples were mounted onto a glass slide using polylysine and attached to a custom-built sample holder. Specimens were imaged on a Zeiss Z1 light-sheet microscope (×20 NA 1.0 water immersion objective), immersed in 1× PBS. Stitching and registration were done using the Imaris stitching and registration software using the default parameters.

*Online data*. An example dataset can be viewed online (https://neuroglancer-demo.appspot.com/#!gs://flyem-user-links/short/2025-01-03.165221.071748.json). This is the sample shown in Fig. 3g: double transgenic zebrafish animal labelling the ventricular and vascular systems (*Tg(foxj1a:eGFP)* × *Tg(flk1:dsRED-CAAX)*), stained against eGFP (magenta) and dsRED (green; 10 days post-fertilization, expanded approximately 2×).

#### WB-ExM-FISH

All solutions were made using molecular grade (RNase-free) water and reagents. A comprehensive, step-by-step protocol is provided in Supplementary Notes.

*Anchoring stock solutions*. Melphalan and Acryloyl-X SE (AcX) were prepared as 2.5 mg ml^−1^ and 10 mg ml^−1^ stocks, respectively, in anhydrous DMSO and stored desiccated. Melphalan-X was prepared by combining melphalan and AcX stocks at a ratio of 4:1 to yield a final concentration of 2 mg ml^−1^ each and incubating at room temperature overnight with shaking. Melphalan-X was aliquoted and stored desiccated.

*Fixation and permeabilization*. Whole larval zebrafish were fixed with 4% paraformaldehyde overnight at 4 °C on a shaker, then washed 4 × 15 min in 1× PBS. Fixed fish were permeabilized for 1 h with PBST-0.5 at room temperature with gentle agitation.

*RNA and protein anchoring*. Fixed and permeabilized fish were washed with MOPS buffer (20 mM, pH 7.7) for 30 min at room temperature. Specimens were next treated with 1 mg ml^−1^ melphalan-X supplemented with 0.1 mg ml^−1^ extra AcX diluted freshly into MOPS buffer overnight at 37 °C, followed by washing in MOPS buffer (2 × 5 min) and then PBS (2 × 5 min). Anchored specimens were then mounted on poly-L-lysine-coated coverslips with the fish positioned on its side.

*Gelation and digestion*. Mounted specimens were incubated in the first gelation solution and gelled as in the protein method variant above. In brief, this included incubating in complete gelation solution, assembling the gelation chamber, gelling at 37 °C, disassembling the chamber and trimming the gel to leave an approximately 2-mm margin around the specimen. The trimmed gel was then incubated in the first digestion solution (500 mM NaCl, 0.3% SDS, 50 mM Tris-HCl pH 8.0 and 1 mM EDTA with proteinase K diluted 1:50 from 800 U ml^−1^ stock) for 4 h at 50 °C, followed by incubation in the second digestion solution (50 mM NaCl, 1% SDS, 50 mM Tris-HCl pH 8.0 and 1 mM EDTA with proteinase K diluted 1:50 from 800 U ml^−1^ stock) overnight at 50 °C. Digested specimens were washed 4 × 15 min in 1× PBS.

*Re-embedding*. Digested specimens were re-gelled as in the protein method above. Gelled specimens were recovered into 1× PBS and trimmed, leaving an approximately 1-mm margin around the specimen.

*Probe hybridization and hybridization chain reaction*. Gels were incubated in hybridization buffer (Molecular Instruments; https://www.molecularinstruments.com/hcr-rnafish-products) for 30 min at 37 °C, followed by incubation in primary probes designed by Molecular Instruments (6 μl in 600 μl hybridization buffer, 10 nM final concentration) overnight at 37 °C. Following hybridization, gels were washed with pre-warmed (37 °C) probe wash buffer (Molecular Instruments) 3 × 30 min, then washed with pre-warmed (37 °C) 1× PBS 3 × 1 h, and once overnight at room temperature. Gels were next incubated in amplification buffer (Molecular Instruments; https://www.molecularinstruments.com/hcr-rnafish-products) for at least 30 min at room temperature. Hairpins were diluted 1:50 in amplification buffer and snap cooled by heating to 95 °C for 90 s followed by cooling at room temperature for 30 min. Gels were incubated for 4 h at room temperature in the dark in hairpin–amplification buffer mix. Amplified gels were washed in 5× SSCT (5× SSC and 0.1% Tween) 2 × 20 min at room temperature, then in 0.5× SSCT (0.5× SSC and 0.1% Tween) 2 × 40 min at room temperature and finally equilibrated in 1× PBS for 2× expansion. The following probes were used: phox2bb_*B*_3 (lot #RTG113), Th_*B*_1 (lot #RTB474), hsd3b1_*B*_5 (lot #RTG103), calca_*B*_2 (lot #RTG105) and pomca_*B*_5 (lot #RTG108).

*Stripping and re-probing*. For multi-round imaging, hybridization chain reaction amplification products and probes were stripped by digestion with DNase followed by re-probing and imaging as described for the first round, above.

*Imaging, tile stitching and data registration*. Samples were processed the same way as WB-ExM-IF samples.

#### Whole-body gel embedding to preserve endogenous fluorescence

Fixed fish were embedded in 2% low-melting-point agarose and permeabilized with 0.1% saponin in PBS overnight at 4 °C. Permeabilized fish were treated with AcX gel anchor solution (20 μg ml^−1^ Acryloyl-X-NHS in 1× PBS for 1 h at room temperature, diluted freshly from 10 mg ml^−1^ stock in anhydrous DMSO). Fish were incubated in 1× gelation solution (10% acrylamide, 5% *N*,*N*′-diallyltartardiamide, 1× PBS, 0.05% APS and 0.05% TEMED) on ice with shaking for 30 min, followed by gelation at 37 °C for 2 h under humidified nitrogen, with a coverslip placed on top of the agarose block. Embedded fish were stained in 1× PBS with DAPI and 0.3% saponin for 2 days.

#### PhotoMap

To quantify deformation introduced by expansion in an unbiased manner, we developed PhotoMap, a method that measures the gel’s deformation field from a pre-imposed reference pattern, inspired by GelMap^32^. In brief, a fluorescent gel (Extended Data Fig. 10a) was photobleached with a regular grid pattern using a two-photon microscope before expansion (Extended Data Fig. 10b), and re-imaged post-expansion (Extended Data Fig. 10c). The resulting grid deformation can be readily visualized and quantified (Extended Data Fig. 10d,e). A comprehensive step-by-step protocol is provided in Supplementary Notes.

*Fluorescent gel formation*. A fluorescent monomer was created by conjugating AF488-NHS (20 mg ml^−1^, 31 mM) to 3-aminopropyl methacrylamide (10 mg ml^−1^, 56 mM) in the presence of triethylamine (10%, 720 mM). The reagents were incubated overnight at room temperature, protected from light. Gel preparation followed the protocol described in WB-ExM-IF, except that 1:100 of the fluorescent monomer was added to the first monomer solution to render the gel intrinsically fluorescent. In brief, samples were fixed, agarose mounted, chemically anchored and polymerized, and then kept unexpanded in the gelation chamber for photobleaching.

*PhotoMap grid formation through photobleaching*. Using a large field-of-view two-photon microscope, a 50 × 50 × 50 μm grid was photobleached into the gel before expansion. Custom code was written to drive the microscope via ScanImage^94^. Regions corresponding to the eyes, which contain light-absorbing pigments, were excluded from the photobleaching pattern to prevent excessive local heating. After photobleaching, the sample was disrupted overnight, expanded and re-imaged using the same microscope at 1 × 1 × 5 μm resolution.

For PhotoMap deformation field analysis, to extract the resulting grid intersections from post-expansion images, we used a two-step procedure combining zero-mean normalized cross-correlation (ZNCC) with peak detection.

For template matching via ZNCC, we first manually selected a representative intersection from the raw image and used this as a template $T\in {{\mathbb{R}}}^{P\times Q}$. The full image $I\in {{\mathbb{R}}}^{N\times M}$ was then scanned using ZNCC, defined as:

21 $${\rm{ZNCC}}(x,y)=\frac{{\sum }_{i,j}({I}_{x+i,y+j}-{\mu }_{I})({T}_{i,j}-{\mu }_{T})}{\sqrt{{\sum }_{i,j}{({I}_{x+i,y+j}-{\mu }_{I})}^{2}}\cdot \sqrt{{\sum }_{i,j}{({T}_{i,j}-{\mu }_{T})}^{2}}},$$

where *μ*_*I*_ and *μ*_*T*_ are the local mean intensities of the image and the template, respectively, over the ROI. This was efficiently computed via fast Fourier transform (FFT)-based cross-correlation and using sliding windows.

For local peak detection, the resulting ZNCC map was passed through a local top-percentile filter, keeping the brightest 10% of pixels per patch (80 × 80 pixels). A Laplacian-of-Gaussian filter was then applied to enhance bright-on-dark blobs corresponding to grid intersections. Peak centres were finally extracted using the peak_local_max function from scikit-image, with minimum distance (50 pixels) and relative threshold parameters empirically tuned to match the grid geometry.

For quantifying local grid distortion, to assess spatial distortions in the photobleached grid post-expansion, two geometric features were computed for each grid point:

- Length deviation: the mean relative deviation of edge lengths (horizontal and vertical) from an ideal spacing *L* = 90 pixels, computed as $1-| | {\rm{vec}}(v)| /L|$.
- Angle deviation: the cosine of the angle between adjacent horizontal and vertical vectors at each point, ideally zero for orthogonal axes (that is, deviation from 90°).

These values were computed across the entire sample. To visualize and compare the spatial distortion statistics, kernel density estimates of each component (length and angle) were computed separately for on-sample and off-sample points. The analysis pipeline of PhotoMap is available (https://github.com/vruetten/PhotoMap.git).

A comprehensive, step-by-step protocol is available on protocols.io: PhotoMap: unbiased mapping of expansion microscopy deformation fields (https://doi.org/10.17504/protocols.io.261ge169wv47/v1).

#### ExM data modelling and quantification

*Building 3D ExM model*. To build the main whole-fish model, WB-ExM-Histo zebrafish datasets were acquired (total protein stain Alexa488-NHS) and imaged on a Zeiss Z1 light-sheet microscope (×20 NA 1.0 water immersion objective). The organs were manually segmented within the Amira software and the meshes imported in the software Blender. To incorporate cellular populations that have too complex morphology or spatial distribution for manual annotation, and that can be molecularly defined, such as ependymal cells and the vasculature, WB-ExM-IF data were utilized. To build meshes, WB-ExM-IF data were up-sampled in *z* by a factor of 2, converted to 8-bit format, denoised (Fiji’s Remove Outliers function), thresholded (Fiji’s Threshold function, Otsu algorithm), opened in Fiji’s 3D viewer as a surface and exported as an stl binary file, ready to be imported into Blender. Meshes from different samples were manually aligned in Blender to a common reference space using the total protein stain present in all datasets.

*Quantification of signal-to-noise ratio in ExM data*. Scattering across the sample was quantified by comparing signal-to-noise ratio within muscle fibres between the near and far-side of the sample, defined as the average difference between minimum and maximum intensity signals across muscle sarcomeres.

*Quantification of enteric motor vagus innervation*. To estimate motor vagus innervation density along the gastrointestinal tract, an animal expressing RFP under the *isl1* promoter (*Tg(isl1CREST-hsp70l:mRFP)*) was stained and expanded using WB-ExM-IF. The gastrointestinal tract was manually segmented using the Amira software. The immunofluorescence signal was averaged radially resulting in a one-dimensional histogram of average innervation density as a function of position along the gastrointestinal tract.

*Tracing of motor nerves*. A high-resolution confocal stack at 4 days post-fertilization (*Tg(VAChTa:eGFP)*) was acquired using a spinning-disk confocal microscope (0.1625 μm × 0.1625 μm × 1 μm voxel size). Nerve bundles and muscle outlines were traced manually using Amira, and the resulting tracts were imported into Blender.

#### Statistics and reproducibility

Images in Figs. 1b,d,g,h, 2h,i, 3g,i and 4b,h,k,n–o and Extended Data Figs. 1a,c,d,f,g, 4a–e, 6b, 7a,h, 8e–j, 9a,b,d–k, 11d–e and 12a–c are representative examples of data collected in at least four animals.

### Ethics statement

All animal procedures were approved by the Institutional Animal Care and Use Committee of the Howard Hughes Medical Institute, Janelia Research Campus and were conducted in accordance with the National Institutes of Health Guide for the Care and Use of Laboratory Animals.

### Reporting summary

Further information on research design is available in the Nature Portfolio Reporting Summary linked to this article.

## Online content

Any methods, additional references, Nature Portfolio reporting summaries, source data, extended data, supplementary information, acknowledgements, peer review information; details of author contributions and competing interests; and statements of data and code availability are available at https://doi.org/10.1038/s41586-026-10979-6.

## Supplementary information

Supplementary Information Notes on imaging and tissue identification; Notes on coherence-based spectral clustering; Notes on functional tissue ensembles and screening for cellular responses; Future Extensions; Table of transgenic lines generated and used; Examples of known roles of calcium in various cell types; Notes on Whole-Body Expansion Microscopy; Detailed Whole-Body Expansion Microscopy (WB-ExM) protocols and Supplementary References.

Reporting Summary

Peer Review File

Supplementary Video 1 **WHOLISTIC imaging - sagittal view**. Sagittal view of *Tg(ubi:tTA; TRE:GCaMP7f)* line with major organs indicated, imaged with 20 × 0.75 NA air objective (sped up 50 × , raw data).

Supplementary Video 2 **WHOLISTIC imaging - dorsal view**. Dorsal view of *Tg(ubi:tTA; TRE:GCaMP7f)* line with major organs indicated, imaged with 10 × 0.45 NA air objective (sped up 50 × , raw data).

Supplementary Video 3 **WHOLISTIC imaging of enteric activity highlighting visceral motion**. Sagittal view of *Tg(ubi:tTA; TRE:GCaMP7f)* line with major organs indicated, imaged with 40 × 1.15 NA water objective (sped up 50 × , raw data).

Supplementary Video 4 **Registration results**. Sagittal view of registration channel derived from *Tg(β*-*actin2:mCherry-CAAX)* line, imaged with 20 × 0.75 NA air objective. Left: data pre-registration. Right: data post-registration.

Supplementary Video 5 **WHOLISTIC imaging of cold response**. Dorsal view of *Tg(ubi:tTA; TRE:GCaMP7f)* line, imaged with 40 × 1.15 NA WI objective, in response to the delivery of cold water (sped up 50 × , raw data). Onset of cold water indicated on the top right.

Supplementary Video 6 **Whole-Body Expansion Microscopy**. Visualization of transgenic animal labeling the autonomic nervous system (*Tg(phox2bb:eGFP)*), stained against eGFP (magenta) with total protein stain (Alexa488-NHS, green) (WB-ExM-IF, expanded 2 × ).

Supplementary Video 7 **3D geometric model of young zebrafish**. 3D model derived from data generated by Whole-Body Expansion Microscopy data containing the major organs of the young zebrafish. Model built and rendered using the software Blender.

Supplementary Video 8 **Visualization of hindbrain ependymal sheet using Whole-Body Expansion Microscopy with immunofluorescence**. Visualization of ventral ependymal sheath using WB-ExM-IF. Sagittal view of transgenic sample labeling the ventricular system (*Tg(foxj1a:eGFP)*), stained against eGFP (magenta) (WB-ExM-IF, expanded 2 × ).

Supplementary Video 9 **Hypoxia-induced blood flow redirection**. Blood flow imaged during normoxia (*left*) and hypoxia (*right*). Sagittal section of a transgenic animal with fluorescently labeled red blood cells (*Tg(gata1:dsRED)*), imaged with 20 × 0.75 NA air objective (sped up 1.5 × , raw data), showing a reduction in mesenteric blood flow upon exposure to hypoxia. This reduction is readily evident in the vascular supply to the endocrine pancreas (indicated in box).

## Data Availability

Raw WHOLISTIC data (https://s3.janelia.org/ahrens-lab/ruettenv/WHOLISTIC/) and raw WB-ExM-immunohistochemistry data (https://s3.janelia.org/ahrens-lab/ruettenv/ExM/) are available. The data can also be browsed on the accompanying website (https://wholistic.janelia.org/); an example WB-ExM-immunohistochemistry dataset can be viewed (https://wholistic.janelia.org/data/exm-demo-dataset-vasculo-ventricular-system/). The plasmid map for *Tg(ubb*^*R*^*:jGCaMP8m)* is available from Addgene (plasmid 232485).

## Code availability

The 3D geometric body model is available^95^ (https://github.com/vruetten/wholebodymodel). The registration code is available in Python on GitHub^96^ (https://github.com/vruetten/wholistic_registration) and in MATLAB on GitHub^97^ (https://github.com/Weizheng96/WHOLISTIC-registration) and Zenodo^98^ (https://doi.org/10.5281/zenodo.21460338). The segmentation code is available on GitHub^99^ (https://github.com/mikarubi/voluseg) and Zenodo^100^ (https://doi.org/10.5281/zenodo.21460398). The PhotoMap analysis pipeline is available on GitHub^101^ (https://github.com/vruetten/PhotoMap) and Zenodo^102^ (https://doi.org/10.5281/zenodo.21460351).
