# Supplementary material for: Imaging cellular activity across all organs reveals body-wide circuits
Source: Nature. 2026 Sep 9;658(8138):1237–47. doi: 10.1038/s41586-026-10979-6 (PMC13716824; doi:10.1038/s41586-026-10979-6)
Supplement: Supplementary file 1 [file 41586_2026_10979_MOESM1_ESM.pdf]

nature portfolio

https://doi.org/10.1038/s41586-026-10979-6

Supplementary information

Imaging cellular activity across all organs reveals body-wide circuits

In the format provided by the authors and unedited

Supplementary information

Notes on imaging and tissue identification

We explored alternative imaging approaches, including the imaging of larger areas and perspectives from different angles, such as dorsal, ventral, and head-on views (Extended Data Fig. 1a,c,d). The identification of all areas was achieved by comparing them with established anatomical references, including histological and electron microscopy databases[1–5]. A minority, approximately 10% of other regions exhibit characteristic textures but remain more difficult to classify (Extended Data Fig. 1a). The ventral view capturing the gills exemplifies a region where it remains challenging to identify tissue boundaries and determine the identity of individual cells. We note that the eye, ear, and swim bladder introduce scattering, resulting in partial occlusion of the underlying structures, including the forebrain, thalamus, and part of the nephric duct.

Notes on coherence-based spectral clustering

Spectral clustering is a well-established method for clustering data[6,7]. The method requires one to define a similarity function between data points, which is often set to the Euclidean distance. However, such a metric is not necessarily a meaningful distance to use between two time series. Correlation would be a better distance measure, but fails to encode that time-lagged activity traces should ideally be considered as close, which results in the breaking up of such groups of traces into multiple clusters. Therefore, we aim to define a distance based on coherence (which is small when coherence is high and vice versa) that can be utilized in spectral clustering.

Background. In spectral clustering, one first defines an adjacency graph. A common choice of similarity function is the Gaussian similarity function defined between x_i and x_j as:

$$\mathcal{W}_{i,j} = \exp\left(-\frac{|x_i - x_j|_2^2}{\tau}\right) \tag{22}$$

where τ defines the range of distances of interest, or width of the neighborhood.

One then builds the normalized graph Laplacian of the adjacency matrix:

$$L = D - W \tag{23}$$

$$L_{\text{norm}} = I - D^{-1}W \tag{24}$$

where D is a diagonal matrix and $d_{i,i} = \sum_j W_{i,j}$. The lowest N eigenvectors of the matrix are then computed and used as the encoding basis set, and finally the k-means algorithm is run and outputs N clusters.

For intuition, we can interpret L as being a transition function of the consensus dynamics of the system. Assuming rows of W already sum to 1 for simplification:

$$\frac{dx}{dt} = -Lx \tag{25}$$
$$\frac{dx}{dt} = -(I-W)x = -x + \sum_j W_{i,j}x \tag{26}$$

where the fixed point is:

$$x = \sum_j W_{i,j}x \tag{27}$$

which is the weighted average of x (also showing that it has to be stable). The eigenvalues thus tell us how quickly consensus, or information, will be distributed. It can be shown that the graph has as many 0 eigenvalues as disconnected components; indeed, consensus would never occur in disconnected graph. The smaller the eigenvalues the slower the diffusion within that mode, and the similarity of the weights of the eigenvectors indicate which entries are similar. Indeed, for any vector f:

$$f'Lf = f'Df - f'Wf \tag{28}$$
$$= \sum_{i=1}^{n} d_i f_i^2 - \sum_{i,j=1}^{n} f_i f_j w_{ij} \tag{29}$$
$$= \frac{1}{2}\left(\sum_{i=1}^{n} d_i f_i^2 - 2\sum_{i,j=1}^{n} f_i f_j w_{ij} + \sum_{j=1}^{n} d_j f_j^2\right) \tag{30}$$
$$= \frac{1}{2}\sum_{i,j=1}^{n} w_{ij}(f_i - f_j)^2. \tag{31}$$

which is minimized if for large similarity w_{ij}, f_i and f_j are close.

Derivation. We first can show that if one wanted to use correlation as similarity metric, we can define a distance which depends on correlation (small when correlation is high and vice-versa) and which can be rewritten as the Euclidean distance between vectors. This then ensures that we can use this defined distance in a Gaussian kernel and that it remains positive semi-definite.

The correlation between vectors $\boldsymbol{x} \in R^N$ and $\boldsymbol{y} \in R^N$ being defined as:

$$\mathcal{R}(\boldsymbol{x}, \boldsymbol{y}) = \frac{\sum_i x_i \cdot y_i}{\sqrt{\sum_i x_i^2}\sqrt{\sum_i y_i^2}} \tag{32}$$

We assume already pre-normalized vectors:

$$x_i = \frac{x_i}{\sqrt{\sum_j x_j^2}} \tag{33}$$

$$y_i = \frac{y_i}{\sqrt{\sum_j y_j^2}} \tag{34}$$

Then:

$$|\boldsymbol{x} - \boldsymbol{y}|_2^2 = 2\sum_i (1 - x_i \cdot y_i) \tag{35}$$

$$= 2(1 - \sum_i (x_i \cdot y_i)) \tag{36}$$

$$= 2(1 - \mathcal{R}(\boldsymbol{x}, \boldsymbol{y})) \tag{37}$$

Thus, the Euclidean distance between normalized vector defines a distance based on correlation.

In the same spirit, one can define a distance based on coherence and show that it can be written as a Euclidean distance.

By definition, coherence is defined as:

$$\mathcal{C}(\boldsymbol{x}, \boldsymbol{y})(\omega) = \frac{|\sum_k \hat{x}_k(\omega)\overline{\hat{y}_k(\omega)}|^2}{\left(\sum_k \hat{x}_k(\omega)\overline{\hat{x}_k(\omega)}\right)\left(\sum_k \hat{y}_k(\omega)\overline{\hat{y}_k(\omega)}\right)} \tag{38}$$

We assume an already normalized spectrum to not carry the normalization term:

$$\sum_k \hat{x}_k(\omega)\overline{\hat{x}_k(\omega)} = 1 \tag{39}$$

Coherence can then be re-written as:

$$\mathcal{C}(\boldsymbol{x},\boldsymbol{y})(\omega) = \left(\sum_k \hat{x}_k(\omega)\overline{\hat{y}_k(\omega)}\right)\left(\sum_l \hat{y}_l(\omega)\overline{\hat{x}_l(\omega)}\right) \tag{40}$$

$$= \left(\sum_k \sum_l \hat{x}_k(\omega)\overline{\hat{y}_k(\omega)}\hat{y}_l(\omega)\overline{\hat{x}_l(\omega)}\right) \tag{41}$$

$$= \mathrm{vec}\left(\hat{X}(\omega)\overline{\hat{X}(\omega)}^\top\right)^\top \mathrm{vec}\left(\hat{Y}(\omega)\overline{\hat{Y}(\omega)}^\top\right) \tag{42}$$

where $\hat{X}(\omega) \in R^k$ is a vector of all of the $\hat{x}_k(\omega)$. Thus, we define $Z(\omega) = \mathrm{vec}\left(\hat{X}(\omega)\overline{\hat{X}(\omega)}^\top\right)$ and $W(\omega) = \mathrm{vec}\left(\hat{Y}(\omega)\overline{\hat{Y}(\omega)}^\top\right)$.

Thereby, we can define a distance based on coherence which can be written as a Euclidean distance between two vectors, thus guaranteeing that one can use this distance and have a Gram matrix that remains psd.

$$|Z(\omega) - W(\omega)|_2^2 = \sum_k \sum_l (\hat{x}_k(\omega)\overline{\hat{x}_l(\omega)})^2 + \sum_k \sum_l (\hat{y}_k(\omega)\overline{\hat{y}_l(\omega)})^2 - 2\mathcal{C}(\boldsymbol{x},\boldsymbol{y}) \tag{43}$$

$$= 2(1 - \mathcal{C}(\boldsymbol{x},\boldsymbol{y})) \tag{44}$$

Normalization. Typically coherence is averaged over frequencies:

$$\mathcal{C}(\boldsymbol{x},\boldsymbol{y}) = \sum_\omega \mathcal{C}(\boldsymbol{x},\boldsymbol{y})(\omega) \tag{45}$$

$$= \sum_\omega \frac{|S_{x,y}(\omega)|^2}{S_{xx}(\omega)S_{y,y}(\omega)} \tag{46}$$

This definition weights each $\mathcal{C}(\omega)$ at each frequency irrespective of the power in that frequency band, this can be detrimental as bands of low power can be dominated by noise. To avoid this, one can normalize by the total power in all frequency bands:

$$\mathcal{C}(\boldsymbol{x},\boldsymbol{y}) = \frac{\sum_\omega |S_{x,y}(\omega)|^2}{\sum_\omega S_{xx}(\omega)S_{y,y}(\omega)} \tag{47}$$

which remains bounded between 0 and 1. In other words, one can normalize the entire vector

$\hat{X} \in R^{K\Omega}$:

$$\hat{x}_k(\omega) \rightarrow \frac{\hat{x}_k(\omega)}{\sqrt{\sum_\omega \sum_k \hat{x}_k(\omega)\overline{\hat{x}_k(\omega)}}} \tag{48}$$

Comparing the use of correlation vs. coherence as metric in Spectral Clustering. As a motivating example as to the use of coherence vs. correlation as a metric, we run the Spectral Clustering algorithm with each metric on a single plane of WHOLISTIC imaging data keeping all the other parameters constant (τ, the length-scale and N, the number of eigenmodes/clusters). We find that coherence-based spectral clustering groups together more compactly tissues: for instance, the proximal part of the nephric tube is grouped into one cluster despite there being significant time delay between dorsal and ventral portions and the fact that they exhibit slightly different waveforms (Extended Data Fig. 3b). In contrast, correlation-based spectral clustering systematically separates them at the cost of finding other clusters in the data (Extended Data Fig. 3a). This, in part, contributed to other clusters being more mixed (Extended Data Fig. 3d-f). We note that these result depend on the number of clusters asked, and when larger numbers of clusters are searched for, both methods break up the nephric tube into disjoint sets, as indeed cells within a segment of pronephric kidney are more coherent within than outside of that segment. In passing, we note that the histogram of coherence coefficients is less uniformly distributed than those of correlation which can guide the choice of τ. Nonetheless both have a local peak at around 0.7 justifying comparing both methods with the same value of τ which we set to 0.3.

Notes on functional tissue ensembles and screening for cellular responses

We further illustrate some functional tissue ensembles identified through the WHOLISTIC workflow and how the motion flow field can be used itself to identify contractile tissue, using the biliary system as an example. While the majority of cells within the epithelium lining the gallbladder sac exhibited activity that appeared largely uncoordinated, a distinct ring-shaped area at the base of the gallbladder sac demonstrated coordinated calcium bursts (Extended Data Fig. 4a-c). By extracting the equivalent region in the original unregistered imaging data, it was found that these calcium bursts occur in synchrony with the constriction of the ring, suggesting the presence of a sphincter (Extended Data Fig. 4e). Subsequent localization of this region within Whole-Body Expansion Microscopy data revealed that this area aligns with the tripartite ductal system connecting the gallbladder, liver, and pancreas to the gastrointestinal tract. This finding posits that the identified contractile sphincter is homologous to the sphincter of Oddi[8], which has not been described in fish; in mammals, it regulates hepato-biliary and pancreatic flow into the gastroin-

testinal tract. Collectively, these findings underscore the capability of the WHOLISTIC workflow to identify small, localized functional units, such as the biliary sphincter, and to correlate their calcium dynamics with the underlying tissue contraction.

WHOLISTIC identifies new cellular response properties to stimuli. To illustrate the integration of WHOLISTIC and WB-ExM as a screening method for uncovering novel cellular properties of molecularly defined cell types, we conducted a WHOLISTIC screen to identify cells that are responsive to thermal changes, specifically a reduction in temperature (Extended Data Fig. 3). Young zebrafish were subjected to cool water (10°C) while calcium dynamics were monitored throughout the organism (see Methods). A specific cellular population exhibited robust, high-amplitude responses (Extended Data Fig. 3a-b). We matched the cells in WHOLISTIC data to the corresponding region in WB-ExM volumes through their characteristic morphology and anatomical distribution (Extended Data Fig. 3c, *left*), which showed that these cells localize to osteochondral tissue (Extended Data Fig. 3c, *center*)[9]. Finally, using WB-ExM-FISH, we established that these cells express col2a1, a marker for chondrocytes (Extended Data Fig. 3c, *right*)[10], establishing that chondrocytes are thermally responsive, displaying a rapid cold-triggered rise in intracellular calcium, a seconds-timescale responsiveness that has not been previously reported. Chondrocytes are known to maintain the extracellular matrix via calcium-dependent intracellular pathways[11,12] and express a diverse array of membrane channels that transduce external stimuli into intracellular signals[13], providing a possible mechanism for thermal responsiveness. For completeness, using WB-ExM data, we reconstructed the full skull of young zebrafish, which is the most challenging tissue in the specimen to expand (Extended Data Fig. 3d, see Methods). This example illustrates how combining WHOLISTIC and WB-ExM allows for the integration of functional properties, molecular cellular identity, and macroscopic anatomy, facilitating the discovery of previously unknown cellular properties.

WHOLISTIC identifies new cellular response properties to drugs. As a demonstration of using WHOLISTIC for drug screening, we performed a WHOLISTIC screen for cells that respond to ketamine, a dissociative anesthetic (Extended Data Fig. 4). Quantifying the strongest responding cells by ranking F_{norm} increases, we identified a functional ensemble that aligns with the outer perimeter of the brain, likely corresponding to the brain meninges (Extended Data Fig. 4a), which is responsive to ketamine (Extended Data Fig. 4b-c). While the effects of ketamine on neurons and glia are documented[14], the role of the meninges is less studied; meninges have not yet been functionally imaged in any species in vivo[15] and remain largely uncharacterized in zebrafish. With no existing selective driver line[16], they are difficult to segment based on anatomy due to the thinness of the tissue. This highlights how WHOLISTIC activity analysis is able to delineate functional ensembles that are otherwise challenging to label – i.e., identify, within the many cells in the body, small

groups of cells with specific dynamics that would otherwise remain undetected.

Future Extensions

We foresee multiple extensions of WHOLISTIC which would extend the scope of this system. In this instantiation, a) samples are embedded in agarose which limits their movement; b) moreover, to best image the viscera, fish are placed on their sides which is not their natural posture; c) furthermore, spinning-disk confocal microscopy was used which induces more bleaching than light-sheet microscopy or 2-photon microscopy and is relatively slower, and d) finally, with the main objective used in this study, 20× 0.75NA, the field of view was restricted to 800 µm ×800 µm. To overcome this, we foresee using a large field of view 2-photon microscope[17] and positioning a 45º mirror next to the fish, making it possible to image the entire length of the fish, positioned in its natural posture, whilst providing access to both the brain and viscera (via the mirror) simultaneously. Such a microscope also includes a resonant scanner and offers the capacity to do dual plane imaging, which would double the frame rate; faster imaging rates could also be achieved through the use of multi-beam optics[18], or Light Beads Microscopy[19]. Similar extensions could be made to freely swimming imaging setups[20] to enable whole-body imaging in freely behaving animals. Furthermore, GCaMP has primarily been optimized for imaging neuronal calcium dynamics, and the dynamic range of the sensor might not be optimal for all other cell types. Whilst we did not observe saturation of variance in cells with high baseline levels (Extended Data Fig. 13), this does not rule out sensor saturation in certain cell types. Quantitative calcium measurements using fluorescence life-time imaging would be the definitive way of assessing the dynamic range of calcium concentrations across cells of the body. Regarding cellular-scale segmentation, while NMF-based source separation is the standard approach in volumetric calcium imaging and substantially reduces signal mixing, complete crosstalk elimination can never be fully guaranteed. This motivated our validation strategy of independently confirming key biological findings using cell-type-specific transgenic lines (e.g., ependymal cells - Fig. 2i-k, Fig. 3i-j, sympathetic neurons - Fig. 4m). A more comprehensive validation approach for future work would be to co-express a randomly sparse, mosaic calcium indicator of a different color and analyze correspondence between dense and sparse datasets across all tissue types. Finally, regarding transgenic coverage, while we qualitatively demonstrate broad pancellular expression, rigorous, automated and scalable quantification of the fraction of labeled cells proved technically challenging (Extended Data Fig. 12c). Future work would benefit from generating a new transgenic line co-expressing GCaMP with a spectrally distinct nuclear marker under the same promoter, enabling unambiguous, automated quantification of labeling density across all tissues.

Table of transgenic lines generated and used

Transgenic Line	Target Cell Population	Protein Function	Source	Species	References
Tg(ubi:tTA; TRE:jRGECO1b)	Pancellular	Red calcium indicator	generated	Danio rerio	Mosimann et al. (2011) | Dana et al. (2016)
Tg(ubi:tTA); Tg(TRE: GCaMP7f)	Pancellular	Green calcium indicator	generated	Danio rerio	Mosimann et al. (2011) | Dana et al. (2019)
Tg(ubbR:jGCaMP8m)	Pancellular	Green calcium indicator	generated	Danio rerio	Bakunaite et al. (2024) | Zhang et al. (2023)
Tg(foxj1a:GCaMP7f)	Motile ciliated cells (ependymal cells)	Green calcium indicator	generated	Danio rerio	Zhang et al. (2020) | Dana et al. (2019)
Tg(elavl3:gtACR2-eYFP)	Panneuronal	Inhibitory opsin & yellow fluorescent marker	generated	Danio rerio	Park et al. (2000) | Mohammad et al. (2017)
Tg(β-actin2:mCherry-CAAX; myl7:GFP)	Pancellular	Red membrane marker & Green cardiac marker	generated	Danio rerio	Fowler et al. (2016)
Tg(VAChTa:Gal4); Tg(UAS: CoChR-eGFP)jf44	Cholinergic neurons (motor neurons)	Excitatory opsin & green fluorescent marker		Danio rerio	Taniguchi et al. (2017) | Mu et al. (2019)
Tg(th:Gal4); Tg(UAS: GCaMP6f)jf46	Catecholaminergic neurons (sympathetic ganglia)	Green calcium indicator		Danio rerio	Li et al. (2015) | Mu et al. (2019)
Tg(flk1:dsRED-CAAX)	Vascular endothelial cells	Red membrane marker		Danio rerio	Fujita et al. (2011)
Tg(gata1:dsRED)	Erythrocytes	Red fluorescent marker		Danio rerio	Traver et al. (2003)
Tg(isl1CREST-hsp70l: mRFP)	Motor neurons	Red fluorescent marker		Danio rerio	Grant et al. (2010)
Tg(phox2bb:eGFP)	Autonomic nervous system	Green fluorescent marker		Danio rerio	Nechiporuk et al. (2007)
Tg(foxj1a:eGFP)	Motile ciliated cells (ependymal cells)	Green fluorescent marker		Danio rerio	Grimes et al. (2016)
Tg(gfap:jRGECO1b)	Glia	Red calcium indicator		Danio rerio	Mu et al. (2019)
Tg(elavl3:H2B-jRGECO1a)	Neuronal	Nuclear localized red calcium inidcator		Danio rerio	Yang et al. (2022)
Tg(ubbR:jGCaMP8m)	Pancellular	Green calcium indicator	generated	Danionella cerebrum	Bakunaite et al. (2024) | Zhang et al. (2023)
TgBAC(lamC1:lamC1-sfGFP)	Basement membrane	Green fluorescent marker		Danio rerio	Yamaguchi, N., Zhang, Z., Schneider, T. et al. Rear traction forces drive adherent tissue migration in vivo. Nat Cell Biol 24, 194–204 (2022). https://doi.org/10.1038/s41556-022-00844-9
mitfa(-/-)	Melanocytes	Missense mutant	generated	Danionella cerebrum	Schulze et al. (2018)

Example of known roles of calcium in various cell types

An overview of calcium's established roles across a broad range of cell types, with associated references. This survey is illustrative rather than exhaustive.

Cell type	Associated Organ/System	Example of role of calcium in cell type	Reference	Reference URL
Neuron	Nervous system (CNS/PNS)	Ca^{2+} influx through voltage-gated CaV channels couples electrical activity to neurotransmitter release by binding synaptotagmins to trigger synaptic vesicle fusion; it also regulates synaptic plasticity and gene transcription.	Sudhof TC. Calcium control of neurotransmitter release. Cold Spring Harbor Perspectives in Biology. 2012 Jan;4(1):a011353. https://doi.org/10.1101/cshperspect.a011353	https://cshperspectives.cshlp.org/content/4/1/a011353.full
Astrocyte	Nervous system (CNS)	Ca^{2+} elevations in astrocytes mediate ATP release.	Khakh, B.S. & McCarthy, K.D. (2015). Astrocyte calcium signaling: from observations to functions and the challenges therein. Cold Spring Harb Perspect Biol. 7(4):a020404.	https://pubmed.ncbi.nlm.nih.gov/25605709/
Microglia	Nervous system (CNS)	Ca^{2+} transients control motility and release of cytokines.	Kettenmann, H. et al. (2011). Physiology of microglia. Physiol Rev. 91(2):461-553.	https://pubmed.ncbi.nlm.nih.gov/21527731/
Radial glial cell	Nervous system (CNS)	Ca^{2+} dynamics regulate neural stem cell proliferation/differentiation. Ca^{2+} oscillations influence cell-cycle progression.	Weissman, T.A. et al. (2004). Calcium waves propagate through radial glial cells and modulate proliferation in the developing neocortex. Neuron. 43(5):647-61.	https://pubmed.ncbi.nlm.nih.gov/15339647/
Oligodendrocyte	Nervous system (CNS)	Ca^{2+} transients control myelin sheath growth. Activity-dependent Ca^{2+} in sheaths promotes actin remodeling and elongation.	Oligodendrocyte calcium signaling promotes actin-dependent myelin sheath extension	https://pubmed.ncbi.nlm.nih.gov/38177161/
Photoreceptors	Eye (retina)	Ca^{2+} controls phototransduction adaptation and synaptic release.	Pugh, E.N. Jr. & Lamb, T.D. (2000). Phototransduction in vertebrate rods and cones: molecular mechanisms of amplification, recovery and light adaptation. Handb Biol Phys. 3:183-255.	https://www.sciencedirect.com/science/article/pii/S1383812100800081
Inner ear hair cell	Ear (auditory/vestibular)	Mechanotransduction induces Ca^{2+} entry and contributes to synaptic release.	Fuchs, P.A. (2005). Time and intensity coding at the hair cell's ribbon synapse. J Physiol. 566 (Pt 1):7-12.	
Neuromast hair cell	Lateral line	Ca^{2+} involved in mechanotransduction and synaptic release.	Synaptically silent sensory hair cells in zebrafish are recruited after damage	https://www.nature.com/articles/s41467-018-03806-8
Endothelial cell	Vasculature	Ca^{2+} controls permeability and angiogenesis.	Endothelial Cell Calcium Signaling during Barrier Function and Inflammation	https://pmc.ncbi.nlm.nih.gov/articles/PMC7074364/
Cardiomyocyte	Heart	Excitation-contraction coupling via Ca^{2+}-induced Ca^{2+} release. L-type Ca^{2+} influx triggers RyR-mediated SR Ca^{2+} release, activating troponin C.	Bers, D.M. (2002). Cardiac excitation-contraction coupling. Nature. 415(6868):198-205.	https://pubmed.ncbi.nlm.nih.gov/11805843/
Mural cell (pericyte/smooth muscle)	Vasculature	Pericyte/smooth muscle contraction depends on Ca^{2+}-calmodulin-MLCK. Vasoactive signals elevate Ca^{2+} to control tone and capillary diameter.	Hill-Eubanks, D.C. et al. (2011). Calcium signaling in smooth muscle. Cold Spring Harb Perspect Biol. 3(9):a004549.	https://pubmed.ncbi.nlm.nih.gov/21709182/
Erythrocyte (red blood cell)	Blood	Ca^{2+} is involved in regulating volume and intercellular adhesion.	Stimulation of human red blood cells leads to Ca2+-mediated intercellular adhesion	https://pubmed.ncbi.nlm.nih.gov/21616535/
Thrombocyte (platelet-equivalent)	Blood	Platelet activation and secretion require Ca^{2+}. Ca^{2+} release/influx drives shape change, integrin activation, granule exocytosis.	Varga-Szabo, D. et al. (2009). Calcium signaling in platelets. J Thromb Haemost. 7(7):1057-66.	https://pubmed.ncbi.nlm.nih.gov/19422456/
Skeletal muscle fiber	Muscle (somitic)	Depolarization triggers SR Ca^{2+} release and contraction. DHPR-RyR coupling releases Ca^{2+}; troponin C binding initiates cross-bridge cycling.	Melzer, W. et al. (1995). The role of Ca^{2+} ions in excitation-contraction coupling of skeletal muscle fibres. Biochim Biophys Acta. 1241(1):59-116.	https://pubmed.ncbi.nlm.nih.gov/7742348/
Smooth muscle cell	Muscle (visceral/vascular)	Ca^{2+}-calmodulin-MLCK pathway drives contraction.	Nelson et al. (2011). Calcium Signaling in Smooth Muscle. Cold Spring Harbor Perspectives in Biology.	https://pmc.ncbi.nlm.nih.gov/articles/PMC3181028/
Chondrocyte	Cartilage	Ca^{2+} mediates mechanotransduction and response to osmotic stress	Li W, Zhou Y, Han L, Wang L, Lucas Lu X. Calcium signaling of primary chondrocytes and ATDC5 chondrogenic cells under osmotic stress and mechanical stimulation. J Biomech. 2022 Dec;145:111388. doi: 10.1016/j.jbiomech.2022.111388. Epub 2022 Nov 17. PMID: 36413831; PMCID: PMC10472919.	https://pmc.ncbi.nlm.nih.gov/articles/PMC10472919/
Osteoblast	Bone	Regulates osteoblast differentiation.	Hao Y, Yang N, Sun M, Yang S, Chen X. The role of calcium channels in osteoporosis and their therapeutic potential. Front Endocrinol (Lausanne). 2024 Aug 7;15:1450328. doi: 10.3389/fendo.2024.1450328. PMID: 39170742; PMCID: PMC11335502.	https://pmc.ncbi.nlm.nih.gov/articles/PMC11335502/
Osteocyte	Bone	Ca^{2+} transients encode mechanical load, induce vesicular release and induce actin contraction.	Morrell, A.E., Brown, G.N., Robinson, S.T. et al. Mechanically induced Ca2+ oscillations in osteocytes release extracellular vesicles and enhance bone formation. Bone Res 6, 6 (2018). https://doi.org/10.1038/s41413-018-0007-x	https://www.nature.com/articles/s41413-018-0007-x

Cell type	Associated Organ/System	Example of role of calcium in cell type	Reference	Reference URL
Osteoclast	Bone	Ca^{2+} regulates cytoskelatal organization, elevated calcium inhibits resorbing activity.	Li, Zhenpeng, Kangmei Kong, and Weili Qi. "Osteoclast and its roles in calcium metabolism and bone development and remodeling." Biochemical and biophysical research communications 343.2 (2006): 345-350.	https://www.sciencedirect.com/science/article/pii/S0006291X06004608#section.0070
Notochord cell	Axial skeleton (embryonic)	Ca^{2+} contributes to cell elongation and intercalation.	Mapping calcium dynamics in a developing tubular structure Jorgen Hoyer, Morsal Saba, Daniel Dondorp, Kushal Kolar, Riccardo Esposito, Marios Chatzigeorgiou bioRxiv 2020.10.16.342535; doi: https://doi.org/10.1101/2020.10.16.342535	https://www.biorxiv.org/content/10.1101/2020.10.16.342535v1.full
Goblet cell	Intestine	Ca^{2+} triggers mucin granule exocytosis.	Vivek Malhotra (2013) TRPM5-mediated calcium uptake regulates mucin secretion from human colon goblet cells eLife 2:e00658.	https://elifesciences.org/articles/00658#content
Hepatocyte	Liver	Hormone-evoked Ca^{2+} waves induce glucose mobilisation through regulation of gluocogenolysis and neoglucogenesis.	Humbert, Alexandre, et al. "Calcium signalling in hepatic metabolism: Health and diseases." Cell (	https://www.sciencedirect.com/science/article/pii/S0143416023000921#sec0002
Pancreatic alpha cell	Pancreas (endocrine)	Voltage-gated Ca^{2+} drives glucagon release at low glucose.	Rorsman P, Braun M, Zhang Q. Regulation of calcium in pancreatic α- and β-cells in health and disease. Cell Calcium. 2012 Mar-Apr;51(3-4):300-8. doi: 10.1016/j.ceca.2011.11.006. Epub 2011 Dec 15. PMID: 22177710; PMCID: PMC3334273.	https://pmc.ncbi.nlm.nih.gov/articles/PMC3334273/
Pancreatic beta cell	Pancreas (endocrine)	Ca^{2+} influx directly triggers insulin exocytosis. KATP closure→depolarization→Cav channels→Ca^{2+}-triggered granule fusion.	Rorsman, P. & Ashcroft, F.M. (2018). Pancreatic β-cell electrical activity and insulin secretion: of mice and men. Physiol Rev. 98(1):117-214.	https://pubmed.ncbi.nlm.nih.gov/29212789/
Juxtaglomerular cells	Kidney (glomerulus)	Ca^{2+} inhibits renin secretion.	Staruschenko A, Alexander RT, Caplan MJ, Ilatovskaya DV. Calcium signalling and transport in the kidney. Nat Rev Nephrol. 2024 Aug;20(8):541-555. doi: 10.1038/s41581-024-00835-z. Epub 2024 Apr 19. PMID: 38641658; PMCID: PMC12036682.	https://pmc.ncbi.nlm.nih.gov/articles/PMC12036682/#S15
Chromaffin cell	Head kidney (adrenal medulla homolog)	Depolarization-triggered Ca^{2+} induces catecholamine secretion.	García, Antonio G., et al. "Calcium signaling and exocytosis in adrenal chromaffin cells." Physiolo	https://journals.physiology.org/doi/full/10.1152/physrev.00039.2005
Interrenal steroidogenic cell	Head kidney (adrenal cortex homolog)	Sustained Ca^{2+} influx increases steroidogenesis.	Rossier, M. F. "T-type calcium channel: a privileged gate for calcium entry and control of adrenal steroidogenesis. Front Endocrinol. 2016; 7: 43."	https://pmc.ncbi.nlm.nih.gov/articles/PMC4873500/
Pituitary corticotroph (ACTH)	Pituitary	CRH stimulates Ca^{2+} signaling to release ACTH. cAMP-PKA primes secretion; Ca^{2+} is final trigger for granule fusion.	Tse, Amy, Andy K. Lee, and W. Tse Frederick. "Ca2+ signaling and exocytosis in pituitary corticotropes." Cell Calcium 51.3-4 (2012): 253-259	https://www.sciencedirect.com/science/article/pii/S0143416011002363
Kupffer cell (liver macrophage)	Liver	Ca^{2+} contributes to Kupffer cell M2 polarization.	Xu, Xue-song, et al. "SCARF1 promotes M2 polarization of Kupffer cells via calcium-dependent PI3K-AKT-STAT3 signalling to improve liver transplantation." Cell proliferation 54.4 (2021): e13022.	https://onlinelibrary.wiley.com/doi/10.1111/cpr.13022
Ultimobranchial (calcitonin) cell	Ultimobranchial gland	Systemic Ca^{2+} regulates calcitonin secretion. High plasma Ca^{2+} stimulates calcitonin release to lower Ca^{2+}; Ca^{2+} acts as both signal and target.	Fudge, N.J., Kovacs, C.S. Physiological studies in heterozygous calcium sensing receptor (CaSR) gene-ablated mice confirm that the CaSR regulates calcitonin release in vivo . BMC Physiol 4, 5 (2004). https://doi.org/10.1186/1472-6793-4-5	https://bmcphysiol.biomedcentral.com/articles/10.1186/1472-6793-4-5#:~:text=The%20calcium%20sensing%20receptor%20(CaSR,responsiveness%20of%20calcitonin%20to%20calcium.
Skin epithelial cells	Skin	Ca2+ signaling drives cell cycle progression	Moore, Jessica L., et al. "Cell cycle controls long-range calcium signaling in the regenerating epid	https://rupress.org/jcb/article/222/7/e202302095/214066/Cell-cycle-controls-long-range-calcium-signaling
Enteroendocrine cell	Intestine	Stimulus-secretion coupling via Ca^{2+}. Nutrient cues evoke Ca^{2+} to release peptides (e.g., 5-HT, CCK);	Davison, Adam, Frank Reimann, and Fiona M. Gribble. "Molecular mechanisms of stimulus detect	https://www.sciencedirect.com/science/article/pii/S0959
Monocyte/Macrophage	Immune (innate)	Purinergic receptors is strongly coupled to Ca2+-signaling; Ca2+ involved in phagosome maturation, engulfment of apoptotic cells, migration.	Desai, Bimal N., and Norbert Leitinger. "Purinergic and calcium signaling in macrophage function	https://pmc.ncbi.nlm.nih.gov/articles/PMC4245916/

Notes on Whole-Body Expansion Microscopy

Expansion Microscopy (ExM) is a form of super-resolution microscopy. Unlike standard super-resolution methods, which either focus on altering the hardware or software, ExM focuses on altering the object to be imaged itself, making it physically larger[21], resulting in both high quality clearing and better effective resolution.

Gel engineering. We found, as previously reported[22], that having a harsh digestion with proteinase K (proK) could allow for more homogeneous expansion; however, expansion quality and antibody signal retention was variable between specimens, limiting the reliability and utility of the method. As a result, we turned to high temperature, high detergent disruption, in which heat is used to denature proteins, as reported in[23]. We reasoned that such hydrolysis-based disruption would result in chemical cleavage of proteins that would be less subject to crowding or to differences in local tissue environment as compared to enzymatic digestion. This approach indeed improved the robustness of digestion; however, as we began working with older samples, propagating cracks re-appeared. Over the course of embryological development, tissue heterogeneity throughout the animals dramatically increases, with for instance the rapid development of cartilage beginning at 6 days post fertilization, which progressively expands and ossifies. After trying a variety of gel recipes to address this, we found that an intermediate monomer concentration gel recipe was least prone to crack formation, and that by re-embedding immediately after disruption, without reducing salt concentration or expanding more than $\sim 1.5\times$, strongly reduced subsequent crack formation and that any cracks that did form (typically in cartilage) did not propagate into neighboring tissues (Extended Data Fig. 3i). An intermediate monomer concentration may produce a better match to tissue elasticity, while the modest first expansion enables more uniform access to the next round of polymers, thereby making the polymer density more homogeneous throughout irrespective of variation in tissue structure. This allows the expansion of zebrafish up to 14 days post fertilization as well as mature *Danionella* (Extended Data Fig. 3i-j).

Immunofluorescence optimization. We found that strongly permeabilizing the fish was critical to avoid the skin impeding antibody access to the tissue, as well as increasing antibody (AB) concentration, incubation time, and temperature in the case of high-abundance targets. Increasing antibody concentration comes at the risk of higher background signal; however, a titration curve of antibody concentration vs. signal showed that the gain in signal far outweighs small increases in background and that at 1:100 we are still not reaching epitope saturation. With long incubations in rich serum, bacterial growth is more likely to occur leading to nonspecific background staining; this can be resolved by processing the samples with minimal delay and adding sodium azide from the point of fixation onward.

Sample mounting and overcoming edge effects. Since the head of the sample is generally wider

than its body, placing samples on their sides often results in slanted samples. Despite this not being a problem for local measurements, this can result in having to acquire a much larger field of view and render sample alignment to a reference highly laborious, requiring hours of manual work. We found that fixed samples can be embedded in low melting temperature agarose (0.7%) where it is easy to optimally orient the sample, stabilizing it in place. Following high-temperature denaturation, agarose pre-embedding did not appear to hinder the ExM expansion process. This process results in more standardized samples. Another related issue was the changes in refractive index that can occur at the edges of the gel as a result of boundary conditions in the gelation process. We resolved this by increasing the agarose gel thickness, ensuring that the sample was embedded away from either surface of the gel preventing edge effects.

Detailed Whole-Body Expansion Microscopy (WB-ExM) protocols.

Detailed protocols can be found at protocols.io:

WHOLISTIC ExM: Whole-Body Expansion Microscopy with Immunofluorescence and Histological Stains : https://dx.doi.org/10.17504/protocols.io.dm6gp9wxjvzp/v5.

WHOLISTIC ExM: Whole-Body Expansion Microscopy with Fluorescence Insitu Hybridization (WB-ExM FISH) : https://dx.doi.org/10.17504/protocols.io.5qpvo9y89v4o/v1.

PhotoMap: unbiased mapping of Expansion Microscopy deformation fields : https://dx.doi.org/10.17504/protocols.io.261ge169wv47/v1.

References

[1] Jean E. Copper, Lynn R. Budgeon, Christina A. Foutz, Damian B. van Rossum, Daniel J. Vanselow, Margaret J. Hubley, Darin P. Clark, David T. Mandrell, and Keith C. Cheng. Comparative analysis of fixation and embedding techniques for optimized histological preparation of zebrafish. *Comparative Biochemistry and Physiology Part - C: Toxicology and Pharmacology*, 208(July 2017):38–46, 2018. ISSN 18781659. doi: 10.1016/j.cbpc.2017.11.003. URL `https://doi.org/10.1016/j.cbpc.2017.11.003`.

[2] Joseph A Holden, Lester L Layfield, and Jennifer L Matthews. *The zebrafish: atlas of macroscopic and microscopic anatomy*. Cambridge University Press, 2013.

[3] Jinxing Lin, Qiusheng Chen, and Jianhua Hu. *Color atlas of zebrafish histology and cytology*. Springer Nature, 2022.

[4] David Grant Colburn Hildebrand, Marcelo Cicconet, Russel Miguel Torres, Woohyuk Choi, Tran Minh Quan, Jungmin Moon, Arthur Willis Wetzel, Andrew Scott Champion, Brett Jesse

Graham, Owen Randlett, George Scott Plummer, Ruben Portugues, Isaac Henry Bianco, Stephan Saalfeld, Alexander David Baden, Kunal Lillaney, Randal Burns, Joshua Tzvi Vogelstein, Alexander Franz Schier, Wei Chung Allen Lee, Won Ki Jeong, Jeff William Lichtman, and Florian Engert. Whole-brain serial-section electron microscopy in larval zebrafish. *Nature*, 545(7654):345–349, 2017. ISSN 14764687. doi: 10.1038/nature22356. URL `http://dx.doi.org/10.1038/nature22356`.

[5] B. Thisse, V. Heyer, A. Lux, V. Alunni, A. Degrave, I. Seiliez, J. Kirchner, JP. Parkhill, and C. Thisse. Spatial and temporal expression of the zebrafish genome by large-scale in situ hybridization screening. *Methods Cell Biol*, 2004. doi: 10.1016/s0091-679x(04)77027-2.

[6] Ulrike Von Luxburg. A tutorial on spectral clustering. *Statistics and Computing*, 17(4): 395–416, 2007. ISSN 09603174. doi: 10.1007/s11222-007-9033-z.

[7] NMD Niezink. *Consensus in networked multi-agent systems*. PhD thesis, Faculty of Science and Engineering, 2011.

[8] Mansour A Ballal and Paul A Sanford. Physiology of the sphincter of oddi-the present and the future?-part 1. *Saudi Journal of Gastroenterology*, 6(3):129–146, 2000.

[9] Erika Kague, Michael Gallagher, Sally Burke, Michael Parsons, Tamara Franz-Odendaal, and Shannon Fisher. Skeletogenic Fate of Zebrafish Cranial and Trunk Neural Crest. *PLoS ONE*, 7(11):1–13, 2012. ISSN 19326203. doi: 10.1371/journal.pone.0047394.

[10] Rodney M. Dale and Jacek Topczewski. Identification of an evolutionarily conserved regulatory element of the zebrafish col2a1a gene. *Developmental Biology*, 357(2):518–531, 2011. ISSN 1095564X. doi: 10.1016/j.ydbio.2011.06.020.

[11] F. Guilak, R. A. Zell, G. R. Erickson, D. A. Grande, C. T. Rubin, K. J. McLeod, and H. J. Donahue. Mechanically induced calcium waves in articular chondrocytes are inhibited by gadolinium and amiloride. *Journal of Orthopaedic Research*, 17(3):421–429, 1999. ISSN 07360266. doi: 10.1002/jor.1100170319.

[12] Joanna F. Weber and Stephen D. Waldman. Calcium signaling as a novel method to optimize the biosynthetic response of chondrocytes to dynamic mechanical loading. *Biomechanics and Modeling in Mechanobiology*, 13(6):1387–1397, 2014. ISSN 16177940. doi: 10.1007/s10237-014-0580-x.

[13] Ali Mobasheri, Csaba Matta, Ilona Uzielienè, Emma Budd, Pablo Martín-Vasallo, and Eiva Bernotiene. The chondrocyte channelome: A narrative review. *Joint Bone Spine*, 86(1): 29–35, 2019. ISSN 17787254. doi: 10.1016/j.jbspin.2018.01.012.

[14] Marc Duque, Alex B. Chen, Eric Hsu, Sujatha Narayan, Altyn Rymbek, Shahinoor Begum, Gesine Saher, Adam E. Cohen, David E. Olson, Yulong Li, David A. Prober, Dwight E. Bergles, Mark C. Fishman, Florian Engert, and Misha B. Ahrens. Ketamine induces plasticity in a norepinephrine-astroglial circuit to promote behavioral perseverance. *Neuron*, page S0896627324008365, December 2024. ISSN 08966273. doi: 10.1016/j.neuron.2024.11.011. URL https://linkinghub.elsevier.com/retrieve/pii/S0896627324008365.

[15] Bernice Grafstein, Shujun Liu, Maria Luisa Cotrina, Steven A Goldman, and Maiken Nedergaard. Meningeal cells can communicate with astrocytes by calcium signaling. *Annals of neurology*, 47(1):18–25, 2000.

[16] Payel Banerjee, Paul Joly, Luc Jouneau, Yan Jaszczyszyn, Mickaël Bourge, Pierre Affaticati, Jean Pierre Levraud, Pierre Boudinot, and Jean Stéphane Joly. Molecular and Cellular Analysis of the Repair of Zebrafish Optic Tectum Meninges following Laser Injury. *Cells*, 11 (13), 2022. ISSN 20734409. doi: 10.3390/cells11132016.

[17] Nicholas James Sofroniew, Daniel Flickinger, Jonathan King, and Karel Svoboda. A large field of view two-photon mesoscope with subcellular resolution for in vivo imaging. *eLife*, 5 (JUN2016):1–20, 2016. ISSN 2050084X. doi: 10.7554/eLife.14472.

[18] Oleg I. Rumyantsev, Jérôme A. Lecoq, Oscar Hernandez, Yanping Zhang, Joan Savall, Radosław Chrapkiewicz, Jane Li, Hongkui Zeng, Surya Ganguli, and Mark J. Schnitzer. Fundamental bounds on the fidelity of sensory cortical coding. *Nature*, 580(7801):100–105, April 2020. ISSN 0028-0836, 1476-4687. doi: 10.1038/s41586-020-2130-2. URL https://www.nature.com/articles/s41586-020-2130-2.

[19] Jeffrey Demas, Jason Manley, Frank Tejera, Kevin Barber, Hyewon Kim, Francisca Martínez Traub, Brandon Chen, and Alipasha Vaziri. High-speed, cortex-wide volumetric recording of neuroactivity at cellular resolution using light beads microscopy. *Nature methods*, 18 (9):1103–1111, September 2021. ISSN 1548-7091. doi: 10.1038/s41592-021-01239-8. URL https://pmc.ncbi.nlm.nih.gov/articles/PMC8958902/.

[20] Dal Hyung Kim, Jungsoo Kim, João C. Marques, Abhinav Grama, David G. C. Hildebrand, Wenchao Gu, Jennifer M. Li, and Drew N. Robson. Pan-neuronal calcium imaging with cellular resolution in freely swimming zebrafish. *Nature Methods*, 14(11):1107–1114, November 2017. ISSN 1548-7105. doi: 10.1038/nmeth.4429. URL https://www.nature.com/articles/nmeth.4429. Publisher: Nature Publishing Group.

[21] Fei Chen, Paul W Tillberg, and Edward S Boyden. Expansion microscopy. *Science*, 347 (6221):543–548, 2015.

[22] Jueun Sim, Chan E Park, In Cho, Kyeongbae Min, Minho Eom, Seungjae Han, Hyungju Jeon, Hyun-Ju Cho, Eun-Seo Cho, Ajeet Kumar, Yosep Chong, Jeong Seuk Kang, Kiryl D Piatkevich, Erica E Jung, Du-Seock Kang, Seok-Kyu Kwon, Jinhyun Kim, Ki-Jun Yoon, Jeong-Soo Lee, Edward S Boyden, Young-Gyu Yoon, Jae-Byum Chang, and John A Paulson. Nanoscale resolution imaging of the whole mouse embryos and larval zebrafish using expansion microscopy. *bioRxiv*, page 2021.05.18.443629, 2022. URL `https://www.biorxiv.org/content/10.1101/2021.05.18.443629v2%0Ahttps://www.biorxiv.org/content/10.1101/2021.05.18.443629v2.abstract`.

[23] Emmanuelle Steib, Rob Tetley, Romain F. Laine, Dominic P. Norris, Yanlan Mao, and Julien Vermot. TissUExM enables quantitative ultrastructural analysis in whole vertebrate embryos by expansion microscopy. *Cell Reports Methods*, 2(10):100311, 2022. ISSN 26672375. doi: 10.1016/j.crmeth.2022.100311. URL `https://doi.org/10.1016/j.crmeth.2022.100311`.
