# Supplementary material for: Imaging cellular activity across all organs reveals body-wide circuits
Source: Nature. 2026 Sep 9;658(8138):1237–47. doi: 10.1038/s41586-026-10979-6 (PMC13716824; doi:10.1038/s41586-026-10979-6)
Supplement: Supplementary file 2 [file 41586_2026_10979_MOESM2_ESM.pdf]

nature portfolio

Corresponding author(s): Virginia Ruetten, Misha Ahrens

Last updated by author(s): Apr 5, 2025

Reporting Summary

Nature Portfolio wishes to improve the reproducibility of the work that we publish. This form provides structure for consistency and transparency in reporting. For further information on Nature Portfolio policies, see our Editorial Policies and the Editorial Policy Checklist.

Statistics

For all statistical analyses, confirm that the following items are present in the figure legend, table legend, main text, or Methods section.

n/a	Confirmed	
☐	☒	The exact sample size (*n*) for each experimental group/condition, given as a discrete number and unit of measurement
☐	☒	A statement on whether measurements were taken from distinct samples or whether the same sample was measured repeatedly
☐	☒	The statistical test(s) used AND whether they are one- or two-sided<br>*Only common tests should be described solely by name; describe more complex techniques in the Methods section.*
☐	☒	A description of all covariates tested
☐	☒	A description of any assumptions or corrections, such as tests of normality and adjustment for multiple comparisons
☐	☒	A full description of the statistical parameters including central tendency (e.g. means) or other basic estimates (e.g. regression coefficient) AND variation (e.g. standard deviation) or associated estimates of uncertainty (e.g. confidence intervals)
☐	☒	For null hypothesis testing, the test statistic (e.g. *F*, *t*, *r*) with confidence intervals, effect sizes, degrees of freedom and *P* value noted<br>*Give P values as exact values whenever suitable.*
☐	☒	For Bayesian analysis, information on the choice of priors and Markov chain Monte Carlo settings
☐	☒	For hierarchical and complex designs, identification of the appropriate level for tests and full reporting of outcomes
☒	☐	Estimates of effect sizes (e.g. Cohen's *d*, Pearson's *r*), indicating how they were calculated

Our web collection on statistics for biologists contains articles on many of the points above.

Software and code

Policy information about availability of computer code

Data collection	The following software was used for data collection: Elements v5 (Nikon).
Data analysis	The following software were used for analysis: Fiji software (v2.14.0), Python (v3.9), Matlab (R2024b). The custom code used can be found at: https:// github.com/mikarubi/voluseg/ and https://github.com/Weizheng96/WHOLISTIC-registration.

For manuscripts utilizing custom algorithms or software that are central to the research but not yet described in published literature, software must be made available to editors and reviewers. We strongly encourage code deposition in a community repository (e.g. GitHub). See the Nature Portfolio guidelines for submitting code & software for further information.

Data

Policy information about availability of data

All manuscripts must include a data availability statement. This statement should provide the following information, where applicable:

- Data availability
- Raw WHOLI 5 TIC data are available at https://s3.janelia.org/ahrens-lab/ruettenv/WHOLI 5 TIC/ and raw WB-ExM-IHC data at https:// s3.janelia.org/ahrens-lab/ruettenv/ExM/. The data can also be browsed on the accompanying website, https://wholistic.janelia.org/; an example WB-ExM-IHC dataset can be viewed at https://wholistic.janelia.org/data/exm-demo-dataset-vasculo-ventricular-system/. The plasmid map for Tg(ubb:jGCaMP8m) is available from Addgene (plasmid #232485).
- Code availability
- The 3D geometric body model is available at https://github.com/vruetten/wholebodymodel. Registration code is available in Python at https://github.com/vruetten/wholistic_registration and in MATLAB at https://github.com/Weizheng96/WHOLI 5 TIC-registration (https:// doi.org/10.5281/zenodo.21460338). 5 egmentation code is available at https://github.com/mikarubi/voluseg (https://doi.org/10.5281/ zenodo.21460398). The PhotoMap analysis pipeline is available at https://github.com/vruetten/PhotoMap (https://doi.org/10.5281/ zenodo.21460351).

Research involving human participants, their data, or biological material

Policy information about studies with human participants or human data. See also policy information about sex, gender (identity/presentation), and sexual orientation and race, ethnicity and racism.

Reporting on sex and gender	NA
Reporting on race, ethnicity, or other socially relevant groupings	NA
Population characteristics	NA
Recruitment	NA
Ethics oversight	NA

Note that full information on the approval of the study protocol must also be provided in the manuscript.

Field-specific reporting

Please select the one below that is the best fit for your research. If you are not sure, read the appropriate sections before making your selection.

☒ Life sciences ☐ Behavioural & social sciences ☐ Ecological, evolutionary & environmental sciences

For a reference copy of the document with all sections, see nature.com/documents/nr-reporting-summary-flat.pdf

Life sciences study design

All studies must disclose on these points even when the disclosure is negative.

Sample size	Sample sizes were determined based on previously published studies and preliminary experiments. No statistical method was explicitly used to predetermine sample sizes; however, our sample sizes align with standard practices in the field to achieve statistical significance.
Data exclusions	No data were excluded from analysis.
Replication	The results regarding hypoxia-induced mesenteric artery constriction were independently and successfully independently reproduced in another laboratory (N>5). The Whole-Body Expansion microscopy protocols were run by three different researchers, each of whom successfully obtained similar outcomes.
Randomization	Samples/organisms/participants were randomly assigned to experimental groups.
Blinding	*No blinding was used in either data collection or analysis. Blinding during data collection was not possible because the experimental condition determined the acquisition protocol and was therefore necessarily known to the experimenter at the microscope. Blinding during analysis was not applied because all reported quantities were extracted by automated pipelines using identical parameters across conditions.*

Reporting for specific materials, systems and methods

We require information from authors about some types of materials, experimental systems and methods used in many studies. Here, indicate whether each material, system or method listed is relevant to your study. If you are not sure if a list item applies to your research, read the appropriate section before selecting a response.

Materials & experimental systems

n/a	Involved in the study	
☐	☒	Antibodies
☒	☐	Eukaryotic cell lines
☒	☐	Palaeontology and archaeology
☐	☒	Animals and other organisms
☒	☐	Clinical data
☒	☐	Dual use research of concern
☒	☐	Plants

Methods

n/a	Involved in the study	
☒	☐	ChIP-seq
☒	☐	Flow cytometry
☒	☐	MRI-based neuroimaging

Antibodies used: rabbit anti-eGFP (Invitrogen, A11122),
chicken anti-RFP (synaptic systems, 409006),
goat anti-rabbit Atto647N (Sigma, 40839),
donkey anti-chicken Alexa568 (Invitrogen, A78950)

Animals and other research organisms

Policy information about studies involving animals; ARRIVE guidelines recommended for reporting animal research, and Sex and Gender in Research

Laboratory animals	Danio rerio (5-14 dpf), Danionella cerebrum (7dpf to 2 months old)
Wild animals	No wild animals were used in the study,
Reporting on sex	Zebrafish sex cannot be determined until ~4 weeks post-fertilization (Anderson et al. 2012}) so the sex of the experimental animals was unknown.<br>Danionella younger than 6 weeks old are sexually immature and could not be sexed; the sex of fish older than 6 weeks are reported in the main text.
Field-collected samples	NA
Ethics oversight	All animal procedures were approved by the Institutional Animal Care and Use Committee (IACUC) of the Howard Hughes Medical Institute, Janelia Research Campus and were conducted in accordance with the National Institutes of Health Guide for the Care and Use of Laboratory Animals.

Note that full information on the approval of the study protocol must also be provided in the manuscript.

Plants

Seed stocks	*Report on the source of all seed stocks or other plant material used. If applicable, state the seed stock centre and catalogue number. If plant specimens were collected from the field, describe the collection location, date and sampling procedures.*
Novel plant genotypes	*Describe the methods by which all novel plant genotypes were produced. This includes those generated by transgenic approaches, gene editing, chemical/radiation-based mutagenesis and hybridization. For transgenic lines, describe the transformation method, the number of independent lines analyzed and the generation upon which experiments were performed. For gene-edited lines, describe the editor used, the endogenous sequence targeted for editing, the targeting guide RNA sequence (if applicable) and how the editor was applied.*
Authentication	*Describe any authentication procedures for each seed stock used or novel genotype generated. Describe any experiments used to assess the effect of a mutation and, where applicable, how potential secondary effects (e.g. second site T-DNA insertions, mosiacism, off-target gene editing) were examined.*
