# Supplementary material for: Imaging cellular activity across all organs reveals body-wide circuits
Source: Nature. 2026 Sep 9;658(8138):1237–47. doi: 10.1038/s41586-026-10979-6 (PMC13716824; doi:10.1038/s41586-026-10979-6)
Supplement: Supplementary file 3 [file 41586_2026_10979_MOESM3_ESM.pdf]

nature portfolio

Peer Review File

Imaging cellular activity across all organs reveals body-wide circuits

Corresponding Author: Dr Misha Ahrens

Any redactions in this file are there to maintain patient confidentiality, the confidentiality of unpublished data, or to remove third-party material.

This file contains all reviewer reports in order by version, followed by all author rebuttals in order by version.

Attachments originally included by the reviewers as part of their assessment can be found at the end of this file.

Version 0:

Reviewer comments:

Referee #1

(Remarks to the Author)
In the manuscript by Ruetten et al., the authors developed a functional imaging method, WHOLISTIC, to record real-time cellular calcium dynamics across most regions of the body in live fish. This was achieved by combining cutting-edge techniques: volumetric fluorescence microscopy, machine learning and multiple genetic models. Using this system, they discovered novel cellular/organ/multi-organ scale responses such as cold-responding chondrocytes, traveling waves in the kidney nephron, ultraslow ependymal responses during periods without motor activity, and hindbrain-gut interactions. They also demonstrated causal relationships in some of their findings using optogenetic tools. Additionally, they established an advanced whole body expansion microscopy to understand ultrastructural anatomy from molecular/subcellular to organ levels. This advanced method revealed previously undescribed anatomical organizations, and some of the results support their functional imaging data in this manuscript.
Overall, the manuscript is well written, and most figures and videos are well organized, making the content accessible to readers. The findings are fascinating and of broad interest. More importantly, the innovative methods presented could become game changers, advancing the system biology as well as general biomedical research by enabling whole-organism analysis from cellular to systems-level resolutions in both health and diseases. This manuscript pushes the boundaries of systems physiology and deserves to be published in a prestigious journal.

Below are my specific comments on the manuscript.

1.The authors use a ubiquitin promoter to drive the expression of GCamps across all cells of the body. They use the tTA system in zebrafish and a direct promoter in Danionella. Even though these promoters are expressed relatively ubiquitously, their expression is not uniform across tissues and developmental stages. Also observing calcium oscillations will require the calcium sensor levels to be in a dynamic range. I suggest that the authors discuss in more detail these limitations and whether they observed correlations between GCamp6s expression and cellular activity and whether low activity in some cells may depend on oversaturation of signal. As nicely described in the introduction, while calcium dynamics have been very well characterized in the brain, we know little about calcium dynamics in other tissues. Hence, these findings will be important for future work using calcium sensors in less studied cell types.
2.Most of the work described on brain-body interaction in this manuscript has been done in zebrafish. Very few experiments were performed in Danionella. Experiments in Danionella show the feasibility of the experimental strategy rather than providing new insights. I do understand the value of showing that the techniques could be applied in other species, like Danionella, and I do not see the need for the authors to reproduce their zebrafish findings in Danionella. However, at this point, the abstract should be rephrased to better represent what experiments were done with Danionella in this manuscript.
3.Sample preparation and normoxic/hypoxic conditions
- In the Methods section, the authors do not describe the use of a perfusion system to maintain optimal physiological temperature and oxygen levels in the E3 media. Additionally, the authors do not describe removing agarose near the mouth and gill, which could restrict gas exchange during functional imaging. These raise questions on the normoxia levels at baseline. If the authors did not use the perfusion system or another oxygenation system, it is likely that the fish experienced

some level of hypoxia during imaging, especially given that the fish were embedded in agarose for 1-2 hours. I recommend that the authors better clarify their methodology in the text and in the material and methods section.
- In relation to the measurement of oxygen levels, it is unclear to me where the oxygen levels were measured. Providing the oxygen levels in the samples (in agarose) rather than in the air chamber may better represent the environment sensed by the animals. This should be further clarified in the text.
- Hypoxia may alter the pH environment experienced by the fish. Can the authors clarify whether this is something they have taken into consideration?
4. In line 130: The term “meningeal cells” may require further validations, since the meningeal layers are thin and difficult to distinguish purely by the volumetric imaging. The authors should consider rephrasing the statement or perform further validations with specific markers of meninges.
5. In line 130: The authors identified cold responding chondrocytes when adding cold water manually, as described in the Methods. To avoid the issue that mechanical forces or another side effects occurs during the manual handling and activates chondrocytes, the authors may want to include a negative control experiment where they deliver water at normal temperature using a similar strategy.
6. Line 262: it is unclear to me whether the authors measure contraction of the gastric sphincter or calcium activity in the cells of the gastric sphincter. This should be clarified.
7. Line 348 and Fig3j, there is a line at the middle of the heatmap. The authors should clarify whether this is the result of bodily movement or of something else.
8. Line 387-388: the authors mentioned there is no major difference in the brain and gill during exposure to hypoxia, but I could not find the actual data. The authors should provide the data to support these claims.
9. Although WHOLISTIC is a powerful method for whole-body imaging of animals, all functional imaging was performed on laterally mounted fish immobilized by 2% agarose, which is not a natural posture of zebrafish. Thus, it might be good to add this limitation in the discussion section and mention that it can be improved in future studies.
10. Line 584: I read that the Danionella samples were imaged in artificial cerebrospinal fluid. This appears surprising because it is quite different from the water the animals live in. The authors will want to clarify why they used such buffer.
11. Data sharing: The authors described in the manuscript that they will be sharing the raw data upon publication. I recommend that the authors provide user friendly data pipelines and robust metadata information, so that the data is useable and useful to the scientific community. These datasets will be extremely valuable and could open up many possibilities to labs that have or do not have the facilities to perform such experiments. The authors designed a webpage describing their findings. The webpage is very well documented and gives good information about the project in general. This webpage could serve as the gateway to the datasets.

Minor textual comments

1. At line 24, please spell the full name of WHOLISTIC first.
2. At line 31, please change “all cells” to “most cells” as it looks the entire body was not imaged (e.g. missing the tail part in the dataset) and some cells may have been missed.
3. Line 78: The subtitle might need to be changed, such as “WHOLISTIC captures cellular calcium activity across organ systems” since they only measured calcium activities.
4. Line 289: It might be better to replace “regular swimming” to “movement” since the imaged fish were immobilized in agarose.

(Remarks on code availability)
We have not been able to do this due to limited time.

Referee #2

(Remarks to the Author)
I co-reviewed this manuscript with one of the reviewers who provided the listed reports.

(Remarks on code availability)

Referee #3

(Remarks to the Author)
The manuscript presents a method aiming at enabling recording calcium dynamics across the entire body in transparent vertebrates. By engineering a transgenic pancellular fish line expressing calcium indicators in nearly all cells across different organs, and combining it with optimized imaging and computational tools, the study demonstrates how coordinated

cellular dynamics across organs can be visualized and analyzed. This approach offers valuable insights into the connection between cellular and organismal physiology, potentially advancing our understanding of complex behaviors and diseases. Some issues should be addressed, below:

Major Comments

1. Calcium is imaged, largely as a matter of convenience. But what is the physiological meaning of calcium in these different cells, tissues, and organs? It would be good to know what calcium signifies in these different locations. Probably it is too much to ask for further experiments along these lines, but perhaps a table reviewing the relevant literature would be helpful, explaining what intracellular calcium signals represent in different cells, and what it is indicating regarding biological dynamics.
2. The manuscript claims “all cells in the body” and other similar claims, throughout. But it is unclear whether all cell types are truly represented – are some organs, like heart, lacking expression? What fraction of cells in each organ is expressing the indicator, and can be imaged? Most of the studies presented seem to focus on a small number of organs - did any experiment truly require analysis of collective or correlated calcium dynamics across all organs in the entire fish? If not, perhaps some of the claims should be toned down.
3. The authors present a novel whole-organism expansion microscopy method (WB-ExM) different from other recent protocol, but it is unclear whether they have validated resolution and distortion in the ways that most novel expansion microscopy method are presented. The authors should characterize the isotropy of the expanded tissue for their method, particularly since the method iterates the process many times, an unusual process.

Minor Comments

1. The authors have demonstrated a cell segmentation method based on spontaneous calcium dynamics and spectral clustering. But does it work in all organ systems? It seems that validation through traditional imaging is inconsistent throughout the paper. It is also concerning that there could be crosstalk from neighboring cells, especially in one-photon imaging, resulting in artifactual correlation of neural activities. Could calcium dynamics across different cell types lead to contamination of signals, impeding the accuracy of spectral clustering relying on spontaneous activities? More validation experiments would clarify and support the proposed segmentation method.
2. Calcium dynamics can be very fast. The use of a spinning disk confocal microscope for imaging at a volumetric rate of 0.3 Hz is well below such speeds in the literature. The slow volumetric rate might lead to missing of much calcium activity, and thus omitted signals or organs. Re-imaging subsets at higher speeds could validate the claims of the paper, and make sure that there are not higher frequency signals that are missing.
3. In Fig. 2 f-g, a tissue-specific kernel was applied for the regression model. The selection process and criteria for those kernels could be better described.
4. The use of various fish lines is confusing to read. Adding a table to list the names of the lines and where they are used would be helpful.
5. In Figure 2n, a color map is used to show the different traces, corresponding to a range of optogenetic stimulation intensities; however, the scale of the stimulation and how it maps to the colors is not provided in the figure.

(Remarks on code availability)

Reviewing the code is beyond our capability, and expertise, alas.

Referee #4

(Remarks to the Author)

The paper by Ruetten et al. images calcium dynamics across the whole body of larval zebrafish, using an approach the authors call WHOLISTIC. The study is impresive in its scope and the number of techniques the authors present:

- Imaging calcium dynamics not only in the brain, but in many other tissues too.
- Improved expansion microscopy protocols.
- Improved analysis of spatiotemporal calcium signals (coherence-based spectral clustering).
- A slightly novel motion correction algorithm.

None of these are innovations per-se, although together they do form a very interesting method and approach that is indeed novel.

In fact, that is my main concern with the paper, that it seems to be more of a methods paper. It is difficult for me to state the key results, beyond the method. As the authors themselves state:

"Collectively, these findings demonstrate how the ubiquitous expression of molecular sensors, in conjunction with advanced motion-corrected spinning-disk confocal microscopy and the computational workflow for cellular and tissue segmentation, together comprising WHOLISTIC, give access to a rich array of cellular dynamics across tissues of the body, thereby facilitating the study of their interactions."

and they refer to WHOLISTIC as a methodology (line 465). As the abstract states: the authors "advance and integrate" already existing methods and I am not sure what the main scientific result of the study is.

If my understanding is correct, fish are mounted on the side (unlike what is shown in Fig 1a), and a field of view of 800 x 600 x 130 microns is recorded shown in Figure 1e. It is important to highlight that this is the field of view, and not Fig 1b or 1c. Then confocal microscopy is used at a rate of 0.3 Hz. In terms of imaging this is not groundbreaking: the Ahrens Lab has outdone this by an order of magnitude. There are many reasons why confocal microscopy is not used typically in in-vivo

studies and in this instance the whole tissue will be constantly irradiated with excitation light.

Personally, if this was to be viewed as a methods paper, I would find it more useful if the improvements mentioned above were more carefully quantified. How much better is the motion correction methods used compared to current methods? How does the coherence-based spectral clustering compare to correlation based clustering? Can the authors plot for example a measure of cluster/eigenmode size as a function of cluster/eigenmode number to backup their argument about compactness of the spatial ROIs of the first method vs the second? Currently, the data presented is a very particular example shown in Ext. Data Fig 4 d-f and there is no quantification of the method in general. Similarly, for the statements made in Ext. Data Fig 4 a-c regarding interpretability of the clusters: the authors themselves mention that the results depende on the number of clusters/eigenmodes, so the reader is left wondering how much better are these methods really. I do not doubt that they are, I do think though that this has to be unequivocally shown, in order to truly appreciate the improvements presented by WHOLISTIC. This is a general point: the methods are reasonably well presented, but they are not appropriately benchmarked with the existing state-of-the-art approaches.

There are a number of minor concerns that I will list below in case they are helpful to the authors (I am sure there are many others, but I think a general restructuring of the manuscript to either present it as a scientific study or a methods study should happen first).

- Line 143: I do not support refering to the calcium dynamics as 'cellular activity'. For neurons this is already established, but there are many things that could constitute activity in general in cells, and as the authors themselves present in the discussion, many other reporters could be used. I strongly encourage the change to 'Ca activity' or 'calcium activity'. Even though this may be slightly more tedious, I believe it is scientifically more appropriate.

- In going from equation (26) to equation (27), the sum in the first term disappears and becomes 1. Should this be an n?

- Line 1068: does psd mean positive semi-definite?

- Could the authors provide full stacks of their new generated transgenic lines?

Overall, I believe that the amount of work that has gone into this study is astounding and I congratulate the authors on it, even if it leaves me slightly confused as to what the message is that I should be taking home.

(Remarks on code availability)

Version 1:

Reviewer comments:

Referee #1

(Remarks to the Author)
All my comments are properly addressed by the authors. I want to congratulate the authors for this extensive and impressive work. I support publication of this manuscript.

(Remarks on code availability)

Referee #2

(Remarks to the Author)
I co-reviewed this manuscript with one of the reviewers who provided the listed reports.

(Remarks on code availability)

Referee #3

(Remarks to the Author)
The authors have addressed my concerns and the paper is acceptable for publication.

(Remarks on code availability)

Referee #4

(Remarks to the Author)
The authors have taken the reviewers' comments seriously and have done a good job of incorporating feedback. Overall, the manuscript is very impressive. Whilst I would have liked to see more of an emphasis on biological findings, the study is faultless and presents a method that will prove to be influential.

(Remarks on code availability)

Referee #5

(Remarks to the Author)
This study developed the WHOLISTIC technique, which aims to image calcium activity in real time across most cells in living zebrafish embryos. The research revealed multiple cross-organ coordination phenomena, including pulsatile traveling waves in the proximal tubule of the nephron, ultraslow oscillations in ependymal cells during quiescence, rapid responses of chondrocytes to cold stimulation, and brainstem-controlled redistribution of blood flow under hypoxic conditions. The image processing method presented here is significant for capturing rapid signaling dynamics in living organisms, though it also has certain limitations.

1. Figure 1h shows the proximal tubule of the zebrafish embryonic pronephros, not the entire nephron. This should be corrected by the authors.

2. The glomerulus is located below the proximal tubule shown in Fig. 1h, not at the position indicated by "G" in the figure. This raises an important technical question: can the method described by the authors adequately resolve deep organs and tissues? What is the maximum imaging depth that this method can simultaneously achieve? If the thickness that can be acquired in a single volume is limited, is the method still sufficient to simultaneously resolve most tissues and organs of the zebrafish embryo?

3. Furthermore, many tissues are composed of thin layers of tightly adhered cells, such as vascular endothelial cells and pericytes, as well as glomerular vascular endothelial cells, podocytes, and mesangial cells. In these tissues, individual cell types often require specific transgenic lines to be distinguished. This raises another question: is the method in this study sufficient to resolve tightly adhered, thin-layered cellular structures?

4. Using higher temporal resolution, this study identified pulsatile traveling waves in the proximal tubule. The proximal tubule can be segmented into continuous functional ensembles, each exhibiting distinct dynamic characteristics. This is a very interesting finding that provides an important tool for studying renal tubule function. The authors suggest that this indicates kidney filtration may in fact be intermittent and pulsatile on shorter timescales. This speculation may be debatable, because this part only labels the proximal tubule, not the glomerulus, which is responsible for filtration. These pulsatile traveling waves might instead be related to proximal tubule epithelial cells driving the flow of intraluminal fluid. Of course, all these speculations require further validation through separate, dedicated studies.

5. Neuromodulation itself is a well-recognized form of inter-organ communication. Therefore, it might be beneficial to include additional examples of inter-organ coordination that go beyond neural or systemic stress-related stimuli.

6. There is a minor data writing issue: two figure legends state $p = 0.3125$, whereas the corresponding figure shows $p = 0.03125$, which appears to be a writing error. Additionally, the figure legend does not indicate what the asterisk (*) represents.

(Remarks on code availability)

Version 2:

Reviewer comments:

Referee #5

(Remarks to the Author)
Although the authors did not fully answer all the questions, I understand that this was mainly due to limitations of the model and existing technologies. Therefore, I have no further questions. In addition, I would like to congratulate the authors on their technical breakthrough!

(Remarks on code availability)

Open Access This Peer Review File is licensed under a Creative Commons Attribution 4.0 International License, which permits use, sharing, adaptation, distribution and reproduction in any medium or format, as long as you give appropriate credit to the original author(s) and the source, provide a link to the Creative Commons license, and indicate if changes were made.
In cases where reviewers are anonymous, credit should be given to 'Anonymous Referee' and the source.
The images or other third party material in this Peer Review File are included in the article's Creative Commons license, unless indicated otherwise in a credit line to the material. If material is not included in the article's Creative Commons license and your intended use is not permitted by statutory regulation or exceeds the permitted use, you will need to obtain permission directly from the copyright holder.
To view a copy of this license, visit https://creativecommons.org/licenses/by/4.0/

Response to Reviewers' Comments

We thank the Reviewers and the Editor for their careful assessment of the manuscript and for the insightful and constructive comments. Below, we address all comments in blue text, and corresponding changes in the revised manuscript are also indicated in blue.

Reviewer #1 (Remarks to the Author)

Reviewer Comment:

In the manuscript by Ruetten et al., the authors developed a functional imaging method, WHOLISTIC, to record real-time cellular calcium dynamics across most regions of the body in live fish. This was achieved by combining cutting-edge techniques: volumetric fluorescence microscopy, machine learning and multiple genetic models. Using this system, they discovered novel cellular/organ/multi-organ scale responses such as cold-responding chondrocytes, traveling waves in the kidney nephron, ultraslow ependymal responses during periods without motor activity, and hindbrain-gut interactions. They also demonstrated causal relationships in some of their findings using optogenetic tools. Additionally, they established an advanced whole-body expansion microscopy to understand ultrastructural anatomy from molecular/subcellular to organ levels. This advanced method revealed previously undescribed anatomical organizations, and some of the results support their functional imaging data in this manuscript. Overall, the manuscript is well written, and most figures and videos are well organized, making the content accessible to readers. The findings are fascinating and of broad interest. More importantly, the innovative methods presented could become game changers, advancing the system biology as well as general biomedical research by enabling whole-organism analysis from cellular to systems-level resolutions in both health and diseases. This manuscript pushes the boundaries of systems physiology and deserves to be published in a prestigious journal.

Below are my specific comments on the manuscript.

Our Response:

We thank Reviewer #1 for their positive assessment of our work and for their comments, which have helped us to improve the manuscript. We address each specific comment below.

Reviewer Comment:

The authors use a ubiquitin promoter to drive the expression of GCaMPs across all cells of the body. They use the tTA system in zebrafish and a direct promoter in Danionella. Even though these promoters

are expressed relatively ubiquitously, their expression is not uniform across tissues and developmental stages. Also observing calcium oscillations will require the calcium sensor levels to be in a dynamic range. I suggest that the authors discuss in more detail these limitations and whether they observed correlations between GCamp6s expression and cellular activity and whether low activity in some cells may depend on oversaturation of signal. As nicely described in the introduction, while calcium dynamics have been very well characterized in the brain, we know little about calcium dynamics in other tissues. Hence, these findings will be important for future work using calcium sensors in less studied cell types.

Our Response:

We thank the reviewer for these thoughtful comments. The concern about the dynamic range of the calcium sensor was also on our mind, since the calcium levels inside cells could fall outside, either below or above, the dynamic range of the sensor, which has sigmoid-like sensitivity. To evaluate this, we considered the standard deviation (σ) of cellular calcium signals, normalized by the cell's baseline brightness (b_0). If calcium levels are outside the sensitive range, one would expect the cell to have a low σ/b_0. We have added this data to Extended Data Figure 13, and we did not observe a systematic trend, with cells exhibiting a broad spread in σ/b_0 across a range of baseline brightness. We note that cells with the very highest baseline (top 1%) levels did not tend to have particularly high σ/b_0, however, these were not below average (dataset mean of σ/b_0 : 0.172, mean of cells with top 1% baseline: 0.173), suggesting that saturation in the low- or high-range is not a limiting factor for most cells. Nevertheless, we agree this could still be a concern for some cells, in part because the calcium buffering properties may differ across cell types. A discussion of this is in the Future Extensions section included in the Supplementary Notes, addressing the fact that the sensor dynamic range needs to match that of cell types and that fluorescence lifetime imaging would be a means of quantitatively assessing mismatches (line 2038):

> *"Furthermore, GCaMP has primarily been optimized for imaging neuronal calcium dynamics, and the dynamic range of the sensor might not be optimal for all other cell types. Whilst we did not observe saturation of variance in cells with high baseline levels (Extended Data Figure 13c), this does not rule out sensor saturation in certain cell types. Quantitative calcium measurements using fluorescence life-time imaging would be the definitive way of assessing the dynamic range of calcium concentrations across cells of the body."*

.

Below is the relevant figure panel (from page 87):

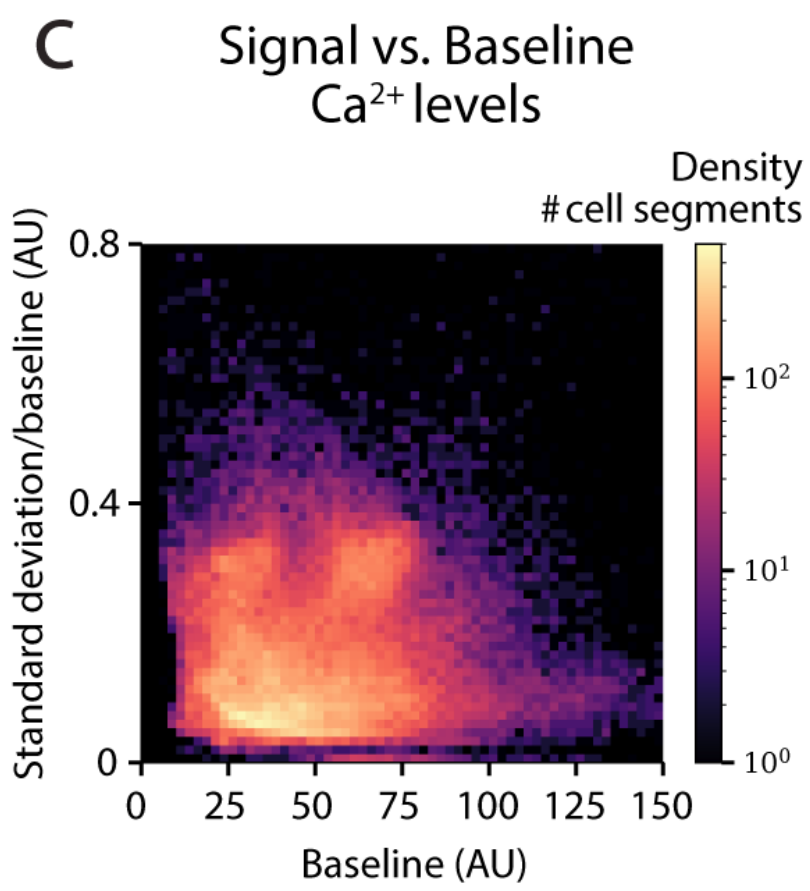

Extended Data Fig. 13: Characterization of GCaMP signal statistics.
c, Baseline normalized standard deviation versus baseline brightness plotted for the dataset presented in Figure 1l. Normalized standard deviation is not systematically lower in cells with very low or very high baseline brightness, suggesting that in the majority of the cells, the sensor does not appear to be under or over saturated.

Regarding expression over development, we agree that this would be interesting to characterize. As development was not the focus of the work we did not explore how expression might change over the lifetime of the animal, in part because most zebrafish studies are done when the animals are transparent ($\sim$ 10 days post fertilization). We did image samples samples at both 7 and 14 days post fertilization and we did not observe any noticeable change in expression density or expression levels across organs (Extended Data Figure 1c).

Regarding uniformity of the expression, we have added an example of expression differences between founders (Extended Data Figure 12), and highlighted the importance of screening for dense expression (line 555):

> *"We note that due to the non-deterministic landing site of the transgene, founders have variation in expression and need to be carefully screened for dense expression (Extended Data Figure 12)."*

We also visualized GCaMP expression along with unbiased labeling of nuclei (via DAPI) and total protein stain to more readily assess expression density. This had to be done in fixed, non-expanded samples as DAPI doesn't penetrate the skin of intact animals and because expansion protocols induce a strong decrease in GCaMP fluorescence. We have added this data to Extended Data Figure 12 (presented below) along with examples of 1) regions well labeled, 2) regions that lacked GCaMP signal due to lack of cellular content (e.g., swim bladder), and 3) regions (minority) that had less consistent labeling (e.g., exocrine pancreas). We have uploaded the data to the website and described the protocol used to generate it. We segmented nuclei using Suite2p, however due to scattering of the fixed tissue,

this only worked well on more superficial tissue. Manually counting the fraction of positive cells as best as we could in one representative founder (by taking a random sample of nuclei and judging if these cells were positive in GCaMP), we found that in most tissues (muscles, gills, brain, gastrointestinal tract, gallbladder, notochord) 85-100% of the tissue was labeled, the kidney had intermediate labeling (75-90%). The one organ that had substantially less cells labeled for some founders was the exocrine pancreas, where the cells lining the tubules (ductal cells) were well labeled ($\sim$ 85-100%) but the acinar cells were poorly labeled. These numbers vary slightly depending on founders, with for instance the exocrine pancreas being fully labeled in one of the two founders presented in Extended Data Figure 12a. Robust and systematic quantification of the fraction of labeled cells would be best done by generating a new transgenic line that expresses GCaMP as well as a nuclear-targeted fluorescent marker, or dissociating all cells, and FACS sorting them according to GCaMP signal (however this would lose spatial information). We have added a discussion on this in the Future Extensions section of the Supplementary Notes (line 2050).

> *"Finally, regarding transgenic coverage, while we qualitatively demonstrate broad pancellular expression, rigorous, automated and scalable quantification of the fraction of labeled cells proved technically challenging (Extended Data Fig. 12c). Future work would benefit from generating a new transgenic line co-expressing GCaMP with a spectrally distinct nuclear marker under the same promoter, enabling unambiguous, automated quantification of labeling density across all tissues."*

We thank the reviewer for this comment, which enabled us to perform the additional analysis and add clarity to the manuscript. Below, is the new Extended Data Figure addressing this comment:

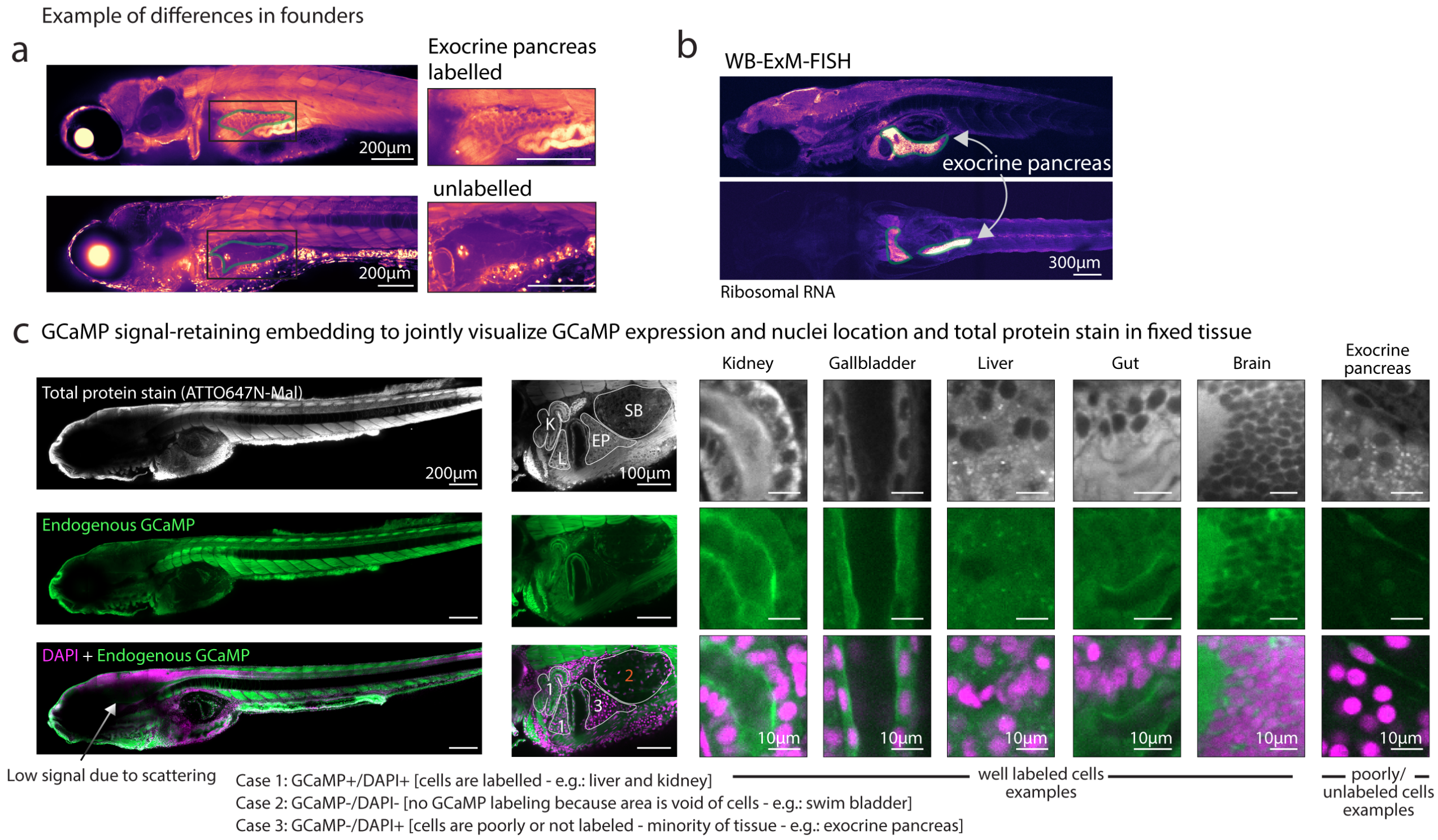

Extended Data Fig. 12: **Example of differences in pancellular founders and joint visualization of GCaMP signal along with nuclei in fixed tissue**
a, Transgenic lines generated via Tol2 transgenesis have non-determinisitc landing sites and are therefore sensitive to positional effects. Consequently, founders exhibit variable expression patterns and must be carefully screened for dense, uniform expression. Shown are offspring from two founders in which the exocrine pancreas is (*top*) or is not (*bottom*) labeled.
b, Sagittal (*top*) and ventral (*bottom*) views of a whole-body expansion microscopy (WB-ExM) sample (7 days post-fertilization) stained for ribosomal RNA, highlighting dense labeling in the exocrine pancreas.
c, GCaMP signal-retaining gel embedding for assessing expression density. Determining which cells express GCaMP requires unbiased localization of all cell bodies, achievable through nuclear labeling. Expansion protocols induce a drop in fluorescence signal and so could not be used. For nuclear DAPI staining, samples need to be fixed which results in more tissue scattering compared to live samples. We developed an embedding method that preserves endogenous GCaMP signal while still providing decent optical access throughout the tissue; we note that the quality of live-imaging or ExM data is still superior to this. *Left*: Example sample showing total-protein stain (ATTO647N-Maleimide, *top*), preserved endogenous GCaMP (*middle*), and DAPI (*bottom*). *Right*: Enlarged views of various tissues (kidney, gallbladder, liver, exocrine pancreas, intestine, brain) illustrating three cases: (1) cells positive for both GCaMP and DAPI; (2) regions lacking both GCaMP and DAPI, corresponding to acellular structures such as the swim bladder and notochord; (3) DAPI-positive nuclei with little, to no surrounding GCaMP signal, indicating poorly labeled cells (e.g., exocrine pancreas in this founder). In general, we found the vast majority of cells to be well labeled.

Reviewer Comment:

2. Most of the work described on brain-body interaction in this manuscript has been done in zebrafish. Very few experiments were performed in Danionella. Experiments in Danionella show the feasibility of the experimental strategy rather than providing new insights. I do understand the value of showing that the techniques could be applied in other species, like Danionella, and I do not see the need for the authors to reproduce their zebrafish findings in Danionella. However, at this point, the abstract should be rephrased to better represent what experiments were done with Danionella in this manuscript.

Our Response:

Thank you for this suggestion. We agree and have revised the abstract to more accurately reflect the extent of the experiments performed in Danionella and their primary role in demonstrating methodological applicability. The relevant parts of the abstract are now phrased as follows (line 26):

> *"By advancing and integrating volumetric fluorescence microscopy, machine learning, and pancellular transgenic expression of calcium sensors in transparent young Danio rerio (zebrafish) and with proof of concept in adult Danionella, the method enables real-time recording of cellular dynamics across the organism."*

Reviewer Comment:

3. Sample preparation and normoxic/hypoxic conditions

   - In the Methods section, the authors do not describe the use of a perfusion system to maintain optimal physiological temperature and oxygen levels in the E3 media. Additionally, the authors do not describe removing agarose near the mouth and gill, which could restrict gas exchange during functional imaging. These raise questions on the normoxia levels at baseline. If the authors did not use the perfusion system or another oxygenation system, it is likely that the fish experienced some level of hypoxia during imaging, especially given that the fish were embedded in agarose for 1-2 hours. I recommend that the authors better clarify their methodology in the text and in the material and methods section.
   - In relation to the measurement of oxygen levels, it is unclear to me where the oxygen levels were measured. Providing the oxygen levels in the samples (in agarose) rather than in the air chamber may better represent the environment sensed by the animals. This should be further clarified in the text.
   - Hypoxia may alter the pH environment experienced by the fish. Can the authors clarify whether this is something they have taken into consideration?

Our Response:

We thank the reviewer for pointing out these potential issues and address these critical points regarding sample preparation and physiological conditions as follows:

Perfusion and Agarose: Fish were imaged at 7 days post fertilization. At this age, cutaneous respiration is considered to be able to meet the oxygen needs of young zebrafish[1]. Given that oxygen readily diffuses through low-concentration agarose gels, it is therefore standard practice in the field to image the fish fully embedded in agarose without removing agarose near the mouth and gills at

this age[2,3]. However, we agree with the reviewer that this does not rule out that levels of oxygen may be slightly lower than if were no agarose at all (although this might not be abnormal for fish since oxygen in water can vary substantially, e.g. if water is stagnant or contains decaying organic material[4]). We therefore considered our assay to be one in which the *changes* in oxygen levels are what is most important. For brevity, we still refer to the baseline condition as 'normoxia' but we now clarify that this refers to the water — and that inside the fish, oxygen levels may be slightly lower than in the free-swimming condition due to the embedding; we have added the following paragraph in the Methods section providing background as to the methodology including references (line 638):

> *"We further note that even if the agarose is 98% water, and commonly used in the field[2,3], given that water is not flowing, this might still result in oxygen levels that are slightly lower than if there was no agarose, and if the animals were freely swimming. We therefore consider our assay to be one in which the change in oxygen levels are what is most important."*

Oxygen Measurement Location: Previously, oxygen levels were measured in the air above the water of the imaging chamber, and the partial oxygen pressure in air equilibrates with that in water fairly quickly because the volume of water is shallow and has a large surface area. We followed the reviewer's suggestion and have now measured oxygen levels in the water-submerged agarose as well. These are indeed consistent with the values in air, and now also provide the time course of equilibration (a few minutes), shown in Figure 4a. Below are the added comments in the Methods section (line 632), and the updated figure panel:

> *"Oxygen levels were measured in the bath using an oxygen micro-optode (Unisense, O_2 MicroOptode) inserted in the agarose Figure 4a. We fit an mono-exponential model to the data using the scipy.optimize.curve_fit function. The model accounts for 86% of the variance with a time constant of ~1.2 per minute."*

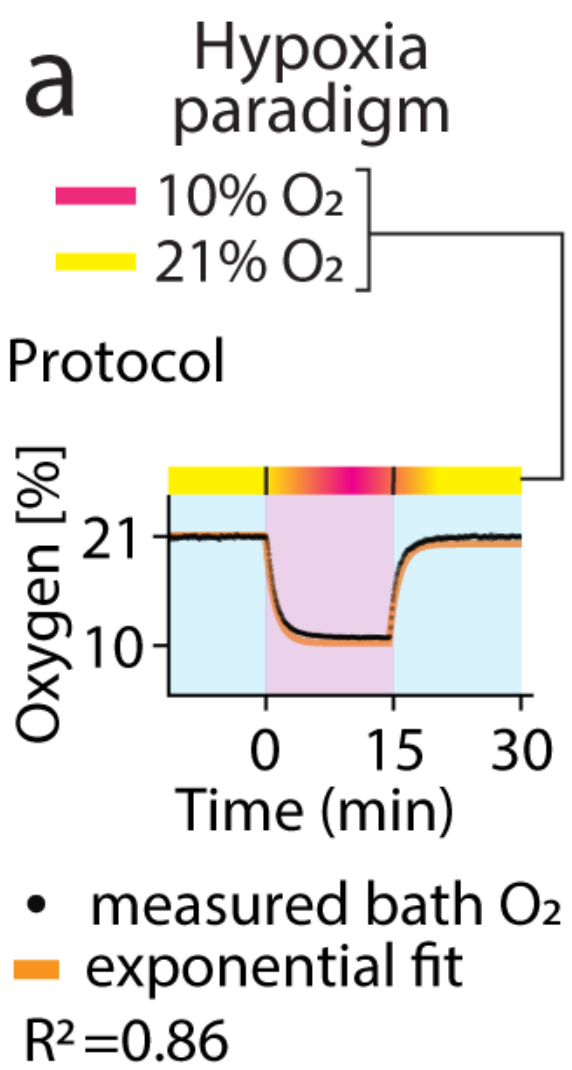

Fig. 4: **Uncovering of body-wide circuit engaged in response to stress.**
a, Hypoxia paradigm. Animals were subjected to cyclic oxygen levels between normoxia (21% O_2) and hypoxia (10% O_2). Black line: measurement of oxygen levels within the water-submerged agarose. The measured O_2 levels approximate exponential kinetics ($\tau \approx 1.2 min$), as expected from a two-compartment diffusive process.

pH Considerations: Regarding if the fish experience changes in pH, it is indeed true that hypoxia could change intracellular pH and, indirectly, possibly blood pH, although pH changes in the water in the chamber, directly caused by changes in water oxygenation would not be expected. We indeed think intracellular pH changes would likely occur[5]. The aim of the experiment was to assess the effects of hypoxia as would be experienced in waters of lowered oxygen content that fish naturally encounter, which would, as noted, cause changes in intracellular pH. As such, we did not aim to control this variable independently, as it is a downstream biological consequence. We have added a comment in the Methods section noting that the hypoxia assay is expected to cause changes in intracellular pH, among the other physiological responses to hypoxia (line 635):

> *"We note that hypoxia is expected to cause changes in acid-base balance within cells[5]. As the aim was to study the physiological consequences of hypoxia, we did not attempt to control tissue pH."*

Reviewer Comment:

4. In line 130: The term "meningeal cells" may require further validations, since the meningeal layers are thin and difficult to distinguish purely by the volumetric imaging. The authors should consider rephrasing the statement or perform further validations with specific markers of meninges.

Our Response:

Point 4 Response: We agree with the reviewer of the need for further validation that the 'brain-border' population corresponds to the meninges. Ideally, there would be a gene marker that labels all meningeal cells and no other cells in the brain. We reviewed the zebrafish literature and contacted the first author of the most comprehensive survey of the meninges in zebrafish[6] and talked to them and found that, unfortunately, to date, all markers only label a subset of cell types in the meninges, and all also label other cell types, which may make stainings difficult to interpret. We therefore attempted to first rule out that the identified 'brain-border' population was neuronal, or glial. For this, we co-imaged the 'brain-border' population, identified by their characteristic location, morphology and elevated baseline calcium levels when exposed to certain drugs (e.g., ketamine, as shown in Extended Data Figure 4), whilst also specifically labeling neurons and astrocytes using cell-type specific transgenic lines, and we could demonstrate that these do not co-localize. To gather further anatomical support, we reviewed the conserved organization of the meninges: one characteristic is that the meninges are separated from the brain parenchyma by the brain's basement membrane. In other words, the meninges (specifically its inner layer - pia in mammals) directly lies on top of the brain's basement membrane[7]. We thus reasoned that if the "brain-border" population lies on the outer surface of the brain's basement membrane, this would be further evidence this corresponds to the primitive meninges. We therefore co-imaged the "brain-border" population in conjunction with a transgenic line labeling basement membranes. We found that the basement membrane separated the brain parenchyma from the "brain-border" cells; in other words, the "brain-border" cells lay on the outer surface of the brain's basement membrane. Thus, we conclude that this population is indeed likely to be part of the primitive meningeal system of the brain. We have added these new experimental results to the Extended Data Figures (Extended Data Figure 4), that we have appended below:

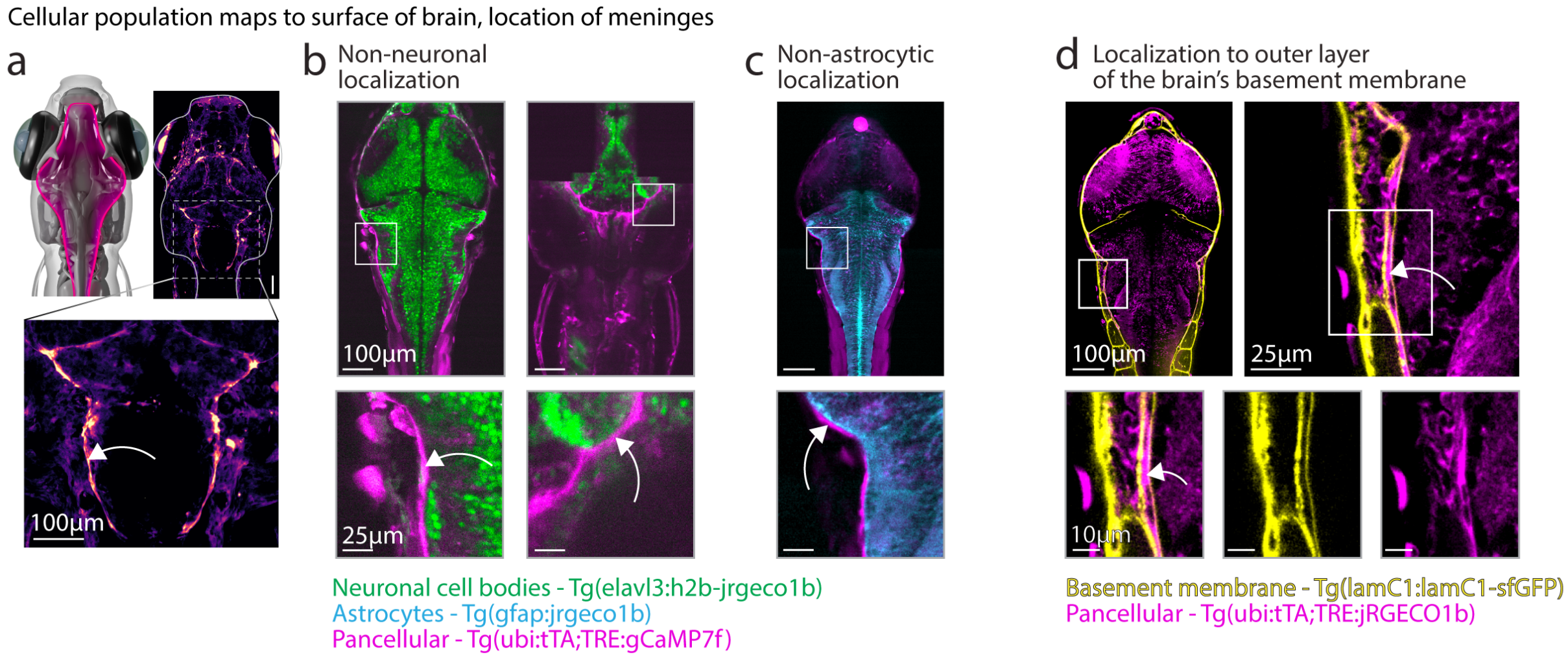

Extended Data Fig. 4: **WHOLISTIC screening for cellular responses to drugs.**
a, Maximum intensity projection showing a brightly fluorescent cellular layer surrounding the brain (top). Enlarged view of the hindbrain (bottom).
b, Border cells (visible among magenta cells) do not colocalize to neurons (green). Image of double transgenic line showing planes at the top (left) and bottom (right) of the brain (*Tg(elavl3:H2B-jRGECO1b; ubi:tTA;TRE:gCAMP7f)*) with inset showing enlarged view of border region. Contrast of magneta adjusted to highlight border cells.
c, Border cells (visible among magenta cells) do not colocalize to astrocytes (blue). Image of double transgenic line (*Tg(gfap:jRGECO1b; ubi:tTA;TRE:gCAMP7f)*) showing non-colocalization of border cells and astrocytes, with inset showing enlarged view of border region. Contrast of magenta channel adjusted to highlight border cells.
d, Border cells (visible among magenta cells) localize to the outer layer of the brain's basement membrane (yellow). Image of double transgenic line (*Tg(lamC1:lamC1-sfGFP; ubi:tTA;TRE:jREGCO1b)*) showing apposition of border cell and brain basement membrane, with inset showing enlarged view of border region. Contrast of magenta channel adjusted to highlight border cells.

Reviewer Comment:

5. In line 130: The authors identified cold responding chondrocytes when adding cold water manually, as described in the Methods. To avoid the issue that mechanical forces or another side effects occurs during the manual handling and activates chondrocytes, the authors may want to include a negative control experiment where they deliver water at normal temperature using a similar strategy.

Our Response:

This is an excellent suggestion. Indeed, chondrocytes have been reported to be mechanically sensitive. To avoid mechanical activation, we had delivered the water whilst trying to minimize disturbances. To confirm that the act of delivering water was not sufficient to explain the responses observed, we have performed the suggested negative control experiment: delivering water first at room temperature and then later water at reduced temperature. We found no significant response to our delivery procedure of the room temperature stimulus, whilst the cold water reliably evoked strong responses in chondrocytes. Thus, the observed responses are selective to cold and not due to sensing of fluid flow. We have added these new experimental results to the Extended Data Figures (Extended Data Figure 4), which we present below:

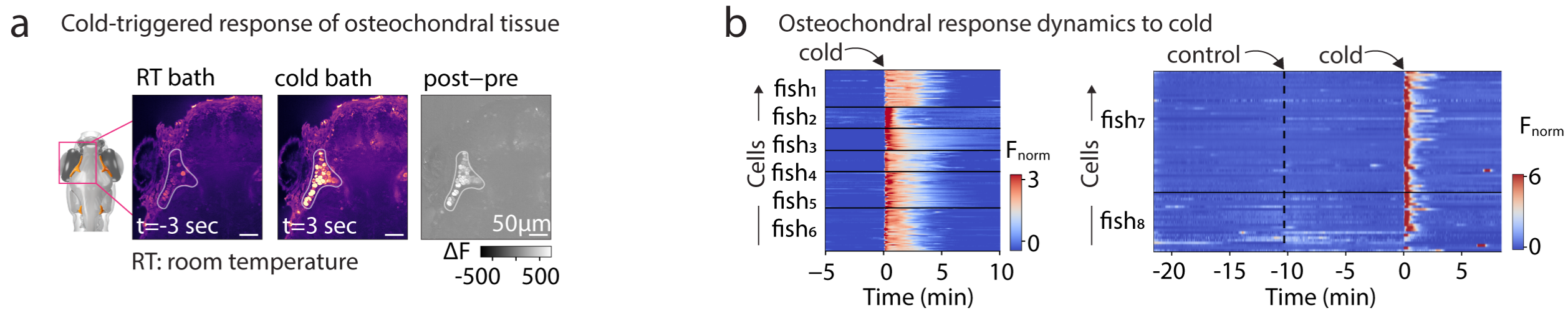

Extended Data Fig. 3: **WHOLISTIC screening for cellular responses to stimuli.**
a, WHOLISTIC screen for cold-responsive cells. Following a 5-minute period of baseline recording, 10°C water is introduced to the imaging chamber. Transverse section of the anterior portion of the cranium shown pre- and post-exposure to cold, and ΔF. Cold-responsive population outlined in gray.
b, Left: raster of Ca^{2+} levels in osteochondral cells aligned to cold stimulus onset, showing time-locked responses in 6 animals. Right: control assay - raster of Ca^{2+} levels in osteochondral cells showing little to no time-locked responses to control stimulus (room temperature water) in 2 animals and responses to cold stimulus.

Reviewer Comment:

6. Line 262: it is unclear to me whether the authors measure contraction of the gastric sphincter or calcium activity in the cells of the gastric sphincter. This should be clarified.

Our Response:

Thank you for pointing out this ambiguity. We have clarified in the text that we are measuring calcium activity in the cells of the gastric sphincter (Extended Data Figure 3) (line263):

> *"We found that activation of neurons within the most caudal segment of the motor vagus induces a dose-dependent calcium activation across the gastric sphincter (Fig. 13m-o), recapitulating the observed coupling identified by the model."*

Reviewer Comment:

7. Line 348 and Fig3j, there is a line at the middle of the heatmap. The authors should clarify whether this is the result of bodily movement or of something else.

Our Response:

We thank the reviewer for looking so carefully at the data. This was actually our attempt at showing an illustrative example which encompassed both the ultra-slow oscillations whilst showing that the motor-locked calcium responses are still sometimes present in their rare occurrence. We have added a trace of estimated motor activity (based on motion of auto-fluorescent pigments) which shows that there were two motor events during the recording, one coupled and one non-coupled to ependymal cell activity. As such the line near the middle of the heatmap isn't a motion artifact, but a calcium transient in

ependymal cells. (We also note that the transient lasts longer than a motion artifact would.) As Figure 2f-g shows, ependymal cells show strong calcium transients during bouts of muscle activity. During periods of prolonged quiescence, there is little motor activity but occasional single events do happen, as quantified in Fig. 3d. We have updated the text to make this clearer in the caption of Fig. 3 and thank the reviewer for their input:

> *"**j**, Top: Kymograph of ependymal cell activity along the hindbrain and spinal cord showing local traveling waves and occasional coupling to sparse motor activity over the course of ∼30 min. Data derived from cell type-specific transgenic (Tg(foxj1a:GCaMP7f)). Bottom: Normalized motor activity trace showing two motor events during the recording."*

As an aside, in revising the graph we noticed that we had made an error in our time axis (the axis and text were not consistent). We went back to the raw data to verify the sampling rate and have updated the timescale. The updated panels are shown below:

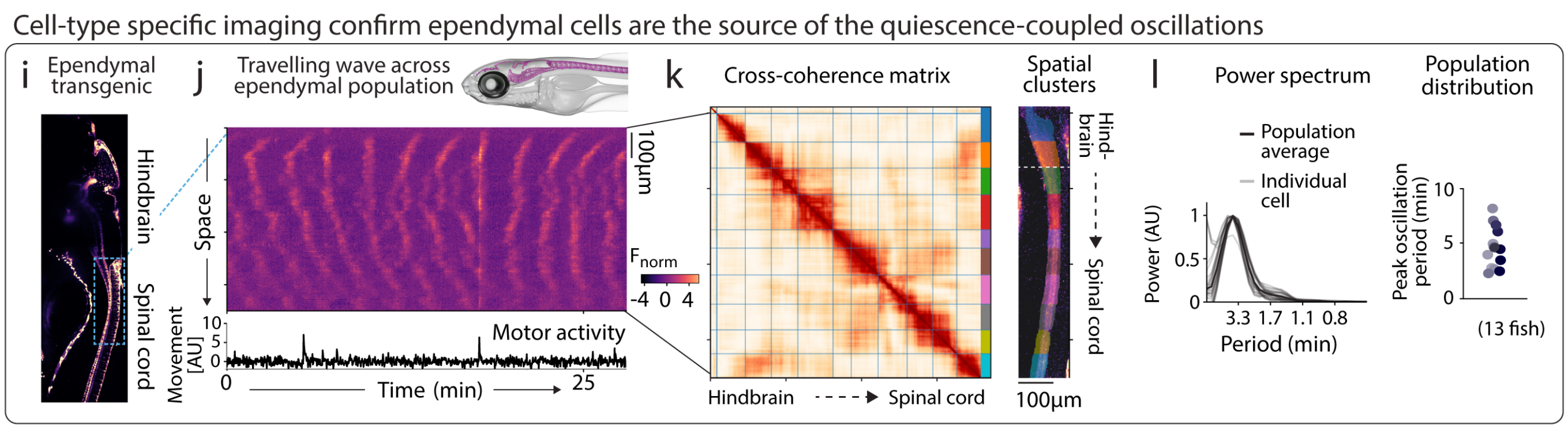

Fig. 3: **Discovery of motor-quiescence coupled ultraslow oscillations and cell-type of origin.**
i, Genetic strategy for imaging ependymal cells through the use of Foxj1a promoter, labeling all motile-ciliated cell including ependymal cells. Sagittal section of foxj1a-transgenic line (max. intensity projection).
j, *Top*: Kymograph of ependymal cell activity along the hindbrain and spinal cord showing local traveling waves and occasional coupling to sparse motor activity over the course of ∼30 min. Data derived from cell type-specific transgenic (*Tg(foxj1a:GCaMP7f)*). *Bottom*: Normalized motor activity trace showing two motor events during the recording.
k, Cross-coherence matrix derived from kymograph data in **j**, showing block-diagonal structure indicative of local coherence clusters. Coherence-based k-means clusters projected onto anatomical space, showing spatial continuity.
l, *Left*: Periodogram of ependymal cell activity. Population mean (dark) and individual cells (light) from example specimen. *Right*: population distribution of peak oscillatory frequency.

Reviewer Comment:

8. Line 387-388: the authors mentioned there is no major difference in the brain and gill during exposure to hypoxia, but I could not find the actual data. The authors should provide the data to support these claims.

Our Response:

We thank the reviewer for asking us about this. We investigated this more closely and found that our initial sentence "In contrast, the brain and gills exhibited minimal change or a decrease in baseline calcium levels during hypoxia" was overly simplified. We looked more carefully at the data and also performed further experiments and observed some changes in baseline calcium level in these organs. We now show in the Extended Data Figure 11 these average tissue baseline calcium dynamics for the organs mentioned, and modified the text to better reflect these. We note that we also observed regional differences within the brain and gills, which would be an interesting future research direction. As this was not the focus of the study we hope that tissue averages provide a sufficient indication of the existence of alteration which we plan to characterize in more depth in future research. To summarize, we removed the overly-simplified statement, and we have updated Extended Data Figure 11 to show in more detail how the dynamics in these tissues unfolds over time. The updated text and new panel are presented below (line 399):

> *"Other organs also showed changes in baseline calcium levels, though the gastrointestinal tract, at the average tissue level, showed among the most notable initial increase in baseline calcium levels (Extended Data Fig. 11a)."*

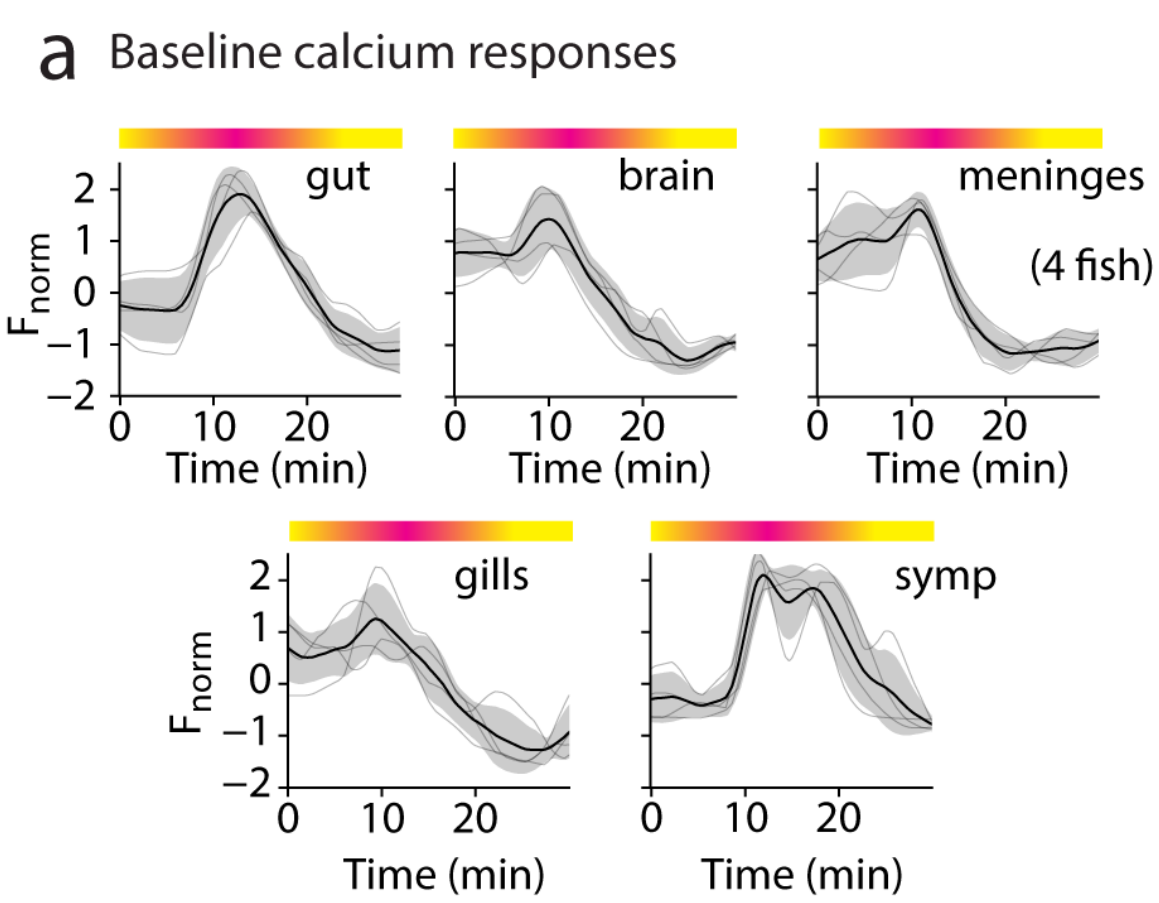

Extended Data Fig. 11: **Body-wide circuit engaged in response to hypoxic stress.**
a, Hypoxia induces changes in baseline calcium levels in multiple organs. Average organ baseline calcium levels (dark: mean response, light: individual animals, shaded gray: standard deviation, N=4).

Reviewer Comment:

9. Although WHOLISTIC is a powerful method for whole-body imaging of animals, all functional imaging was performed on laterally mounted fish immobilized by 2% agarose, which is not a natural posture of zebrafish. Thus, it might be good to add this limitation in the discussion section and mention that it can be improved in future studies.

Our Response:

We agree and have edited the text in the Discussion section to note this (line 462):

"Future implementations of WHOLISTIC in freely behaving animals will enable the study of body-wide cellular control systems in naturalistic settings, rather than in an embedded preparation."

We have further added a discussion in the Supplemental Notes (Future Extensions section) where we highlight that the animal is not in its natural posture, and outline means of overcoming this in future iterations of the system, as well as the possibility of combining microscopes compatible with imaging freely-behaving fish with whole body imaging (line 2025):

"We foresee multiple extensions of WHOLISTIC which would extend the scope of this system. In this instantiation, a) samples are embedded in agarose which limits their movement; moreover, to best image the viscera, b) fish are placed on their sides which is not their natural posture; furthermore, spinning-disk confocal microscopy was used which induces c) more bleaching than light-sheet microscopy or 2-photon microscopy and is relatively slow. Finally, d) with the main objective used in this study, 20×0.75NA, the field of view was restricted to 800 μm ×800 μm. To overcome this, we foresee using a large field of view 2-photon microscope[8] and positioning a 45° mirror next to the fish, making it possible to image the entire length of the fish, positioned in its natural posture, whilst providing access to both the brain and viscera (via the mirror) simultaneously. Such a microscope also includes a resonant scanner and offers the capacity to do dual plane imaging, which would double the frame rate. Similar extensions could be made to freely swimming imaging setups[9] to enable whole body imaging in freely behaving animals."

Reviewer Comment:

10. Line 584: I read that the Danionella samples were imaged in artificial cerebrospinal fluid. This appears surprising because it is quite different from the water the animals live in. The authors will want to clarify why they used such buffer.

Our Response:

The use of aCSF for Danionella imaging was for historical reasons as we had begun doing so when first imaging this species of fish. However, the use of aCSF is not required and we agree that it is a distraction. We therefore repeated the experiments in normal fish water and achieved similar results. We now show these data in Extended Data Figure 1, and we have removed all data collected in aCSF. The new panel is shown below:

d Calcium activity dynamics

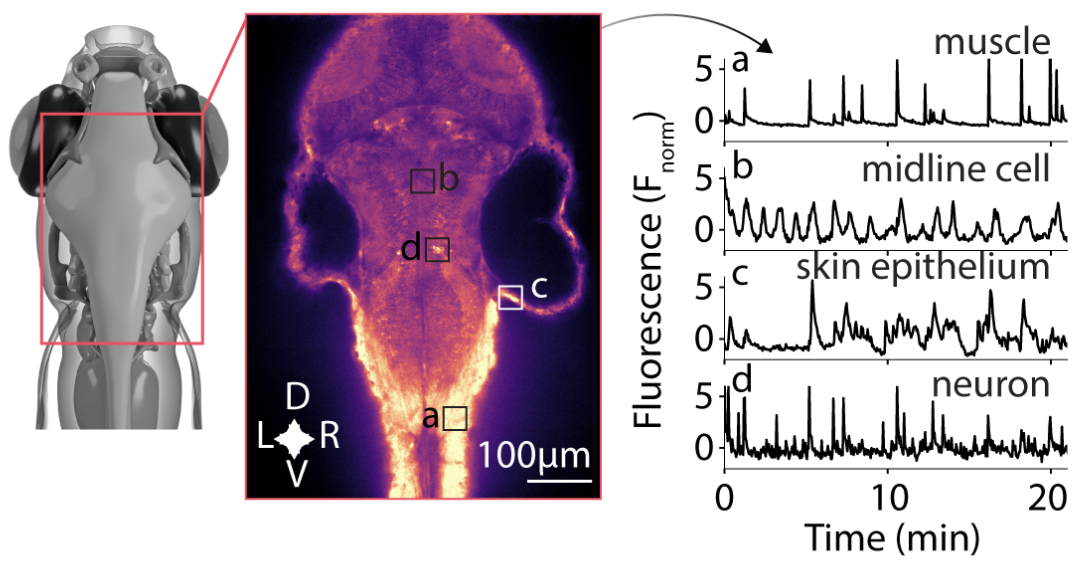

Extended Data Fig. 1: **WHOLISTIC transgenics and workflow.**
d, Calcium activity dynamics from *Danionella cerebrum* WHOLISTIC line. *Left*: dorsal view of brain. *Right*: example time-series from a) muscle, b) cell in midline of brain (unidentified), c) skin epithelium and d) neuron.

Reviewer Comment:

11. Data sharing: The authors described in the manuscript that they will be sharing the raw data upon publication. I recommend that the authors provide user friendly data pipelines and robust metadata information, so that the data is useable and useful to the scientific community. These datasets will be extremely valuable and could open up many possibilities to labs that have or do not have the facilities to perform such experiments. The authors designed a webpage describing their findings. The webpage is very well documented and gives good information about the project in general. This webpage could serve as the gateway to the datasets.

Our Response:

We thank the reviewer for their positive feedback and suggestions. We fully agree with the importance of data sharing and usability. The project webpage indeed serves as the primary gateway to access all datasets and associated resources. We have updated the website with a data-sharing page: https://wholistic.janelia.org/data/. This page contains a list of codebases, list of transgenic lines, datasets with accompanying metadata, and detailed step-by-step protocols. We have also made the ExM protocols available on protocols.io: dx.doi.org/10.17504/protocols.io.dm6gp9wxjvzp/v3. We are committed to providing open-access to these resources and maintaining them to be up-to-date. Below are some screenshots of the website:

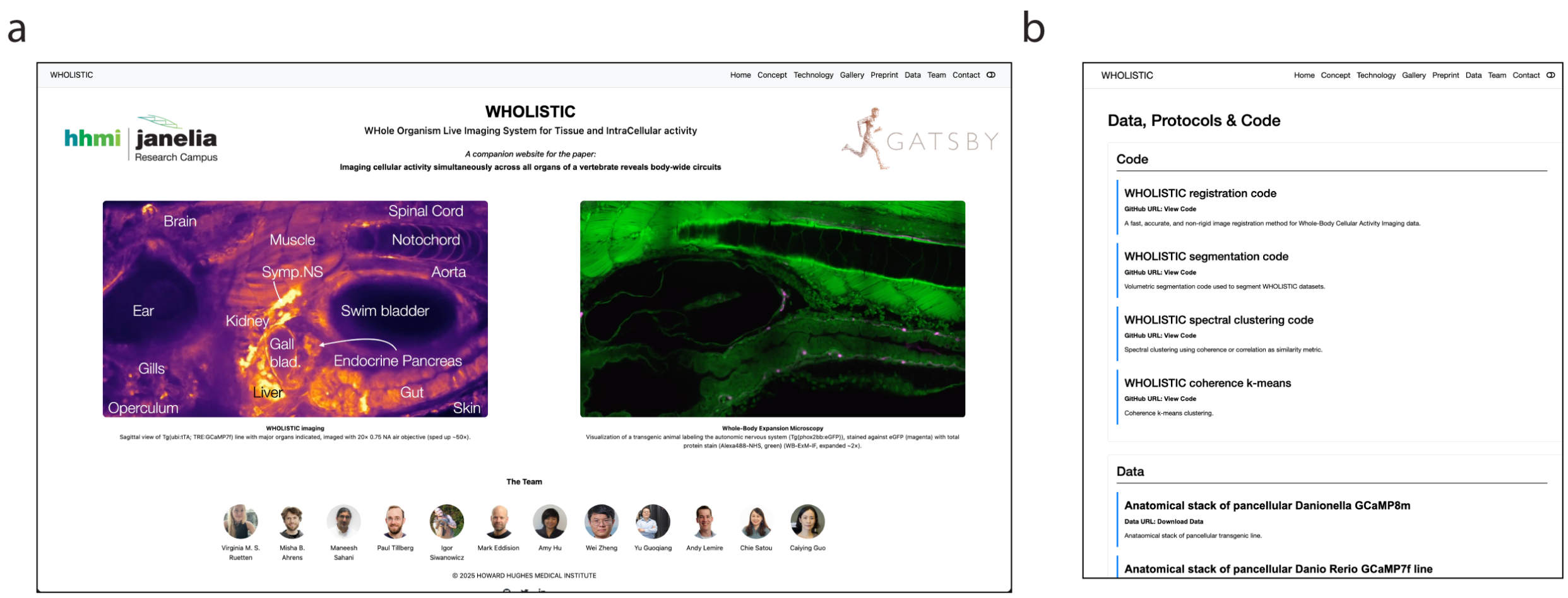

Review Data Fig. 1: **WHOLISTIC website for centralized distribution of resources**
a, Home page of WHOLISTIC website hosted at https://wholistic.janelia.org/ with access points to pages describing technicalities of the methodology as well resource Data page.
b, Added Data page for centralized hosting of data, protocols and code.

Minor textual comments (Reviewer #1)

Reviewer Comment:

At line 24, please spell the full name of WHOLISTIC first.

Our Response:

The full name "WHole Organism Live Imaging System for recording Tissue and IntraCellular activity (WHOLISTIC), is now introduced at its first mention in the Summary (line 24).

Reviewer Comment:

2. At line 31, please change "all cells" to "most cells" as it looks the entire body was not imaged (e.g. missing the tail part in the dataset) and some cells may have been missed.

Our Response:

Thank you, this has been changed. We now use "most cells" (line 32 in the revised manuscript) or similar phrasing where appropriate.

Reviewer Comment:

3. Line 78: The subtitle might need to be changed, such as "WHOLISTIC captures cellular calcium activity across organ systems" since they only measured calcium activities.

Our Response:

The subtitle has been changed to “WHOLISTIC captures cellular calcium activity across organ systems.”

Reviewer Comment:

4. Line 289: It might be better to replace “regular swimming” to “movement” since the imaged fish were immobilized in agarose.

Our Response:

We thank to reviewer for this comment. To avoid any ambiguity, we have replaced “regular swimming” with “motor activity” as we think it best describes the observation, without implying that the animals are free to move (line 292 in the revised manuscript).

Reviewer #1 (Remarks on code availability)

Reviewer Comment:

We have not been able to do this due to limited time.

Reviewer #2 (Remarks to the Author)

Reviewer Comment:

I co-reviewed this manuscript with one of the reviewers who provided the listed reports.

Reviewer #3 (Remarks to the Author)

Reviewer Comment:

The manuscript presents a method aiming at enabling the recording of calcium dynamics across the entire body in transparent vertebrates. By engineering a transgenic pancellular fish line expressing calcium indicators in nearly all cells across different organs, and combining it with optimized imaging and computational tools, the study demonstrates how coordinated cellular dynamics across organs can be visualized and analyzed. This approach offers valuable insights into the connection between cellular and organismal physiology, potentially advancing our understanding of complex behaviors and diseases. Some issues should be addressed, below:

Our Response:

We thank Reviewer #3 for their constructive feedback and suggestions for improving our manuscript. We address the major and minor comments below.

Major Comments (Reviewer #3)

Reviewer Comment:

Calcium is imaged, largely as a matter of convenience. But what is the physiological meaning of calcium in these different cells, tissues, and organs? It would be good to know what calcium signifies in these different locations. Probably it is too much to ask for further experiments along these lines, but perhaps a table reviewing the relevant literature would be helpful, explaining what intracellular calcium signals represent in different cells, and what it is indicating regarding biological dynamics.

Our Response:

We appreciate the reviewer's question about the physiological meaning of calcium across different cell types. This is an important point that helps contextualize our findings, and we have considered this deeply as well, leading to our discussion in the manuscript about the need to use additional indicators to gain more insight into cell-biological processes. As the reviewer suggested, we have added a supplementary table summarizing the established roles of calcium signaling across a range of cell types, along with key references (presented below; see line 2061). We make sure to mention that the list is not exhaustive because not all the relevant literature can be captured in one table, and because we note that for several cell types studied in our paper (such as nephric epithelial cells), the precise functional meaning of calcium transients remains to be fully elucidated, which represents an exciting direction for future investigation. We are also excited about extending WHOLISTIC to other modalities beyond calcium to provide complementary views of cellular physiology, including, for example, relationships between calcium and secreted molecules, which could be visualized by multiplexing different indicators in different spectral channels.

Example of known roles of calcium in various cell types

Cell type	Associated Organ/System	Example of role of calcium in cell type	Reference	Reference URL
Neuron	Nervous system (CNS/PNS)	Ca^{2+} influx through voltage-gated CaV channels couples electrical activity to neurotransmitter release by binding synaptotagmins to trigger synaptic vesicle fusion; it also regulates synaptic plasticity and gene transcription.	Sudhof TC. Calcium control of neurotransmitter release. Cold Spring Harbor Perspectives in Biology. 2012 Jan;4(1):a011353. https://doi.org/10.1101/cshperspect.a011353	https://cshperspectives.cshlp.org/content/4/1/a011353.full
Astrocyte	Nervous system (CNS)	Ca^{2+} elevations in astrocytes mediate ATP release.	Khakh, B.S. & McCarthy, K.D. (2015). Astrocyte calcium signaling: from observations to functions and the challenges therein. Cold Spring Harb Perspect Biol. 7(4):a020404.	https://pubmed.ncbi.nlm.nih.gov/25605709/
Microglia	Nervous system (CNS)	Ca^{2+} transients control motility and release of cytokines.	Kettenmann, H. et al. (2011). Physiology of microglia. Physiol Rev. 91(2):461-553.	https://pubmed.ncbi.nlm.nih.gov/21527731/
Radial glial cell	Nervous system (CNS)	Ca^{2+} dynamics regulate neural stem cell proliferation/differentiation. Ca^{2+} oscillations influence cell-cycle progression.	Weissman, T.A. et al. (2004). Calcium waves propagate through radial glial cells and modulate proliferation in the developing neocortex. Neuron. 43(5):647-61.	https://pubmed.ncbi.nlm.nih.gov/15339647/
Oligodendrocyte	Nervous system (CNS)	Ca^{2+} transients control myelin sheath growth. Activity-dependent Ca^{2+} in sheaths promotes actin remodeling and elongation.	Oligodendrocyte calcium signaling promotes actin-dependent myelin sheath extension	https://pubmed.ncbi.nlm.nih.gov/38177161/
Photoreceptors	Eye (retina)	Ca^{2+} controls phototransduction adaptation and synaptic release.	Pugh, E.N. Jr. & Lamb, T.D. (2000). Phototransduction in vertebrate rods and cones: molecular mechanisms of amplification, recovery and light adaptation. Handb Biol Phys. 3:183-255.	https://www.sciencedirect.com/science/article/pii/S1383812100800081
Inner ear hair cell	Ear (auditory/vestibular)	Mechanotransduction induces Ca^{2+} entry and contributes to synaptic release.	Fuchs, P.A. (2005). Time and intensity coding at the hair cell's ribbon synapse. J Physiol. 566 (Pt 1):7-12.	
Neuromast hair cell	Lateral line	Ca^{2+} involved in mechanotransduction and synaptic release.	Synaptically silent sensory hair cells in zebrafish are recruited after damage	https://www.nature.com/articles/s41467-018-03806-8
Endothelial cell	Vasculature	Ca^{2+} controls permeability and angiogenesis.	Endothelial Cell Calcium Signaling during Barrier Function and Inflammation	https://pmc.ncbi.nlm.nih.gov/articles/PMC7074364/
Cardiomyocyte	Heart	Excitation-contraction coupling via Ca^{2+}-induced Ca^{2+} release. L-type Ca^{2+} influx triggers RyR-mediated SR Ca^{2+} release, activating troponin C.	Bers, D.M. (2002). Cardiac excitation-contraction coupling. Nature. 415(6868):198-205.	https://pubmed.ncbi.nlm.nih.gov/11805843/
Mural cell (pericyte/smooth muscle)	Vasculature	Pericyte/smooth muscle contraction depends on Ca^{2+}-calmodulin-MLCK. Vasoactive signals elevate Ca^{2+} to control tone and capillary diameter.	Hill-Eubanks, D.C. et al. (2011). Calcium signaling in smooth muscle. Cold Spring Harb Perspect Biol. 3(9):a004549.	https://pubmed.ncbi.nlm.nih.gov/21709182/
Erythrocyte (red blood cell)	Blood	Ca^{2+} is involved in regulating volume and intercellular adhesion.	Stimulation of human red blood cells leads to Ca2+-mediated intercellular adhesion	https://pubmed.ncbi.nlm.nih.gov/21616535/
Thrombocyte (platelet-equivalent)	Blood	Platelet activation and secretion require Ca^{2+}. Ca^{2+} release/influx drives shape change, integrin activation, granule exocytosis.	Varga-Szabo, D. et al. (2009). Calcium signaling in platelets. J Thromb Haemost. 7(7):1057-66.	https://pubmed.ncbi.nlm.nih.gov/19422456/
Skeletal muscle fiber	Muscle (somitic)	Depolarization triggers SR Ca^{2+} release and contraction. DHPR-RyR coupling releases Ca^{2+}; troponin C binding initiates cross-bridge cycling.	Melzer, W. et al. (1995). The role of Ca^{2+} ions in excitation-contraction coupling of skeletal muscle fibres. Biochim Biophys Acta. 1241(1):59-116.	https://pubmed.ncbi.nlm.nih.gov/7742348/
Smooth muscle cell	Muscle (visceral/vascular)	Ca^{2+}-calmodulin-MLCK pathway drives contraction.	Nelson et al. (2011). Calcium Signaling in Smooth Muscle. Cold Spring Harbor Perspectives in Biology.	https://pmc.ncbi.nlm.nih.gov/articles/PMC3181028/
Chondrocyte	Cartilage	Ca^{2+} mediates mechanotransduction and response to osmotic stress	Li W, Zhou Y, Han L, Wang L, Lucas Lu X. Calcium signaling of primary chondrocytes and ATDC5 chondrogenic cells under osmotic stress and mechanical stimulation. J Biomech. 2022 Dec;145:111388. doi: 10.1016/j.jbiomech.2022.111388. Epub 2022 Nov 17. PMID: 36413831; PMCID: PMC10472919.	https://pmc.ncbi.nlm.nih.gov/articles/PMC10472919/
Osteoblast	Bone	Regulates osteoblast differentiation.	Hao Y, Yang N, Sun M, Yang S, Chen X. The role of calcium channels in osteoporosis and their therapeutic potential. Front Endocrinol (Lausanne). 2024 Aug 7;15:1450328. doi: 10.3389/fendo.2024.1450328. PMID: 39170742; PMCID: PMC11335502.	https://pmc.ncbi.nlm.nih.gov/articles/PMC11335502/
Osteocyte	Bone	Ca^{2+} transients encode mechanical load, induce vesicular release and induce actin contraction.	Morrell, A.E., Brown, G.N., Robinson, S.T. et al. Mechanically induced Ca2+ oscillations in osteocytes release extracellular vesicles and enhance bone formation. Bone Res 6, 6 (2018). https://doi.org/10.1038/s41413-018-0007-x	https://www.nature.com/articles/s41413-018-0007-x

Cell type	Associated Organ/System	Example of role of calcium in cell type	Reference	Reference URL
Osteoclast	Bone	Ca^{2+} regulates cytoskelatal organization, elevated calcium inhibits resorbing activity.	Li, Zhenpeng, Kangmei Kong, and Weili Qi. "Osteoclast and its roles in calcium metabolism and bone development and remodeling." Biochemical and biophysical research communications 343.2 (2006): 345-350.	https://www.sciencedirect.com/science/article/pii/S0006291X06004608#section.0070
Notochord cell	Axial skeleton (embryonic)	Ca^{2+} contributes to cell elongation and intercalation.	Mapping calcium dynamics in a developing tubular structure<br>Jorgen Hoyer, Morsal Saba, Daniel Dondorp, Kushal Kolar, Riccardo Esposito, Marios Chatzigeorgiou<br>bioRxiv 2020.10.16.342535; doi: https://doi.org/10.1101/2020.10.16.342535	https://www.biorxiv.org/content/10.1101/2020.10.16.342535v1.full
Goblet cell	Intestine	Ca^{2+} triggers mucin granule exocytosis.	Vivek Malhotra (2013) TRPM5-mediated calcium uptake regulates mucin secretion from human colon goblet cells eLife 2:e00658.	https://elifesciences.org/articles/00658#content
Hepatocyte	Liver	Hormone-evoked Ca^{2+} waves induce glucose mobilisation through regulation of gluocogenolysis and neoglucogenesis.	Humbert, Alexandre, et al. "Calcium signalling in hepatic metabolism: Health and diseases." Cell (	https://www.sciencedirect.com/science/article/pii/S0143416023000921#sec0002
Pancreatic alpha cell	Pancreas (endocrine)	Voltage-gated Ca^{2+} drives glucagon release at low glucose.	Rorsman P, Braun M, Zhang Q. Regulation of calcium in pancreatic α- and β-cells in health and disease. Cell Calcium. 2012 Mar-Apr;51(3-4):300-8. doi: 10.1016/j.ceca.2011.11.006. Epub 2011 Dec 15. PMID: 22177710; PMCID: PMC3334273.	https://pmc.ncbi.nlm.nih.gov/articles/PMC3334273/
Pancreatic beta cell	Pancreas (endocrine)	Ca^{2+} influx directly triggers insulin exocytosis. KATP closure→depolarization→Cav channels→Ca^{2+}-triggered granule fusion.	Rorsman, P. & Ashcroft, F.M. (2018). Pancreatic β-cell electrical activity and insulin secretion: of mice and men. Physiol Rev. 98(1):117-214.	https://pubmed.ncbi.nlm.nih.gov/29212789/
Juxtaglomerular cells	Kidney (glomerulus)	Ca^{2+} inhibits renin secretion.	Staruschenko A, Alexander RT, Caplan MJ, Ilatovskaya DV. Calcium signalling and transport in the kidney. Nat Rev Nephrol. 2024 Aug;20(8):541-555. doi: 10.1038/s41581-024-00835-z. Epub 2024 Apr 19. PMID: 38641658; PMCID: PMC12036682.	https://pmc.ncbi.nlm.nih.gov/articles/PMC12036682/#S15
Chromaffin cell	Head kidney (adrenal medulla homolog)	Depolarization-triggered Ca^{2+} induces catecholamine secretion.	García, Antonio G., et al. "Calcium signaling and exocytosis in adrenal chromaffin cells." Physiolo	https://journals.physiology.org/doi/full/10.1152/physrev.00039.2005
Interrenal steroidogenic cell	Head kidney (adrenal cortex homolog)	Sustained Ca^{2+} influx increases steroidogenesis.	Rossier, M. F. "T-type calcium channel: a privileged gate for calcium entry and control of adrenal steroidogenesis. Front Endocrinol. 2016; 7: 43."	https://pmc.ncbi.nlm.nih.gov/articles/PMC4873500/
Pituitary corticotroph (ACTH)	Pituitary	CRH stimulates Ca^{2+} signaling to release ACTH. cAMP-PKA primes secretion; Ca^{2+} is final trigger for granule fusion.	Tse, Amy, Andy K. Lee, and W. Tse Frederick. "Ca2+ signaling and exocytosis in pituitary corticotropes." Cell Calcium 51.3-4 (2012): 253-259	https://www.sciencedirect.com/science/article/pii/S0143416011002363
Kupffer cell (liver macrophage)	Liver	Ca^{2+} contributes to Kupffer cell M2 polarization.	Xu, Xue-song, et al. "SCARF1 promotes M2 polarization of Kupffer cells via calcium-dependent PI3K-AKT-STAT3 signalling to improve liver transplantation." Cell proliferation 54.4 (2021): e13022.	https://onlinelibrary.wiley.com/doi/10.1111/cpr.13022
Ultimobranchial (calcitonin) cell	Ultimobranchial gland	Systemic Ca^{2+} regulates calcitonin secretion. High plasma Ca^{2+} stimulates calcitonin release to lower Ca^{2+}; Ca^{2+} acts as both signal and target.	Fudge, N.J., Kovacs, C.S. Physiological studies in heterozygous calcium sensing receptor (CaSR) gene-ablated mice confirm that the CaSR regulates calcitonin release in vivo . BMC Physiol 4, 5 (2004). https://doi.org/10.1186/1472-6793-4-5	https://bmcphysiol.biomedcentral.com/articles/10.1186/1472-6793-4-5#:~:text=The%20calcium%20sensing%20receptor%20(CaSR,responsiveness%20of%20calcitonin%20to%20calcium.
Skin epithelial cells	Skin	Ca2+ signaling drives cell cycle progression	Moore, Jessica L., et al. "Cell cycle controls long-range calcium signaling in the regenerating epid	https://rupress.org/jcb/article/222/7/e202302095/214066/Cell-cycle-controls-long-range-calcium-signaling
Enteroendocrine cell	Intestine	Stimulus-secretion coupling via Ca^{2+}. Nutrient cues evoke Ca^{2+} to release peptides (e.g., 5-HT, CCK);	Davison, Adam, Frank Reimann, and Fiona M. Gribble. "Molecular mechanisms of stimulus detect	https://www.sciencedirect.com/science/article/pii/S0959
Monocyte/Macrophage	Immune (innate)	Purinergic receptors is strongly coupled to Ca2+-signaling; Ca2+ involved in phagosome maturation, engulfment of apoptotic cells, migration.	Desai, Bimal N., and Norbert Leitinger. "Purinergic and calcium signaling in macrophage function	https://pmc.ncbi.nlm.nih.gov/articles/PMC4245916/

Reviewer Comment:

2. The manuscript claims "all cells in the body" and other similar claims, throughout. But it is unclear whether all cell types are truly represented – are some organs, like heart, lacking expression? What fraction of cells in each organ is expressing the indicator, and can be imaged? Most of the studies presented seem to focus on a small number of organs - did any experiment truly require analysis of collective or correlated calcium dynamics across all organs in the entire fish? If not, perhaps some of the claims should be toned down.

Our Response:

We thank the reviewer for this important point about the comprehensiveness of our imaging and the scope of our claims. We have carefully reviewed the manuscript and moderated our language where appropriate, changing "all cells" to "most cells" or similar phrasing throughout (e.g. line 32).

Regarding whether experiments required whole-body analysis: We agree that some of the individual experiments could potentially be performed with targeted approaches, however we would respectfully advance that the main findings of the paper substantially benefited from, or required, simultaneous whole-body imaging. For instance, uncovering the coupling between muscle groups and numerous other cell types across the body would have been laborious to do otherwise, requiring the generation of numerous transgenics and running of many experiments. The motor-quiescence-coupled ependymal oscillations were discovered through a whole-body screening, and narrowed down on a cell type that we would not have specifically targeted. In summary, using a single transgenic line and analysis pipeline, WHOLISTIC revealed these unexpected cellular interactions across anatomically distant regions, demonstrating how more comprehensive imaging can complement targeted investigations for biological discovery.

Regarding labeling, the heart is labeled and was actually present in Extended Data Figure 1 - we have now labeled the data with location of ventricle to make it clearer to spot. That said, its dynamics are fast, and so capturing them faithfully would require more tailored experiments. As calcium dynamics in the heart have already been the focus of several papers[10,11,12] we opted not to focus on these.

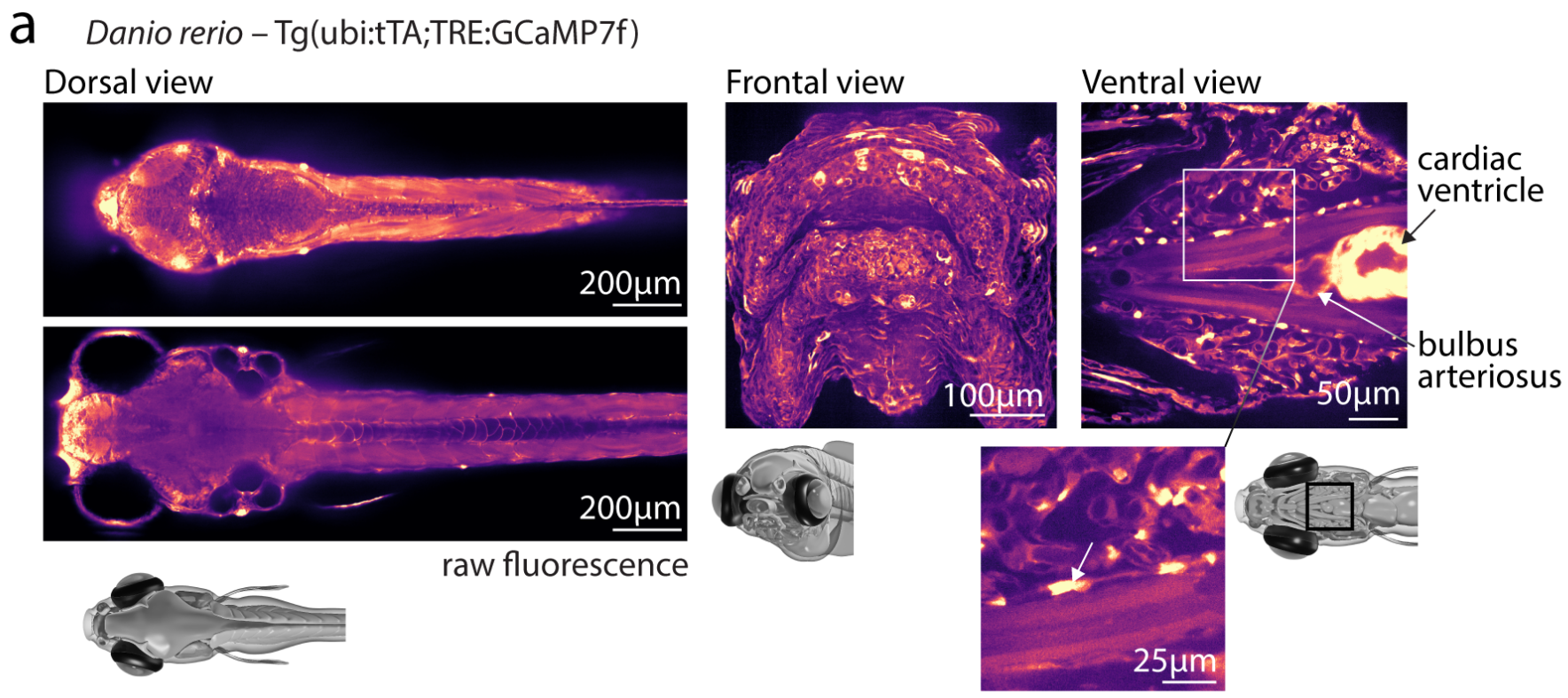

Extended Data Fig. 1: **WHOLISTIC transgenics and workflow.**
a, Complementary views of WHOLISTIC zebrafish transgenic. *Left to right*: 1) dorsal view (two planes at different depths), 2) frontal view (maximum projection), 3) ventral views of gill and heart regions with cardiac ventricle and bulbus arteriosus labeled, along with enlarged view containing unidentified cell types.

Regarding the broader question of labeling density, we fully agree with the reviewer of the importance of this questions. Ideally, we would quantitatively assess the fraction of cells labeled. However, obtaining meaningful estimates is not straightforward due to large differences in cell morphologies and the presence of GCaMP-positive regions that do not contain cell bodies per se (e.g., neuropil in the brain). We reasoned that if we could label all nuclei, we could then assess what fraction contain a GCaMP-positive nuclei or are surrounded by GCaMP-positive cytosol.

We explored multiple strategies to label nuclei while preserving endogenous GCaMP signal:

1. We attempted to use a transgenic line from another group designed to label all nuclei, but found this line to be sparser than ours; therefore, this was not a suitable strategy.
2. We tried live nuclei-labeling strategies (e.g., Hoechst); however, even with extensive protocol optimization, we achieved only superficial labeling of the skin and superficial structures.
3. We tried fixing, clearing, and labeling with DAPI, but found image quality to be significantly inferior to live imaging, making it difficult to discern deeper nuclei, even when using 2-photon microscopy.
4. We tried our whole-body expansion protocol combined with immunolabeling using anti-GCaMP antibody; however, multiple rounds of optimization failed due to target density (GCaMP being expressed in most cells), which rapidly led to antibody depletion; therefore, this strategy could not be used.
5. We attempted to adapt our whole-body expansion protocol to retain GCaMP signal using mild disruption, but found that even a modest 1-hour disruption at 50°C was sufficient to destroy all GCaMP signal.

6. Finally, we attempted a modification of whole-body ExM requiring no disruption while still providing some clearing. This succeeded: we could visualize endogenous GCaMP signal, nuclei (via DAPI), and total-protein stain, with no visible cracks and improved clearing compared to fixing and clearing alone. Nonetheless, substantial scattering remained, such that deeper tissues appear substantially dimmer than superficial ones. We present these data in Extended Data Figure 12.

Using the data from the final strategy, we segmented nuclei using Cellpose (Extended Data Figure 12d) and could extract nuclei throughout the tissue. However, identifying which nuclei are associated with GCaMP-positive cytoplasm, and doing so algorithmically in a robust manner is challenging due to:

1. Signal attenuation along sample depth of the sample due to scattering, which means if one wants to compare brightness to a threshold to ascertain labeling, this threshold would need to vary in space to compensate for baseline attenuation
2. The variability of GCaMP expression between nuclei and cytosol of cells. This prevents the straightforward strategy of, for instance, computing the fluorescence ratio between the nuclei and its outer cytoplasmic rim, which would self-normalize for scattering/attenuation.

We also considered using total protein stain signal within nuclei as a reference (assuming roughly similar protein concentration across nuclei) to compute the ratio of nuclei GCaMP brightness to total protein stain as a metric of expression that would be normalized for signal scattering across the sample. However, some cells (e.g., nephric cells) have nuclei as bright as their cytoplasm, whereas others (e.g., neurons) have nuclei much dimmer than their labeled cytosol (that would bring them below the cut-off threshold for qualifying them as labeled). Thus, normalizing average GCaMP nuclear signal by average total protein nuclear signal did not seem a reliable approach either. We also considered measuring signal in an annulus outside each segmented nucleus, normalized by the NHS signal of that same region, but since protein content differs substantially between cells, this too proved unsuitable as a normalizer. Despite successfully segmenting a large fraction of nuclei while retaining decent-quality GCaMP signal, we were unsatisfied by the obvious metrics one might be able apply across the whole dataset in a scaleable and automated way. We have uploaded the data to the website and described the protocol used to generate it. Finally, we resorted to manually counting the fraction of positive cells as best as we could in one representative founder (by taking a random sample of segmented nuclei and judging if these cells were positive), we found that in most tissues (muscles, gills, brain, gastrointestinal tract, gallbladder, notochord) 85-100% of the tissue was labeled, the kidney had intermediate labeling (75-90%). The one organ that had substantially less cells labeled was the exocrine pancreas, where the cells lining the tubules (ductal cells) were well labeled ($\sim$ 85-100%) but the acinar cells were poorly labeled. These numbers vary slightly depending on founders, with for instance the exocrine pancreas being fully

labeled in one of the two founders presented in Extended Data Figure 12a. As mentioned, we have uploaded the full dataset to the website so that users that inspect the data. We have added these data to Extended Data Figure 12 along with examples of 1) regions well labeled, 2) regions not labeled due to lack of cellular content (e.g., swim bladder) and 3) areas that had poor labeling (minority) (e.g., exocrine pancreas). We have added our preliminary analysis of nuclear segmentation (Extended Data Figure 12d) and ratio of GCaMP to total protein stain signal with nuclei (Extended Data Figure 12e); however, we think the utility of this metric is limited due to the subtleties of GCaMP localization, which appears to be cell-type dependent. We think that addressing this question systematically and at scale would be best done by generating a new transgenic line that expresses GCaMP as well as a nuclear-targeted fluorescent marker, combined with further improvement of our Whole Body Expansion protocol to further decrease scattering whilst retaining GCaMP signal, or dissociating all cells and FACS sorting them (however, this would lose spatial information). We made our best effort at answering this question meaningfully as we also believe it is important, and hope that our intermediary result and new data enabling joint visualization of endogenous GCaMP and nuclei, which allows for better assessment of labeling density, will satisfy the reviewer - we have added a discussion on this in the Future Extensions section included in the Supplementary Notes, that we have copied below (line 2050):

> *"Finally, regarding transgenic coverage, while we qualitatively demonstrate broad pancellular expression, rigorous, automated and scalable quantification of the fraction of labeled cells proved technically challenging (Extended Data Figure 12c). Future work would benefit from generating a new transgenic line co-expressing GCaMP with a spectrally distinct nuclear marker under the same promoter, enabling unambiguous, automated quantification of labeling density across all tissues."*

Below is the updated Extended Data Figure 1, and the new Extended Data Figure 12 generated to address the review comments:

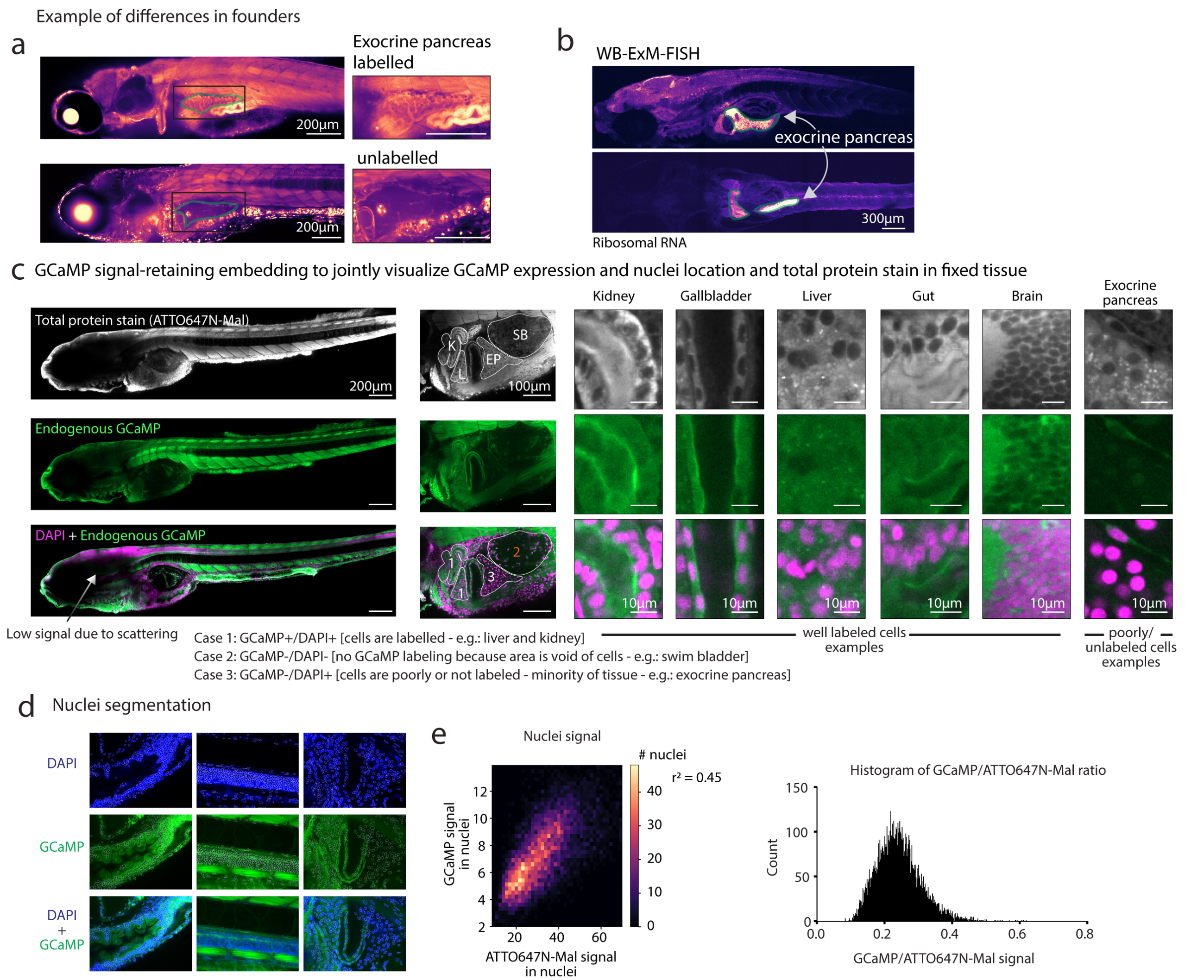

Extended Data Fig. 12: **Example of differences in pancellular founders and joint visualization of GCaMP signal along with nuclei**

a, Transgenic lines generated via Tol2 transgenesis have non-deterministic landing sites and are therefore sensitive to positional effects. Consequently, founders exhibit variable expression patterns and must be carefully screened for dense, uniform expression. Shown are offspring from two founders in which the exocrine pancreas is (*top*) or is not (*bottom*) labeled.
b, Sagittal (*top*) and ventral (*bottom*) views of a whole-body expansion microscopy (WB-ExM) sample (7 days post-fertilization) stained for ribosomal RNA, highlighting dense labeling in the exocrine pancreas.
c, GCaMP signal-retaining gel embedding for assessing expression density. Determining which cells express GCaMP requires unbiased localization of all cell bodies, achievable through nuclear labeling. Expansion protocols induce a drop in fluorescence signal and so could not be used. For nuclear DAPI staining, samples need to be fixed which results in more tissue scattering compared to live samples. We developed an embedding method that preserves endogenous GCaMP signal while still providing decent optical access throughout the tissue; we note that the quality of live-imaging or ExM data is still superior to this. *Left*: Example sample showing total-protein stain (ATTO647N-Maleimide, *top*), preserved endogenous GCaMP (*middle*), and DAPI (*bottom*). *Right*: Enlarged views of various tissues (kidney, gallbladder, liver, exocrine pancreas, intestine, brain) illustrating three cases: (1) cells positive for both GCaMP and DAPI; (2) regions lacking both GCaMP and DAPI, corresponding to acellular structures such as the swim bladder and notochord; (3) DAPI-positive nuclei with little, to no surrounding GCaMP signal, indicating poorly labeled cells (e.g., exocrine pancreas in this founder). In general, we found the vast majority of cells to be well labeled.
d, Example of nuclei segmentation using CellPose (outlined in white).
e, *Left*: 2D histogram of average nuclear GCaMP signal vs. average nuclear total protein signal, showing these to be correlated. *Right*: 1D histogram of ratio between nuclear GCaMP signal and average nuclear total protein signal, showing a unimodal distribution.

Reviewer Comment:

3. The authors present a novel whole-organism expansion microscopy method (WB-ExM) different from other recent protocol, but it is unclear whether they have validated resolution and distortion in the ways that most novel expansion microscopy method are presented. The authors should characterize the isotropy of the expanded tissue for their method, particularly since the method iterates the process many times, an unusual process.

Our Response:

We appreciate the reviewer's question. We have performed further characterization of our expansion method that we now present in Extended Data Figures 9-10. Specifically, we have considered:

Resolution and clearing quality: We have quantified the resolution across an expanded sample using a commonly used approach in the field of super-resolution microscopy - Fourier Ring Correlation[13], and have added this to the Supplementary Material (Extended Data Figure 9d - see below). Critically, we found minimal attenuation in resolvable spatial frequencies across the sample depth, demonstrating excellent clearing quality throughout the tissue.

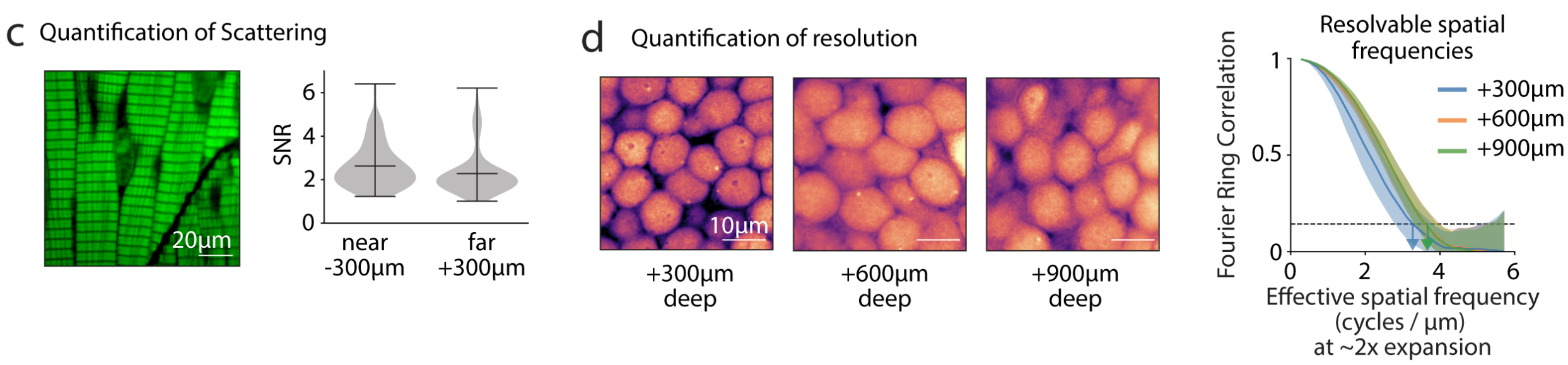

Extended Data Fig. 9: **Whole-Body Expansion Microscopy (WB-ExM) to map ultrastructure, RNA and protein across the body.**
c, Quantification of clearing quality of WB-ExM sample. *Left*: Image of muscle sarcomeres in WB-ExM sample stained with total protein stain (Alexa488-NHS). *Right*: Distribution of signal to noise ratio (peak to trough) in sarcomeres at far and near side of the sample.
d, Quantification of resolvable spatial frequencies in WB-ExM sample. *Left*: Image of neurons located at different depths within the brain, stained with total protein stain (Alexa488-NHS). *Right*: Fourier ring correlation of WB-ExM data at different depths showing little attenuation in resolvable frequencies. The spatial frequency at which the correlation falls below 1/7 is taken as the resolution cut-off[13].

Isotropy and distortion: Quantifying the distortion and isotropy across such large samples in an unbiased way is challenging. Previous studies have often relied on measuring centriole diameter[14,15] to assess local deformation, but this approach has significant limitations: (1) it samples only a single subcellular feature that may not reflect tissue-scale deformations, (2) some tissues (like the vacuolated notochord) lack centrioles in large regions, and (3) it would not detect common artifacts like organ detachment (e.g., brain separating from skull base). We therefore deemed that, whilst doable, measuring a single subcellular feature, or even a class of a subcellular feature, was unlikely to convincingly prove the quality of our protocol.

We therefore developed PhotoMap (Extended Data Figure 10, line 325, 1095, 2164, and protocols.io - see below), a method to quantify gel deformation fields across entire samples in an unbiased manner. A evenly spaced 3D grid is photobleached into the sample, embedded into a gel engineered to be intrinsically fluorescent, prior to expansion and re-imaged afterward, enabling direct visualization and quantification of spatial distortions throughout the tissue. We found that, overall, the distortions are relatively limited, with the exception of the cleithrum, the earliest mineralizing bone in zebrafish[16], which introduced deformations, nonetheless these remained local and did not propagate. We also provide a comprehensive step-by-step protocol in the Supplementary Notes to enable reproduction of this method, and have also made this available at protocols.io: dx.doi.org/10.17504/protocols.io.dm6gp9wxjvzp/v4, and made the analysis code available at: https://github.com/vruetten/PhotoMap.git. Below, is the new figure panel that we have added thanks to the reviewer's comment:

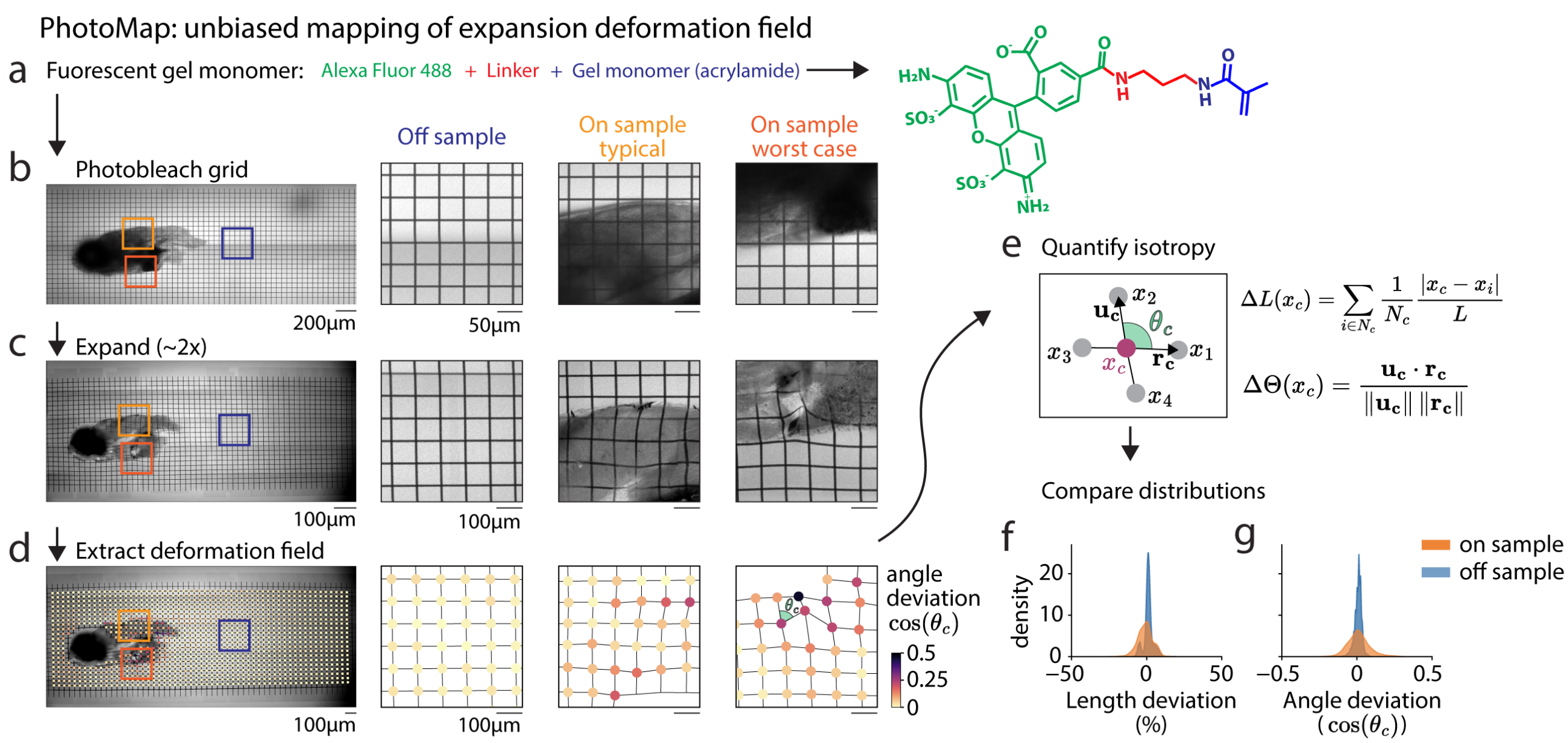

Extended Data Fig. 10: **PhotoMap: Unbiased mapping of expansion deformation field.**
a, Schematic of fluorescent monomer. A fluorescent dye is covalently conjugated to the gel monomer, rendering the gel intrinsically fluorescent.
b, Grid photobleaching. Using a two-photon microscope, a three-dimensional grid ($50 \times 50 \times 50$ μm) was photobleached into both the sample and the surrounding gel. *Left*: whole-gel image with the grid visible. *Right*: example crops showing regions containing no sample, brain tissue, and the cleithrum, the earliest mineralizing bone in zebrafish[16].
c, Visualization of deformation after expansion. Same views as in **b** imaged after tissue disruption and gel expansion.
d, Extraction of the deformation field. Grid intersections were algorithmically detected throughout the sample. Same views as in **b**, with intersection points color-coded by their deviation from the ideal 90° intersection angle.
e, Quantification of isotropy. For each grid intersection, the mean deviation from the target length L to its four nearest neighbors was measured, along with the deviation from the ideal 90° intersection angle.
f, Deformation field comparison. Distributions of length deviation (*left*) and angle deviation (*right*) measured in off-sample (blue) and on-sample (orange) regions. On-sample distributions are broader but remain within a relatively narrow range.

We thank the reviewer for this comment and the opportunity to develop this method for quantifying distortions.

Minor Comments (Reviewer #3)

Reviewer Comment:

The authors have demonstrated a cell segmentation method based on spontaneous calcium dynamics and spectral clustering. But does it work in all organ systems? It seems that validation through traditional imaging is inconsistent throughout the paper. It is also concerning that there could be crosstalk from neighboring cells, especially in one-photon imaging, resulting in artifactual correlation of neural activities. Could calcium dynamics across different cell types lead to contamination of signals, impeding the accuracy of spectral clustering relying on spontaneous activities? More validation experiments would clarify and support the proposed segmentation method.

Our Response:

We thank the reviewer for these thoughtful questions about signal crosstalk and segmentation reliability. We have carefully considered the suggestion for additional validation experiments. After reflecting on this, we have performed new characterization of the spatial resolution across the sample and we believe the combination of our methodological approach and existing validations address these concerns. We explain our reasoning below.

Addressing crosstalk through microscopy and analysis: The concern about crosstalk is important. We chose spinning-disk confocal microscopy specifically because it rejects scattered light more effectively than light-sheet approaches. For segmentation, we used constrained non-negative matrix factorization (NMF) - a standard method in volumetric calcium imaging[17] - which explicitly models camera pixels as receiving photons from multiple overlapping sources and performs linear source separation. Regarding whether this faithfully captures signals from individual cells, we first performed a new analysis of spatial resolution, and then consider case studies of specific cell types as a validation of how NMF reaches the same conclusions as cell-type specific studies.

New analysis - spatial resolution characterization: To better characterize our achievable resolution, which would limit subsequent segmentation, we performed Fourier Ring Correlation[13] (FRC) analysis across the sample (Extended Data Figure 1e). This shows we reliably resolve $\sim$5 μm objects (cell-sized) across most of the imaged volume, with expected degradation in high-scattering regions. Our segmentation conservatively extracts "cellular segments" rather than claiming complete cell boundaries - we have further emphasized this in the text. We note that NMF can, in principle, outperform the resolution reported by FRC, because NMF makes use of temporal fluctuations.

Existing experimental evidence: Regarding the concern that the extracted timeseries may mix GCaMP signal from nearby cells, we took findings from imaging the densely-labeled tissues and tested

them in cell type-specific transgenic lines. These experiments were reported in the previous, submitted version of the manuscript, but we thought it would be useful to summarize them here, as well as discuss the implications of these results in the context of the reviewer's concern. To summarize, we performed independent validations for our key biological findings to confirm that signals extracted from dense imaging could be independently identified in sparse transgenic lines in order to confirm that these were not the result of demixing artifacts, as we, like the reviewer, also wanted this confirmation:

- Motor-coupled ependymal cell dynamics: We identified motor-coupled ependymal cell dynamics in WHOLISTIC data (Figure 2f-h), then confirmed this in a cell-type-specific line (Tg(foxj1a:GCaMP7f)) generated for this purpose, recovering identical dynamics and ruling out that the results arose from demixing artifacts (Figure 2i-k).
- State-dependent oscillations: Oscillatory dynamics extracted from dense imaging (Figure 3a, Extended Data Figure 8a-d) were independently validated in the sparse ependymal-specific line (Figure 3j).
- Sympathetic ganglia: Cells identified within the sympathetic ganglion in WHOLISTIC data showed hypoxia responses (Figure 4k-l). We confirmed this finding in a sympathetic-specific line (Tg(th:gal4;UAS:GCaMP6f)) showing similar dynamics (Figure 4m).

These validations span different anatomical regions and cell types. We focused validation efforts on key biological findings rather than attempting to validate segmentation in every organ system, as the latter would require generating dozens of cell-type-specific lines. We recognize this represents a methodological choice, and that more comprehensive validation across all tissues would strengthen confidence. However, we believe the convergence of appropriate microscopy, standard algorithms that have appropriate assumptions, quantified spatial resolution, and multiple independent biological validations provides reasonable evidence for reliable signal extraction from our segmentation approach.

We have also added an acknowledgment in the Supplementary Notes (Future Extensions section) that some degree of signal mixing cannot be completely eliminated with any dense volumetric imaging method, which motivated our strategy of independent validation for key findings. In this context, we have also added a discussion in the Future Extensions section of the Supplementary Notes about how the cell type-specific experiments provide a degree of validation of the signal extraction procedure, while acknowledging that additional validation across the body, for example by co-expressing a mosaic, randomly expressed calcium indicator of a different color and analyzing the dense and randomly-sparse datasets for correspondence, would be a desirable future direction. We have copied this discussion below (line 2043):

"Regarding cellular-scale segmentation, while NMF-based source separation is a standard approach in volumetric calcium imaging and substantially reduces signal mixing, complete crosstalk elimination can never be fully guaranteed. This motivated our validation strategy of independently confirming key biological findings using cell-type-specific transgenic lines (e.g., ependymal cells - Figure 2i-k, Figure 3j, sympathetic neurons - Figure 4k-l). A more comprehensive validation approach for future work would be to co-express a randomly sparse, mosaic calcium indicator of a different color and analyze correspondence between dense and sparse datasets across all tissue types."

Below is the new Extended Data Figure mentioned quantifying spatial resolution:

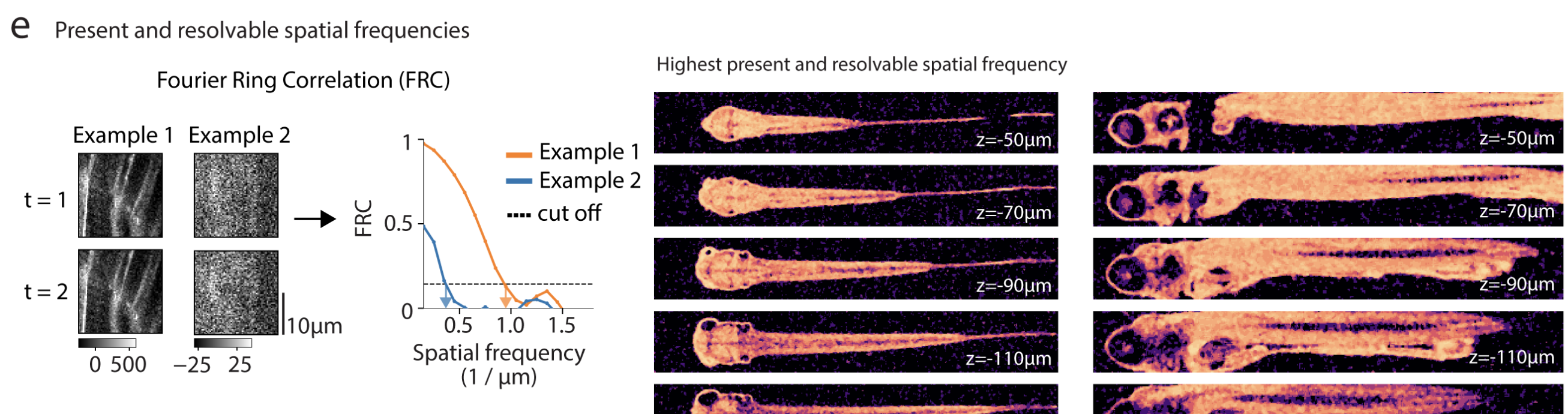

$$\mathrm{FRC}(f_\ell) = \frac{\sum_{(f_x,f_y)\in f_\ell} \mathrm{Re}\Big[F_1(f_x,f_y)\,F_2^*(f_x,f_y)\Big]}{\sqrt{\Big(\sum_{(f_x,f_y)\in\mathcal{R}_\ell} |F_1(f_x,f_y)|^2\Big)\Big(\sum_{(f_x,f_y)\in\mathcal{R}_\ell} |F_2(f_x,f_y)|^2\Big)}}$$

Extended Data Fig. 1: **WHOLISTIC transgenics and workflow.**
e, Present and resolvable frequencies across the sample. Left: schematic of the Fourier Ring Correlation (FRC) method for quantifying the reliably resolvable spatial frequencies within an image. Two independent images of the same object are acquired, their 2D Fourier transforms computed, and the phase correlation calculated, radially averaged, and normalized. The spatial frequency at which the correlation falls below 1/7 is taken as the resolution cut-off[13]. Right: spatial map of the highest spatial frequency at which the FRC remains above the 1/7 threshold. High spatial frequencies are detectable across most of the imaged volume, with localized reductions in regions between the ear and in deep pharyngeal areas between the gills.

Reviewer Comment:

2. Calcium dynamics can be very fast. The use of a spinning disk confocal microscope for imaging at a volumetric rate of 0.3 Hz is well below such speeds in the literature. The slow volumetric rate might lead to missing of much calcium activity, and thus omitted signals or organs. Re-imaging subsets at higher speeds could validate the claims of the paper, and make sure that there are not higher frequency signals that are missing.

Our Response:

We thank the reviewer for this important concern about temporal resolution. We agree that 0.3 Hz volumetric imaging is slow and that faster imaging would be beneficial. To address this, we performed

faster imaging on subvolumes and single planes at rates up to ~6 Hz and developed a new analysis method to quantify the signal content captured at different across different temporal frequencies.

To systematically assess which tissues require faster imaging, we developed Fourier Temporal Correlation (FTC), a method that quantifies the reliable temporal frequency content in timeseries (Extended Data Figure 13a-b). This analysis reveals that neurons and muscles exhibit some reliable signals above 1 Hz (thus benefiting from faster imaging), nonetheless the most reliable signal occurs at frequencies below 0.3 Hz and would be captured by the current imaging rates. Visceral organs like the kidney show primarily low-frequency signals that are well-captured at 0.3 Hz sampling rate. This provides a quantitative, tissue-specific answer to the question "how fast should we image?"

We would like to emphasize that the current imaging rates are not a fundamental limit and that relatively-straightforward microscope optimization, such as dual-plane imaging approaches enabled by the 2P-RAM mesoscope[8] available from Thorlabs, or the Light Beads Microscope[18], allow for faster imaging. As the first report about WHOLISTIC, we did not push in this direction and focused on providing a comprehensive set of tools. We have now added a discussion of avenues for faster imaging systems in the Future Extensions section of the Supplementary Notes (line 2025):

> *"In this instantiation, a) samples are embedded in agarose which limits their movement; b) moreover, to best image the viscera, fish are placed on their sides which is not their natural posture; c) furthermore, spinning-disk confocal microscopy was used which induces more bleaching than light-sheet microscopy or 2-photon microscopy and is relatively slow, and d) finally, with the main objective used in this study, 20×0.75NA, the field of view was restricted to 800 μm ×800 μm. To overcome this, we foresee using a large field of view 2-photon microscope[8] and positioning a 45º mirror next to the fish, making it possible to image the entire length of the fish, positioned in its natural posture, whilst providing access to both the brain and viscera (via the mirror) simultaneously. Such a microscope also includes a resonant scanner and offers the capacity to do dual plane imaging, which would double the frame rate; faster imaging rates could also be achieved through the use of multi-beam optics[19], or Light Beads microscopy[18]."*

In the current work, the 0.3 Hz rate represents a practical trade-off. Standard light-sheet microscopes that enable faster volumetric imaging suffer from degradation of the light-sheet due to scattering and refraction in heterogeneous tissue, exciting fluorescence outside the focal plane, and the wide-field detection of such systems fails to reject this out-of-focus and scattered emission, resulting in poor image quality for whole-body imaging. Spinning-disk confocal microscopy provides the optical sectioning needed for reliable whole-body imaging but is slower. We acknowledge this is a limitation of the current platform — future iterations will focus on increasing speed while maintaining optical quality.

In summary, we agree with the reviewer that faster imaging would be valuable and have demonstrated this through direct comparisons at higher frame rates. The FTC analysis provides a framework for users to assess what is the sampling rates are best suited for specific tissues of interest. We present the new Extended Data Figure below:

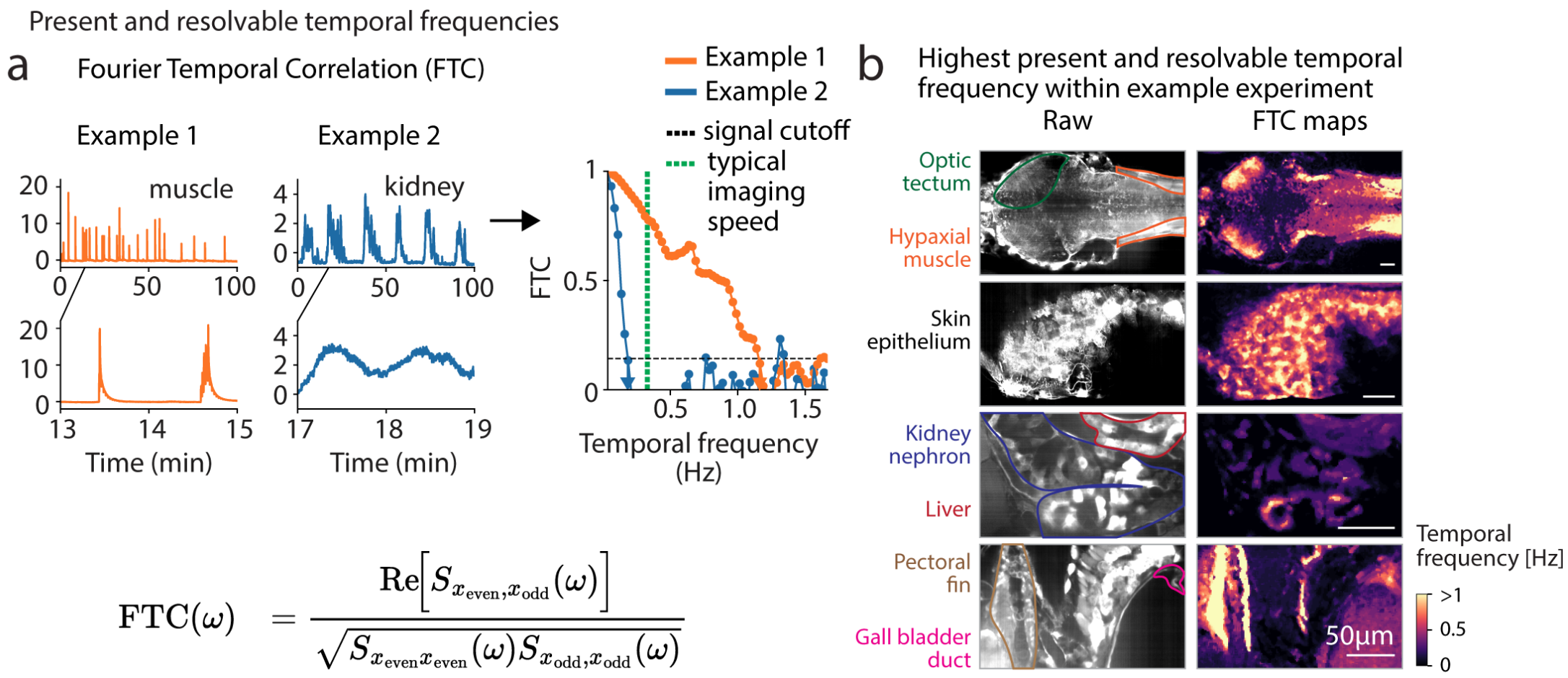

Extended Data Fig. 13: **Characterization of GCaMP signal statistics.**
a, Schematic of the Fourier Temporal Correlation (FTC) method for quantifying the reliably resolvable temporal frequencies within a timeseries. Alternating timepoints are used as independent measurements of the same underlying process, their cross-spectrum is computed, and the phase correlation calculated. The temporal frequency at which the correlation falls below 1/7 is taken as the resolution cut-off (can be adjusted as desired). This provides an estimate of the minimum imaging speed required to capture temporal dynamics in different tissues without losing information. The vertical dashed lines indicates the typical imaging speed used in this manuscript.
b, Spatial maps of the highest temporal frequency at which the FTC score remained above the signal threshold (user adjustable). The scores were computed on ∼45 min single plane higher-speed (>6Hz) datasets. We note that this provides a lower bound: organs may exhibit higher frequencies that can be resolved but that were not observed in the experiment. Neurons and muscles (orange trace in a) exhibited as expected high-frequency activity, whilst the kidney (blue trace in a) only had resolvable power in the lower temporal frequencies.

Reviewer Comment:

3. In Fig. 2 f-g, a tissue-specific kernel was applied for the regression model. The selection process and criteria for those kernels could be better described.

Our Response:

Thank you for pointing this out. We have expanded the description of the model as well as the fitting process for the tissue-specific kernels which itself determines what the kernel is, in the Methods section. We have added the following description (line 891):

"A lag-regression model is a predictive model for time-series data in which a regression equation is used to predict the current value of the dependent variable, $y(t)$, based on on both the current, and past (i.e.: lagged), values of an explanatory variable, $x(t), x(t-1), ..., x(t-L)$ where L is the maximum number of lags. This is the equivalent to assuming that the observed dependent variable

arises from the convolution of the independent variable with a learnt kernel, which is parametrized by a set of weights, one for each lag:

$$y(t) = \sum_{m=1}^{M} \sum_{\tau=0}^{L} x_m(t-\tau) \cdot k_m(\tau) \tag{1}$$

where M is the number of regressors/independent variables, and L is the maximum number of lags. The kernel parameters (k_m) are fit to minimize the mean squared reconstruction error (that is: $\|y - \bar{y}\|_2^2$ where y is a vector containing the observations, and $\bar{y}$ is the model prediction), by finding the least squares solution using the numpy.linalg.solve function. This approach allows each tissue to have different temporal response properties (e.g., neurons show fast responses while other tissues tend to show slower, more prolonged responses to motor activity).

The mean activity of the muscle hyper-clusters (grouped clusters/cellular ensembles) were converted into a weighted binary trace by identifying the onset of motor contraction and weighting them by the power of the contraction. Contraction onset and offset were identified by finding positive deflections above the noise floor and the time at which the curve returned to the noise floor; power was defined by the area under the curve of these two time points. For each cell $y_i \in Y$, a lag regression model is fit, $y_i(t) = \sum_{m=1}^{M} \sum_{\tau=0}^{L} x_m(t-\tau) \cdot k_m(\tau)$ where M is the number of muscle regressors included (4 to 6), L the number of time lags included (60 seconds), $y_i(t)$ and $x_m(t)$ is the activity of a cell i (y_i), and muscle regressor m x_m), at time t. A time lag of 60 seconds was chosen, as we wanted to capture relatively short-lived responses. The identified kernel values are 3-fold cross-validated and the average R^2 value on held-out data is reported."

Reviewer Comment:

4. The use of various fish lines is confusing to read. Adding a table to list the names of the lines and where they are used would be helpful.

Our Response:

Thank you for the helpful suggestion. We have added a new table in the Supplementary Notes, also included below, where we list all the transgenic lines used, including a description of their expression pattern, along with relevant references (line 2057).

Table of transgenic lines generated and used

Transgenic Line	Target Cell Population	Protein Function	Source	Species	References
Tg(ubi:tTA; TRE:jRGECO1b)	Pancellular	Red calcium indicator	generated	Danio rerio	Mosimann et al. (2011) | Dana et al. (2016)
Tg(ubi:tTA); Tg(TRE: GCaMP7f)	Pancellular	Green calcium indicator	generated	Danio rerio	Mosimann et al. (2011) | Dana et al. (2019)
Tg(ubbR:jGCaMP8m)	Pancellular	Green calcium indicator	generated	Danio rerio	Bakunaite et al. (2024) | Zhang et al. (2023)
Tg(foxj1a:GCaMP7f)	Motile ciliated cells (ependymal cells)	Green calcium indicator	generated	Danio rerio	Zhang et al. (2020) | Dana et al. (2019)
Tg(elavl3:gtACR2-eYFP)	Panneuronal	Inhibitory opsin & yellow fluorescent marker	generated	Danio rerio	Park et al. (2000) | Mohammad et al. (2017)
Tg(β-actin2:mCherry-CAAX; myl7:GFP)	Pancellular	Red membrane marker & Green cardiac marker	generated	Danio rerio	Fowler et al. (2016)
Tg(VAChTa:Gal4); Tg(UAS: CoChR-eGFP)jf44	Cholinergic neurons (motor neurons)	Excitatory opsin & green fluorescent marker		Danio rerio	Taniguchi et al. (2017) | Mu et al. (2019)
Tg(th:Gal4); Tg(UAS: GCaMP6f)jf46	Catecholaminergic neurons (sympathetic ganglia)	Green calcium indicator		Danio rerio	Li et al. (2015) | Mu et al. (2019)
Tg(flk1:dsRED-CAAX)	Vascular endothelial cells	Red membrane marker		Danio rerio	Fujita et al. (2011)
Tg(gata1:dsRED)	Erythrocytes	Red fluorescent marker		Danio rerio	Traver et al. (2003)
Tg(isl1CREST-hsp70l: mRFP)	Motor neurons	Red fluorescent marker		Danio rerio	Grant et al. (2010)
Tg(phox2bb:eGFP)	Autonomic nervous system	Green fluorescent marker		Danio rerio	Nechiporuk et al. (2007)
Tg(foxj1a:eGFP)	Motile ciliated cells (ependymal cells)	Green fluorescent marker		Danio rerio	Grimes et al. (2016)
Tg(gfap:jRGECO1b)	Glia	Red calcium indicator		Danio rerio	Mu et al. (2019)
Tg(elavl3:H2B-jRGECO1a)	Neuronal	Nuclear localized red calcium inidcator		Danio rerio	Yang et al. (2022)
Tg(ubbR:jGCaMP8m)	Pancellular	Green calcium indicator	generated	Danionella cerebrum	Bakunaite et al. (2024) | Zhang et al. (2023)
TgBAC(lamC1:lamC1-sfGFP)	Basement membrane	Green fluorescent marker		Danio rerio	Yamaguchi, N., Zhang, Z., Schneider, T. et al. Rear traction forces drive adherent tissue migration in vivo. Nat Cell Biol 24, 194–204 (2022). https://doi.org/10.1038/s41556-022-00844-9
mitfa(-/-)	Melanocytes	Missense mutant	generated	Danionella cerebrum	Schulze et al. (2018)

Reviewer Comment:

5. In Figure 2n, a color map is used to show the different traces, corresponding to a range of optogenetic stimulation intensities; however, the scale of the stimulation and how it maps to the colors is not provided in the figure.

Our Response:

We apologize for this oversight and thank the reviewer for spotting this. The legend for Figure 2n has been updated to include a color bar and corresponding scale bar for the optogenetic stimulation duration; the stimulus light intensity was kept constant. Below is the updated figure:

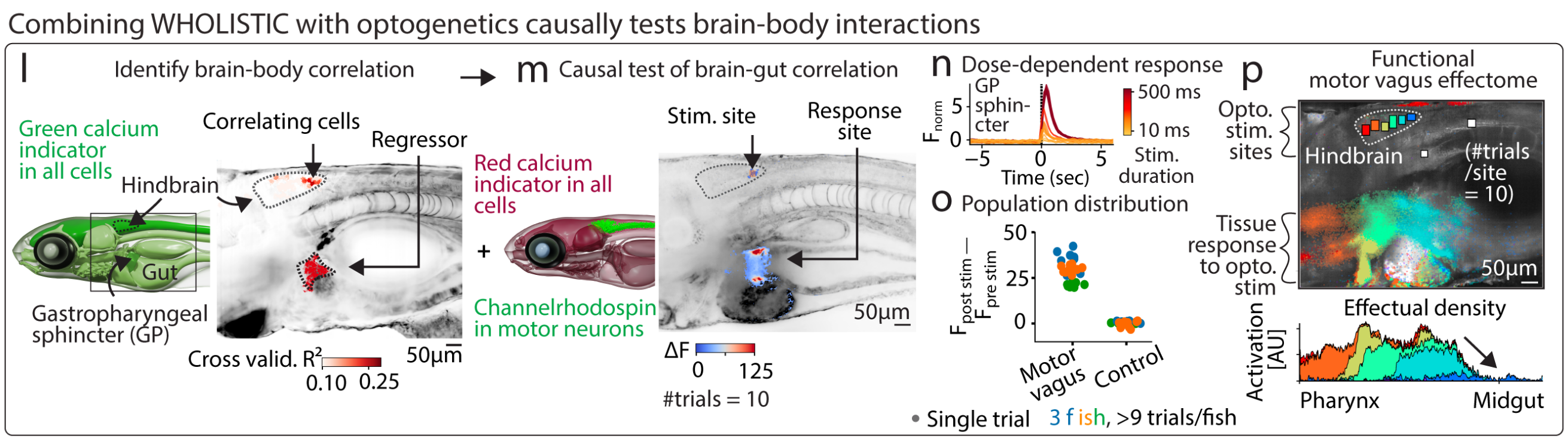

Fig. 13: **Identification and causal test of intra- and inter-organ coupling through WHOLISTIC.**
l, *Left*: schematic outline of the location of the hindbrain and the gastropharyngeal (GP) sphincter. *Right*: a small group of cells located in the hindbrain have high loadings on the GP sphincter regressor. Maximum projection of brain, alpha-weighted by the variance explained by sphincter calcium activity (R^2) in held-out data, overlaid onto anatomical reference (gray), with location of GP sphincter functional ensemble shown in flat red.
m, *Left*: genetic strategy for optogenetic stimulation of motor vagal cells during WHOLISTIC imaging. *Right*: stimulus-triggered average responses overlaid with anatomical reference (# trials = 10, where # stands for 'number' throughout); stimulation site and full motor vagus outline indicated.
n, Gastric sphincter calcium activity showing dose-dependent response to optogenetic activation of caudal motor vagal neurons.
o, Quantification of change in fluorescence at the gastric sphincter following stimulation of caudal motor vagus or control location along the spinal cord (3 animals, >9 trials/animal).
p, *Top*: optogenetic tiling across the motor vagus to assess the functional topological relationship between motor vagus and visceral organs. Trial-triggered average response of motor vagal nuclei stimulation sites, pixels weighted and color coded according to the ROI (region of interest) inducing the most activity (# trials/location = 10), overlaid with anatomical reference (gray). *Bottom*: plot of responsive tissue volume (activation density) along the anterior-posterior axis of the gastrointestinal tract.

Reviewer #3 (Remarks on code availability)

Reviewer Comment:

Reviewing the code is beyond our capability, and expertise, alas.

Reviewer #4 (Remarks to the Author)

Reviewer Comment:

The paper by Ruetten et al. images calcium dynamics across the whole body of larval zebrafish, using an approach the authors call WHOLISTIC. The study is impressive in its scope and the number of techniques the authors present:

- Imaging calcium dynamics not only in the brain, but in many other tissues too.
- Improved expansion microscopy protocols.
- Improved analysis of spatiotemporal calcium signals (coherence-based spectral clustering).
- A slightly novel motion correction algorithm.

None of these are innovations per-se, although together they do form a very interesting method and approach that is indeed novel.

In fact, that is my main concern with the paper, that it seems to be more of a methods paper. It is difficult for me to state the key results, beyond the method. As the authors themselves state:

“Collectively, these findings demonstrate how the ubiquitous expression of molecular sensors, in conjunction with advanced motion-corrected spinning-disk confocal microscopy and the computational workflow for cellular and tissue segmentation, together comprising WHOLISTIC, give access to a rich array of cellular dynamics across tissues of the body, thereby facilitating the study of their interactions.”

and they refer to WHOLISTIC as a methodology (line 465). As the abstract states: the authors ”advance and integrate” already existing methods and I am not sure what the main scientific result of the study is.

If my understanding is correct, fish are mounted on the side (unlike what is shown in Fig 1a), and a field of view of 800 x 600 x 130 microns is recorded shown in Figure 1e. It is important to highlight that this is the field of view, and not Fig 1b or 1c. Then confocal microscopy is used at a rate of 0.3 Hz. In terms of imaging this is not groundbreaking: the Ahrens Lab has outdone this by an order of magnitude. There are many reasons why confocal microscopy is not used typically in in-vivo studies and in this instance the whole tissue will be constantly irradiated with excitation light.

Personally, if this was to be viewed as a methods paper, I would find it more useful if the improvements mentioned above were more carefully quantified. How much better is the motion correction methods used compared to current methods? How does the coherence-based spectral clustering compare to correlation based clustering? Can the authors plot for example a measure of cluster/eigenmode size as a function of cluster/eigenmode number to backup their argument about compactness of the spatial ROIs of the first method vs. the second? Currently, the data presented is a very particular example shown in Ext. Data Fig 4 d-f and there is no quantification of the method in general. Similarly, for the statements made in Ext. Data Fig 4 a-c regarding interpretability of the clusters: the authors

themselves mention that the results depended on the number of clusters/eigenmodes, so the reader is left wondering how much better are these methods really. I do not doubt that they are, I do think though that this has to be unequivocally shown, in order to truly appreciate the improvements presented by WHOLISTIC. This is a general point: the methods are reasonably well presented, but they are not appropriately benchmarked with the existing state-of-the-art approaches.

Our Response:

We thank the reviewer for their thoughtful assessment and constructive suggestions regarding further method characterization. The reviewer's suggestions have substantially improved the paper by prompting us to perform more rigorous benchmarking and quantitative characterization.

Regarding the main message: We agree that WHOLISTIC integrates multiple techniques, though many of these we also improve upon (e.g.,: registration, expansion microscopy); furthermore, we believe that this integration of components enables a new approach to studying multi-tissue/organ coordination that wasn't possible before. The key contributions of the paper are threefold:

1. First, we demonstrate that simultaneous cellular-resolution recording across multiple organ systems in a living vertebrate is now feasible;
2. Second, we provide a complimentary set of new transgenic lines, protocols and analysis pipelines that enable a) WHOLISTIC imaging, b) processing of the datasets and their transformation into interpretable time-series, c) a means of contextualizing findings with high-resolution anatomical and molecular data provided by our new Whole Body Expansion Microscopy protocol
3. Third, we show that this capability enables discovery of previously inaccessible inter-organ coordination phenomena that would be difficult or impossible to uncover with traditional single-tissue approaches. The specific biological discoveries — muscle coordination patterns, motor-quiescence coupled ependymal oscillations, and brain-controlled blood flow redistribution during hypoxia — serve as proof-of-principle demonstrations that whole-body imaging can reveal numerous physiological properties.

We welcome the constructive comments of the reviewer and have conducted extensive additional analyses and collected new data to further characterize our methodology.

Motion correction benchmarking (NEW - Extended Data Figure 2c): Following the reviewer's suggestion to quantify how much better our registration is, we have compared and benchmarked our registration method against existing registration methods commonly used in the field and added this work to the supplementary figures (Extended Data Figure 2c). We generated synthetic datasets by applying known motion fields to experimental images, varying displacement amplitude, spatial scale,

and noise levels. We then compared five registration methods: WHOLISTIC registration, B-spline, Demons, NoRMCorre, Suite2p, and WHOLISTIC registration without the multiscale pyramid scheme. For each method we computed both image-based metrics (mean squared error of registered images) and motion-field accuracy (MSE of recovered versus ground-truth displacement fields). This latter metric approach prevents rewarding methods that achieve good image similarity through incorrect deformation fields (overfitting). We find that WHOLISTIC registration outperforms existing methods, with the pyramid approach being a critical component.

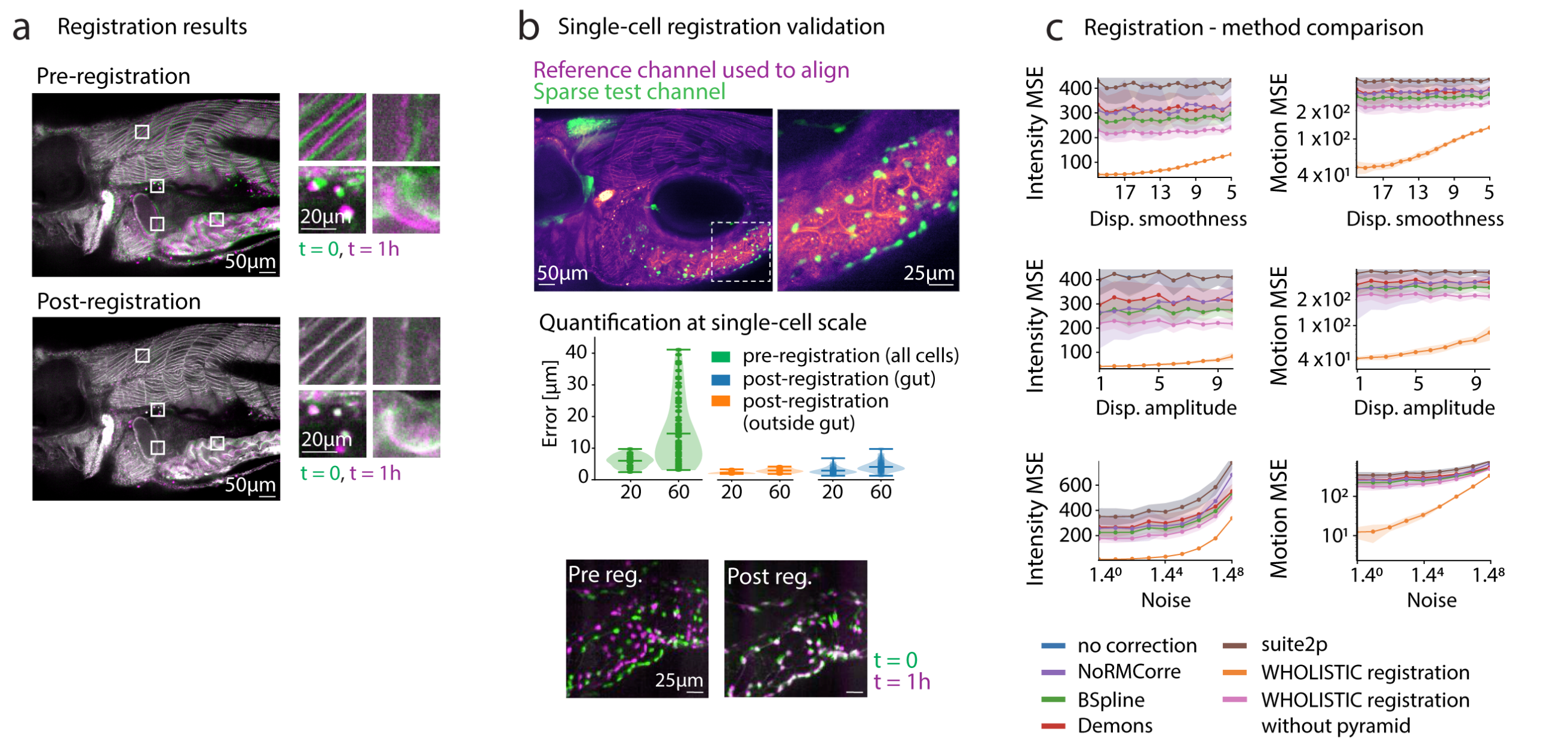

Extended Data Fig. 2: **WHOLISTIC registration.**
a, Registration results. Overlay of data from the start of an experiment (green) and 1 hour into recording (magenta). *Top left*: pre-registration overlay showing significant drift. *Bottom left*: post-registration overlay showing alignment correction in the same regions and at the same timepoints. *Right*: enlarged views of muscle, gallbladder, skin, and gut regions pre- and post-registration.
b, Validation of registration results. *Top left*: double transgenic line used for validation (*Tg(β-actin:mCherry-CAAX; phox2bb:eGFP)*) with inset showing enlarged view of the gut – the most challenging region to register. *Top right*: overlay of sparse cell line at the start of the experiment (green) and 1 hour in (magenta), pre-registration (left) and post-registration (right). *Bottom*: violin plots of cellular displacement after 20 and 60 minutes: pre-registration displacement (green), post-registration displacement in gut (blue), and in the rest of the viscera and brain (orange).
c, Registration method comparison. Synthetic datasets were generated by applying known motion fields to experimental images, varying displacement radius, amplitude, and noise to test robustness. Each dataset was registered with six methods: WHOLISTIC registration, WHOLISTIC registration without pyramid scheme, B-spline, Demons, NoRMCorre, Suite2p. Mean squared error (MSE) between referenced and registered pixel values was computed, as well as MSE of the motion vector field to penalize potential over-fitting.

Spatial resolution quantification (NEW - Extended Data Figures 1e, 9d): We have characterized the present and resolvable spatial frequencies across the sample both in functional data (Extended Data Figure 1e) and expanded data (Extended Data Figure 9d) using a method from the field of super-resolution microscopy: Fourier Ring Correlation[13]. This analysis shows we reliably resolve ∼5 μm features (cell-sized) across most of the imaged volume, with reduced resolution in limited regions (ears, eyes, and regions posterior to them where light scattering is higher). In expanded samples, we find that

there is minimal degradation of resolution with depth, confirming excellent clearing of the method.

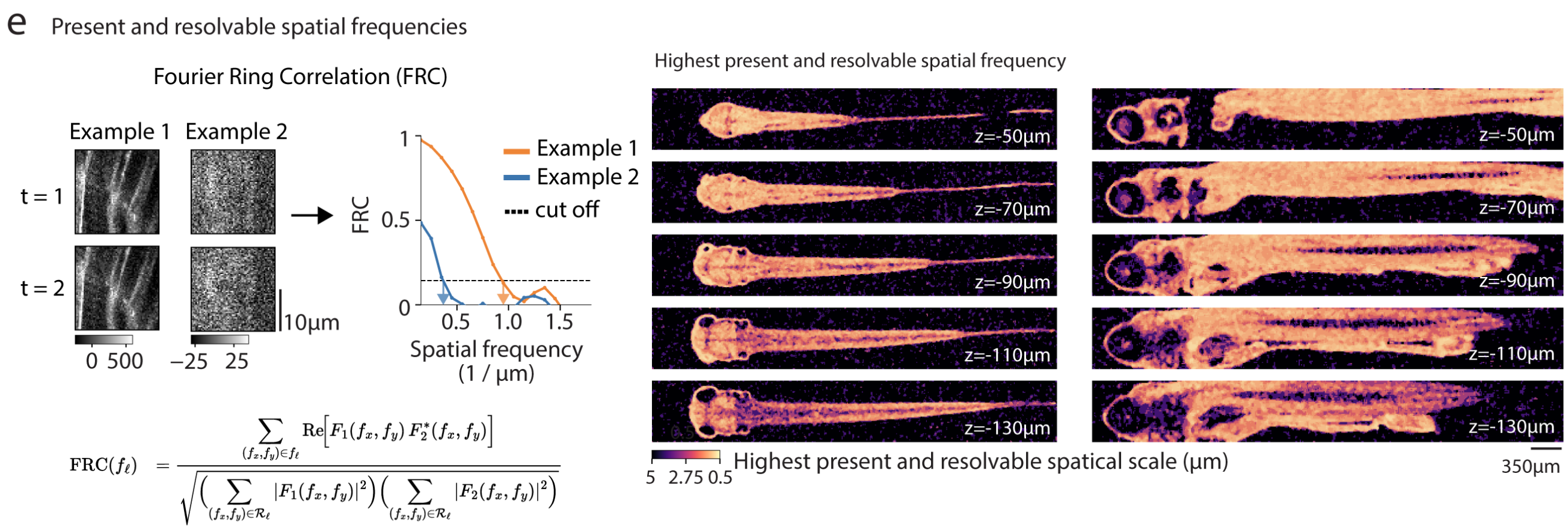

Extended Data Fig. 1: **WHOLISTIC transgenics and workflow.**
e, Present and resolvable frequencies across the sample. Left: schematic of the Fourier Ring Correlation (FRC) method for quantifying the reliably resolvable spatial frequencies within an image. Two independent images of the same object are acquired, their 2D Fourier transforms computed, and the phase correlation calculated, radially averaged, and normalized. The spatial frequency at which the correlation falls below 1/7 is taken as the resolution cut-off[13] (user adjustable). Right: spatial map of the highest spatial frequency at which the FRC remains above the 1/7 threshold. High spatial frequencies are detectable across most of the imaged volume, with localized reductions in regions between the ear and in deep pharyngeal areas between the gills.

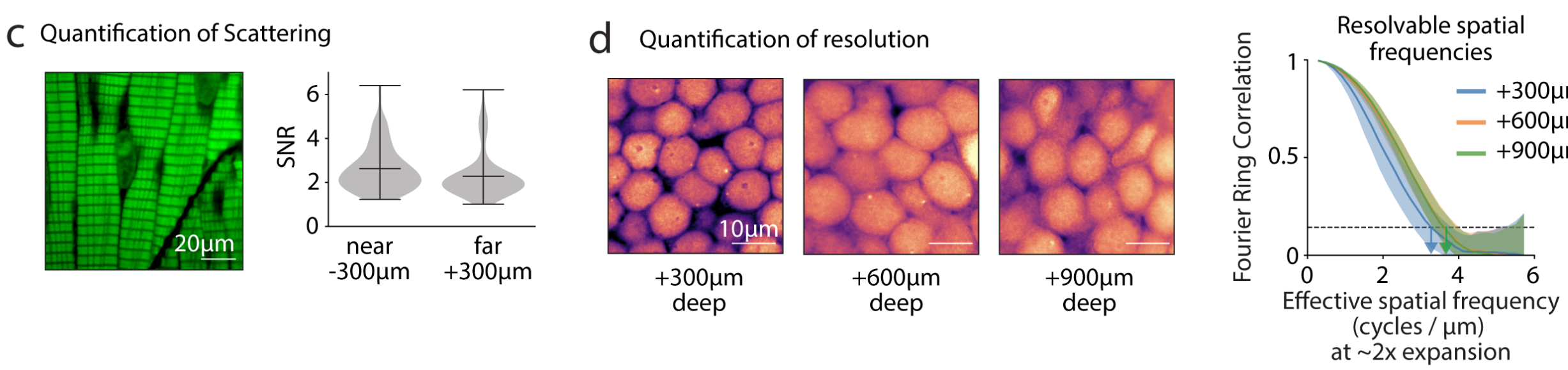

Extended Data Fig. 9: **Whole-Body Expansion Microscopy (WB-ExM) to map ultrastructure, RNA and protein across the body.**
c, Quantification of clearing quality of WB-ExM sample. *Top*: Image of muscle sarcomeres in WB-ExM sample stained with total protein stain (Alexa488-NHS). *Bottom*: Distribution of signal to noise ratio (peak to trough) in sarcomeres at far and near side of the sample.
d, Quantification of resolvable spatial frequencies in WB-ExM sample. *Left*: Image of neurons located at different depths within the brain, stained with total protein stain (Alexa488-NHS). *Right*: Fourier ring correlation of WB-ExM data at different depths showing little attenuation in resolvable frequencies. The spatial frequency at which the correlation falls below 1/7 is taken as the resolution cut-off[13].

Expansion microscopy isotropy quantification (NEW - Extended Data Figure 10): We developed PhotoMap, a new method that photobleaches a regular grid into samples that have been embedded in a gel engineered to be be intrinsically fluorescent, enabling quantification of geometric distortions post-expansion across the samples. This provides unbiased, sample-wide assessment of expansion uniformity, showing largely uniform expansion except in mineralizing bone regions, where the deformation nonetheless remains local. We provide a comprehensive step-by-step protocol in the Supplementary Notes to enable reproduction of this method.

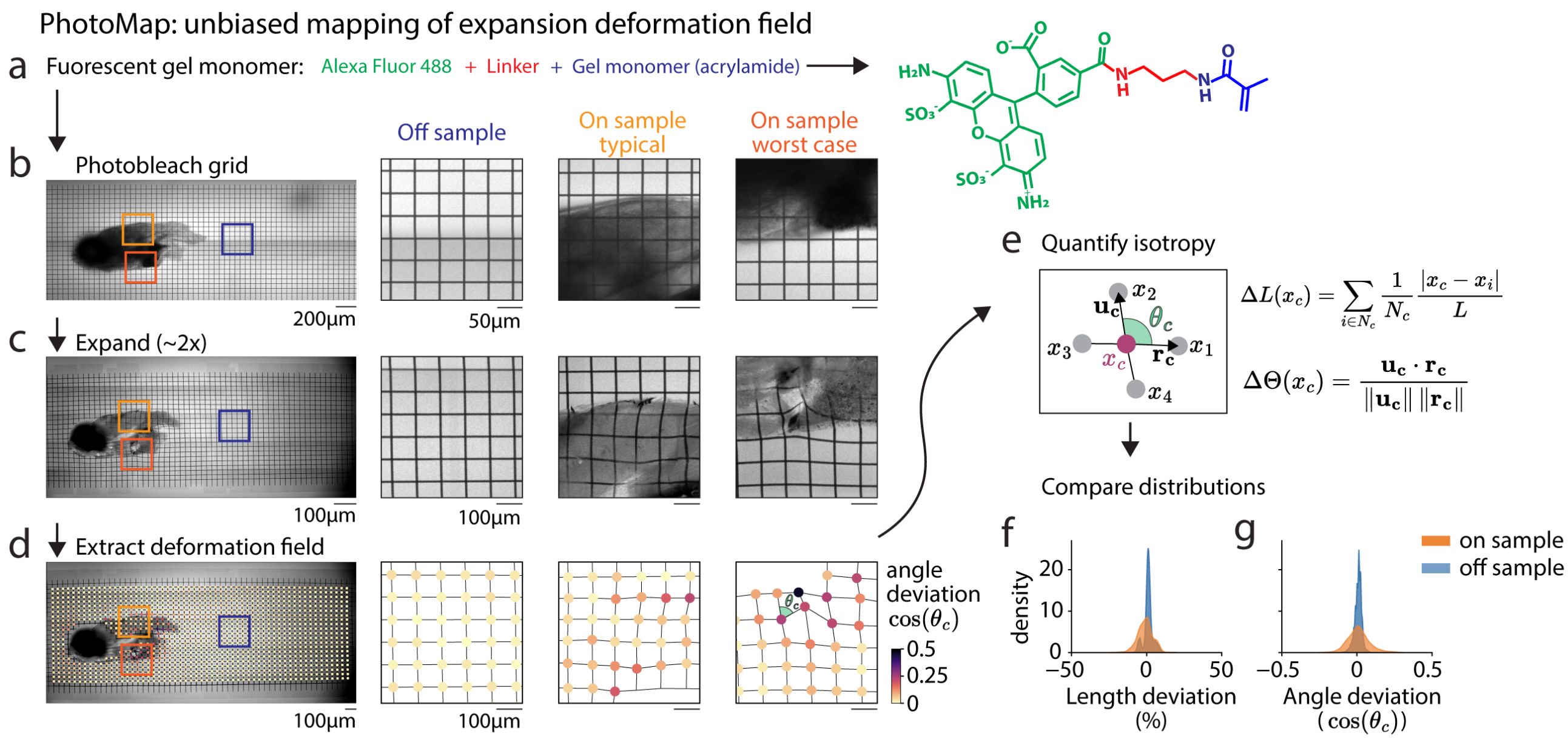

Extended Data Fig. 10: **PhotoMap: Unbiased mapping of expansion deformation field.**
a, Schematic of fluorescent monomer. A fluorescent dye is covalently conjugated to the gel monomer, rendering the gel intrinsically fluorescent.
b, Grid photobleaching. Using a two-photon microscope, a three-dimensional grid ($50 \times 50 \times 50\ \mu$m) was photobleached into both the sample and the surrounding gel. *Left*: whole-gel image with the grid visible. *Right*: example crops showing regions containing no sample, brain tissue, and the cleithrum, the earliest mineralizing bone in zebrafish[16].
c, Visualization of deformation after expansion. Same views as in **b** imaged after tissue disruption and gel expansion.
d, Extraction of the deformation field. Grid intersections were detected throughout the sample. Same views as in **b**, with intersection points color-coded by their deviation from the ideal 90° intersection angle.
e, Quantification of isotropy. For each grid intersection, the mean deviation from the target length L to its four nearest neighbors was measured, along with the deviation from the ideal 90° intersection angle.
f, Deformation field comparison. Distributions of length deviation (*left*) and angle deviation (*right*) measured in off-sample (blue) and on-sample (orange) regions. On-sample distributions are broader but remain within a relatively narrow range.

Temporal frequency characterization (NEW - Extended Data Figure 13): Regarding the imaging speed and field of view, we agree that 0.3 Hz is not groundbreaking and that the Ahrens lab has achieved faster imaging rates in the brain. However, the system uses light-sheet microscopy which, while faster, suffers from degradation of the light-sheet due to scattering and refraction in heterogeneous tissue, exciting fluorescence outside the focal plane. Moreover, the wide-field detection path of most light-sheet systems fails to reject this out-of-focus and scattered emission, resulting in poor image quality for whole-body samples. We tested multiple types of light-sheet system (SimView[20], Luxendo MuVi-SPIM with electronic confocal slit detection[21] and Ahrens lab model) but found none of them to be adequate. Our use of spinning-disk confocal microscopy was a deliberate trade-off: sacrificing speed to achieve the optical sectioning necessary for reliable signal extraction across the heterogeneous body. To quantitatively assess how fast one should image the sample, given that tissues display different spectra, we derived a method to quantify the present and resolvable temporal frequencies present in a standard experiment by generalizing Fourier Ring Correlation to the time domain (Extended Data Figure 13). While this doesn't address the relatively slow imaging rates, this provides a quantification of signal

distribution and future iterations will aim to improve imaging speed while maintaining optical quality, potentially through advanced 2-photon designs, which we mention in the Discussion section, and further discuss in detail in the Future Extensions section of the Supplementary Notes.

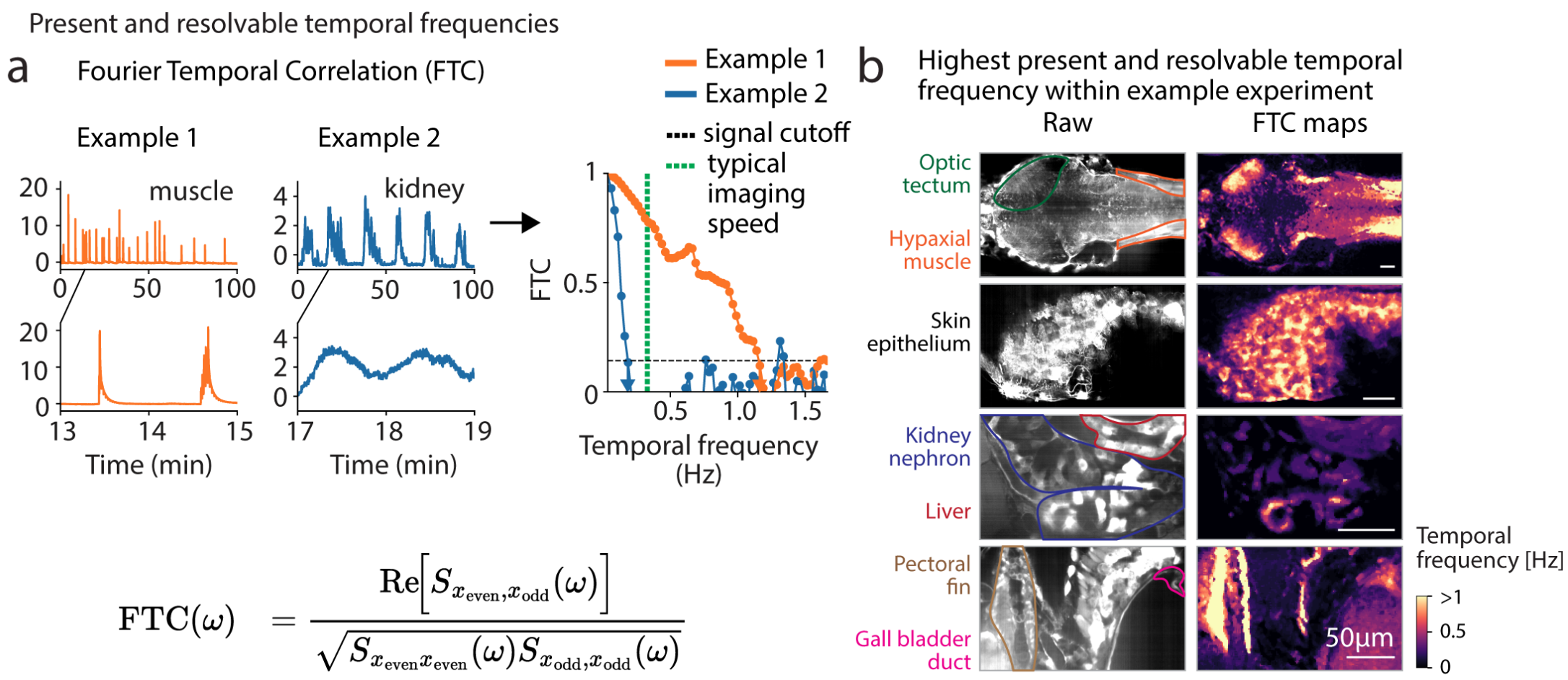

Extended Data Fig. 13: **Characterization of GCaMP signal statistics.**
a, Schematic of the Fourier Temporal Correlation (FTC) method for quantifying the reliably resolvable temporal frequencies within a timeseries. Alternating timepoints are used as independent measurements of the same underlying process, their cross-spectrum is computed, and the phase correlation calculated. The temporal frequency at which the correlation falls below 1/7 is taken as the resolution cut-off (can be adjusted as desired). This provides an estimate of how fast one needs to image to extract all available information.
b, Spatial maps of the highest temporal frequency at which the FTC score remained above the 1/7 across different tissues. The scores were computed on 45 min datasets. We note that this provides a lower bound: organs may exhibit higher frequencies that can be resolved but that were not observed in the experiment. Neurons and muscles (orange trace in a) exhibited as expected high-frequency activity, whilst the kidney (blue trace in a) only had resolvable power in the lower temporal frequencies.

Coherence vs. correlation clustering (EXPANDED - Extended Data Figure 5): The reviewer asked whether coherence-based clustering is quantitatively better than correlation-based clustering. We want to be transparent: we needed a principled method for dimensionality reduction, chose spectral clustering as a well-established approach, and found that both coherence and correlation metrics can work and that they emphasize different features.

Why we needed clustering: With thousands of cellular segments per dataset, we required a systematic method to group cells into interpretable functional tissue ensembles for analysis and visualization. Spectral clustering is a principled and established clustering approach.

Why we chose coherence: We observed traveling waves and temporally structured activity across many tissues (kidney nephron waves in Figure 1j-k, ependymal waves in Figure 3j). Coherence-based spectral clustering is specifically designed to capture functional coupling that includes temporal delays and filtering relationships, making it well-suited for these phenomena. Our codebase includes both coherence and correlation options. *Quantitative comparison*: We created a new toy example demonstrating when using coherence can be useful (Extended Data Figure 5a-c): coherence identifies clusters defined by temporal structure (delays, differentiation, resonance), while correlation identifies synchronous co-

activation. When we computed cluster separability metrics on our biological data, scores were similar, likely because our data contains a broad range of relationships (synchronous, delayed, propagating). *On "better" clustering*: We had made sure in the original text to never use the word "better". The reviewer notes that examples like the kidney being split into multiple clusters versus a single cluster make it "hard to say which is better." We fully agree, and thus had made sure to never say "better" in the text — as this depends on the scientific question: To identify traveling waves, grouping all components together is useful; for identifying the individual units of the kidney, breaking up the traveling wave can be of value. Nonetheless, we had written "Coherence is a frequency-based analogue to correlation, which is invariant to phase lags and thus better groups cells engaged in spatiotemporally coupled activity" - we agree that this is inaccurate and should say 'temporally coupled lagged activity' which we have now corrected. We have also updated the main text to clarify that spectral clustering with correlation also produces anatomically meaningful clusters, and that our choice of coherence specifically emphasizes traveling waves and temporally structured activity rather than being universally superior. *On anatomical fidelity claims*: We had written in the supplemental text "The clusters using coherence tend to be more compact allowing for easier anatomical identification". The reviewer rightly notes this would require substantial backing. Moreover, given the some cell types are distributed over large areas, compactness is a poor proxy for anatomical fidelity. We had used this property to highlight one population that is known to be compact (main sympathetic ganglion), however this is not generally an appropriate measure of cluster quality. We thank the reviewer for highlighting this. In general, we found the results using coherence to often be more interpretable, and this is why we used it; that said, ground truth for "correct" functional parcelation is, for the most part still an area of research, and metrics like spatial compactness can be misleading since functional ensembles (e.g., distributed neural circuits) need not be spatially compact, thus we removed the panels in Extended Data Figure 4 relating to cluster compactness.

We hope that this explains our methodological choice as one guided for the specific biological phenomena we investigated, and that our clarifications in the text better reflect that this is one valid approach among several.

We have revised the main section of the text with the following (line 154):

> *"To this end, we implemented spectral clustering, a principled approach for grouping cells with related activity patterns[22]. We used coherence as the similarity metric, a frequency-based measure that, unlike correlation, is invariant to phase lags and thus groups cells engaged in temporally structured activity such as traveling waves (Figure 1j, see Methods). We note that using correlation as the similarity metric is equally sensible; our choice of coherence was motivated by our interest in capturing delayed activity patterns prevalent in our data (Extended Data Figure 5). "*

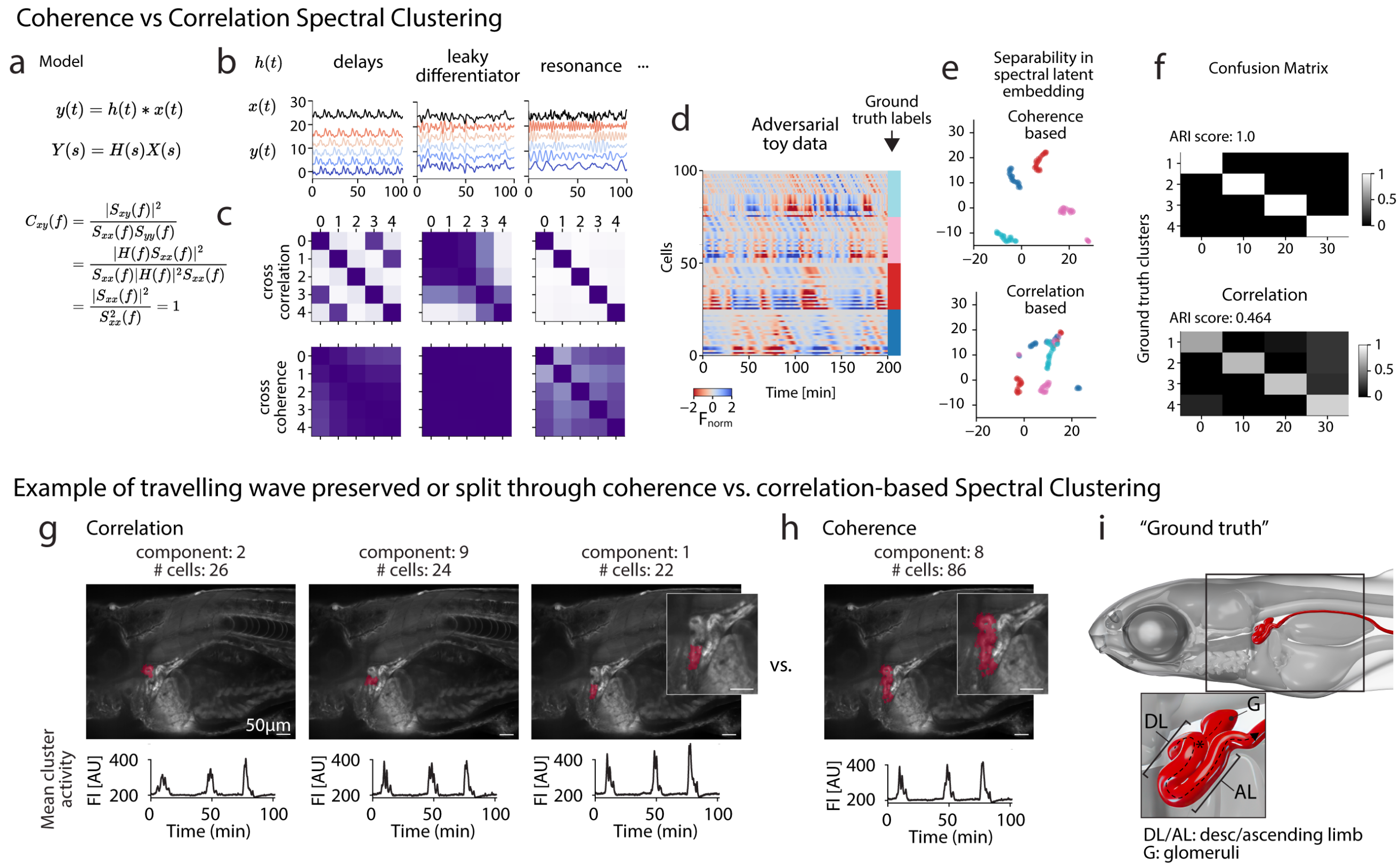

Extended Data Fig. 5: **Coherence vs. correlation-based spectral clustering.**
a, Any two signals $y(t)$, $x(t)$ which are linearly filtered version of each other will have a coherence of one. Definition of coherence.
b, Example of time-series which are coherent but not necessarily correlated. Timeseries $y(t)$ are filtered versions of $x(t)$, illustrating delay lines (left), leaky differentiators (center) and resonance (right).
c, Cross-coherence vs. cross-correlation matrix of signals in b. Coherence better captures the relationship between these timeseries.
d, Adversarial toy data constructed similarly as in b). The data consists of 4 clusters of noisy coherent timeseries. Left: ground truth labels of cluster identity.
e, Spectral latent embedding. Embedding of timeseries using coherence-based clustering (top) or correlation-based clustering.
f, Confusion matrix. Results of coherence vs. correlation-based clustering on adversarial toy data fit with 4 clusters. Values correspond to fraction of cells that were correctly assigned. ARI (adjusted random score): 1 for coherence, and 0.464 for correlation.
g, Comparison of coherence vs. correlation-based spectral clustering. Using correlation as a metric, the descending nephron is grouped into a single cluster. Anatomical footprint of clusters (red) superimposed on anatomical reference (gray). Mean cluster activity trace (bottom).
h, Using coherence as a metric, the descending nephron is grouped into a single cluster. Anatomical footprint of clusters (red) superimposed on anatomical reference (gray). Mean cluster activity trace (bottom).
i, Model of zebrafish nephron based on WB-ExM-Histo data (same as in Fig. 13).

Clarifications on existing content: Regarding imaging parameters, the mounting orientation has now been clarified in the figure caption of Figure 1:

*"**b**, Transgenic zebrafish (7 days post-fertilization) expressing GCaMP7f pancellularly (Tg(ubi:tTA;TRE:GCaMP7f)), imaged with spinning-disk confocal microscopy. For optimal access to the visceral organs, samples are mounted on their side."*

In line we the reviewer, we did not want to mislead the reader regarding volume imaged and so we had also made sure that the light source in Figure 1a only illuminates the part of the animal that we image to highlight that more experiments are focused on imaging the anterior 1/3 of the animal where most

organs are located. We had also made sure to represent the acquisition paradigm in the first figure with representative area covered (Figure 1e). Within this subpanel we alse included a cartoon of the fish with an outline of area imaged to make sure that the acquired volume is clear and doesn't rely on the reader being familiar with zebrafish anatomy.

We hope that these additions, particularly the systematic benchmarking and quantitative resolution characterizations, substantially address the reviewer's comments about appropriately characterizing methodological improvements.

In summary, we have substantially strengthened the quantitative characterization of WHOLISTIC's methodology and thank the review for helping us improve our manuscript. We present below the updated and new Extended Data Figures that we have included in the manuscript to address the reviewers feedback:

Minor Concerns (Reviewer #4)

Reviewer Comment:

Line 143: I do not support referring to the calcium dynamics as 'cellular activity'. For neurons this is already established, but there are many things that could constitute activity in general in cells, and as the authors themselves present in the discussion, many other reporters could be used. I strongly encourage the change to Ca activity' or calcium activity'. Even though this may be slightly more tedious, I believe it is scientifically more appropriate.

Our Response:

We agree with the reviewer that "cellular activity" is imprecise. We have systematically reviewed the manuscript and changed all instances of "cellular activity" to "calcium activity" or "calcium dynamics" to maintain scientific precision, including on line 145 (corresponding to line 143 of the initial manuscript). We thank the reviewer for this suggestion, which improves clarity and avoids overgeneralizing from calcium measurements to overall cellular state.

Reviewer Comment:

In going from equation (26) to equation (27), the sum in the first term disappears and becomes 1. Should this be an n?

Our Response:

Thank you for catching this. The transition from equation (26) to equation (27) assumes pre-normalized vectors, which was stated in the text preceding the equation, which is the origin of the 1. We agree that this wasn't the clearest way of outlining the derivation, therefore we have changed the order so that it is more explicitly stated (line 1938):

"The correlation between vectors $\boldsymbol{x} \in R^N$ *and* $\boldsymbol{y} \in R^N$ *being defined as:*

$$\mathcal{R}(\boldsymbol{x}, \boldsymbol{y}) = \frac{\sum_i x_i \cdot y_i}{\sqrt{\sum_i x_i^2}\sqrt{\sum_i y_i^2}} \tag{2}$$

We assume already pre-normalized vectors:

$$x_i = \frac{x_i}{\sqrt{\sum_j x_j^2}} \tag{3}$$

$$y_i = \frac{y_i}{\sqrt{\sum_j y_j^2}} \tag{4}$$

Then:

$$\begin{aligned} |\boldsymbol{x} - \boldsymbol{y}|_2^2 &= 2\sum_i (1 - x_i \cdot y_i) & (5) \\ &= 2(1 - \sum_i (x_i \cdot y_i)) & (6) \\ &= 2(1 - \mathcal{R}(\boldsymbol{x}, \boldsymbol{y})) & (7) \end{aligned}$$

"

Reviewer Comment:

Line 1068: does psd mean positive semi-definite?

Our Response:

We apologize for accidentally including an unexplained abbreviation. We indeed meant "positive semi-definite" and have now spelled this out in full throughout the manuscript for improved clarity.

Reviewer Comment:

Could the authors provide full stacks of their new generated transgenic lines?

Our Response:

Thank you for this suggestion. We now include full image stacks demonstrating the expression patterns of the newly generated transgenic lines, which are now available through the accompanying website, https://wholistic.janelia.org/data/ and referenced in the Supplementary Notes.

Reviewer Comment:

Overall, I believe that the amount of work that has gone into this study is astounding and I congratulate the authors on it, even if it leaves me slightly confused as to what the message is that I should be taking home.

Our Response:

We thank the reviewer again for their recognition of the extensive work in this study and all of their constructive feedback. We hope that our revised manuscript, with strengthened quantitative characterization of the methodology and clearer articulation of the biological discoveries enabled by WHOLISTIC, better conveys the aim of the paper.

References

[1] Michael G. Jonz. Respiratory System. In *The Zebrafish in Biomedical Research*, pages 103–107. Elsevier, 2020. ISBN 978-0-12-812431-4. doi: 10.1016/B978-0-12-812431-4.00010-5. URL `https://linkinghub.elsevier.com/retrieve/pii/B9780128124314000105`.

[2] Marco dal Maschio, Joseph C. Donovan, Thomas O. Helmbrecht, and Herwig Baier. Linking Neurons to Network Function and Behavior by Two-Photon Holographic Optogenetics and Volumetric Imaging. *Neuron*, 94(4):774–789.e5, May 2017. ISSN 0896-6273. doi: 10.1016/j.neuron.2017.04.034. URL `https://www.sciencedirect.com/science/article/pii/S0896627317303628`.

[3] Jason Manley and Alipasha Vaziri. Whole-brain neural substrates of behavioral variability in the larval zebrafish. *eLife*, 13, May 2025. doi: 10.7554/eLife.97014.2. URL `https://elifesciences.org/reviewed-preprints/97014`. Publisher: eLife Sciences Publications Limited.

[4] John C. Davis. Minimal Dissolved Oxygen Requirements of Aquatic Life with Emphasis on Canadian Species: a Review. *Journal of the Fisheries Research Board of Canada*, 32(12):2295–2332, December 1975. ISSN 0015-296X. doi: 10.1139/f75-268. URL `http://www.nrcresearchpress.com/doi/10.1139/f75-268`.

[5] Erik R Swenson. The many acid–base manifestations and consequences of hypoxia. *Current Opinion in Physiology*, 7:72–81, February 2019. ISSN 24688673. doi: 10.1016/j.cophys.2019.01.003. URL https://linkinghub.elsevier.com/retrieve/pii/S2468867319300033.

[6] Payel Banerjee, Paul Joly, Luc Jouneau, Yan Jaszczyszyn, Mickaël Bourge, Pierre Affaticati, Jean Pierre Levraud, Pierre Boudinot, and Jean Stéphane Joly. Molecular and Cellular Analysis of the Repair of Zebrafish Optic Tectum Meninges following Laser Injury. *Cells*, 11(13), 2022. ISSN 20734409. doi: 10.3390/cells11132016.

[7] Alexei Verkhratsky and Arthur M. Butt. Chapter 3 - Astroglial physiology. In Alexei Verkhratsky and Arthur M. Butt, editors, *Neuroglia*, pages 89–197. Academic Press, 2023. ISBN 978-0-12-821565-4. doi: https://doi.org/10.1016/B978-0-12-821565-4.00009-2. URL https://www.sciencedirect.com/science/article/pii/B9780128215654000092.

[8] Nicholas James Sofroniew, Daniel Flickinger, Jonathan King, and Karel Svoboda. A large field of view two-photon mesoscope with subcellular resolution for in vivo imaging. *eLife*, 5(JUN2016):1–20, 2016. ISSN 2050084X. doi: 10.7554/eLife.14472.

[9] Dal Hyung Kim, Jungsoo Kim, João C. Marques, Abhinav Grama, David G. C. Hildebrand, Wenchao Gu, Jennifer M. Li, and Drew N. Robson. Pan-neuronal calcium imaging with cellular resolution in freely swimming zebrafish. *Nature Methods*, 14(11):1107–1114, November 2017. ISSN 1548-7105. doi: 10.1038/nmeth.4429. URL https://www.nature.com/articles/nmeth.4429. Publisher: Nature Publishing Group.

[10] Manuel Vicente, Jussep Salgado-Almario, Ariel A. Valiente-Gabioud, Michelle M. Collins, Pierre Vincent, Beatriz Domingo, and Juan Llopis. Early calcium and cardiac contraction defects in a model of phospholamban R9C mutation in zebrafish. *Journal of Molecular and Cellular Cardiology*, 173:127–140, December 2022. ISSN 0022-2828. doi: 10.1016/j.yjmcc.2022.10.005. URL https://www.sciencedirect.com/science/article/pii/S0022282822005399.

[11] Phong D. Nguyen, Iris Gooijers, Giulia Campostrini, Arie O. Verkerk, Hessel Honkoop, Mara Bouwman, Dennis E. M. de Bakker, Tim Koopmans, Aryan Vink, Gerda E. M. Lamers, Avraham Shakked, Jonas Mars, Aat A. Mulder, Sonja Chocron, Kerstin Bartscherer, Eldad Tzahor, Christine L. Mummery, Teun P. de Boer, Milena Bellin, and Jeroen Bakkers. Interplay between calcium and sarcomeres directs cardiomyocyte maturation during regeneration. *Science*, 380(6646):758–764, May 2023. doi: 10.1126/science.abo6718. URL https://www.science.org/doi/10.1126/science.abo6718. Publisher: American Association for the Advancement of Science.

[12] Jussep Salgado-Almario, Manuel Vicente, Yillcer Molina, Antonio Martinez-Sielva, Pierre Vincent, Beatriz Domingo, and Juan Llopis. Simultaneous imaging of calcium and contraction in the beating heart of zebrafish

larvae. *Theranostics*, 12(3):1012–1029, January 2022. ISSN 1838-7640. doi: 10.7150/thno.64734. URL https://pmc.ncbi.nlm.nih.gov/articles/PMC8771564/.

[13] Sami Koho, Giorgio Tortarolo, Marco Castello, Takahiro Deguchi, Alberto Diaspro, and Giuseppe Vicidomini. Fourier ring correlation simplifies image restoration in fluorescence microscopy. *Nature communications*, 10(1): 3103, 2019.

[14] Emmanuelle Steib, Rob Tetley, Romain F. Laine, Dominic P. Norris, Yanlan Mao, and Julien Vermot. TissUExM enables quantitative ultrastructural analysis in whole vertebrate embryos by expansion microscopy. *Cell Reports Methods*, 2(10):100311, 2022. ISSN 26672375. doi: 10.1016/j.crmeth.2022.100311. URL https://doi.org/10.1016/j.crmeth.2022.100311.

[15] Davide Gambarotto, Fabian U. Zwettler, Maeva Le Guennec, Marketa Schmidt-Cernohorska, Denis Fortun, Susanne Borgers, Jörn Heine, Jan-Gero Schloetel, Matthias Reuss, Michael Unser, Edward S. Boyden, Markus Sauer, Virginie Hamel, and Paul Guichard. Imaging cellular ultrastructures using expansion microscopy (U-ExM). *Nature Methods*, 16(1):71–74, January 2019. ISSN 1548-7105. doi: 10.1038/s41592-018-0238-1. URL https://www.nature.com/articles/s41592-018-0238-1. Publisher: Nature Publishing Group.

[16] Dylan J. M. Bergen, Erika Kague, and Chrissy L. Hammond. Zebrafish as an Emerging Model for Osteoporosis: A Primary Testing Platform for Screening New Osteo-Active Compounds. *Frontiers in Endocrinology*, 10:6, January 2019. ISSN 1664-2392. doi: 10.3389/fendo.2019.00006. URL https://www.ncbi.nlm.nih.gov/pmc/articles/PMC6361756/.

[17] Yu Mu, Davis V. Bennett, Mikail Rubinov, Sujatha Narayan, Chao Tsung Yang, Masashi Tanimoto, Brett D. Mensh, Loren L. Looger, and Misha B. Ahrens. Glia Accumulate Evidence that Actions Are Futile and Suppress Unsuccessful Behavior. *Cell*, 178(1):27–43.e19, 2019. ISSN 10974172. doi: 10.1016/j.cell.2019.05.050.

[18] Jeffrey Demas, Jason Manley, Frank Tejera, Kevin Barber, Hyewon Kim, Francisca Martínez Traub, Brandon Chen, and Alipasha Vaziri. High-speed, cortex-wide volumetric recording of neuroactivity at cellular resolution using light beads microscopy. *Nature methods*, 18(9):1103–1111, September 2021. ISSN 1548-7091. doi: 10.1038/s41592-021-01239-8. URL https://pmc.ncbi.nlm.nih.gov/articles/PMC8958902/.

[19] Oleg I. Rumyantsev, Jérôme A. Lecoq, Oscar Hernandez, Yanping Zhang, Joan Savall, Radosław Chrapkiewicz, Jane Li, Hongkui Zeng, Surya Ganguli, and Mark J. Schnitzer. Fundamental bounds on the fidelity of sensory cortical coding. *Nature*, 580(7801):100–105, April 2020. ISSN 0028-0836, 1476-4687. doi: 10.1038/s41586-020-2130-2. URL https://www.nature.com/articles/s41586-020-2130-2.

[20] Raju Tomer, Khaled Khairy, Fernando Amat, and Philipp J Keller. Quantitative high-speed imaging of entire developing embryos with simultaneous multiview light-sheet microscopy. *Nature Methods*, 9(7):755–763,

July 2012. ISSN 1548-7091, 1548-7105. doi: 10.1038/nmeth.2062. URL https://www.nature.com/articles/nmeth.2062.

[21] Gustavo de Medeiros, Nils Norlin, Stefan Gunther, Marvin Albert, Laura Panavaite, Ulla-Maj Fiuza, Francesca Peri, Takashi Hiiragi, Uros Krzic, and Lars Hufnagel. Confocal multiview light-sheet microscopy. *Nature Communications*, 6(1):8881, November 2015. ISSN 2041-1723. doi: 10.1038/ncomms9881. URL https://www.nature.com/articles/ncomms9881. Publisher: Nature Publishing Group.

[22] Ulrike Von Luxburg. A tutorial on spectral clustering. *Statistics and Computing*, 17(4):395–416, 2007. ISSN 09603174. doi: 10.1007/s11222-007-9033-z.

Response to Reviewer:

We thank the Reviewer for assessing the manuscript and providing careful and useful feedback, especially regarding the pronephric system.

Detailed responses:

This study developed the WHOLISTIC technique, which aims to image calcium activity in real time across most cells in living zebrafish embryos. The research revealed multiple cross-organ coordination phenomena, including pulsatile traveling waves in the proximal tubule of the nephron, ultraslow oscillations in ependymal cells during quiescence, rapid responses of chondrocytes to cold stimulation, and brainstem-controlled redistribution of blood flow under hypoxic conditions. The image processing method presented here is significant for capturing rapid signaling dynamics in living organisms, though it also has certain limitations.

1. Figure 1h shows the proximal tubule of the zebrafish embryonic pronephros, not the entire nephron. This should be corrected by the authors.

Thank you for pointing out this inaccuracy; we have updated both the label on Figure 1h and its associated legend to reflect the more precise nomenclature suggested by the reviewer (glomerulus and proximal tubule).

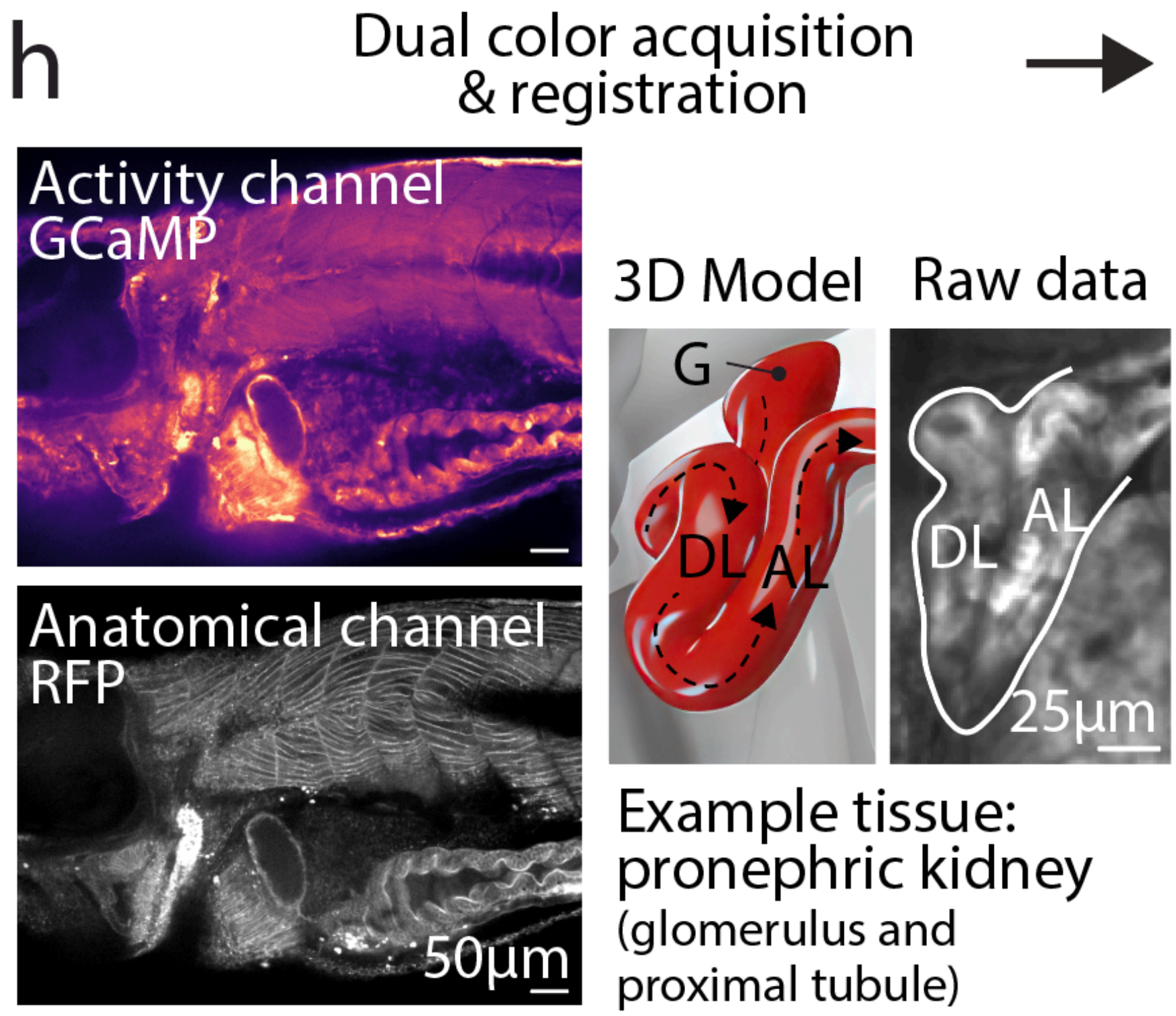

Updated Fig 1. h, Left: dual-color imaging combining pancellular sensor line (e.g., GCaMP7f) with membrane-targeted anatomical reference line. Right: example tissue: inset showing 3D model and enlarged view of the anterior part of the pronephric kidney (glomerulus and proximal tubules - G: glomerulus, DL: descending limb, AL: ascending limb).

2. The glomerulus is located below the proximal tubule shown in Fig. 1h, not at the position indicated by "G" in the figure. This raises an important technical question: can the method described by the authors adequately resolve deep organs and tissues? What is the maximum imaging depth that this method can simultaneously achieve? If the thickness that can be acquired in a single volume is limited, is the method still sufficient to simultaneously resolve most tissues and organs of the zebrafish embryo?

The 3D model in Fig. 1h was derived exclusively from whole-body expansion microscopy (ExM) data and therefore has no bearing on the depth-dependence of WHOLISTIC functional imaging resolution. We therefore address the Reviewer's two concerns separately.

Imaging depth and resolution: Depth- and location-dependence of functional imaging resolution is now quantified in Extended Data 1e (added in response to a related query from another reviewer), which demonstrates reliable cellular resolution up to 130 µm. The glomerulus, lying near the midline, is among the more challenging structures to capture, but it is labelled and resolvable with the 40x 1.15 NA objective used in Fig. 3e (example confocal data are shown below). As noted in the Discussion, two-photon imaging (in place of spinning-disk confocal) will further extend access to deep structures.

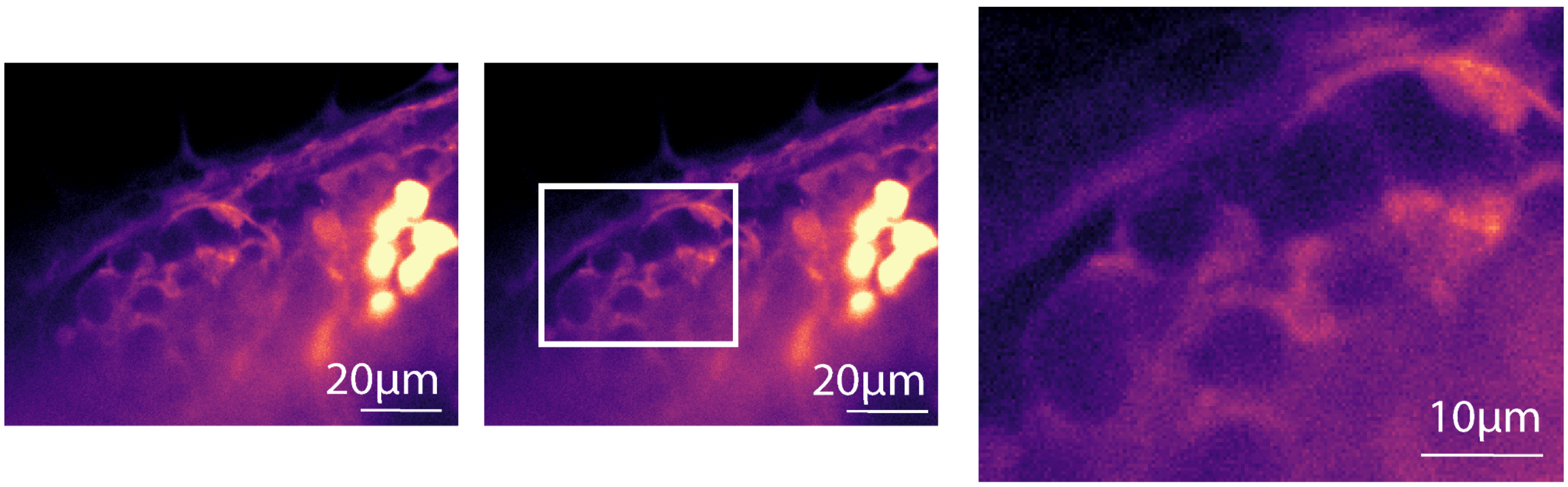

Figure R1. Visualization of the region of the larval zebrafish glomeruli.
Spinning-disk confocal image of a pancellular GCaMP7f line, Tg(ubi:tTA;TRE:GCaMP7f), acquired with a 40x 1.15 NA objective at the midline of the animal.
(a) Example field showing a section of the glomerulus area (left), which appears as a single large cluster of rounded, lobulated profiles, alongside nearby active sympathetic ganglia (bright cells, right). The dark band across the upper portion is the notochord, whose vacuolated cells are largely non-fluorescent. Scale bar, 20 µm.
(b) Same field as (a); white box indicates the zoomed-in region of the glomerulus shown in (c).
(c) Enlarged view of the boxed region in (b), resolving ring-like profiles with darker lumina, together forming a lobulated structure. Cellular-scale detail is visible. Scale bar, 10 µm.

Position of the glomerulus relative to the proximal tubule: Reviewing our anatomical data and open-access data, we find that the dorsal tip of the glomerulus is at the same level as and slightly dorsal to, the dorsal tip of the proximal tubule (data sources examples: https://zfin.org/zf_info/anatomy/120hrs/S075.html, https://zf.hms.harvard.edu/). That said, the 3D model was built from multiple ExM datasets in which different anatomical structures (vasculature, autonomic nervous

system, ventricles) were separately labelled and then manually aligned, a step that could introduce small alignment error. We noted that while the Methods section describes the constituent datasets, it did not provide detail about how these were aggregated into a single model; we have expanded this section to specify that this was done manually based on the total protein channel present in all datasets,

"Meshes from different samples were manually aligned in Blender to a common reference space using the total protein stain present in all datasets."

We have made and included new renderings of the nephron to better convey the 3D organization of this structure. We hope we have understood the Reviewer's concern and that these items will address it.

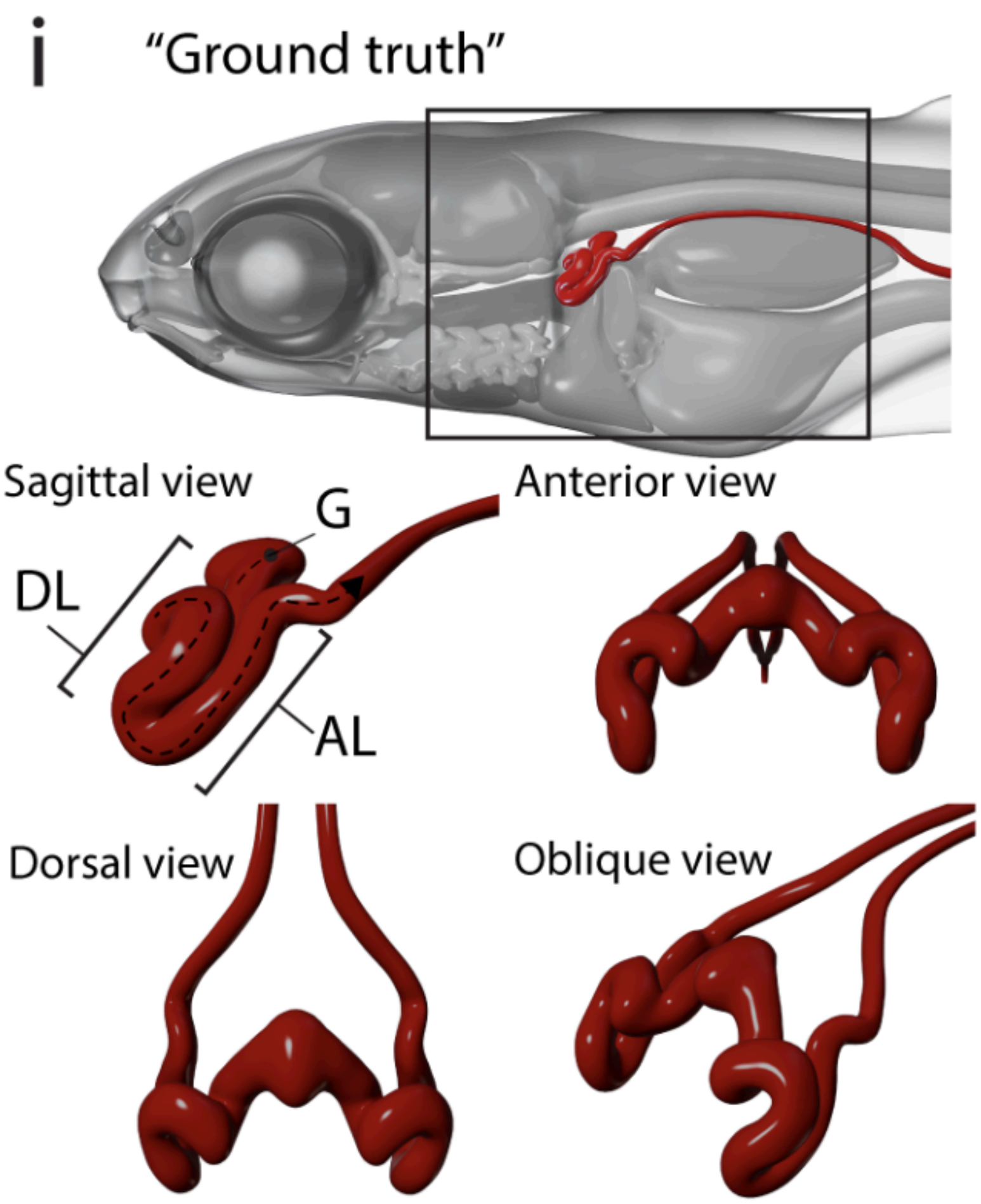

Updated Extended Data Fig 5. i, Model of pronephric kidney (glomerulus and proximal tubules - G: glomerulus, DL: descending limb, AL: ascending limb) based on WB-ExM-Histo data viewed from multiple angles.

3. Furthermore, many tissues are composed of thin layers of tightly adhered cells, such as vascular endothelial cells and pericytes, as well as glomerular vascular endothelial cells, podocytes, and mesangial cells. In these tissues, individual cell types often require specific transgenic lines to be distinguished. This raises another question: is the method in this study sufficient to resolve tightly adhered, thin-layered cellular structures?

We were also very interested in this question and included two relevant proofs of principle showing that it is indeed possible. First, we identified the brain's meninges, a thin tissue of exactly this kind; functional imaging and analysis allowed this layer to be extracted by virtue of calcium signals co-varying across meningeal cells, despite its thinness (Extended Data Fig. 4). Second, we resolved the ependymal cells (Figure 3), a single-cell layer lining the ventricles and central canal, including activity along their thin ventrally projected processes, despite the fact that they were not specifically labelled. We note that our segmentation and clustering are activity-based, so identifying a thin or sparse population requires its activity to be distinguishable from that of neighboring cells. We cannot assume this holds for every thin tissue, and some may indeed require secondary transgenic labelling. Nonetheless, these two cases demonstrate that WHOLISTIC functional imaging is in principle capable of resolving thin-layered structures in functional imaging data.

4. Using higher temporal resolution, this study identified pulsatile traveling waves in the proximal tubule. The proximal tubule can be segmented into continuous functional ensembles, each exhibiting distinct dynamic characteristics. This is a very interesting finding that provides an important tool for studying renal tubule function. The authors suggest that this indicates kidney filtration may in fact be intermittent and pulsatile on shorter timescales. This speculation may be debatable, because this part only labels the proximal tubule, not the glomerulus, which is responsible for filtration. These pulsatile traveling waves might instead be related to proximal tubule epithelial cells driving the flow of intraluminal fluid. Of course, all these speculations require further validation through separate, dedicated studies.

We are grateful for these insights and will correct the text to communicate these uncertainties and need for further experimentation to test more specific hypotheses about this system.

"The spatial propagation, coupled with the observed quiescence between bursts, suggests that kidney filtration and/or intraluminal flow – up to now measured at low temporal resolution and conceptualized as a continuous or continuously oscillating process – may in fact be intermittent and pulsatile on shorter timescales. Distinguishing these processes and confirming this interpretation will require further dedicated experiments."

5. Neuromodulation itself is a well‑recognized form of inter‑organ communication. Therefore, it might be beneficial to include additional examples of inter-organ coordination that go beyond neural or systemic stress-related stimuli.

We thank the Reviewer for raising this. We agree that inter-organ coordination extends well beyond neural signaling, and that endocrine, humoral, and neuromodulatory loops are necessary for achieving a complete picture. The present manuscript is focused on establishing the platform and demonstrating its capacity to resolve such interactions, rather than on a comprehensive survey of inter-organ signaling modalities, which we feel is beyond the scope of this work. Characterizing these additional loops at cellular resolution across organs is, however, a direction the platform is well suited to, especially in conjunction with genetically-encoded sensors for such neuromodulators, and one we are excited to pursue in future work.

6. There is a minor data writing issue: two figure legends state p = 0.3125, whereas the corresponding figure shows p = 0.03125, which appears to be a writing error. Additionally, the figure legend does not indicate what the asterisk (*) represents.

We appreciate this identification of the error and have corrected it.
